# Supplementary material for: Phylogeny of Paleozoic limbed vertebrates reassessed through revision and expansion of the largest published relevant data matrix
Source: PeerJ. 2019 Jan 4;6:e5565. doi: 10.7717/peerj.5565 (PMC6322490; doi:10.7717/peerj.5565)
Supplement: Supplemental Information 1 [file peerj-07-5565-s001.pdf]

Appendix S1: Complete list of characters and modifications to the data matrix of RC07, with reports of new observations of specimens.

The names, the abbreviations and the order of all characters and their states are unchanged from RC07 unless a change is explained. We renumbered the characters we did not delete from 1 to 277, so the character numbers do not match those of RC07. However, merged characters retain the abbreviations of all their components: PREMAX 1-2-3 (our character 1) consists of the characters PREMAX 1, PREMAX 2 and PREMAX 3 of RC07, while MAX 5/PAL 5 (our ch. 22) is assembled from MAX 5 and PAL 5 of RC07. We did not add any characters, except for splitting state 1 of INT FEN 1 into the new state 1 of INT FEN 1 (ch. 84) and states 1 and 2 of the new character MED ROS 1 (ch. 85), undoing the merger of PIN FOR 1 and PIN FOR 2 (ch. 91 and 92) and splitting state 0 of TEETH 3 into the new state 0 of TEETH 3 (ch. 183) and the entire new character TEETH 10 (ch. 190). A few characters have additional states or are recoded in other ways. Deleted characters are retained here, together with the reasons why we deleted them and the changes we made to their scores.

All multistate characters mention in their names whether they are ordered, unordered, or treated according to a stepmatrix.

Taxa are by default mentioned in the same order as in the matrix, at least within the same paragraph.

In the interest of making our scoring decisions transparent, the taxa we have added as separate OTUs are mentioned in cases where their scores could be controversial or, of course, when we report previously unknown scores for them based on pers. obs. of specimens. Their names are preceded by an asterisk; the names of taxa that are not included (but mentioned for comparison) are marked with two asterisks.

Similarly, scores we have decided not to change are mentioned in cases where they could be controversial. Lack of mention implies lack of change from RC07.

"*Broiliellus*" is *B. brevis* throughout; other species, including the type species *B. texensis*, were not considered (as explained in Material and methods: Treatment of OTUs: Taxa added as parts of existing OTUs).

1. PREMAX 1-2-3: **Caudodorsal process of premaxilla: broad, low, indistinct (0); alary process (vaguely triangular, not occupying entire available mediolateral width at its base) (1); moderately tall, vaguely rectangular, or acutely triangular but occupying the entire mediolateral width between the nares and the median suture at its base (2); narrow and long, along the sagittal plane or parasagittal (3) (unordered).** Within state 3, the mediolateral position of the process is not considered, because it probably depends on the width of the premaxilla.

This character changes states from 0 to 1 in the ontogeny of *Apateon gracilis* (Schoch & Fröbisch, 2006); we have tried to take this into account when scoring OTUs known only from immature or paedomorphic individuals.

RC07 treated this character complex as three separate characters: PREMAX 1, "Premaxillary alary process: absent (0); present (1)"; PREMAX 2, "Premaxilla alary process shorter than wide (0) or as long as/longer than wide (1)"; and PREMAX 3, "Premaxilla alary process less than (0) or at least one-third as wide as premaxilla (1)". Not only are PREMAX 2 and PREMAX 3 inapplicable when PREMAX 1 has state 0; they do not (even together) cover the diversity of shapes of the contact between premaxilla and nasal seen in the taxon sample. Accordingly, we have replaced all three characters by ch. 26 of Marjanović & Laurin (2009), itself based on ch. 2 of Marjanović & Laurin (2008) and the work of Good & Wake (1992).

Crassigyrinus and *Microbrachis* were scored for PREMAX 2 and PREMAX 3 by RC07 in spite of being also scored PREMAX 1(0), which, as mentioned, made PREMAX 2

and PREMAX 3 inapplicable. Some OTUs were scored for the latter two characters in spite of being also scored PREMAX 1(?), which had the same effect.

State 0 occurs in *Eusthenopteron* (Jarvik, 1967; Brazeau & Ahlberg, 2006), *Panderichthys* (Vorobyeva & Schultze, 1991), *Ventastega* (Ahlberg et al., 2008), *Acanthostega* (Clack, 2007), *Ichthyostega* (Clack & Milner, 2015: fig. 8), *Whatcheeria* (Lombard & Bolt, 1995), *Baphetes* and *Megalocephalus* (Beaumont, 1977), *Eucritta* (Clack, 2001 – it looks like 2 in the reconstruction, but the photo strongly suggests the pieces of bone in question are median rostrals rather than processes of the premaxilla), *Chenoprosopus* (Langston, 1953), *Cochleosaurus* (Sequeira, 2004), *Neldasaurus* (Chase, 1965), *Caerorhachis* (Ruta, Milner & Coates, 2002 – though 1 and 2 would also be more or less defensible scores), *Eoherpeton* (Smithson, 1985, though this is somewhat arguable), *Proterogyrinus* (Holmes, 1984), *Archeria* (Holmes, 1989), *Discosauriscus* (Klembara & Ruta, 2005a), *Keraterpeton*, *Batrachiderpeton*, *Ptyonius* and *Sauropleura* (Bossy & Milner, 1998), *Ariekanerpeton* (Laurin, 1996b; Klembara & Ruta, 2005a) and *Utegenia* (Laurin, 1996c; Klembara & Ruta, 2004a). We have further assigned state 0 to the frogs *Notobatrachus* (Báez & Nicoli, 2004), *Vieraella* (Báez & Basso, 1996) and **Liaobatrachus* (Dong et al., 2013): their so-called alary processes are entirely ventral to the nares and are laterally bordered by a neomorphic fenestra that is more or less confluent with the naris on the same side.

Colosteus and *Greererpeton* are here tentatively scored 1 (Smithson, 1982; Hook, 1983; see Bolt & Lombard, 2010, for grounds for caution). State 1 further occurs in *Isodectes* (Sequeira, 1998), *Trimerorhachis* (Schoch & Milner, 2013), *Balanerpeton* (Milner & Sequeira, 1994), Dendrerpetidae (Holmes, Carroll & Reisz, 1998), *Eryops*, *Acheloma* (Polley & Reisz, 2011 – although it is arguably a special case), *Ecolsonia* (Berman, Reisz & Eberth, 1985), *Amphibamus* (Schoch, 2001), *Doleserpeton* (Bolt, 1969; Sigurdsen & Bolt, 2010), *Platyrhinops* (Clack & Milner, 2010), *Micromelerpeton* (arguably borderline to state 3: Schoch, 2009), *Apateon* (Schoch & Fröbisch, 2006), *Karaurus* (Ivachnenko, 1978: fig. 1; D. M. and M. L., pers. obs. of unnumbered MNHN cast of PIN 2585/2), *Dolichopareias* (Andrews & Carroll, 1991), *Capetus* (Sequeira & Milner, 1993) and, perhaps surprisingly, **Utaherpeton* (Carroll, Bybee & Tidwell, 1991: fig. 6.1, 7).

State 2 occurs in *Tulerpeton* (as far as can be told from the isolated premaxilla + vomer; Lebedev & Clack, 1993), *Crassigyrinus* (Clack, 1998), Albanerpetidae, *Eocaecilia* (Jenkins, Walsh & Carroll, 2007), *Pholiderpeton attheyi* (Panchen, 1972), *Anthracosaurus* (Clack, 1987a), *Pholiderpeton scutigerum* (Clack, 1987b), *Bruktererpeton* (Boy & Bandel, 1973), *Gephyrostegus* (Carroll, 1970; Klembara et al., 2014), *Seymouria* (Laurin, 1996a; see Marjanović & Laurin [2009: Electronic Supplementary Material 1] for discussion), *Captorhinus* (Fox & Bowman, 1966; Heaton, 1979), *Petrolacosaurus* (Reisz, 1981), *Westlothiana* (Smithson et al., 1994), *Batropetes* (Carroll, 1991; Glienke, 2013), *Tuditanus*, *Pantylus*, *Asaphestera*, *Saxonerpeton*, *Hapsidopareion*, *Micraroter*, *Pelodosotis*, *Cardiocephalus*, *Hyloplesion* and *Odonterpeton* (CG78), *Rhynchonkos* (CG78; Szostakiwskyj, Pardo & Anderson, 2015), *Microbrachis* (Vallin & Laurin, 2004), *Stegotretus* (Berman, Eberth & Brinkman, 1988), *Diceratosaurus*, *Diplocaulus*, *Diploceraspis*, *Scincosaurus*, *Adelogyrinus*, *Silvanerpeton* (Ruta & Clack, 2006) and *Tseajaia* (Moss, 1972).

State 3 occurs in *Phonerpeton* (Dilkes, 1990: fig. 3), *Eoscopus* (Daly, 1994), *Valdotriton* (Evans & Milner, 1996), *Diadectes* (Berman, Sumida & Martens, 1998), *Limnoscelis* (Reisz, 2007; Berman, Reisz & Scott, 2010), *Lethiscus* (Pardo et al., 2017) and *Orobates* (Berman et al., 2004).

Unknown (but not scored as such by RC07) in *Edops* (Romer & Witter, 1942 – the area is reconstructed in MCZ 1378: D. M., pers. obs.), *Leptorophus* and *Schoenfelderpeton* (Boy, 1986, 1987), *Triadobatrachus* (Roček & Rage, 2000), *Solenodonsaurus* (Laurin & Reisz, 1999), *Kotlassia* (Bulanov, 2003), *Paleothyris* (Carroll, 1969b), *Adelospondylus*

(Andrews & Carroll, 1991), *Urocordylus* (Bossy & Milner, 1998), *Leptoropha* & *Microphon* (Bulanov, 2003 – known to be 0 in juvenile skulls, but this could be ontogenetic), *Ossinodus* (Warren, 2007), *Pederpes* (Clack & Finney, 2005); also unknown in **Gerobatrachus* (Marjanović & Laurin, 2009: Electronic Supplementary Material 1).

Euryodus is polymorphic, possessing states 0 and 2 (CG78).

Brachydectes has state 1 or 2 (Wellstead, 1991; Pardo & Anderson, 2016).

Oestocephalus (specimen drawings in Carroll, 1998a) and *Phlegethontia* (Anderson, 2007a) have state 2 or 3.

**Acanthostomatops* is polymorphic, in at least one case (Witzmann & Schoch, 2006a: fig. 3D) showing states 1 and 2 on different sides of the same individual.

States 1 and 3 occur in "large adults" of **Glanochthon* (Schoch & Witzmann, 2009b: fig. 2).

The premaxillae of **Quasicaecilia* are unknown, but the nasals make state 3 impossible (Pardo, Szostakiwskyj & Anderson, 2015); we have thus scored state 0, 1 or 2.

2. PREMAX 4: **Premaxilla with flat, expanded anteromedial dorsal surface and elongated along its lateral margin but not along its medial margin, when observed in dorsal aspect: absent (0); present (1).** This character is a case of non-additive binary coding; it is likely that dividing state 0 (which is defined only as everything that is not state 1) into several states would reveal further phylogenetic signal.

State 0 is present in *Ventastega* (Ahlberg, Lukševičs & Lebedev, 1994). We have kept it for *Acanthostega*, *Ichthyostega* and the colosteids because the anteromedial surface is probably not "expanded", although the lateral margin is much longer than the medial one (Clack, 2007; Bolt & Lombard, 2010; Porro, Rayfield & Clack, 2015; Clack & Milner, 2015); the same holds for **Elginerpeton* (Ahlberg, 1995).

State 1 is, however, present in *Phlegethontia* (Anderson, 2007a).

This character is invisible in **Saharastega*; there are so many fractures that each of them could be a suture (D. M., pers. obs. of MNN MOR 73).

3. PREMAX 7: **Ratio of maximum width of both premaxillae together to maximum width of skull roof: ≥ 0.5 (0);]0.5-0.4] (1);]0.4-0.3] (2);]0.3-0.2] (3); < 0.2 (4) (ordered).** The wording of this character, but not its scores, changed substantially between Ruta, Coates & Quicke (2003) and RC07. Ruta, Coates & Quicke (2003) put it as follows:

"PREMAX 7. *Premaxillae more (0) or less than (1) two-thirds as wide as skull.* This is a modified version of one of Gauthier et al.'s (1988b) characters, found ubiquitously among 'reptiliomorphs', and which characterizes also several lepospondyls and some primitive lissamphibians (e.g. *Eocaecilia*; Carroll, 2000). Narrow premaxillae, even in stem-amniotes showing broad and spade-shaped snouts (e.g. *Discosauriscus*; Klembara, 1997), contrast with the broad premaxillae of temnospondyls and several stem-tetrapods."

Compare RC07:

"PREMAX 7. *Premaxillae more (0) or less than (1) two-thirds as wide as skull table.* The total width of the conjoined premaxillae is measured as the distance between their lateralmost extremities; the total skull table width is between the lateral extremities of the suspensoria."

Thus, the 2007 version described the width of the skull, but called it "skull table width". This appears to be yet another instance of the constant confusion of the terms "skull roof" and "skull table" by RC07 (see main text). However, we have measured the total width of the premaxillae ("between their lateralmost extremities") and the maximum width of the skull roof ("between the lateral extremities of the suspensoria") in all taxa in this matrix (Data S5); it turns out that a ratio of 0.67 or more **does not occur in the original or the expanded**

taxon sample – except in *Caerorhachis* (Ruta, Milner & Coates, 2002), which was scored as unknown in RC07. Only 11 OTUs even reach a ratio of 0.5. Moreover, the correlation between the scores by RC07 and the ratios we have calculated is quite low (Appendix-Table 1), and it does not improve if we boldly interpret "two-thirds" as "one-third" (which would give state 0 to 65 of the 127 measurable OTUs).

Because Ruta, Coates & Quicke (2003) claimed that this character carried phylogenetic signal, we did not want to simply delete it as parsimony-uninformative. We therefore speculated that the change from "skull" to "skull table" may in this case really have been a correction and measured the width of the skull table (see main text and legend to Data S5; including "tabular horns") in all taxa in this matrix as well. Indeed, 50 of 129 measurable OTUs have a ratio of two-thirds or more, but the correlation between the ratios we have found and the scores by RC07 remains extremely low (Data S5).

Except for a few cases of missing data, RC07 assigned state 1 to all amniotes, diadectomorphs, "microsaurs", seymouriamorphs, anthracosaurs, *Silvanerpeton*, *Gephyrostegus*, *Diplocaulus*, *Diploceraspis* and finally *Eocaecilia* (mentioned in the quote above), and state 0 to all the rest. It appears to us that they did not measure most OTUs at all, but instead scored them after presumed close relatives – which would be an example of circular logic, because those presumptions of relationship are the very hypotheses that their phylogenetic analyses aimed to test. We have therefore entirely discarded the original scores and state definitions.

Regardless of whether the skull table or the entire skull roof is measured, this character is continuous; the question thus arises of how best to divide it into states, and whether to choose the table or the whole roof. Concerning the second question, we have arbitarily chosen the whole roof in order to conform to the definition by RC07 and the name of the 2003 version of the character. The ideal solution to the first question would be stepmatrix gap-weighting (Wiens, 2001) as used by Marjanović & Laurin (2008), but PAUP* only allows 32 states, while our matrix has 128 measurable OTUs with 122 different values (so that arbitrary binning and averaging would have to be performed), and even with "only" 32 states the calculation time would skyrocket. The character lacks large morphological gaps except toward the extremes, and it seems clear that the existing small gaps are artefacts of taxon sampling because the taxa we have added have filled many (Appendix-Table 1; Data S5). We have arbitrarily created a state for each interval of 0.1, with the two OTUs above 0.6 (*Caerorhachis*, *Crassigyrinus*) and the two below 0.1 (*Diplocaulus*, *Diploceraspis*) merged into the neighboring bins to avoid making a near-uninformative state in *Caerorhachis* and *Crassigyrinus*, and a near-uninformative state correlated with the famous boomerang shape of the skull in *Diplocaulus* and *Diploceraspis*.

The ratios, sources, and state changes are presented in Appendix-Table 1; they and our measurements are contained in Data S5.

Baphetes is polymorphic: *B. kirkbyi* has state 1 (Beaumont, 1977: fig. 21), *B. orientalis* changes from state 1 to state 0 in ontogeny (Beaumont, 1977: fig. 25; Milner, Milner & Walsh, 2009).

**Saharastega* is too poorly preserved to measure, but states 0 and 4 can still be excluded (D. M., pers. obs. of MNN MOR 73): the squished skull roof was something like 28 cm wide, the minimum width across both premaxillae is about 7 cm, the maximum about 12 cm, corresponding to ratios of 0.25 to 0.43 – adding further margins of error, we have scored state 1, 2 or 3.

The skull roof width of **Archaeovenator* has not been reconstructed, but the ratio of premaxillary width to skull table width is 0.225 (Data S5; Reisz & Dilkes, 2003); it follows that the ratio of premaxillary width to skull roof width must have been 0.225 or lower, meaning states 3 or 4.

Appendix-Table 1: Ratios of premaxillary width to skull roof width, and changes to the scores of character 3 (PREMAX 7). Taxa underlain in blue were scored 0 by RC07, taxa underlain in yellow were scored 1, and the remainder were scored as unknown or have been added by us; the latter are marked with an asterisk. Abbreviation: *Ph.*, *Pholiderpeton*. See Data S5 and its legend for more information.

OTU	Ratio (rounded)	New score	Measured in:
Brachydectes	0.780	0	Pardo & Anderson, 2016: fig. 4B
Caerorhachis	0.689	0	Ruta, Milner & Coates, 2002
Crassigyrinus	0.620	0	Clack, 1998
Phlegethontia	0.569	0	Anderson, 2007a
Ventastega	0.568	0	Ahlberg et al., 2008
Cochleosaurus	0.556	0	Schoch & Milner, 2014: fig. 13D
**Spathicephalus*	0.544	0	Beaumont & Smithson, 1998: fig. 5
**Erpetosaurus*	0.541	0	Milner & Sequeira, 2011
Eryops	0.535	0	Schoch & Milner, 2014: fig. 3C
Dendrerpetidae	0.533	0	*Dendrysekos*: Schoch & Milner, 2014: fig. 17C
Karaurus	0.508	0	Ivachnenko, 1978
Edops	0.491	1	Schoch & Milner, 2014: fig. 13A
Chenoprosopus	0.484	1	Schoch & Milner, 2014: fig. 13E
Capetus	0.479	1	Sequeira & Milner, 1993
**Konzhukovia*	0.479	1	Gubin, 1991: drawing 6a
Albanerpetidae	0.477	1	*Celtedens*: McGowan, 2002
Orobates	0.469	1	Kissel, 2010: fig. 32B
Balanerpeton	0.462	1	Schoch & Milner, 2014: fig. 17A
Amphibamus	0.462	1	Schoch & Milner, 2014: fig. 30
**Llistrofus*	0.456	1	Bolt & Rieppel, 2009
**Pholidogaster*	0.452	1	Panchen, 1975
Eucritta	0.446	1	Clack, 2001: fig. 8
Ptyonius	0.446	1	Bossy & Milner, 1998
**Sclerocephalus*	0.445	1	Schoch & Witzmann, 2009a
**Mordex*	0.445	1	Schoch & Milner, 2014: fig. 32A
**Micropholis*	0.443	1	Schoch & Rubidge, 2005: fig. 3B
Vieraella	0.440	1	Báez & Basso, 1996: fig. 6, 7
Eoscopus	0.432	1	Daly, 1994: fig. 3
Doleserpeton	0.430	1	Sigurdsen & Bolt, 2010
Trimerorhachis	0.427	1	Schoch & Milner, 2014: fig. 20A
**Acanthostomatops*	0.426	1	Witzmann & Schoch, 2006a
**Nigerpeton*	0.419	1	Steyer et al., 2006
Apateon	0.417	1	Schoch & Fröbisch, 2006: fig. 1D
**Iberospondylus*	0.411	1	Laurin & Soler-Gijón, 2006: fig. 1A; left side approximately doubled
Platyrhinops	0.408	1	Clack & Milner, 2010: fig. 9
Panderichthys	0.407	1	Vorobyeva & Schultze, 1991
Greererpeton	0.402	1	Smithson, 1982
Lethiscus	0.402	1	J. Pardo, pers. comm. 2017
Ichthyostega	0.404	1	Clack & Milner, 2015: fig. 8
**Palatinerpeton*	0.399	2	Boy, 1996: fig. 3

Saxonerpeton	0.395	2	CG78: fig. 22
**Glanochthon*	0.392	2	Schoch & Witzmann, 2009b: fig. 2A approximately doubled
Eocaecilia	0.386	2	Jenkins, Walsh & Carroll, 2007
Diadectes	0.385	2	Kissel, 2010: fig. 36B
Valdotriton	0.377	2	Evans & Milner, 1996
Phonerpeton	0.375	2	Dilkes, 1990: fig. 1
**Deltaherpeton*	0.370	2	Bolt & Lombard, 2010: fig. 2; left premaxilla doubled, otherwise right side approximately doubled
**Gerobatrachus*	0.370	2	estimated from Anderson et al., 2008a: fig. 2b
Acanthostega	0.368	2	Porro, Rayfield & Clack, 2015
**Cheliderpeton*	0.366	2	Werneburg & Steyer, 2002
Westlothiana	0.365	2	Smithson et al., 1994
Isodectes	0.365	2	Schoch & Milner, 2014: fig. 20E
Megalocephalus	0.362	2	Beaumont, 1977: fig. 8
**Liaobatrachus*	0.360	2	Dong et al., 2013
Gephyrostegus	0.360	2	Klembara et al., 2014
Asaphestera	0.358	2	CG78: fig. 7
**Carrolla*	0.355	2	Maddin, Olori & Anderson, 2011
Acheloma	0.348	2	Polley & Reisz, 2011
**Pangerpeton*	0.346	2	estimated from Wang & Evans, 2006
Ecolsonia	0.344	2	Berman, Reisz & Eberth, 1985: fig. 5A
Micromelerpeton	0.341	2	Boy, 1995: fig. 8A
Solenodonsaurus	0.339	2	Danto, Witzmann & Müller, 2012; maximum possible width of premaxilla estimated
**Saharastega*	0.339	2	Damiani et al., 2006
**Chroniosaurus*	0.335	2	Clack & Klembara, 2009
Silvanerpeton	0.333	2	Ruta & Clack, 2006
Rhynchonkos	0.330	2	CG78: fig. 63
Microphon	0.328	2	Bulanov, 2003: fig. 22
Broiliellus	0.327	2	Carroll, 1964
Eoherpeton	0.326	2	Panchen, 1975
Schoenfelderpeton	0.325	2	Boy, 1986: fig. 13
Colosteus	0.322	2	Hook, 1983
Anthracosaurus	0.321	2	Clack, 1987a
**Lydekkerina*	0.320	2	Hewison, 2007: fig. 30
Seymouria	0.317	2	Laurin, 1996a
Keraterpeton	0.314	2	Bossy & Milner, 1998
Paleothyris	0.313	2	Carroll, 1969b: fig. 4B
Micraroter	0.313	2	CG78: fig. 56
Ossinodus	0.309	2	Warren, 2007
**Palaeoherpeton*	0.306	2	Panchen, 1964
Sauropleura	0.306	2	Bossy & Milner, 1998
Oestocephalus	0.306	2	Anderson, 2003b
Pelodosotis	0.304	2	CG78: fig. 48
Euryodus	0.304	2	CG78: fig. 37
**Tungussogyrinus*	0.304	2	Werneburg, 2009
Ph. attheyi	0.299	3	Panchen, 1972

Discosauriscus	0.299	3	Klembara et al., 2006
Leptorophus	0.298	3	Boy, 1986: fig. 4
Pederpes	0.298	3	Clack & Finney, 2005
Limnoscelis	0.298	3	Kissel, 2010: fig. 13A[1]
Eusthenopteron	0.293	3	Clack, 2007
Microbrachis	0.292	3	Vallin & Laurin, 2004
Batropetes	0.291	3	Glienke, 2013: fig. 2
*Caseasauria	0.290	3	*Eothyris*: Reisz, Godfrey & Scott, 2009
**Archegosaurus*	0.289	3	Witzmann, 2006: fig. 5
Scincosaurus	0.289	3	Milner & Ruta, 2009
Proterogyrinus	0.277	3	Holmes, 1984
**Utaherpeton*	0.272	3	Carroll, Bybee & Tidwell, 1991
Stegotretus	0.271	3	Berman, Eberth & Brinkman, 1988: fig. 10B
Hyloplesion	0.271	3	CG78: fig. 89B
Ph. scutigerum	0.271	3	Clack, 1987b
Ariekanerpeton	0.271	3	Klembara & Ruta, 2005a
**Crinodon*	0.268	3	CG78: fig. 11
Captorhinus	0.266	3	Fox & Bowman, 1966: fig. 3
Urocordylus	0.262	3	Bossy & Milner, 1998
Neldasaurus	0.254	3	Schoch & Milner, 2014: fig. 20C
Notobatrachus	0.252	3	Báez & Nicoli, 2004
Diceratosaurus	0.250	3	Bossy & Milner, 1998
Utegenia	0.250	3	Klembara & Ruta, 2004a
Bruktererpeton	0.246	3	estimated based on Boy & Bandel, 1973: fig. 7
Tseajaia	0.245	3	Berman, Sumida & Lombard, 1992: fig. 11
Tuditanus	0.240	3	Carroll & Baird, 1968: fig. 9
**Karpinskiosaurus*	0.240	3	Klembara, 2011
Petrolacosaurus	0.238	3	Berman, Sumida & Lombard, 1992: fig. 11
Cardiocephalus	0.235	3	CG78: fig. 69
**Pseudophlegethontia*	0.233	3	Anderson, 2003b: fig. 3
**Australerpeton*	0.228	3	Eltink et al., 2016: fig. 5
Archeria	0.227	3	Holmes, 1989
**Chelotriton*	0.226	3	Marjanović & Witzmann, 2015: fig. 7
**Neopteroplax*	0.222	3	Romer, 1963: fig. 3
Kotlassia	0.216	3	Bulanov, 2003: fig. 30
**Bystrowiella*	0.203	3	Witzmann & Schoch, 2017: fig. 15C
**Sparodus*	0.193	4	Carroll, 1988
**Platyoposaurus*	0.193	4	Gubin, 1991: drawing 3a
Odonterpeton	0.188	4	CG78: fig. 99B
Batrachiderpeton	0.153	4	Bossy & Milner, 1998
Pantylus	0.149	4	Romer, 1969: fig. 1
Diplocaulus	0.091	4	Olson, 1951: pl. 5B left side doubled
Diploceraspis	0.077	4	Beerbower, 1963: fig. 2

[1] A line drawing identical to Berman, Reisz & Scott (2010: fig. 3A), but slightly more convenient to measure.

4. PREMAX 8: **Anteriormost surface of premaxilla oriented obliquely, so that mouth opens subterminally: absent (0); present (1).**

Ventastega (Ahlberg, Lukševičs & Lebedev, 1994) and *Lethiscus* (Anderson, Carroll & Rowe, 2003; Pardo et al., 2017) have state 0.

Panderichthys is famous for having a subterminal mouth (state 1; e.g. Brazeau & Ahlberg, 2006). This condition has also been reconstructed for *Ichthyostega* (Clack & Milner, 2015: fig. 8).

Unknown in *Edops* (Romer & Witter, 1942; D. M., pers. obs. of MCZ 1378).

Although we have kept the score of 1 for *Batropetes*, this seems to be a very weak case (Glienke, 2013, 2015).

Conversely, while the premaxillae of **Quasicaecilia* are unknown, the nasals them-selves are recurved so that the nostrils opened on the ventral side of the snout (Pardo, Szostakiwskyj & Anderson, 2015); the mouth was inevitably subterminal, so we have scored state 1.

5. PREMAX 9: **Maxilla in ventral view more or less limited to toothrow (0); contributes to the palate labial to the choana for at least twice the width of the toothrow (1).** The original wording was: "Shelf-like contact between premaxilla and maxilla occurring mesial to marginal tooth row on palate and extending medially for at least twice the width of such a row: absent (0); present (1)", but it took us a long time to understand this wording. – From here on, RC07 consistently wrote "mesial" (toward the jaw symphysis, along the curvature of the jaw) when they were clearly aiming at "medial" (toward the sagittal plane) and actually meant "lingual" (toward the tongue, at 90° to the curvature of the jaw – caudal at the symphysis, medial around the jaw joints).

Ventastega (Ahlberg, Lukševičs & Lebedev, 1994), most likely *Amphibamus* (Schoch & Milner, 2014: fig. 30B; implicitly Daly, 1994), Albanerpetidae (McGowan, 2002, Venczel & Gardner, 2005) and *Lethiscus* (Anderson, Carroll & Rowe, 2003) have state 0. We also continue to consider the tiny teeth of frogs, which create a wide palatal contribution not only labial to the choana, but along the entire length of the maxilla, as state 0, and have therefore kept the scores of *Notobatrachus* and *Vieraella*.

Orobates has state 0 (Nyakatura et al., 2015: digital reconstruction).

In *Ossinodus* (Warren, 2007), the contribution of the maxilla to the palate lateral of the choana is easily twice as wide as the toothrow (not counting the very large caniniforms) at the caudal end of the choana (at the mediolateral suture to the palatine), but only about once as wide at the rostral end (at the suture to the premaxilla). We count this as state 1.

We have scored state 0 for **Perittodus* and **Aytonerpeton* after Clack et al. (2016: matrix).

6. TEC 1: **Anterior tectal: present (0); absent (1).** We follow Panchen (1967), Beaumont (1977), Clack (1998) and RC07 in considering the septomaxilla homologous to the lateral rostral rather than the anterior tectal, because the septomaxilla lies caudal and/or ventral to the naris, like the lateral rostral and unlike the dorsally positioned anterior tectal (contra Sigurdsen & Green, 2011: supp. inf.), though we would like to point out that this question has received disquietingly little attention in the literature. Clack et al. (2012a) presented a phylogenetic, arguably scenario-based argument for the reduction and complete loss of the lateral rostral and for the homology of anterior tectal and septomaxilla. We fear that this question cannot be decided without new material, because at the moment the seeming disappearance of the anterior tectal, the seeming disappearance of the lateral rostral, and the seeming appearance of the septomaxilla are optimized as happening in indistinguishable places in the tree – we need more fossil noses from the Late Devonian and the Mississippian.

A good candidate for possessing both an anterior tectal and a septomaxilla is **Aytonerpeton*, but this is currently unclear and requires further study, if not further specimens (see below).

State 0 is almost certainly present in *Ventastega* (Ahlberg, Lukševičs & Lebedev, 1994).

The condition is unknown in *Colosteus* and *Greererpeton* (Bolt & Lombard, 2010), in *Whatcheeria* (Lombard & Bolt, 1995) and in *Batropetes* (Glienke, 2013, 2015).

Phonerpeton was scored as unknown in RC07. Although many sutures are difficult or impossible to find in AMNH 7150 or MCZ 2313, there is no evidence for an anterior tectal in either of them; in particular, the margins of both nares are undamaged in both skulls (D. M., pers. obs.). We have therefore scored state 1 for *Phonerpeton*.

State 1 is likewise present in *Lethiscus* (Pardo et al., 2017).

Due to the argument above, we have scored the mystery bone of **Ymeria* as the septomaxilla, so that the presence of the anterior tectal is unknown.

In **Pholidogaster*, Panchen (1975) reconstructed state 1 with confidence. However, of the two specimens, the type (which exposes the dorsal surface) has undergone compression and shearing (Panchen, 1975: 614; Bolt & Lombard, 2010), including but not limited to disruption by the tusks on the dentary and the palatine (Panchen, 1975: fig. 11; Bolt & Lombard, 2010) which could have obscured the distinction between a break and a suture between the nasal, the anterior tectal and/or the prefrontal (the nasal-prefrontal suture is shown in fig. 11 as having an unusual shape and running in part in a lateral-line groove on the strongly ornamented snout). The referred specimen is only exposed in ventral view and heavily damaged medial to the septomaxilla (Panchen, 1975: fig. 13). Given that the only illustrations (other than fig. 12, a not very large photograph of the type skull) are thick-lined line drawings, restudy of both specimens will be necessary – and perhaps insufficient – to resolve this and related issues. In short, we have joined the skepticism of Bolt & Lombard (2010) and scored this character as unknown.

Panchen (1964) thought that anterior tectals were present in **Palaeoherpeton*; we follow his later assessment (Panchen, 1972: 287) that these areas were in fact part of the lacrimals and have therefore scored state 1.

We accept the inference of Ahlberg (1995) that a slot on the premaxilla of **Elginerpeton* is the sutural surface for the anterior tectal, and have therefore scored state 0.

The septomaxilla identified in **Aytonerpeton* by Clack et al. (2016) is clearly an anterior tectal; we have scored state 0.

7. SPTMAX 1-2: **Septomaxilla with exposure on skull surface (0), wholly inside nostril (1), absent (2) (unordered).** RC07 had deleted the character SPTMX 1 of Ruta, Coates & Quicke (2003), which concerned the presence of the septomaxilla; SPTMX 2 was "Septomaxilla a detached ossification inside nostril: no (0); yes (1)". We have assigned state 2 only to taxa of which many articulated skulls are known; otherwise we have interpreted absence as possible post-mortem loss or incomplete preparation (as cautioned by RC07) and scored it as partial uncertainty (state 1 or 2). Nonetheless, state 2 is present in *Acanthostega* (Ahlberg, Lukševičs & Lebedev, 1994; Clack, 1994a, 2002, 2003b; Clack et al., 2012a; by implication Porro, Rayfield & Clack, 2015), *Microbrachis* (no evidence of a septomaxilla in 100 inspected specimens: Olori, 2015), apparently *Lethiscus* (Pardo et al., 2017) and *Phlegethontia* (Anderson, 2002, 2007a) as well as in *Notobatrachus*, from which no septomaxilla has been reported despite the enormous number of known and superbly preserved individuals (Báez & Basso, 1996; Báez & Nicoli, 2004, 2008); *Acanthostega* was scored SPTMAX 2(0) by RC07, the others as unknown. – We have also assigned state 2 to the added OTU **Sclerocephalus* (Schoch & Witzmann, 2009a).

Panderichthys (Vorobyeva & Schultze, 1991), *Baphetes* (judging from the presence of dermal ornament on the septomaxilla: Milner, Milner & Walsh, 2009), *Trimerorhachis* (Milner & Schoch, 2013), *Eocaecilia* (Jenkins, Walsh & Carroll, 2007), *Gephyrostegus* (apparently: Klembara, 2014: fig. 5B) and *Seymouria* (Laurin, 1996a; Klembara et al., 2005) show state 0. While the septomaxilla is not preserved in *Batropetes*, Glienke (2013: 79) confidently reconstructed state 0 for this taxon as well, "owing to the premaxilla and the naris" (and presumably the prefrontal).

We have tentatively kept state 0 for *Ichthyostega* under the assumption that the lateral rostral identified by Jarvik (1996) was correctly identified as such and is homologous to the septomaxilla (see above); but we caution that it has not been found again (Clack & Milner, 2015).

Romer & Witter (1942) stated very explicitly that *Edops* has state 0. However, assuming that both they and D. M. (pers. obs.) have traced the sutures in MCZ 1378 correctly, the sculpture on the septomaxilla is much lower and finer than that on the rest of the skull roof, and the surface lies on a more ventral level, bounded by a sharp vertical step formed by the maxilla and the lacrimal (the margin of the nasal, which most likely participated, is not preserved). This constitutes state 1. – The same condition occurs in **Pholidogaster* (Panchen, 1975: 617).

Acheloma shows state 1 (Dilkes & Reisz, 1987). *Phonerpeton*, on the other hand, has state 0 (D. M., pers. obs. of the type specimen, MCZ 1419).

State 1 is present in *Doleserpeton* (Sigurdsen & Bolt, 2010) as well as *Hapsidopareion*, *Micraroter* and *Rhynchonkos* (CG78).

Following Clack & Milner (2010), we assign state 1 or 2 to *Eoscopus* and *Platyrhinops*.

While *Colosteus* and *Greererpeton* have been reconstructed as possessing state 2 (Smithson, 1982; Hook, 1983), they should rather be scored as unknown (Bolt & Lombard, 2010).

**Acanthostomatops* is apparently polymorphic, showing states 0 and 1 (illustrations in Witzmann & Schoch, 2006a). It is possible that this is ontogenetic, as state 0 is seemingly only found in the largest specimens, but the sample size is probably not large enough to tell.

We have tentatively scored state 0 for **Ymeria* as explained for TEC 1.

The septomaxilla identified in **Aytonerpeton* by Clack et al. (2016) is clearly an anterior tectal (see TEC 1 immediately above). Whether a septomaxilla is or was present is unclear: the caudoventral rim of the naris is damaged, and a fragment bounded by the naris, the anterior tectal, the lacrimal and the maxilla may or may not be the septomaxilla, as may be the continuation of the maxilla in the naris (no sutures are visible anywhere in the supplementary video).

8. NAS 1: **Paired nasals: absent (0); present (1).** We interpret this character as referring to identifiable nasals as separate bones; *Eusthenopteron* and *Panderichthys* have a "postrostral mosaic" (now state 84(0)) which contains several candidates for nasal homologues, so we have scored them as unknown, unlike *Diplocaulus* and *Diploceraspis*, in which nasals are definitely absent according to published descriptions.

Ventastega has state 1 (Lukševičs, Ahlberg & Clack, 2003, Ahlberg et al., 2008). Bulanov (2003: fig. 30) reconstructed state 1 for *Kotlassia*; judging from the text (p. S53), this appears to be correct.

Although there is evidence of nasals in **Palatinerpeton*, the sagittal suture of the dorsal side of the skull is entirely unknown (Boy, 1996), so we cannot tell if the presumed nasals were fused and have scored **Palatinerpeton* as unknown.

9. NAS 2: **Nasals more (0) or less than (1) one-third as long as frontals.**

State 0 is present in *Panderichthys* (whichever bones actually are the nasals: Vorobyeva & Schultze, 1991), *Ventastega* (Ahlberg et al., 2008), *Microbrachis* (Vallin & Laurin, 2004), *Hyloplesion* (CG78), *Lethiscus* (Pardo et al., 2017), *Oestocephalus* (Carroll, 1998a; Anderson, 2003a) and *Phlegethontia* (Anderson, 2007a). Bulanov (2003: fig. 30) reconstructed state 0 for *Kotlassia*; judging from the text (p. S53), this appears to be correct.

State 0 is furthermore plesiomorphic for Albanerpetidae, as far as can be reconstructed from the fact that it occurs in both species of *Celtedens* (McGowan, 2002) as well as in *Albanerpeton pannonicum* (Venczel & Gardner, 2005). Only *A. inexpectatum* shows state 1 (Estes & Hoffstetter, 1976), if that reconstruction is even correct (Venczel & Gardner, 2005); 1 is the state RC07 ascribed to Albanerpetidae as a whole. Unfortunately, no other albanerpetids preserve nasals.

The state of this character is unknown in *Adelospondylus*, *Adelogyrinus* and *Dolichopareias* (Andrews & Carroll, 1991) and *Leptoropha* (Bulanov, 2003).

10. NAS 5: **Narial margins of nasals parallel to each other and to the sagittal plane (0), at an angle (so that, if extended as straight lines, they would meet rostral to the snout) (1).** The original wording, "Nasals broad plates delimiting most of the posterodorsal and me[d]ial margins of nostrils and with lateral margins diverging abruptly in their anterior portions: absent (0), present (1)", describes a combination of three characters: the length/width ratio of the nasals (possibly correlated to the length-width ratio of the snout and thus probably to the nasal/frontal length ratio, NAS 2, as well as the nasal/parietal length ratio, NAS 6), the relative position of nasals and external nares (plausibly correlated to the position and shape of the nasal-premaxilla suture, PREMAX 1-2-3), and the character described here.

This character is inapplicable when the nasal does not participate in the narial margin. This is the case in *Acanthostega*, *Ichthyostega* and **Aytonerpeton*, where the anterior tectal intervenes, in *Proterogyrinus* (Holmes, 1984), which has a premaxilla-lacrimal suture instead, and possibly in *Colosteus*, *Greererpeton* and **Pholidogaster* (Smithson, 1982; Hook, 1983; Bolt & Lombard, 2010; see above under TEC 1 – ch. 6), in which the narial region is poorly preserved and the premaxilla and the prefrontal or the anterior tectal may meet instead of the nasal and the naris. The state of this character is furthermore unknown in *Westlothiana* (Smithson et al., 1994), *Tuditanus* (CG78) and *Lethiscus* (Wellstead, 1982; Anderson, Carroll & Rowe, 2003).

State 0 is found contrary to RC07 in *Phonerpeton* (D. M., pers. obs. of AMNH 7150 and MCZ 2313), and *Ptyonius* (Bossy & Milner, 1998). We also assign state 0 to *Phlegethontia* (Anderson, 2007a), where the margins form an extremely small angle, to **Nigerpeton*, where in the best-preserved snout (Sidor, 2013) the left nasal only contributes to the rostral margin of the naris, while the right naris forms a symmetric mediolateral notch in the right nasal.

State 1 is almost ubiquitous, being found in *Crassigyrinus* (Clack, 1998), *Whatcheeria* (Bolt & Lombard, 2000), *Baphetes* (adult) and *Megalocephalus* (Beaumont, 1977), *Eucritta* (Clack, 2001), *Chenoprosopus* (Langston, 1953), *Isodectes* (Sequeira, 1998), *Trimerorhachis* (Milner & Schoch, 2013), Dendrerpetidae (Holmes, Carroll & Reisz, 1998), *Eryops* (Sawin, 1941), *Broiliellus* (Carroll, 1964; Schoch, 2012), *Eoscopus* (Daly, 1994), *Micromelerpeton* (though the least paedomorphic morphotype is somewhat borderline: Boy, 1995), Albanerpetidae (Venczel & Gardner, 2005), *Eocaecilia* (Jenkins, Walsh & Carroll, 2007), *Caerorhachis* (Ruta, Milner & Coates, 2002), *Eoherpeton* (though this is not entirely clear: Panchen, 1975; Smithson, 1985), *Archeria* (Holmes, 1989), *Pholiderpeton attheyi* (Panchen, 1972), *Anthracosaurus* (Clack, 1987a), *Pholiderpeton scutigerum* (Clack, 1987b), *Bruktererpeton* (Boy & Bandel, 1973: pl. 8), *Gephyrostegus* (Carroll, 1970; Klembara et al., 2014), *Soleno-*

donsaurus (Danto, Witzmann & Müller, 2012: fig. 8A), *Kotlassia* (Bulanov, 2003: S53, S54), *Discosauriscus*, *Ariekanerpeton*, *Microphon* and *Utegenia* (Bulanov, 2003, 2014; Klembara & Ruta, 2004a, 2005a), *Diadectes* (Berman, Sumida & Lombard, 1992; Berman, Sumida & Martens, 1998), *Limnoscelis* (Fracasso, 1983; Berman, Reisz & Scott, 2010), *Captorhinus* (Fox & Bowman, 1966; Heaton, 1979), *Paleothyris* (perhaps a bit borderline; Carroll, 1969b), *Petrolacosaurus* (Reisz, 1981), *Batropetes* (Carroll, 1991; Glienke, 2013), *Pantylus*, *Asaphestera*, *Saxonerpeton*, *Hapsidopareion*, *Micraroter*, *Pelodosotis*, *Rhynchonkos*, *Cardiocephalus*, *Euryodus*, *Microbrachis*, *Hyloplesion* and *Odonterpeton* (CG78; Vallin & Laurin, 2004; Szostakiwskyj, Pardo & Anderson, 2015), *Stegotretus* (Berman, Eberth & Brinkman, 1988), *Brachydectes* (Wellstead, 1991; Pardo & Anderson, 2016), *Acherontiscus* (Carroll, 1969a), *Adelogyrinus* and *Dolichopareias* (Andrews & Carroll, 1991), *Batrachiderpeton* (Bossy & Milner, 1998), *Diceratosaurus* (Jaekel, 1903; A. C. Milner, 1980; Bossy & Milner, 1998), *Oestocephalus* (Carroll, 1998a), *Capetus* (Sequeira & Milner, 1993), apparently *Orobates* (Berman et al., 2004), *Pederpes* (Clack & Finney, 2005), *Silvanerpeton* (Ruta & Clack, 2006), and *Tseajaia* (Moss, 1972; Berman, Sumida & Lombard, 1992).

11. NAS 6: **Parietal/nasal length ratio less than (0) or greater than 1.45 (1).**

State 1 is known to occur in *Panderichthys* (no matter which of the candidates are in fact the nasals: Clack, 2007), *Trimerorhachis* (Milner & Schoch, 2013), *Amphibamus* (Milner, 1982; Schoch & Milner, 2014), *Eocaecilia* (Jenkins, Walsh & Carroll, 2007), *Karaurus* (Ivachnenko, 1978), *Batropetes* (Glienke, 2013), *Asaphestera*, *Saxonerpeton*, *Cardiocephalus* and *Euryodus* (CG78), and *Brachydectes* (Wellstead, 1991; Pardo & Anderson, 2016). Bulanov (2003: fig. 30) further reconstructed state 1 for *Kotlassia*; judging from the text (p. S53), this appears to be correct.

Westlothiana is polymorphic: it shows state 1 on the left and (as scored by RC07) state 0 on the right side of the type specimen (Smithson et al., 1994). *Discosauriscus pulcherrimus* has state 0 as scored by RC07 (Klembara, 1997: fig. 33), while *D. austriacus* has state 1 (Klembara, 1997: fig. 27; Klembara et al., 2006: fig. 4C); we have accordingly scored polymorphism for *Discosauriscus*.

Unknown in *Stegotretus* (Berman, Eberth & Brinkman, 1988), *Acherontiscus* (Carroll, 1969a), *Adelospondylus*, *Adelogyrinus* and *Dolichopareias* (Andrews & Carroll, 1991); inapplicable to *Phlegethontia*, where the parietals are absent (Anderson, 2002, 2007a).

Unknown and likely borderline in **Pholidogaster* (Panchen, 1975).

12. PREFRO 1: **Separately ossified prefrontal: present (0); absent (1).**

State 0 is present in *Ventastega* (Ahlberg, Lukševičs & Lebedev, 1994); we follow Ascarrunz et al. (2016) in also scoring it for *Triadobatrachus*.

In **Palaeoherpeton* the sutures between the prefrontal, lacrimal and jugal have not been traced (Panchen, 1964); given the sutures to the postfrontal and the quadratojugal, we presume that if any of these bones was absent, that was the lacrimal (LAC 1(?)), so we have scored state 0 of the present character and also of PREFRO 7 while leaving the other PREFRO characters as unknown.

Similarly, the region where the suture between prefrontal and lacrimal would be expected is not preserved in **Neopteroplax* (Romer, 1963: fig. 1); we have again concluded that the presence of the lacrimal is unknown. Even so, however, **Neopteroplax* has PREFRO 2(0).

We have scored **Aytonerpeton* as unknown because the supplementary video of Clack et al. (2016) hints that the supposed nasal may be composed of the nasal and the prefrontal.

13. PREFRO 2: **Prefrontal less than (0) or more than (1) three times longer than wide […] in dorsal aspect.**

State 0 is present in *Ventastega* (Ahlberg et al., 2008).

Colosteus has state 0 regardless of how the prefrontal is reconstructed (Hook, 1983; Bolt & Lombard, 2010). *Greererpeton*, however, does just reach state 1 even if a large anterior tectal is assumed, so that it was scored correctly in RC07 in any case (Bolt & Lombard, 2010).

If the prefrontal is correctly identified as such, *Triadobatrachus* must share state 0 (Ascarrunz et al., 2016: fig. 12).

State 1 is found in *Acanthostega* (Porro, Rayfield & Clack, 2015), *Eocaecilia* (Jenkins, Walsh & Carroll, 2007), *Gephyrostegus* (Klembara et al., 2014) and *Solenodonsaurus* (Danto, Witzmann & Müller, 2012) as well as in Glienke's (2013, possibly also 2015) reconstructions of *Batropetes*.

The adult specimen of *Baphetes orientalis* has state 0 on the left but state 1 on the right side (Beaumont, 1977: fig. 25), making *Baphetes* polymorphic.

Albanerpeton pannonicum, the only albanerpetid that can be scored with confidence, just barely reaches state 1 (Venczel & Gardner, 2005), so we ascribe this state to Albanerpetidae as a whole.

Unknown in *Lethiscus* due to insufficient preservation (Pardo et al., 2017).

**Beiyanerpeton* is always close to the cutoff point, but at least one specimen has state 0 on the left and state 1 on the right side (Gao & Shubin, 2012: fig. 2); we have scored it as polymorphic.

**Australerpeton* is polymorphic, sometimes within an individual (Eltink et al., 2016: fig. 2–5).

We have assigned state 1 to **Quasicaecilia* by measuring along the curve formed by the orbit (Pardo, Szostakiwskyj & Anderson, 2015: fig. 3A). In strict rostrocaudal terms, the prefrontal is about as long as it could be without extending dorsal to the nostril, and it is not as short as it could be.

The stippled lines in Anderson (2003a: fig. 3A) are justified by Pardo et al. (2017: ext. data fig. 4, video) to the extent of making state 1 very probable for **Coloraderpeton*.

14. PREFRO 3: **Antorbital portion of prefrontal forming near-equilateral triangular lamina: absent (0); present (1).** State 0, which unites a wide range of different states, may have to be split to reveal more phylogenetic signal.

State 0 is present in *Ventastega* (Ahlberg, Lukševičs & Lebedev, 1994) and *Solenodonsaurus* (Danto, Witzmann & Müller, 2012) as well as in the most mature specimens of *Micromelerpeton* (Schoch, 2009b: fig. 2b).

State 1 is documented in *Ichthyostega* (a larger version perhaps: Clack & Milner, 2015: fig. 8), *Balanerpeton* (arguably: Milner & Sequeira, 1994), *Phonerpeton* (Dilkes, 1990; D. M., pers. obs. of AMNH 7150 and MCZ 2313), *Kotlassia* (Bulanov, 2003: fig. 30), *Discosauriscus* (both species, though sometimes borderline: Klembara, 1997), and *Ariekanerpeton* (Klembara & Ruta, 2005a), and makes a surprise appearance in *Lethiscus* (Pardo et al., 2017: extended data fig. 1a, b, 3b).

Baphetes is best scored as unknown because of its antorbital fenestrae. Even so, however, the shape of the rostral end of the prefrontal only allows state 0 in *Megalocephalus* (Beaumont, 1977) and **Spathicephalus* (Smithson et al., 2017: fig. 3C).

Eucritta is polymorphic, sometimes within the same individual (Clack, 2001: fig. 6).

The condition is unknown in *Valdotriton* (Evans & Milner, 1996), *Westlothiana* (Smithson et al., 1994) and *Tseajaia* (Moss, 1972; Berman, Sumida & Lombard, 1992).

**Acanthostomatops* is polymorphic, sometimes within the same individual (Witzmann & Schoch, 2006a). This may be at least partly ontogenetic, in that state 0 is only found in large individuals, but these same individuals are sometimes polymorphic.

deleted PREFRO 6: **Prefrontal/premaxilla suture: absent (0); present (1).**

State 0 is present in *Ventastega* (Ahlberg et al., 2008).

Unknown in *Colosteus* and *Greererpeton* (Bolt & Lombard, 2010; see above under TEC 1 – ch. 6) as well as *Adelospondylus* (Andrews & Carroll, 1991). We have also scored *Diplocaulus* as unknown, because it lacks nasals (see NAS 1 above) that would separate the prefrontals from the premaxillae – just like *Diploceraspis*, which was already scored as unknown.

State 1 is possibly present in *Albanerpeton inexpectatum*, but absent in *A. pannonicum* and in *Celtedens* (Venczel & Gardner, 2005). According to Gardner, Evans & Sigogneau-Russell (2003), *Albanerpeton* and *Celtedens* are sister-groups; the condition in the sister-group to the clade formed by both, *Anoualerpeton*, is unknown. We consequently infer that state 0 is the plesiomorphy for Albanerpetidae and have scored Albanerpetidae as possessing state 0.

This reduces the distribution of state 1 to *Acherontiscus*, which is very fragmentarily preserved (Carroll, 1969a); even accepting that *Acherontiscus* has state 1, the character is parsimony-uninformative in the original taxon sample, so we have deleted it. This also relieves us from worrying about correlation with PREFRO 8 (see below) – PREFRO 6(1) is probably impossible without PREFRO 8(2) (which is unknown but likely in *Acherontiscus*).

State 1 is unambiguously present in **Utaherpeton* (Carroll, Bybee & Tidwell, 1991), but, given the uncertainty in *Acherontiscus*, we have not reintroduced this character for the analysis with added taxa, either as a character or as an additional state of PREFRO 8 (which would then need a stepmatrix).

15. PREFRO 7: **Prefrontal without (0) or with (1) stout, lateral outgrowth.**

State 0 is present in *Ventastega* (Ahlberg, Lukševičs & Lebedev, 1994), *Triadobatrachus* (Ascarrunz et al., 2016: fig. 4, 12) and *Diploceraspis* (Beerbower, 1963).

State 1 is not limited to baphetids – *Karaurus* possesses a very clear case of it (Ivachnenko, 1978; D. M. and M. L., pers. obs. of unnumbered MNHN cast of PIN 2585/2), and *Acanthostega* has a small version (Porro, Rayfield & Clack, 2015).

We have scored **Spathicephalus* as polymorphic, although it is conceivable that the observed distribution (*S. mirus*: 0; *S. marsdeni*: 1) is ontogenetic because the only known specimen of *S. marsdeni* is half the size of that of *S. mirus* (Smithson et al., 2017).

16. PREFRO 8: **Caudal margin of external naris, including anterior tectal and septomaxilla, formed by lacrimal/nasal contact (0) or maxilla/nasal contact (excluding the lacrimal from the margin) (1); prefrontal entering nostril margin (2) (unordered).** The original wording was: "Prefrontal entering nostril margin: no (0); yes (1)." Now that we have split state 0 to make explicit which conditions it represents, this character makes use of the phylogenetic signal in the difference between the new states 0 and 1. We count the anterior tectal and the septomaxilla as part of the naris even if the latter lies entirely on the skull surface (as the former always does); this way, correlation with TEC 1 and SPTMAX 2 can be avoided.

State 0 is present in *Ventastega* (Ahlberg et al., 2008) and *Micraroter* (CG78).

State 1 is rare outside of stereospondylomorphs (**Sclerocephalus*, **Cheliderpeton*, **Archegosaurus*, **Konzhukovia*, **Lydekkerina* [polymorphic, see below], **Glanochthon*, **Platyoposaurus*, **Australerpeton*), but occurs in other particularly long-snouted animals,

namely *Megalocephalus* (Beaumont, 1977), *Chenoprosopus* (Hook, 1993; Reisz, Berman & Henrici, 2005), *Neldasaurus* (Chase, 1965; Boy, 1993; Milner & Schoch, 2013), *Pholiderpeton attheyi* (Panchen, 1972), **Nigerpeton* (Steyer et al., 2006) and probably **Saharastega* (unless the lacrimal had a quite unusual shape: Damiani et al., 2006; D. M., pers. obs. of MNN MOR 73). However, state 1 is also found in the distinctly short-snouted *Microphon gracilis* (even though *M. exiguus* and *M. arcanus* retain state 0, so that we have scored *Microphon* as polymorphic; Bulanov, 2003); outside the present taxon sample, it is shared by many short-snouted amniotes, and the snout of **Sclerocephalus* is not all that long either.

We have kept state 2 for *Acheloma*, *Phonerpeton* and *Ecolsonia* (and also scored it for **Mordex*); however, an indeterminate juvenile trematopid (Dilkes, 1993: fig. 4) shows state 0 instead. This implies that the naris only reached the prefrontal later during its allometric growth (NOS 3(2)), which in turn suggests that the OTUs with NOS 3(2) should be scored as unknown for the present character; in **Mordex*, however, state 2 is already seen in an aquatic larva (Werneburg, 2012a: fig. 14d).

Unknown in *Colosteus*; *Greererpeton* (like **Pholidogaster*) has state 0 or 2 (Bolt & Lombard, 2010; see above under TEC 1 – ch. 6). *Pholiderpeton scutigerum* has state 0 or 1.

In *Eocaecilia* (Jenkins, Walsh & Carroll, 2007), *Diploceraspis* (Beerbower, 1963), and *Phlegethontia* (Anderson, 2002), the lacrimal is absent, but the prefrontal is present. We have scored them as possessing the observed state 2 or the impossible state 0 – state 1 would be possible but is not observed. The presence of a lacrimal is unknown in *Valdotriton*, but we have scored it the same way because the prefrontal does appear to have reached the naris, and the maxilla would have needed an extremely tall process to reach the nasal (Evans & Milner, 1996: compare p. 632 to fig. 6b).

The lacrimal is likewise absent and the prefrontal present in **Chelotriton*; we have scored it as possessing the observed state 1 or the impossible state 0.

Glienke (2013) stated that *Batropetes fritschi* and *B. niederkirchensis* have state 2, and reconstructed both of them accordingly (if only in stippled lines for *B. fritschi*). Glienke (2015: ch. 5) scored all four species as having state 2 – but stated (p. 7) and illustrated (fig. 1D) that *B. palatinus* just barely has state 0, and made clear (p. 15) that neither the prefrontal nor the lacrimal of *B. appelensis* are well enough known to rule out any of the three states. Glienke (2015) found *B. appelensis* to be the sister-group of the other three species together, and *B. palatinus* as the closest relative of *B. fritschi*; consequently, the plesiomorphic state of *Batropetes* cannot be determined without an outgroup, and we have scored *Batropetes* as possessing both state 0 and state 2.

A rather clear case of state 2 occurs in *Lethiscus* (Pardo et al., 2017: especially extended data figure 3b).

**Lydekkerina* has states 0 and 1 (Jeannot, Damiani & Rubidge, 2006).

We have scored state 0 or 2 for **Aytonerpeton*.

Incidentally, as for a few other characters (see below), RC07: 94 claimed that this character "shows no clear phylogenetic pattern". Yet, the distribution of its states is far from chaotic. According to Analysis R4, state 2 is an autapomorphy of Holospondyli (reversed in *Lethiscus*, *Batropetes palatinus*, **Quasicaecilia* and the *Batrachiderpeton-Diplocaulus-Diploceraspis* clade, as well as Lissamphibia in those trees where it lies within Holospondyli); a synapomorphy of **Mordex*, *Acheloma*, *Phonerpeton*, and *Ecolsonia*, reversed in the clade of all other dissorophoids; and a synapomorphy of *Isodectes* and **Erpetosaurus* (reversed in *Trimerorhachis*). Clearly isolated occurrences of state 2 are limited to *Crassigyrinus* and *Karaurus*. In total, there are only 11–12 transitions to or from state 2 for 150 OTUs.

deleted PREFRO 9: **Prefrontal/maxilla suture rostral to lacrimal: absent (0); present (1).** The original wording did not distinguish a suture rostral to the lacrimal from a suture caudal to it; the latter condition is covered by LAC 2, see below.

State 0 is present in *Ventastega* (Ahlberg et al., 2008).

Unknown in *Colosteus* and *Greererpeton* (Bolt & Lombard, 2010) as well as *Adelospondylus* and *Dolichopareias* (Andrews & Carroll, 1991).

This character is inapplicable when the lacrimal is absent (LAC 1(1) below) or unknown; this is the case in *Eocaecilia* (Jenkins, Walsh & Carroll, 2007) and *Valdotriton* (Evans & Milner, 1996).

The redefinition further confers state 0 on *Karaurus* (Ivachnenko, 1978) and *Diplocaulus* (Bossy & Milner, 1998).

This leaves state 1 solely to, probably, *Adelogyrinus* (Andrews & Carroll, 1991). The character is therefore parsimony-uninformative; we have accordingly deleted it.

17. PREFRO 10: **Prefrontal contributes to more (0) or less than (1) half of orbit anterome[d]ial margin.** We interpret "anteromesial margin" as the rostromedial/rostrodorsal quarter of the orbit margin; state 1 means that less than half of this quarter is contributed by the prefrontal. This character has to be scored as unknown for all baphetoids except *Eucritta* because the antorbital fenestra occupies at least the other half of this quarter.

State 0 is present in *Ventastega* (Ahlberg, Lukševičs & Lebedev, 1994), *Ecolsonia* (Berman, Reisz & Eberth, 1985), *Amphibamus* (Milner, 1982: fig. 3a; Daly, 1994: fig. 18), *Platyrhinops* (Clack & Milner, 2010), *Schoenfelderpeton* (Boy, 1987), *Eocaecilia* (Jenkins, Walsh & Carroll, 2007), *Karaurus* (Ivachnenko, 1978) and *Urocordylus* (Bossy & Milner, 1998).

State 1 is found in *Ichthyostega* (Clack & Milner, 2015), *Greererpeton* (Smithson, 1982), *Proterogyrinus* (Holmes, 1984), *Westlothiana* (Smithson et al., 1994), *Micraroter* and *Hyloplesion* (CG78) and *Diploceraspis* (Beerbower, 1963). It also appears to be reached in **Spathicephalus* (Smithson et al., 2017: fig. 3C).

Apateon is polymorphic, sometimes within the same individual (Schoch & Milner, 2008; Fröbisch & Schoch, 2009b). So is *Diplocaulus* (Bossy & Milner, 1998).

Unknown or borderline in *Eoscopus* (appears to be exactly borderline: Daly, 1994: fig. 3), *Micromelerpeton* (Schoch, 2009b: fig. 2b), *Leptorophus* (Boy, 1987), *Valdotriton* (Evans & Milner, 1996), *Pholiderpeton scutigerum* (Clack, 1987b), *Bruktererpeton* (Boy & Bandel, 1973), *Lethiscus* (reconstructed as borderline: J. Pardo, pers. comm.; Pardo et al., 2017: ext. data fig. 3b, c, suggest state 0, but may not be in strict lateral view) and **Nigerpeton* (state 1 cannot be excluded: D. M., pers. obs. of MNN MOR 70).

18. LAC 1: **Separately ossified lacrimal: present (0); absent (1).**

Eocaecilia has state 1 (Jenkins, Walsh & Carroll, 2007), as does *Diploceraspis* (Beerbower, 1963).

The condition in *Valdotriton* (Evans & Milner, 1996) and *Westlothiana* (Smithson et al., 1994) is unknown.

19. LAC 2: **Contact between lacrimal and orbit (0); prefrontal contacts jugal or maxilla at its orbital margin (1).** We have added a mention of the maxilla to state 1 so that *Karaurus*, which lacks jugals, can be scored (as having state 1).

This character is inapplicable when the lacrimal is missing, e.g. in *Eocaecilia* (see LAC 1 above). It is further inapplicable when the orbit has a large rostroventral extension that separates the jugal from the prefrontal, in other words, in OTUs with state ORB 3/LAC 5(2) – the baphetoids. Similarly, we have scored *Isodectes* and *Micromelerpeton* as unknown, where

the lateral exposure of the palatine (state MAX 5/PAL 5(2)) is so long as to reach the prefrontal, excluding the lacrimal from the orbit margin entirely (Sequeira, 1998; Schoch, 2009b: fig. 2b).

State 0 is now known in *Ossinodus* (Warren, 2007) and appears to be preserved in *Kotlassia* (Bulanov, 2003: fig. 30).

The condition is unknown in *Proterogyrinus* (Holmes, 1984) as well as in *Adelospondylus*, *Adelogyrinus* and *Dolichopareias* (Andrews & Carroll, 1991).

Crassigyrinus has both states, sometimes within the same individual (Clack, 1998).

20. LAC 4: **Lacrimal without (0) or with (1) dorsome[d]ial digitiform process.** Because this process lies at the orbit margin, state 1 is impossible when the lacrimal does not participate in the orbit margin; we have accordingly scored this character as unknown in all taxa that have or may have state LAC 2(1), as well as in all baphetoids due to their antorbital emargination.

We have further scored *Tseajaia* as unknown (Moss, 1972; Berman, Sumida & Lombard, 1992; D. M., pers. obs. of CM 38033).

State 0 is now known in *Brachydectes* (Pardo & Anderson, 2016) and *Ossinodus* (Warren, 2007) and appears to be preserved in *Kotlassia* (Bulanov, 2003: fig. 30).

State 1 is documented in Albanerpetidae (Venczel & Gardner, 2005), *Pelodosotis* (CG78) and probably borderline in *Microbrachis* (Vallin & Laurin, 2004).

Colosteus (Hook, 1983) is polymorphic. So is *Trimerorhachis*, where *T. insignis* has state 1 but the other species all have state 0 (Milner & Schoch, 2013); this potentially contradicts the finding by Milner & Schoch (2013) that *T. insignis* lacks autapomorphies.

LAC 5 is merged with ORB 3, see below.

deleted LAC 6: **Portion of lacrimal lying anteroventral to orbit abbreviated: absent (0); present (1).** RC07 explained this character as follows: “In several lepospondyls (as well as in some temnospondyls), the antorbital part of the lacrimal is considerably foreshortened, regardless of relative snout proportions[,] and barely extends for one fourth of its total length anteroventral to the orbit.”

Despite this statement, and a similar one by Ruta, Coates & Quicke (2003: 307) which names several lepospondyls as having state 1, state 0 is instead present in all “lepospondyls”, including *Acherontiscus* and the adelogyrinids, according to all sources. Usually the entire lacrimal lies rostral to the orbit; even in small, large-eyed animals like *Doleserpeton* (Sigurd-sen & Bolt, 2010; correctly scored 0 in RC07), *Batropetes* (Carroll, 1991; Glienke, 2013, 2015), *Microbrachis* (Vallin & Laurin, 2004) and the urocordylids (Bossy, 1976), more than half of it does. With state 0 occurring even in *Acheloma* (Bolt, 1974; Dilkes & Reisz, 1987; arguably Olson, 1941) and *Phonerpeton* (Dilkes, 1990, 1993; D. M., pers. obs. of USNM 437796 and MCZ 2313), **state 1 does not occur in this matrix at all** despite having been scored for 18 OTUs in RC07; this makes the character parsimony-uninformative, so we have deleted it.

In the future, this character could be made informative by redefining it to describe how much of the ventral margin of the orbit the lacrimal forms, that is, how far the lacrimal extends caudally rather than rostrally to the rostral margin of the orbit. If redefined in such a way, however, this character would have to be merged with MAX 5/PAL 5 (see below).

21. MAX 3-9: **Caudal end of maxilla lying caudal to caudal margin of orbit (0), between caudal margin of orbit and caudal margin of vomer (1), at the same level as the caudal**

end of the vomer or rostral to it (2) (ordered). This character is ordered because potentially continuous characters should be ordered (Wiens, 2001; Grand et al., 2013).

RC07 used two separate characters, MAX 3 having the caudal margin of the orbit and MAX 9 the caudal margin of the vomer as the threshold. We have merged them because half of the states of these characters predicted each other – except in *Brachydectes* (see below), the vomer never extends caudal to the orbit, which means that MAX 3(0) predicted MAX 9(0), while MAX 9(1) required MAX 3(1).

State 0 occurs in *Cochleosaurus* (Sequeira, 2004), *Eocaecilia* (Jenkins, Walsh & Carroll, 2007), *Bruktererpeton* (Boy & Bandel, 1973), *Gephyrostegus* (most likely: Klembara et al., 2014) and *Lethiscus* (Pardo et al., 2017). We have also scored it for *Triadobatrachus*: although the caudal ends of the preserved fragments of the maxillae lie rostral of the caudal ends of the orbitotemporal fenestrae, they lie well caudal of the caudal margins of the largest possible eyes that would fit into the fenestrae (Ascarrunz et al., 2016: fig. 4, 12, 3D model 1).

State 1 is seen in *Batropetes* (Glienke, 2013, 2015) and most likely *Solenodonsaurus* (Danto, Witzmann & Müller, 2012). We have also scored it for *Brachydectes* where the caudal ends of orbit and maxilla variably coincide or nearly so (apparently independently of ontogeny – J. Pardo, pers. comm. – so that fig. 4A and 4C of Pardo & Anderson, 2016, represent different individuals), while the vomer has unusual caudal processes that extend farther caudal than both the orbit and the maxilla (Pardo & Anderson, 2016: fig. 4C).

Westlothiana has states 0 or 1 (Smithson et al., 1994). The same is the best score for Albanerpetidae (Venczel & Gardner, 2005: fig. 4, 8) and **Saharastega* (D. M., pers. obs. of MNN MOR 73). In several specimens of **Liaobatrachus*, however, the eyes themselves are preserved (Dong et al., 2013), so that its orbitotemporal fenestra does not prevent us from scoring state 0.

The maxillae of **Quasicaecilia* are unknown. State 0 is nonetheless ruled out by the fact that the jaw articulation lay well rostral of the caudal margin of the orbit; we have scored state 1 or 2.

22. MAX 5/PAL 5: **Ventral margin of the orbit formed by: lacrimal-jugal, prefrontal-jugal or lacrimal-postorbital suture (0); maxilla (1); lateral exposure of the palatine (2) (unordered).** This is another merger of two characters that partly predicted each other. State 0 includes cases where the mentioned suture is rostroventral to the orbit, so that the entire ventral margin is formed by the jugal alone. It also includes the unique condition of *Trimerorhachis* (excluding ?*T. sandovalensis*), where the jugal (let alone the maxilla and the palatine) is excluded from the orbit margin by a long lacrimal-postorbital suture (Milner & Schoch, 2013); making this condition a state of its own would be pointless, because it is unique, and would necessitate a stepmatrix for this character, so we have kept the score of 0 for *Trimerorhachis*.

When the jugal is absent (see JUG 1 below: *Triadobatrachus*, *Brachydectes*, **Beiyanerpeton*), state 0 and 1 cannot be distinguished; when the palatine is absent (see VOM 5-10/PAL 8/PTE 10-12-18/INT VAC 1 below: *Oestocephalus*; *Phlegethontia* as already scored by RC07), states 1 and 2 cannot be distinguished; when both are absent (*Karaurus*, *Valdotriton*, *Notobatrachus*, ?*Vieraella*, **Liaobatrachus*, **Pangerpeton*, **Chelotriton*), this character is entirely inapplicable.

Apateon was scored as showing state 1 in RC07, but acquired state 2 after metamorphosis, judging from the one known adult specimen of *A. gracilis* (Schoch & Fröbisch, 2006); we have scored state 2. *Schoenfelderpeton* was given state 1 or 2 by RC07; we have scored it (and also **Tungussogyrinus*) as unknown because the jugal does not (yet) extend to the region ventral to the orbit in the first place in the known specimens.

Among albanerpetids, only *Albanerpeton pannonicum* can be scored with confidence; it shows state 0. The dorsal margin of the maxilla is similar enough in all albanerpetids that Venczel & Gardner (2005) consider it likely that the same state prevailed throughout Albanerpetidae.

State 0 further occurs in *Ossinodus* (Warren, 2007).

Rhynchonkos has state 1 (Szostakiwskyj, Pardo & Anderson, 2015).

Sigurdsen & Green (2011: appendix 2) stated that this character was unknown in *Amphibamus*; Schoch & Milner (2014: fig. 30A), however, showed state 2 in a new reconstruction based in part on Schoch's personal observations. We have therefore tentatively scored state 2.

Acheloma dunni is a special case, in that (Polley & Reisz, 2011) the palatine and the ectopterygoid are exposed laterally (like in state 2) but do not participate in the orbit margin, being separated from the latter by a very long lacrimal-jugal suture (state 0). We have kept its score of 0, not least because *A. cumminsi* lacks a lateral exposure of the palatine altogether (Polley & Reisz, 2011), but caution that this may be ontogeny- or size-related: perhaps, as the orbit shrinks in relation to the rest of the skull, state 2 would generally turn into state 0 and leave such a lateral exposure of the palatine that does not participate in the orbit margin. This idea may be supported by the fact that in *Phonerpeton* (already scored as polymorphic), AMNH 7150 shows state 2 on the left side but possibly reaches state 0 on the right (Dilkes, 1990: fig. 3; D. M., pers. obs.).

*Caseasauria has states 0 and 1 (*Eothyris* has state 1, *Oedaleops* has state 0, and the condition in *Eocasea* is unknown: Reisz, Godfrey & Scott, 2009; Reisz & Fröbisch, 2014).

23. MAX 6: **Maxillary arcade closed (0) or open (1) posteriorly.** In state 1, the orbit is open (caudo)ventrally; (caudo)ventrally open temporal fenestrae as in *Oestocephalus* and most likely **Coloraderpeton* do not prevent state 0.

State 0 appears to be preserved in *Kotlassia* (Bulanov, 2003: fig. 30).

Schoenfelderpeton is now scored as unknown (like **Tungussogyrinus*), because the observed condition (state 1) is likely due to paedomorphosis, if not indeed larval age of the known individuals. This is based on the ontogeny of its close relative *Apateon*.

Brachydectes, in contrast, is now scored as possessing state 1; from what is known of the ontogeny of this "lepospondyl", there is no evidence it would ever have reached state 0 (Pardo & Anderson, 2016). The jugal and the quadratojugal bones are lacking entirely (see JUG 1 and QUAJUG 1 below).

Notobatrachus reigi has state 0 (Báez & Nicoli, 2008). As the jugal and the quadratojugal are absent in *N. degiustoi* (Báez & Nicoli, 2008), and the caudal end of the maxilla is not received by the palatine as it is in *Brachydectes*, we interpret the open maxillary arcade of *N. degiustoi* as inapplicable and have scored *Notobatrachus* as possessing state 0.

Unknown in *Triadobatrachus* (all sources).

24. MAX 7: **Dorsal margin of maxilla forming distinct dorsal 'step': no (0); yes (1).**

State 1 is not confined to pantylids – it occurs in *Broiliellus* (Carroll, 1964: fig. 9B) and *Doleserpeton* (Sigurdsen & Bolt, 2010) as well as some but not all specimens of **Glanochthon* (Schoch & Witzmann, 2009b). Surprisingly, it is also found in **Pholidogaster* (Panchen, 1975: fig. 15) and **Neopteroplax* (Romer, 1963: fig. 4 – less well visible in fig. 1), even though both have state MAX 8(0) and in the former the entire maxilla lies ventral to the nostril; these cases show that this character is independent from MAX 8.

**Platyoposaurus* shows a borderline condition (Gubin, 1991: drawing 3) that we have also counted as state 1. We have further counted the condition in **Erpetosaurus*, where the

nares lie so far dorsomedial that the maxillae, which have state MAX 8(0), have a long dorsomedial process to reach their ventrolateral margin (Milner & Sequeira, 2011).

25. MAX 8: **Dorsal margin of maxilla: low compared to naris and/or septomaxilla (0); tall and rounded (1); distinct process (2) (unordered).** The original wording mentions an "approximately rectangular flange", which is called "subrectangular" in the next sentence; such a condition does not occur in the taxon sample (except arguably **Iberospondylus*) – the dorsal process (if present) is always rounded and/or triangular to varying degrees. Furthermore, there were unambiguous mistakes in the scoring – *Triadobatrachus* was scored as possessing the (sub)rectangular process even though the whole region is unknown. Rather than jettisoning this character, we have coded the states that we see in the sampled taxa. The character is unordered because intermediates between 1 and 2 as well as directly between 0 and 2 exist, see below.

State 0: *Eusthenopteron*, *Panderichthys* and *Acanthostega* (Long & Gordon, 2004; Porro, Rayfield & Clack, 2015), *Ventastega* (Ahlberg, Lukševičs & Lebedev, 1994; Ahlberg et al., 2008), *Ichthyostega* (Ahlberg, Clack & Blom, 2005), *Colosteus* (Hook, 1983), *Greererpeton* (Smithson, 1982), *Crassigyrinus* (Clack, 1998), *Whatcheeria* (Lombard & Bolt, 1995), *Baphetes* and *Megalocephalus* (Beaumont, 1977), *Edops* (Romer & Witter, 1942; D. M., pers. obs. of MCZ 1378), *Chenoprosopus* (Hook, 1993), *Isodectes* (Sequeira, 1998), *Trimerorhachis* (Case, 1935; Milner & Schoch, 2013), *Balanerpeton* (Milner & Sequeira, 1994), Dendrerpetidae (A. R. Milner, 1980, 1996; Godfrey, Fiorillo & Carroll, 1987; Holmes, Carroll & Reisz, 1998), *Eryops* (Sawin, 1941), *Acheloma* (Bolt, 1974), *Ecolsonia* (Berman, Reisz & Eberth, 1985), *Broiliellus* (Carroll, 1964; Schoch, 2012), *Eoscopus* (Daly, 1994; Huttenlocker, Small & Pardo, 2007), *Platyrhinops* (Schoch, 2002: fig. 4), *Micromelerpeton* (Boy, 1995), *Eocaecilia* (Jenkins, Walsh & Carroll, 2007), *Caerorhachis* (Ruta, Milner & Coates, 2002), *Eoherpeton* (Panchen, 1975; Smithson, 1985), *Proterogyrinus* (Holmes, 1984), *Archeria* (Holmes, 1989), *Pholiderpeton attheyi* (Panchen, 1972), *Anthracosaurus* (Panchen, 1977), *Pholiderpeton scutigerum* (Clack, 1987b), *Bruktererpeton* (Boy & Bandel, 1973), *Gephyrostegus* (Brough & Brough, 1967), *Solenodonsaurus* (Laurin & Reisz, 1999), *Kotlassia* (Bulanov, 2003), *Discosauriscus* (Klembara, 1993, 1997; Klembara et al., 2006, 2007), *Seymouria* (Laurin, 2000; Klembara et al., 2005), *Diadectes* (Berman, Sumida & Lombard, 1992; Berman, Sumida & Martens, 1998), *Limnoscelis* (Romer, 1946; Berman, Reisz & Scott, 2010), *Captorhinus* (Heaton, 1979), *Paleothyris* (Carroll, 1969b), *Petrolacosaurus* (Reisz, 1981), *Westlothiana* (Smithson et al., 1994), all "microsaurs" (CG78; Berman, Eberth & Brinkman, 1988; Caroll, 1991; Glienke, 2013, 2015; Szostakiwskyj, Pardo & Anderson, 2015) except *Pantylus* (which has state 2: Romer, 1969; CG78), *Brachydectes* (Wellstead, 1991; Pardo & Anderson, 2016), *Acherontiscus* (Carroll, 1969a), *Dolichopareias* (Andrews & Carroll, 1991), all "nectrideans" (Jaekel, 1903: pl. II; Moodie, 1912; Bossy & Milner, 1998; Milner & Ruta, 2009) except *Diploceraspis* (unknown because the skull is so flattened and it is not clear if maxilla and lacrimal are separate: Beerbower, 1963), *Lethiscus* (Pardo et al., 2017), *Oestocephalus* (Carroll, 1998a), *Phlegethontia* (Anderson, 2002), *Ariekanerpeton* (Laurin, 1996b; Klembara & Ruta, 2005a), *Capetus* (Sequeira & Milner, 1993), *Orobates* (Berman et al., 2004), *Pederpes* (Clack & Finney, 2005), *Silvanerpeton* (Ruta & Clack, 2006), *Tseajaia* (Moss, 1972; D. M., pers. obs. of CM 38033) and *Utegenia* (Laurin, 1996c; Klembara & Ruta, 2004a).

State 1: *Cochleosaurus* (Sequeira, 2004: fig. 8A), *Phonerpeton* (Dilkes, 1990; D. M., pers. obs. of AMNH 7150 – counting the orbit and the lateral exposure of the palatine rather than the caudodorsal expansion of the naris), *Karaurus* (Ivachnenko, 1978), *Leptoropha* and *Microphon* (Bulanov, 2003), *Ossinodus* (Warren, 2007), arguably **Iberospondylus* (Laurin & Soler-Gijón, 2006) and apparently **Mordex* (Werneburg, 2012a: fig. 17b); a surprise

appearance occurs in **Aytonerpeton* (Clack et al., 2016: fig. 4 and especially supplementary video 2).

State 2: *Doleserpeton* (Sigurdsen & Bolt, 2010), *Apateon* (Werneburg, 1991: fig. 2, 5), *Leptorophus* (somewhat uncertain: Boy, 1986), *Schoenfelderpeton* (Boy, 1986), Albanerpetidae where codable, though close to state 0 (unknown in *Celtedens*: McGowan, 2002; unique condition in *Anoualerpeton priscum*: Gardner, Evans & Sigogneau-Russell, 2003: fig. 3D$_1$), *Pantylus* (Romer, 1969; CG78), *Notobatrachus* (like Albanerpetidae: Estes & Reig, 1973; Báez & Nicoli, 2004).

Unknown: *Eucritta* (Clack, 2001 – the fossils are so crushed and split through the bone that the photo, the specimen drawing, the reconstruction, and the text do not really cooperate), *Adelospondylus* and *Adelogyrinus* (Andrews & Carroll, 1991).

We have scored *Neldasaurus* as possessing state 0 or 1 because of its intermediate condition (Chase, 1965). *Amphibamus* is scored the same way based on Schoch (2001: fig. 4), because illustrations of its skull in lateral view do not seem to exist. *Vieraella* is likewise partially uncertain (Estes & Reig, 1973; Báez & Basso, 1996).

Valdotriton has state 1 or 2 ("The maxillae bear facial processes of uncertain size": Evans & Milner, 1996: 632).

Because *Eothyris* has state 1 on the right and state 2 on the left side (Reisz, Godfrey & Scott, 2009; D. M., pers. obs. of MCZ 1161), we have scored polymorphism for *Caseasauria.

All three states occur in **Glanochthon* (Schoch & Witzmann, 2009b: fig. 2).

26. FRO 1: **Frontal unpaired (0) or paired (1).**

State 0 is a very rare condition in this matrix. It is called 0 instead of 1 because RC07 assigned it to the outgroup (*Eusthenopteron*), but the large unpaired bone in the "snout mosaic" is just one of several possibilities for homologues of the frontals. (It is reminiscent of the "pineal plate" of stem-gnathostomes.) Judging from the fontanelle of *Ventastega* (Ahlberg et al., 2008) and *Acanthostega* (Clack, 2003b; Porro, Rayfield & Clack, 2015), it is at least as probable that the large median bone disappeared and lacks a homologue in limbed vertebrates (except possibly the sporadic interfrontal/interfrontonasal, see IFN 1 below), while one of the bone pairs lateral to it is homologous to the frontals. We have accordingly scored *Eusthenopteron* as unknown.

State 1 is present in *Ventastega* (Lukševičs, Ahlberg & Clack, 2003; Ahlberg et al., 2008).

27. FRO 2: **Frontals shorter than parietals (0), approximately equal in length (1), or longer than parietals (2) (ordered).** We have exchanged states 1 and 2 to make it possible to order this continuous character. In part, it appears, RC07 had already done that in the matrix but not in the character list.

State 1 or 2 is present in *Ventastega* (Ahlberg et al., 2008). We have also scored state 1 or 2 for *Crassigyrinus*: state 1 is present in the reconstruction by Clack (1998), which has the postfrontals meeting each other between the frontals and the parietals – given the stippled lines and poor preservation of the skull surface, we do not feel confident about this.

The frontals become longer in the ontogeny of *Apateon*; adults have state 2 as already scored (Schoch & Fröbisch, 2006). For this reason, we have scored *Leptorophus* as having state 1 or 2, and *Schoenfelderpeton* as unknown.

Neldasaurus (Chase, 1965, though arguably borderline), *Broiliellus* (Carroll, 1964; Schoch, 2012), *Pantylus* (Romer, 1969; CG78), *Adelogyrinus* (Andrews & Carroll, 1991), *Diceratosaurus*, *Diplocaulus* and *Ptyonius* (Bossy & Milner, 1998) and *Diploceraspis* (Beerbower, 1963) show state 0.

State 1 is found in *Trimerorhachis* (Milner & Schoch, 2013), *Micromelerpeton* (Schoch, 2009b: fig. 2b) and *Eocaecilia* (Jenkins, Walsh & Carroll, 2007).

Acanthostega (Clack, 2007; Porro, Rayfield & Clack, 2015), *Ichthyostega* (Clack & Milner, 2015: fig. 8), *Saxonerpeton*, *Pelodosotis* and *Rhynchonkos* (CG78; Szostakiwskyj, Pardo & Anderson, 2015: fig. 1), *Sauropleura* (Bossy, 1976, Bossy & Milner, 1998) and *Lethiscus* (Wellstead, 1982) show state 2.

The condition of *Pholiderpeton scutigerum* (Clack, 1987b), *Dolichopareias* (Andrews & Carroll, 1991) and *Ossinodus* (Warren, 2007) is unknown.

States 0 and 1 are reconstructed for different albanerpetids (Venczel & Gardner, 2005); we have scored both states. Both also seem to occur in different species of *Batropetes* (Glienke, 2013, 2015), where neither is clearly plesiomorphic.

States 1 and 2 are both found in *Balanerpeton* (left vs. right side: Milner & Sequeira, 1994) and *Platyrhinops* (Clack & Milner, 2010) as well as **Mordex* (usually left vs. right side: Werneburg, 2012a); in all of these cases we have scored polymorphism.

Complete frontals and parietals are apparently seldom found in the same specimen of **Branchiosaurus*; the largest ones where both seem to be preserved in full have state 1 (Werneburg, 2012a).

28. FRO 4: **Prefrontal/postfrontal suture (0); frontal contributes to orbit margin (1).** State 0 was originally called "Frontal excluded from […] orbit margin"; we have defined it more precisely and scored the character as inapplicable when the pre- and/or the postfrontal is absent, which is the case in Albanerpetidae, *Karaurus*, *Triadobatrachus*, *Valdotriton*, *Brachydectes*, *Notobatrachus* and *Vieraella* in the original taxon sample. However, because the aïstopod "postorbital" may be an ontogenetic fusion product of postorbital and postfrontal (Anderson, Carroll & Rowe, 2003: fig. 9B), we have scored the aïstopods: *Lethiscus* has state 1 (J. Pardo, pers. comm.; although the lateral process on the frontal mentioned by Pardo et al., 2017, but absent from their figures, appears not to exist, the frontal has a long contribution to the orbit margin), *Oestocephalus* has state 0 as already scored (Anderson, 2003a), and *Phlegethontia* has state 1 (Anderson, 2002, 2007a).

The condition is furthermore unknown in *Pholiderpeton scutigerum* (Clack, 1987b).

Ventastega (Ahlberg et al., 2008) shows state 0, as does *Asaphestera* (CG78).

Trimerorhachis is polymorphic (Milner & Schoch, 2013).

Apateon dracyi has state 0; but because Schoch & Milner (2008) and Fröbisch & Schoch (2009b) found this to be an unambiguous reversal within *Apateon*, we have kept the score of 1 for *Apateon*. Among branchiosaurids, state 0 does occur in **Tungussogyrinus* (Werneburg, 2009) and **Branchiosaurus* (Schoch & Milner, 2008; Werneburg, 2012a).

Klembara et al. (2014) have documented state 0 in *Gephyrostegus*.

CG78 reconstructed state 1 in *Saxonerpeton*. It is not evident why they did so, however; the drawings of the specimens do not indicate either state, and the skull table is not mentioned in the text at all. We have kept its score as unknown. Similarly, we have changed the score of *Odonterpeton* to unknown because CG78 reconstructed state 0 on the left but state 1 on the right side, mentioned in the text that the right postfrontal is not preserved, and included a specimen drawing that does not clarify the situation.

It is possible (Sumida, Pelletier & Berman, 2014) that *Oedaleops* is polymorphic, specifically that some specimens show state 0 rather than the state 1 preserved in others (Reisz, Godfrey & Scott, 2009; Sumida, Pelletier & Berman, 2014). For the time being, we have ignored this and scored only state 1 for *Caseasauria; state 1 is seen in *Eothyris* (Reisz, Godfrey & Scott, 2009), and the condition in *Eocasea* is unknown.

The smallest specimen of **Acanthostomatops* probably has state 1, but all others clearly show state 0 (Witzmann & Schoch, 2006a), so we have scored *Acanthostomatops* as possessing state 0, even though this character does not show ontogenetic variation elsewhere.

The holotype of **Konzhukovia vetusta* is polymorphic, with the left side showing state 0 and the right side state 1 (Gubin, 1991: drawing 6a).

Like *Brachydectes* (see above), **Beiyanerpeton* possesses prefrontals but lacks postfrontals; yet, the frontal does not contribute to the orbit margin, because the prefrontals contact processes of the parietals that look as if the postfrontals had fused to the parietals. This intriguing condition is common in extant salamanders, yet absent in *Karaurus* and, as far as known, all other Mesozoic salamanders except **Beiyanerpeton* (ch. 61 of Gao & Shubin, 2012) – *Valdotriton* has the processes, but they may not have reached the prefrontals (Evans & Milner, 1996: fig. 6b); for this reason, and because it is restricted to a single OTU in the original taxon sample, we have refrained from coding this condition as a third state of the present character. **Beiyanerpeton* is scored as unknown.

One specimen of **Platyoposaurus* has state 1 on the right side (Gubin, 1991: 34). We have counted this as polymorphism.

29. FRO 5: **Coossified frontal and parietal (frontoparietal bone): absent (0); present (1).**

Ventastega has state 0 (Ahlberg et al., 2008).

There is no evidence of a preserved frontal or a preserved rostral edge of a parietal in **Sparodus* (D. M., pers. obs. of NHMW 1899/0003/0006); the "frontal" in the mirrored fig. 1A of Carroll (1988) is the right prefrontal, as is already clear by comparison to the left side of the same figure. The most defensible score is therefore "?", contra Carroll (1988).

30. FRO 6: **Frontal anterior margin deeply wedged between nasal posterolateral margins for at least 1/3 of the length of the nasals: absent (0); present (1).** We interpret state 1 as meaning that both frontals together form such a wedge.

Ventastega has state 0 (Ahlberg et al., 2008), as do *Adelospondylus*, *Adelogyrinus* and *Dolichopareias* (Andrews & Carroll, 1991) and *Lethiscus* (Pardo et al., 2017). Although *Limnoscelis* has a conspicuous wedge, it has state 0 as well (Berman, Reisz & Scott, 2010: fig. 3); *Gephyrostegus*, too, does not reach state 1 (Klembara et al., 2014).

State 1 is found in *Acanthostega* (Porro, Rayfield & Clack, 2015), Albanerpetidae (Venczel & Gardner, 2005) and *Phlegethontia* (Anderson, 2007a).

Danto, Witzmann & Müller (2012) followed RC07 in scoring *Solenodonsaurus* as possessing state 1, but, according to their figures, the wedge is shallow or absent (as far as it is not obscured by the strong interdigitation). We have assigned state 0 to it.

The only known skull of *Westlothiana* is polymorphic, showing state 0 on the left and 1 on the right side (Smithson et al., 1994).

In *Sauropleura pectinata*, state 1 (which was scored in RC07) was not quite reached: although a long wedge is present, the nasals participate in the extreme elongation of the tip of the snout. Because this elongation is autapomorphic, we have kept state 1 for *Sauropleura*, as found in *S. scalaris* (both known ontogenetic stages) and *S. bairdi* (Bossy & Milner, 1998: fig. 53A–D, 58A, 72A, 73A).

**Glanochthon* is polymorphic (Schoch & Witzmann, 2009b: fig. 2).

In **Palaeoherpeton* (Panchen, 1964: fig. 11), state 1 is reached on the right side but not on the left, even though the suture on the left side runs rostromedial to caudolateral as well; we have scored polymorphism.

31. PAR 1: **Supratemporal/postparietal suture (0); parietal/tabular suture (1).** This character is inapplicable when any of these bones are missing. RC07 did not specify state 0

(calling the character "Parietal/tabular suture: absent (0); present (1)"), leading them to score many cells in ways predictable from other cells.

Megalocephalus is polymorphic: state 1 is found on the right side of the holotype, state 0 everywhere else (Beaumont, 1977: 52).

The supratemporal is missing, as far as known, in all albanerpetids (except possibly ***Shirerpeton*), as well all "lepospondyls" except the following, which all have state 1 (as already scored for all except the last two): *Westlothiana* (Smithson et al., 1994), *Ptyonius* and *Sauropleura* (Bossy & Milner, 1998), *Lethiscus* (Pardo et al., 2017) and *Oestocephalus* (Anderson, 2003a: fig. 3C).

By comparison with *Oestocephalus*, **Coloraderpeton* and to a lesser degree *Lethiscus* (Anderson, 2003a; Pardo et al., 2017) it seems evident that the supposed right supratemporal of **Pseudophlegethontia* (Anderson, 2003b) is the process of the parietal that contacts the tabular. (On the left side, however, the bone dorsomedial of the squamosal probably is a supratemporal; we have scored it as such in the next and other characters below.) The supposed postfrontal may well belong to the parietal, too, for the same reasons. We have scored state 1 of the present character.

32. PAR 2/POSFRO 3/INTEMP 1/SUTEMP 1: **Intertemporal present (0); supratemporal/postfrontal contact (1); parietal/postorbital contact, supratemporal present (2); parietal/postorbital or parietal/squamosal contact, supratemporal absent (3); tabular/postfrontal contact, supratemporal absent (4) (stepmatrix).** Four redundant characters have been merged: no known limbed vertebrate that has an intertemporal (INTEMP 1(1) in the original) lacks a supratemporal (SUTEMP 1(0)); states 1 through 3 track gradual shrinking of the supratemporal (its rostral end reaches the postfrontal in state 1, but not in state 2, and in states 3 and 4 the bone is entirely absent); all states other than 0 are impossible when the intertemporal is present; and a parietal-postorbital contact (states 2 and 3, originally PAR 2(1)) is impossible when the supratemporal is too large (states 0 and 1) as well as when the tabular contacts the postfrontal (state 4, originally POSFRO 3(1)). The stepmatrix for this character is Appendix-Table 2.

Whenever there is an intertemporal, it contacts the postfrontal and the supratemporal, separating the parietal and the postorbital. The only possible exception is the right side of one specimen of *Greererpeton* (Smithson, 1982): it has a tiny extra bone that could be an atavistic reappearance of the intertemporal (even though a long parietal-postorbital contact is present) or a pathological neomorph; see various "branchiosaurs" for supposed occurrences of such phenomena (Boy, 1972). We have therefore scored *Greererpeton* as possessing state 2 of this character (and as unknown for the INTEMP characters, see below).

Mentioning the squamosal in state 3 accounts for *Eocaecilia*, which is unique in this matrix in combining absence of the postorbital with presence of the tabular and the postfrontal. Taxa that lack not only the supratemporal but also the tabular and/or postfrontal (*Acherontiscus*, Adelogyrinidae, *Odonterpeton*, *Brachydectes*, modern amphibians other than *Eocaecilia*, *Phlegethontia* and **Quasicaecilia*) are scored as having state 3 or 4.

State 0 is present in *Panderichthys* (Vorobyeva & Schultze, 1991) and in *Ventastega* (Lukševičs, Ahlberg & Clack, 2003; Ahlberg et al., 2008).

Solenodonsaurus has state 1 according to Danto, Witzmann & Müller (2012).

The condition of *Dolichopareias* is wholly unknown (Andrews & Carroll, 1991).

Although the description of this character does not quite fit *Lethiscus* or *Oestocephalus*, where the parietal laterally participates in the margin of the temporal fenestra and a single bone occupies the places where the postfrontal and the postorbital would be expected, we have kept state 2 for both: in *Oestocephalus*, the supratemporal and/or the "postfrontal" would have to be extremely long and narrow to meet each other along the unusually elongate parietal

(Anderson, 2003b: fig. 2, 3C); in *Lethiscus*, the parietal even has a wide lateral flange between the caudal margin of the "postorbital" and the rostral margin of the supratemporal (Wellstead, 1982; Pardo et al., 2017), so that the latter two bones would again need to have highly unusual shapes to meet each other if the temporal fenestra were absent. – Much the same holds for **Coloraderpeton* (Anderson, 2003a; Pardo et al., 2017), which we have also scored 2.

To account for its possible intertemporal, we have scored *Ossinodus* as showing state 0 or 2.

**Spathicephalus* is polymorphic, with state 1 in *S. marsdeni* and state 2 in *S. mirus* (Smithson et al., 2017).

**Pseudophlegethontia* has state 0, 1 or 2 following the argument under the preceding character.

For Analysis EB, this character is split as follows:
PAR 2/POSFRO 3: **Postfrontal contacts supratemporal or tabular (0); parietal contacts postorbital or squamosal (1).** Inapplicable when the intertemporal is present, and also when the tabular or the postfrontal is absent.
INTEMP 1/SUTEMP 1: **Intertemporal and supratemporal present (0); intertemporal absent, supratemporal present (1); both absent (2) (ordered)**.

Appendix-Table 2: Stepmatrix for character 32 (PAR 2/POSFRO 3/INTEMP 1/SUTEMP 1).

**from ↓ to →**	**0**	**1**	**2**	**3**	**4**
**0**	0	1	1	2	3
**1**	1	0	1	1	2
**2**	1	1	0	1	2
**3**	2	1	1	0	1
**4**	3	2	2	1	0

33. PAR 4: **Anterior margin of parietal lying in front of (0), level with (1), or behind (2) orbit midlength (ordered).** We have ordered this continuous character.

Because of the orbitotemporal fenestra, batrachians (already done for *Karaurus* by RC07) are scored as having their observed state (corresponding to the clearly wrong assumption that the entire orbitotemporal fenestra is homologous to the orbit) or higher. In the case of *Valdotriton*, this means a score as unknown.

State 1 is present in *Ventastega* (Ahlberg et al., 2008) and arguably *Eryops* (D. M., pers. obs. of MCZ 1129).

State 2 is recorded in Albanerpetidae (Venczel & Gardner, 2005), *Kotlassia* (Bulanov, 2003), *Brachydectes* (Pardo & Anderson, 2016) and *Ossinodus* (Warren, 2007).

**Sparodus* is best scored as unknown, see FRO 5 above.

**Archegosaurus* has state 1 on the left and state 2 on the right side in at least two individuals (Witzmann, 2006).

Even if the caudal angle of the left orbit (indenting the postorbital) is ignored as a taphonomic artefact, **Neopteroplax* reaches state 1 on the left side – quite likely not on the right, but most of the margin of the right orbit is not preserved – according to the specimen drawing (Romer, 1963: fig. 1) but not the reconstruction (fig. 3). While the text mentions dorsoventral compression, we cannot imagine what kind of rostrocaudal shear could be compatible with the specimen drawing; given the precedent of **Archegosaurus*, we have scored

states 1 and 2 in order to avoid scoring just state 1 as a certainty. (There is currently no way of scoring "state 1 and possibly state 2".)

34. PAR 5: **Anteriormost third of parietals not wider (0) or at least marginally wider (1) than frontals.**

State 0 is present in *Ventastega* (Ahlberg et al., 2008), *Ichthyostega* (Jarvik, 1996; Clack & Milner, 2015: fig. 8), *Cochleosaurus* (Sequeira, 2004), *Ecolsonia* (Berman, Reisz & Eberth, 1985), *Broiliellus* (Carroll, 1964; Schoch, 2012), *Amphibamus* (Schoch, 2001), *Micromelerpeton* (Boy, 1995: fig. 8), *Apateon* (throughout its ontogeny: Schoch & Fröbisch, 2006), *Karaurus* (Ivachnenko, 1978), *Gephyrostegus* (Klembara et al., 2014), *Tuditanus*, *Sax-onerpeton* and *Hapsidopareion* (CG78), *Rhynchonkos* (Szostakiwskyj, Pardo & Anderson, 2015) and *Lethiscus* (Pardo et al., 2017).

State 1 is found in *Acanthostega* (Porro, Rayfield & Clack, 2015).

Dendrerpetidae is polymorphic: while *Dendrysekos* has state 1 as scored in RC07 (Holmes, Carroll & Reisz, 1998), most or all specimens of *Dendrerpeton* (all species: A. R. Milner, 1980, 1996) have state 0. **Acanthostomatops* (Witzmann & Schoch, 2006a) and **Glanochthon* (Schoch & Witzmann, 2009b) are polymorphic as well. While only state 1 is known in the single individual of *Batropetes niederkirchensis* (Glienke, 2013: fig. 2), *B. frit-schi* is polymorphic (Glienke, 2013: fig. 6), as is the reconstructed individual of *B. palatinus* because its two parietals have such different lengths (Glienke, 2015: fig. 1E).

Unclear and probably about equal in *Edops* (D. M., pers. obs. of MCZ 1378; the reconstruction by Romer & Witter [1942] just barely indicates state 1, but seems not to ac-knowledge a patch of reconstructed surface that probably covers at least part of the parietal/ postfrontal suture).

A (separate) parietal is absent in *Triadobatrachus* (see FRO 5 above), *Phlegethontia*, *Notobatrachus* and *Vieraella*; we have accordingly scored them as unknown.

The state of **Pseudophlegethontia* depends on whether the supposed postfrontal belongs to the parietal (see ch. 31, PAR 1, above); we have scored it as unknown.

35. PAR 6: **Parietals more (0) or less (1) than two and a half times as long as wide each.** "Each" is added; we have interpreted it from the coding, which makes clear that this character does not concern the combined widths of both parietals.

Brachydectes is apparently polymorphic, with Permian specimens having state 1 and Carboniferous specimens reaching state 0 (Wellstead, 1991).

**Sparodus* is probably borderline, but unknown, see FRO 5 above.

**Lydekkerina* is polymorphic (Jeannot, Damiani & Rubidge, 2006).

36. PAR 7: **Squamosal participates in dorsal surface of skull roof: no (0); yes (1).** The original wording, "Parietal/squamosal suture extending in part onto the dorsal surface of the skull table: no (0); yes (1)", is not applicable to the vast majority of the taxon sample, because the parietal and the squamosal are separated by other bones; yet, all those OTUs were scored as possessing state 0. We therefore speculate that our wording, which can be applied to all taxa in this matrix, was intended.

Skutschas & Martin (2011) suggested that state 1 results from fusion of the supratemporal to the squamosal. While it is not testable (except by phylogenetic bracketing) if this has happened in the extinct salamanders they had in mind (ontogenetic series are not known), state 1 occurs in several taxa in this matrix that retain a separate supratemporal. One example is *Ichthyostega* (Clack & Milner, 2015: fig. 8). Another is the one dendrerpetid skull that is not squished flat (Holmes, Carroll & Reisz, 1998) – which leaves us to suspect that

most small temnospondyls should actually be scored 1 as well, although we have remained conservative and kept state 0 for all except Dendrerpetidae.

Triadobatrachus is somewhat disarticulated and crushed flat, making it difficult to judge whether the parietal/squamosal contact was on the dorsal or the lateral side of the skull roof; we have scored it as unknown.

Captorhinus is borderline in that the parietal-squamosal suture forms the boundary between the skull table and the cheek (Heaton, 1979); unlike RC07, we have chosen to count this as state 0. State 0 is further present in *Ventastega* (Ahlberg et al., 2008), *Paleothyris* (Carroll, 1969b), *Batropetes* (Carroll, 1991; Glienke, 2013), *Microbrachis* (Olori, 2015) and *Tseajaia* (Moss, 1972). Under our reinterpretation of this character, state 0 is also found in Albanerpetidae (McGowan, 2002; Venczel & Gardner, 2005) and *Notobatrachus* (Estes & Reig, 1973; Báez & Nicoli, 2004).

We retain the score of *Petrolacosaurus* (Reisz, 1981) as possessing state 1 because we consider the dorsally-facing supratemporal fenestrae to form part of the dorsal surface of the skull.

We assign state 1 to **Acanthostomatops* because the squamosal extends medially beyond the lateral margin of the supratemporal (Witzmann & Schoch, 2006a); the skull is apparently very flat, and illustrations (let alone reconstructions) in lateral view seem not to exist, but the supratemporal generally does not participate in the lateral skull surface.

37. PAR 8: **Parietal/frontal suture strongly interdigitating: no (0); yes (1).**

State 0 is present in *Ventastega* (Ahlberg et al., 2008).

Although "strongly" is not defined, we have scored *Kotlassia* as possessing state 0 (Bulanov, 2003: fig. 30).

Apateon has state 1 based on the largest metamorphic individual (Werneburg, 1991: fig. 5b). State 1 is further found in *Baphetes* (Milner, Milner & Walsh, 2009), *Edops* (Romer & Witter, 1942; D. M., pers. obs. of MCZ 1378), *Trimerorhachis* (Milner & Schoch, 2013), *Balanerpeton* (Milner & Sequeira, 1994) and *Diadectes* (Berman, Sumida & Lombard, 1992; Berman, Sumida & Martens, 1998). We have further assigned state 1 to *Gephyrostegus* based on Klembara et al. (2014: especially fig. 1A, 5B).

Batropetes is polymorphic (Glienke, 2013, 2015).

Hapsidopareion probably qualifies for state 1 (CG78: fig. 13A), which we have therefore scored.

Unknown in *Acanthostega* (Porro, Rayfield & Clack, 2015).

Phlegethontia lacks parietals (Anderson, 2002), so we have scored it as unknown.

Assuming that the existing reconstructions of *S. mirus* are too schematic or thick-lined to score this character, we have scored state 1 for **Spathicephalus* based on *S. marsdeni* (Smithson et al., 2017: fig. 3C).

38. PAR 9: **Parietal/postparietal suture strongly interdigitating: no (0); yes (1).** RC07 stated that "[t]here appears to be no clear phylogenetic signal associated with this character". It has at most 28 steps on the shortest trees from Analysis R4 (158 OTUs); state 1 is an autapomorphy of Temnospondyli except *Eucritta* (reversed in part of *Colosteus*, in *Megalocephalus*, Cochleosauridae, *Eryops*, *Neldasaurus* and in most dissorophoids, with a reappearance in **Gerobatrachus*), of *Archeria*, of **Palaeoherpeton* + *NSM 994 GF 1.1 + **Neopteroplax*, of *Solenodonsaurus*, *Diadectes*, *Caseasauria, *Pantylus*, *Hyloplesion* and *Keraterpeton*, and holds the aïstopod-urocordylid-adelospondyl clade together; it may also be homologous between **Sparodus*, **Llistrofus* and *Brachydectes*.

Kotlassia has state 0 (Bulanov, 2003: fig. 30); *Eoscopus* is somewhat borderline (Daly, 1994), but we prefer scoring it as sharing state 0.

State 1 occurs in *Acanthostega* (Clack, 2007; Porro, Rayfield & Clack, 2015), *Baphetes* (Beaumont, 1977: fig. 25; Milner, Milner & Walsh, 2009), *Edops* (Romer & Witter, 1942; D. M., pers. obs. of MCZ 1378), *Trimerorhachis* (Milner & Schoch, 2013), *Balanerpeton* (Milner & Sequeira, 1994), *Diadectes* (Berman, Sumida & Lombard, 1992; Berman, Sumida & Martens, 1998) and *Limnoscelis* (Reisz, 2007; Berman, Reisz & Scott, 2010), probably also **Saharastega* (D. M., pers. obs. of MNN MOR 73).

Unclear in *Hapsidopareion* (CG78: fig. 13A).

This character is inapplicable to *Odonterpeton* because *Odonterpeton* lacks postparietals, as explained in the next character.

Assuming that the existing reconstructions of *S. mirus* are too schematic or thick-lined to score this character, we have scored state 1 for **Spathicephalus* based on *S. marsdeni* (Smithson et al., 2017: fig. 3C).

**Bystrowiella* has state 1 on the ventral side, but state 0, which we have scored, on the dorsal side (Witzmann & Schoch, 2017: 716, fig. 2).

39. POSPAR 1-2: **Postparietal(s) paired (0), single (1), or absent (2) (unordered).** The two original characters, presence/absence of postparietals and absence/presence of median fusion of the postparietals, make each other inapplicable, so we have fused them.

State 0 is present in *Ventastega* (Ahlberg et al., 2008).

States 0 and 1 are known in *Ichthyostega* (Clack & Milner, 2015); in the absence of evidence on whether this could be ontogenetic, we have scored polymorphism.

There is no evidence for postparietals in any albanerpetid, so we have scored Albanerpetidae as having state 2.

Bruktererpeton has state 0 or 1 (Boy & Bandel, 1973). We have scored **Gerobatrachus* the same way because it is not evident whether the sutured fragment close to the caudal end of the skull table belongs to the parietals or the postparietals.

Odonterpeton was interpreted as possessing state 1 by CG78. However, the supposed suture between the left parietal and the postparietal consists of a discontinuous series of cracks (D. M., pers. obs. of USNM 4465+4467, the holotype). We have accordingly assigned state 2 to *Odonterpeton* and would like to use this opportunity to mention that fig. 98A and 99A of CG78 differ from the specimen in other ways as well; they are idealized and simplified to the point of being unreliable. In particular, the suture between the right parietal and the "postparietal" has an additional curve to the left at its caudal end, so that the asymmetry between the parietals is considerably smaller than one would think.

The condition is unknown in *Adelogyrinus* and *Dolichopareias* (Andrews & Carroll, 1991).

Although a stippled line indicates a suture in the reconstruction drawings of *Lethiscus* by Pardo et al. (2017), it is not apparent in the scan images or in Wellstead (1982); we have kept state 0 or 1 for the moment.

As we have not seen the type specimen, we have trouble understanding the condition of *Tseajaia*: Moss (1972: 10) reported state 1, and his photos (pl. 1, 3) are compatible with this – if they show a suture, then that suture is much thinner than the others in that skull. Berman, Sumida & Lombard (1992: 490) said explicitly that Moss was wrong (reiterated by Berman, Reisz & Scott, 2010: 192) and presented another photo of the same skull (the holotype, UCMP 59012) which is much brighter, shows drastically narrower sutures, and lacks a strong contrast between the suture between the postparietals and the other sutures. It looks like all sutures in that photo are enhanced in black ink, though. In the photo of UCMP 59012 shown by Reisz (2007: pl. 26), the postparietals look very clearly separate. D. M. has seen CM 38033, a largely complete skeleton containing a complete skull illustrated by Berman, Sumida & Lombard (1992: fig. 9-2, 10-2), but this does not help for this character,

because the occipital region is preserved as tiny fragments and there is generally no safe way of distinguishing sutures from cracks in this specimen; indeed, Berman, Sumida & Lombard (1992: fig. 10-2) did not reconstruct the postparietal(s) of the skull of CM 38033, and while Reisz (2007) briefly described that skull, he did not mention this feature and did not illustrate it. Supported by Reisz (2007: pl. 26), we accept the more recent interpretation (Berman, Sumida & Lombard, 1992; Berman, Reisz & Scott, 2010) and have kept the score of *Tseajaia* as 0, unusual though this is for a diadectomorph (Reisz, 2007: 243, listed “single median postparietal” as an autapomorphy of Diadectomorpha; Berman, Reisz & Scott, 2010, repeated that the postparietal is single in *Limnoscelis*, *Orobates* and *Diadectes* as scored by RC07).

*Caseasauria is polymorphic: state 0 is found in *Eothyris* and *Eocasea*, state 1 in *Oedaleops* (Reisz, Godfrey & Scott, 2009; Reisz & Fröbisch, 2014).

Reisz & Dilkes (2003) were cautious, but we accept their argument for state 0 in **Archaeovenator*.

40. POSPAR 3-6: **Dorsally exposed part of postparietals together less (0) or more than four times as wide (mediolaterally) as long (1) or absent, postparietals entirely on occipital surface of skull (2) (ordered).** We have fused two characters (POSPAR 3: size of dorsal exposure; POSPAR 6: presence of dorsal exposure) that we consider parts of a single continuous character. The original wording had “postparietal” instead of “postparietals together”, but that does not remotely fit the original scores, so it was probably not intended.

State 0 is present in *Ventastega* (Ahlberg et al., 2008), *Ichthyostega* (Clack, 2007), *Eucritta* (Clack, 2001), *Isodectes* (Sequeira, 1998), Dendrerpetidae (Holmes, Carroll & Reisz, 1998), *Eryops* (D. M., pers. obs. of MCZ 1129), *Acheloma* (Polley & Reisz, 2011), *Phonerpeton* (Dilkes, 1990), *Ecolsonia* (Berman, Reisz & Eberth, 1985), *Platyrhinops* (Clack & Milner, 2010), *Micromelerpeton* (Schoch, 2009b: fig. 2b), *Apateon*, *Leptorophus* and *Schoenfelderpeton* (Schoch & Milner, 2008), *Bruktererpeton* (Boy & Bandel, 1973), probably *Solenodonsaurus* (Danto, Witzmann & Müller, 2012), *Discosauriscus* (Klembara, 1997; Klembara et al., 2006), *Seymouria* (Laurin, 1996a; Klembara et al., 2005), *Microbrachis* (Vallin & Laurin, 2004; Olori, 2015), *Lethiscus* (Wellstead, 1982; Pardo et al., 2017), and *Ariekanerpeton*, *Leptoropha* and *Microphon* (Bulanov, 2003).

State 1 is found in *Amphibamus* (Schoch & Milner, 2014: fig. 30B; possibly Daly, 1994: fig. 18) and *Limnoscelis* (Reisz, 2007), and in *Diploceraspis* because of its “horns” (Beerbower, 1963).

Diadectes possesses both state 0 (North American species: Berman, Sumida & Lombard, 1992) and state 1 (*D. absitus*: Berman, Sumida & Martens, 1998).

Saxonerpeton has state 1 (CG78: fig. 17).

Hyloplesion has state 2 (CG78; D. M., pers. obs. of NHMW 1983/82/54 and other NHMW specimens).

Pelodosotis was scored POSPAR 3(0) and POSPAR 6(?) in RC07. Of these contradictory scores, the former is correct (CG78); *Pelodosotis* thus possesses state 0 of the present character.

Adelogyrinus and *Dolichopareias* have state 0 or 1 (Andrews & Carroll, 1991).

Because the skull roof of **Gerobatrachus* is exposed in ventral view only, we have scored it as having state 0 or 1.

*Caseasauria shows both state 1 (in *Eothyris* and possibly *Eocasea*; Reisz, Godfrey & Scott, 2009; Reisz & Fröbisch, 2014) and state 2 (*Oedaleops*; Reisz, Godfrey & Scott, 2009).

41. POSPAR 4-8: **Edge between the dorsal and the caudal surfaces of the skull lacking (0) or possessing (1) a caudal process in the midline.** This is a fusion of two characters we deem not merely correlated but identical, with POSPAR 4 (“Postparietals without (0) or with

(1) median lappets") meaning the combination of a caudal process with a vertical occipital surface and POSPAR 8 ("Postparietals without (0) or with (1) sinuous posterior ridge") the combination of a caudal process with an inclined occipital surface. (The difference between a vertical and an inclined surface is character POSPAR 7, see below.) Fitting this interpretation, POSPAR 8(1) was only scored for *Micraroter* and *Pelodosotis*, while POSPAR 4(1) was limited to *Crassigyrinus*, *Whatcheeria*, embolomeres other than *Eoherpeton*, and *Ptyonius*. – Our wording makes the character applicable to taxa that lack postparietals; in particular, *Triadobatrachus* (all sources and pers. obs.) has state 0, and *Batropetes* has state 1 (Glienke, 2013, 2015).

Crassigyrinus in fact has a very clear case of state 0 (Panchen, 1985; Clack, 1998). We have also kept state 0 for *Micromelerpeton* and *Apateon* because this condition is seen in the most mature known specimens (Schoch, 2009b: fig. 2b; Schoch & Fröbisch, 2006).

State 1 is present in *Ventastega* (Ahlberg et al., 2008), *Balanerpeton* (weakly: Milner & Sequeira, 1994), Dendrerpetidae (weakly: A. R. Milner, 1980, 1996; Holmes, Carroll & Reisz, 1998), *Platyrhinops* (Clack & Milner, 2010), *Eocaecilia* (Jenkins, Walsh & Carroll, 2007), *Diadectes* (Berman, Sumida & Lombard, 1992; very weakly expressed in *D. absitus*, but present throughout [Berman, Sumida & Lombard, 1998]; see also Case, 1910), *Asaphestera* (where the caudal process comprises the entire caudal edge of the postparietal; CG78), *Brachydectes* (Pardo & Anderson, 2016: fig. 3D, 4B), *Oestocephalus* (Carroll, 1998a; Anderson, 2003a), *Capetus* (Sequeira & Milner, 1993), *Orobates* (Berman et al., 2004), and *Tseajaia* (Moss, 1972; Berman, Sumida & Lombard, 1992; Reisz, 2007: pl. 26; D. M., pers. obs. of CM 38033).

Solenodonsaurus was scored POSPAR 4(?) but POSPAR 8(0) in RC07. The latter is correct according to Danto, Witzmann & Müller (2012).

Ossinodus was scored in the same ways in RC07. Here, we have kept the question mark, because precisely that part of the postparietals is not preserved (Warren, 2007).

State 1 is seen in a skull fragment referred to *Baphetes kirkbyi* (Beaumont, 1977: fig. 20(*a*)); this area is damaged in the holotype of that species (Beaumont, 1977: fig. 18), but *B. orientalis* has state 0 (Beaumont, 1977: fig. 25), so we have scored polymorphism for *Baphetes*.

Likewise, *Sauropleura* is polymorphic: *S. scalaris* and *S. pectinata* have state 1, but *S. bairdi* shows state 0 (Bossy & Milner, 1998: fig. 53). **Micropholis* has both states as well (Schoch & Rubidge, 2005: fig. 3).

Probably unknown in *Kotlassia* (Bulanov, 2003: fig. 28–30); we have changed the score.

Unclear in *Lethiscus* (Pardo et al., 2017).

Because of possible damage (D. M., pers. obs. of MNN MOR 70), we have scored **Nigerpeton* as unknown.

Though damage makes MNN MOR 73 difficult to interpret (D. M., pers. obs.), **Saharastega* most likely has state 0.

Gently rounded but present (state 1) in **Beiyanerpeton* (Gao & Shubin, 2012: fig. 3).

POSPAR 5 is merged with OPI 2, see below.

42. POSPAR 7: **Postparietals without (0) or with (1) posteroventrally sloping occipital exposure.** Only unusually low angles count as state 1; the occipital surface is almost never perfectly vertical.

State 0 is present in *Ventastega* (Ahlberg et al., 2008).

State 1 occurs in *Limnoscelis* (Reisz, 2007; Berman, Reisz & Scott, 2010), *Oestocephalus* (Carroll, 1998a; Anderson, 2003a) and *Orobates* (Berman et al., 2004).

Diadectes is polymorphic (Berman, Sumida & Martens, 1998: 57).

The condition is unknown in *Westlothiana* (Smithson et al., 1994).

Adults of **Acanthostomatops* have state 0 (Witzmann & Schoch, 2006a).

43. POSPAR 9: **Edge between the dorsal and the caudal surfaces of the skull without (0) or with (1) broad, concave posterior emargination in the central bones.** Our wording makes the character applicable to taxa whose postparietals lie entirely on the occipital surface (POSPAR 3-6(2)) or are absent (POSPAR 1-2(2)); namely, *Triadobatrachus* (all sources) has state 0, while *Captorhinus* (Heaton, 1979) and *Batropetes* (Glienke, 2013, 2015) have state 1, though *B. fritschi* may be considered borderline (Glienke, 2013).

State 0 is present in *Ventastega* (Ahlberg et al., 2008). We have also assigned it to **Saharastega*, though damage makes MNN MOR 73 difficult to interpret (D. M., pers. obs.).

According to RC07, state 1 is limited to "some" diplocaulids (i.e. all except *Keraterpeton*); however, other than in *Captorhinus* and *Batropetes* as mentioned above, it is also found in *Ichthyostega* (Clack & Milner, 2015: fig. 8), *Edops* (arguably borderline: D. M., pers. obs. of MCZ 1378), *Trimerorhachis* (Milner & Schoch, 2013), Dendrerpetidae (weakly: A. R. Milner, 1980, 1996; Holmes, Carroll & Reisz, 1998), *Acheloma* (borderline: Polley & Reisz, 2011), *Eoscopus* (Daly, 1994), *Discosauriscus* (Klembara, 1997; Klembara et al., 2006), *Seymouria* (Laurin, 1996a: fig. 3A; Klembara et al., 2006), *Diadectes* (Berman, Sumida & Lombard, 1992: fig. 1, 3), *Limnoscelis* (Romer, 1946; Fracasso, 1983; Reisz, 2007; Berman, Reisz & Scott, 2010), *Brachydectes* (Pardo & Anderson, 2016: fig. 4B), *Oestocephalus* (Carroll, 1998a; Anderson, 2003a), *Ariekanerpeton* (Klembara & Ruta, 2005a), *Capetus* (Sequeira & Milner, 1993), *Orobates* (marginally: Berman et al., 2004) and *Ossinodus* (Warren, 2007), as well as in **Micropholis* (Schoch & Rubidge, 2005), **Nigerpeton* (D. M., pers. obs. of MNN MOR 70) and **Sclerocephalus* (only the type species, the only one considered here, and only in late ontogenetic stages: Schoch & Witzmann, 2009a).

Although the reconstruction of *Baphetes kirkbyi* by Beaumont (1977: fig. 21) shows an almost straight margin as previously scored, the specimen drawings in the same work (fig. 18, 20) clearly show state 1, which is also shown in both the specimen drawing and the reconstruction of *B. orientalis* (Beaumont, 1977: fig. 25).

Acanthostega appears to be polymorphic (Porro, Rayfield & Clack, 2015: fig. 3E, 4B). So is *Megalocephalus* (Beaumont, 1977: fig. 10(*b*), 11(*a*)).

Eryops is polymorphic, the broad-skulled morph having state 0 and the narrow-skulled morph, to which the holotype of the type species (*E. megacephalus*) belongs, having state 1 (Werneburg, 2007a: figs. 6, 7). So is **Acanthostomatops* (Witzmann & Schoch, 2006a).

Microphon exiguus changes from state 0 to state 1 in ontogeny (Bulanov, 2003: fig. 16, 19; 2014); only the "juvenile" stage of *M. gracilis* is known, so we cannot take its state 0 at face value. We have therefore assigned state 1 to *Microphon*. Similarly, only the "juvenile" stage of *Leptoropha* is known, so we have scored it as unknown as well even though it shows the originally scored state 0.

We have scored *Tseajaia* as unknown, because the state depends on the unclear position of the suture between postparietal and tabular. Moss (1972) located this suture lateral enough for state 1 to result, Berman, Sumida & Lombard (1992) preferred a more medial position that would cause state 0, and Reisz (2007: 245) simply said "the size of the tabular is uncertain in *Tseajaia*".

It seems fair to assign state 1 to **Karpinskiosaurus*, although it is arguably borderline (Klembara, 2011).

Of the **Cheliderpeton* specimens figured by Werneburg & Steyer (2002), the smallest and the largest have arguably borderline cases of state 1, while at least two others (fig. 1b, 6) clearly have state 0; we have scored **Cheliderpeton* as polymorphic.

We have scored **Konzhukovia* as having state 1, but it is arguably borderline (Gubin, 1991).

**Australerpeton* is polymorphic (Eltink et al., 2016: fig. 2).

44. POSPAR 10: **Nasals not smaller (0) or smaller (1) than postparietals.**

State 0 is present in *Ventastega* (Ahlberg et al., 2008).

Judging from their own figures, Danto, Witzmann & Müller (2012) miscoded this character in *Solenodonsaurus*, which clearly has state 0 (as scored by RC07), not 1: the nasals have several times the area of the postparietals.

State 1 occurs in *Brachydectes* (Pardo & Anderson, 2016: fig. 2E, 4B).

45. POSFRO 1: **Separately ossified postfrontal: present (0); absent (1).**

In the aïstopods, a single bone occupies the area where the postorbital and the postfrontal would be expected. (The separation in *Lethiscus* identified by Wellstead, 1982, is a break: Pardo et al., 2017; J. Pardo, pers. comm.) This bone has been variously identified as one or the other in the literature; there seems to be no evidence on which to base a decision, and the ontogeny of *Phlegethontia* suggests that the bone is a fusion product of postorbital and postfrontal (Anderson, Carroll & Rowe, 2003: fig. 9b). We have arbitrarily followed the latest publication (Pardo et al., 2017) and, by analogy, the tradition for **dinosaurs in scoring the postfrontal as absent and the postorbital (POSORB 1, ch. 61 below) as present; however, we have also scored several POSORB characters as unknown for all aïstopods under the assumption that the dorsal ossification center of *Phlegethontia* may be the postfrontal. Reexamination of ***Ophiderpeton* could help to test this approach. – We have scored **Pseudophlegethontia* as unknown for all POSFRO and POSORB characters; see ch. 31 (PAR 1) above.

State 0 is present in *Ventastega* (Ahlberg et al., 2008) and *Bruktererpeton* (Boy & Bandel, 1973).

46. POSFRO 4: **Postfrontal posterior margin lying flush with jugal posterior margin: no (0); yes (1).** Most or all cases of state 1 are only approximate, making it difficult to decide where exactly state 0 should begin. We have changed as few scores as possible, except for scoring all aïstopods as unknown (see immediately above).

Ventastega (Ahlberg et al., 2008) and *Westlothiana* (Smithson et al., 1994) have state 0.

Orobates shows state 1 (Berman et al., 2004), as does *Microphon* (Bulanov, 2003).

**Cheliderpeton* passes from state 0 to state 1 in ontogeny (Werneburg & Steyer, 2002: fig. 1), so we have scored the latter.

Nonetheless, **Glanochthon* is polymorphic (Schoch & Witzmann, 2009b: fig. 2), and so is **Australerpeton* (Eltink et al., 2016: fig. 2).

47. INTEMP 2: **Intertemporal not interdigitating (0) or interdigitating (1) with cheek.** “There appears to be no signal associated with the derived state of this character” according to RC07. Yet, even for the full taxon sample (Analysis R4), this character has only six steps. State 1 is an autapomorphy of Whatcheeriidae, Temnospondyli and/or *Caerorhachis*, possibly homologous among these groups (unambiguously so in MPTs where *Ossinodus* is not a whatcheeriid); of (*Pholiderpeton attheyi* + *Anthracosaurus* + *NSM 994 GF 1.1 + **Palaeoherpeton* + **Neopteroplax*); and of (*Seymouria* (*Kotlassia*, **Karpinskiosaurus*)) – the only unambiguous reversal in all MPTs occurs in *Capetus*. Thus, even though the present character is unknown or inapplicable in most OTUs, it clearly displays phylogenetic signal.

State 0 is present in *Ventastega* (Ahlberg et al., 2008).

Trimerorhachis has state 1 (Milner & Schoch, 2013); so does **Saharastega* (D. M., pers. obs. of MNN MOR 73).

We have scored *Ossinodus* as having state 1 because the suture between skull table and cheek is not smooth and because a separate intertemporal is fairly likely present (Warren, 2007). Of ch. 32 of this matrix (PAR 2/POSFRO 3/INTEMP 1/SUTEMP 1), we have assigned state 0 (intertemporal present) or 2 (parietal-postorbital contact, supratemporal present) to it.

Although the expression is very weak, we have kept state 1 for *Cochleosaurus* (Sequeira, 2004) and assigned it to **Karpinskiosaurus* (Klembara, 2011).

48. INTEMP 3: **Intertemporal/squamosal contact: absent (0); present (1).** RC07 called it a suture, but explained it as a contact in the next sentence; we have therefore scored any contact between these two bones as state 1, without considering whether it counts as a suture.

State 1 is thus present in *Ventastega* (Ahlberg et al., 2008), *Crassigyrinus* (Clack, 1998: fig. 4), apparently *Eoherpeton* (Smithson, 1985: fig. 7; tentatively accepted in the reconstruction, fig. 8) and *Gephyrostegus* (Klembara et al., 2014).

State 0 is found in *Panderichthys* (Vorobyeva & Schultze, 1991).

As for the preceding character, we have scored *Ossinodus* as showing state 1 in case there is a suture separating the possible intertemporal from the postorbital (Warren, 2007).

49. INTEMP 4: **Intertemporal not (0) much smaller than supratemporal in area and about as wide as long (1).** The original name was: "Intertemporal shaped like a small, subquadrangular bone, less than half as broad as the supratemporal: absent (0); present (1)"; but under this definition, state 1 is limited to the right side of the largest *Eucritta* specimen (Clack, 2001: fig. 6), making the character parsimony-uninformative. RC07 had scored state 1 also for *Baphetes*, *Isodectes*, *Trimerorhachis* and *Balanerpeton*, but both *B. kirkbyi* (Beaumont, 1977: fig. 21) and *B. orientalis* (Milner, Milner & Walsh, 2009: fig. 5) have state 0 by the width criterion, as do *Isodectes* (by width – the shape is somewhat arguable: Sequeira, 1998), *Trimerorhachis* (both by shape and by width: Milner & Schoch, 2013) and *Balanerpeton* (by width: Milner & Sequeira, 1994). State 0 is further present in *Ventastega* (by shape and almost certainly width: Ahlberg et al., 2008); as for the preceding two characters, we have scored *Ossinodus* as showing state 0 in case there is a suture separating the possible intertemporal from the postorbital (Warren, 2007).

Rather than deleting this character, however, we turned to the explanation of this character by RC07 (p. 96), which speaks of "a diminutive intertemporal" and ends in: "The ornamented surface of the intertemporal is approximately square and can be 'contained' within the supratemporal in the derived condition [= state 1] of this character." Indeed, intertemporals seem to occur in two size classes. If we replaced "broad" by "long" in the original name, state 1 would occur in some individuals of *Isodectes* and nowhere else; specifying a square shape would restrict state 1 to *Baphetes orientalis* (not *B. kirkbyi*, where the intertemporal is pentagonal) and again the right side of the largest *Eucritta* specimen; the present formulation, however, justifies the original scores (*Trimerorhachis* may be a borderline case, but we have kept it), except for making Dendrerpetidae polymorphic (A. R. Milner, 1980; Godfrey, Fiorillo & Carroll, 1987; Holmes, Carroll & Reisz, 1998).

Utegenia seems to change from state 1 to state 0 in ontogeny (Klembara & Ruta, 2004a); we have kept state 0.

50. SUTEMP 2: **Rostral border of temporal embayment formed only by squamosal (0) or at least in part by supratemporal (1).** Except for the clarifying addition of "at least in part", this wording is equivalent to the description of this character by Ruta, Coates & Quicke

(2003). RC07 changed that to: "Supratemporal forming entire edge of dorsalmost part (in lateral aspect) of temporal notch: no (0); yes (1)." When we use this wording, however, we cannot replicate the coding of this character, because we do not know where to draw the line between the "notch" and the rest of the "embayment" that RC07 mention in the explanation of this character. (No explanation was given in the 2003 version.) – We here use "tympanic/otic/temporal/spiracular notch/embayment" as synonyms regardless of inferred functions.

Contrary to the coding by RC07, this character is inapplicable when there is no embayment (SQU 3(0), see below). It is furthermore not applicable to *Acanthostega*, because the entire edge of its temporal embayment is formed by the tabular, or to taxa with a squamosal/tabular suture (the ones to which TAB 5 is applicable).

State 1 is present in *Ventastega* (Ahlberg et al., 2008), *Crassigyrinus* (Clack, 1998), *Whatcheeria* (Lombard & Bolt, 1995) and *Silvanerpeton* (Ruta & Clack, 2006: fig. 3A).

Diadectes is polymorphic (North American species: 0 [Berman, Sumida & Lombard, 1992]; *D. absitus*: 1 [Berman, Sumida & Martens, 1998]).

This character is difficult to apply to **Saharastega* (D. M., pers. obs. of MNN MOR 73); we have scored it as unknown.

Romer (1963) quite unambiguously showed state 1 on the preserved left side in the drawing of the specimen of **Neopteroplax* (fig. 1). The reconstruction (fig. 3, 4), however, the left side has a tabular/squamosal suture, and the right side – which is not preserved – has state 1. The text does not mention this question, except for stating (p. 423): "Laterally, intertemporal and supratemporal curve smoothly downward from the plane of the skull table." Encouraged by this sentence, which implies state 1 assuming that there is no squamosal/tabular suture, we have accepted the specimen drawing at face value and scored state 1.

**Australerpeton* is polymorphic, with both states apparently occurring in the two largest specimens (Eltink et al, 2016: fig. 2).

51. SUTEMP 3: **Supratemporal narrow and strap-like, at least three times as long as wide: absent (0); present (1).**

The text and the skull reconstruction by Smithson et al. (1994) ascribe state 0 to *Westlothiana* (making it similar to diadectomorphs and basal amniotes), and RC07 accepted this. However, based on the specimen drawing (Smithson et al., 1994: fig. 5B), we strongly suspect that the occipital bone plate in question belongs to the squamosal rather than to the supratemporal. If this is correct, the supratemporal is within the range of state 1 (which is otherwise limited to urocordylids, aïstopods, and the diadectomorph *Orobates*). We have therefore changed the score of *Westlothiana* to 1, and hope that the ongoing redescription of *Westlothiana* (M. Ruta, pers. comm. 2015; Clack & Milner, 2015) will clarify the issue. It is highly unfortunate that the published specimens were split through the bone, so that one slab contains the dorsal side of the skull roof in ventral view and the other side holds the ventral side of the skull roof in dorsal view.

Lethiscus has state 0, which we have scored, unless (and then only borderline) if measured along the curve of its long axis (Pardo et al., 2017).

52. SUTEMP 4: **Supratemporal/squamosal suture: smooth (0); interdigitating (1).** According to RC07, "no clear signal is associated with the distribution of the derived state". At 20 steps for 158 OTUs (Analysis R4), the state distribution of this character is indeed not very tidy; nonetheless, state 1 is synapomorphic of *Whatcheeria* and *Pederpes*, of Temnospondyli (reversed in **Deltaherpeton* and at least six times among traditional temnospondyls), of *Seymouria*, *Kotlassia* and **Karpinskiosaurus* and also of **Coloraderpeton* and **Pseudophlegethontia* (unknown in other aïstopods except *Oestocephalus*). Clearly, this character is not useless.

State 0 is present in *Ventastega* (Ahlberg et al., 2008) and *Acanthostega* (Porro, Rayfield & Clack, 2015). We have also assigned it to Dendrerpetidae, where the suture is not straight (especially in lateral view) but still smooth (A. R. Milner, 1980, 1996; Holmes, Carroll & Reisz, 1998).

State 1 is known in *Acheloma* (Polley & Reisz, 2011) and in one middle-sized specimen of **Acanthostomatops* (Witzmann & Schoch, 2006a: fig. 2C), while the others have state 0. We have also scored state 1 in **Nigerpeton*; the suture is difficult to find and to confirm, but it is clearly not a straight line (D. M., pers. obs. of MNN MOR 70).

Unknown in *Lethiscus* (Pardo et al., 2017: ext. data fig. 2, 3).

**Spathicephalus* has state 1 (Smithson et al., 2017: fig. 3C). So does **Pseudophlegethontia* (on the left side; see ch. 31, PAR 1).

**Glanochthon* is polymorphic (Schoch & Witzmann, 2009b: fig. 2).

We have assigned state 1 to **Platyoposaurus*, though it is a somewhat borderline case (Gubin, 1991: drawing 3). Similarly, although weak, interdigitation is present in **Pholidogaster* (Panchen, 1975: fig. 14) and **Australerpeton* (Barberena, 1998: fig. 3).

53. TAB 1/SQU 4: **Separately ossified tabular: present (0); absent (1).** RC07 distinguished TAB 1, which had the present name, from SQU 4, which described the absence (0) or presence (1) of a single bone in the places normally occupied by the squamosal, the tabular, and the supratemporal. SQU 4(1) was limited to the adelogyrinids. Based on which bones are lost in other taxa in this matrix (and elsewhere among limbed vertebrates), we interpret the "squamosotabular bone" (Andrews & Carroll, 1991) as simply the squamosal, so that the adelogyrinids lack tabulars and SQU 4 turns out to be a duplicate of TAB 1. We do, however, identify potential homologues of the "tabular horns" in *Adelospondylus*, which we have scored TAB 2(1) and TAB 6(1), and *Adelogyrinus*, which we have scored TAB 6(1) (TAB 2 being unknown), both after the drawings by Andrews & Carroll (1991).

Ventastega shows state 0 (Ahlberg et al., 2008).

State 1 is present in Albanerpetidae (McGowan, 2002).

State 0, tentatively reconstructed by Jenkins, Walsh & Carroll (2007), appears likely for *Eocaecilia*: what appears to be the tabular or supratemporal is caudally broken on the left side of the crushed type specimen (MNA V8066, formerly MCZ 9010), but appears to have reached the caudal edge of the skull table on the right side (D. M., pers. obs.).

54. TAB 2-3-9: **"Ventral tabular horn" (caudal process of tabular lying ventral to tabular ornamented surface): absent (0); pointed (1); button-like (2); rectangular (3) (unordered).** No sequence for ordering is apparent: the longest rectangles are as long as the longest buttons, while the shortest rectangles are extremely short; the widest buttons are no wider than the widest horns, and the widest horns are at least as wide as the widest rectangles, while the narrowest horns are extremely narrow.

RC07 distinguished three characters: TAB 2, the presence or absence of a "[b]lade-like […] horn"; TAB 3, the presence or absence of a "[r]ounded, button-like posterior process"; and TAB 9, the presence or absence of a "[d]orsoventrally flattened, posteriorly directed, subhorizontal outgrowth" from the ventral rather than the caudal surface of the tabular. Tellingly, these characters had almost completely mutually exclusive distributions: TAB 2(1) was assigned to *Acanthostega*, *Crassigyrinus*, *Whatcheeria*, *Caerorhachis*, all anthracosaurs, *Gephyrostegus*, *Silvanerpeton* and *Utegenia*; TAB 3(1) was scored for *Greererpeton*, *Baphetes*, *Megalocephalus*, *Edops*, "*Dendrerpeton*" and *Pederpes*; TAB 9(1) was ascribed to *Discosauriscus*, *Ariekanerpeton*, *Microphon* and *Utegenia*. Most of these OTUs were scored 0, rather than "unknown", for the other two characters, which means that

RC07 treated these three characters as describing three different processes that were not homologous to each other.

From comparison across the taxon sample, it is obvious that all three processes are primary homologues of each other; indeed, state TAB 3(1) becomes state TAB 2(1) in the ontogeny of *Apateon pedestris* (compare fig. 7 of Boy & Sues, 2000, to fig. 3D of Schoch & Milner, 2008). "Horns" are apparently always at or close to the lateral margin of the tabular, while "buttons" are usually near the medial margin; but buttons can be approximately in the middle as in various baphetids (Beaumont, 1977), quite close to the lateral margin (immature *Apateon pedestris*: Boy & Sues, 2000: fig. 7; *Pederpes*: Clack & Finney, 2005; **Bystrowiella*: Witzmann & Schoch, 2017: fig. 2A), and even at the lateral margin, projecting lateral to it (**Spathicephalus*: Beaumont & Smithson, 1998). "Flattened processes" stand out from the ventral side of the tabular to which they are attached (all the way to the caudal edge of the skull table); but so, in many or perhaps all cases, do "horns" (*Platyrhinops*: Clack & Milner, 2010; *Anthracosaurus*: Clack, 1987a: fig. 2; **Branchiosaurus*: Werneburg, 2012a: fig. 7) and even "buttons" (baphetoids: Beaumont, 1977, Beaumont & Smithson, 1998; *Greererpeton*: Smithson, 1982: fig. 13B).

All four states are mutually exclusive; this fact lets us merge all three of the original characters.

To their description of TAB 2, RC07 (p. 97) added: "Under the definition of a tabular 'horn', we include processes of the subdermal part of the bone, which in anthracosaurs has also a separate dermal component." This dermal component, the "dorsal tabular horn", is not limited to anthracosaurs, however; what is unique to anthracosaurs is that the dorsal and the ventral horn point in different directions (most prominently in *Proterogyrinus*, but also in *Anthracosaurus* and to a lesser degree *Pholiderpeton attheyi*: Clack, 1987a). More commonly, it lies directly on top of the ventral horn, and that this is the condition seen in the other anthracosaurs which have a single, dorsoventrally thick "horn" per side (e.g. **Neopteroplax*: Romer, 1963). *Gephyrostegus* even has a dorsal horn without a ventral horn: the horn is a dorsoventrally very thin process of the ornamented surface alone (Klembara et al., 2014). We have redefined TAB 6 to code for the presence or absence of the "dorsal tabular horn".

State 0 is present in *Solenodonsaurus* (Danto, Witzmann & Müller, 2012).

State 1 occurs in *Ventastega* (Ahlberg et al., 2008), *Ichthyostega* (Clack & Milner, 2015: fig. 8), *Eryops* (Sawin, 1941), *Acheloma* (Polley & Reisz, 2011), most likely *Broiliellus* (Carroll, 1964: fig. 9), *Eoscopus* (Daly, 1994), *Platyrhinops* (Clack & Milner, 2010; Werneburg, 2012a), *Apateon* (Schoch & Milner, 2008) and *Leptorophus* (Boy, 1986, 1987; Schoch, 2014a) as well as apparently *Chenoprosopus* (Hook, 1993), *Westlothiana* (Smithson et al., 1994: fig. 5A) and *Adelospondylus* (Andrews & Carroll, 1991; see TAB 1/SQU 4). Further, **Nigerpeton* has state 1 (D. M., pers. obs. of MNN MOR 69 and MNN MOR 70); the "horn" is merely curved ventrally and therefore only shown as a stippled line in Steyer et al. (2006: fig. 2A).

State 2 is found in *Trimerorhachis* ("only a very rudimentary horn well set off from the sculptured part of the bone": Milner & Schoch, 2013: 99), likely *Phonerpeton* (Dilkes, 1990), and *Limnoscelis* ("a distinct, low, dome-like swelling of unknown function": Berman, Reisz & Scott, 2010: 196, fig. 3, 4).

Kotlassia (Bulanov, 2003: fig. 30) and *Seymouria* (Laurin, 1996a, 2000; Klembara et al., 2007) have state 3, even though the rectangle becomes extremely short in (adult) *S. baylorensis* and *Kotlassia*.

Unknown in *Balanerpeton* (Milner & Sequeira, 1994), Dendrerpetidae (A. R. Milner, 1980, 1996; Holmes, Carroll & Reisz, 1998) and *Capetus* (Sequeira & Milner, 1993).

The tabulars of *Brachydectes* are so modified that this character is hard to apply (Pardo & Anderson, 2016); we have scored it as unknown.

Best scored as unknown in **Saharastega* due to damage and problems of applicability (D. M., pers. obs. of MNN MOR 73).

The condition of **Karpinskiosaurus* is intermediate between state 1 and 2 (Klembara, 2011: fig. 3C); we have scored partial uncertainty.

In **Sclerocephalus*, the ornamented surface possibly grows over the ventral horn in large specimens, but this is not the case in the specimen illustrated by Schoch & Witzmann (2009a: fig. 5A) which clearly shows state 1. Schoch (2009a), however, stated that state 0 occurs in the "terrestrial" morph and state 1 is restricted to the aquatic morph; we have accepted this and have scored **Sclerocephalus* as polymorphic, but caution that preservation may cause false appearances of state 0 when skulls are only exposed in dorsal view.

deleted TAB 4: **Tabular/squamosal suture extending onto skull table dorsal surface: present (0); absent (1).** This character was scored (usually 1) for almost all taxa in RC07, yet almost none possess a tabular/squamosal suture. Because it is further influenced by presence and position of the supratemporal and the temporal embayment, it would be inapplicable to most of the taxon sample if taken literally. It could be reinterpreted as the presence or absence of participation of the squamosal in the dorsal surface of the skull roof, but that is already PAR 7 (ch. 36). We have therefore deleted this character. As it was scored by RC07, it required the same number of steps under the TH and the LH.

55. TAB 5: **Tabular/squamosal suture: smooth (0); interdigitating (1).** This character is inapplicable in the absence of contact between the tabular and the squamosal, notably in *Eusthenopteron* and *Panderichthys*, which were originally scored 0.

Broiliellus has state 0 (Carroll, 1964: fig. 9).

Batropetes is polymorphic (Glienke, 2013, 2015).

State 1 occurs in *Brachydectes* (Pardo & Anderson, 2016: fig. 3A, C).

**Micropholis* shows state 1 when the suture is present and long enough to tell (Schoch & Rubidge, 2005: fig. 1C, 3; not the oversimplified fig. 2C, which shows the same specimen as fig. 1C).

In *Ecolsonia* (Berman, Reisz & Eberth, 1985), **Acanthostomatops* (Witzmann & Schoch, 2006a) and **Erpetosaurus* (Milner & Sequeira, 2011), the suture is probably too short to tell.

56. TAB 6: **"Dorsal tabular horn": absent (0); present (1).**

The original wording was: "Tabular (including its ornamented surface) elongate posterolaterally or posteriorly in the form of a massive, horn-like process, conferring a boomerang-like shape to skull outline in plan view: absent (0); present (1)". (Evidently "plan" means dorsal and ventral.) State 1 was exclusively limited to *Diplocaulus* and *Diploceraspis*. To make this character more useful, we have excluded the size of the tabulars in this character (very large ones are necessary to give a boomerang shape to the entire skull instead of just the skull table) and compromised on "massive", interpreting this character as the presence or absence of tabular "horns" composed of the ornamented surface (different from TAB 2-3-9) that extend markedly caudal to the postparietals. Thus, *Baphetes* (Beaumont, 1977: fig. 18, 20), *Megalocephalus* (Beaumont, 1977: fig. 10(*b*), 11(*a*)), *Platyrhinops* (Clack & Milner, 2010), *Micromelerpeton* (Schoch, 2009), **Micropholis* (Schoch & Rubidge, 2005: fig. 3) and **Cheliderpeton* (Werneburg & Steyer, 2002) are polymorphic, while state 1 alone is documented in *Ventastega* (Ahlberg et al., 2008), *Acanthostega* (Clack, 2003), *Chenoprosopus* (Hook, 1993; Reisz, Berman & Henrici, 2005), *Cochleosaurus* (Sequeira, 2004), *Trimerorhachis* (Milner & Schoch, 2013), *Balanerpeton* (at least sometimes: Milner & Sequeira, 1994: fig. 2, 4, 5), Dendrerpetidae (A. R. Milner, 1980, 1996; Holmes, Carroll & Reisz, 1998),

Eryops (both morphotypes: Werneburg, 2007b), *Acheloma* (Maddin, Reisz & Anderson, 2010; Polley & Reisz, 2011), *Broiliellus* (Carroll, 1964), tentatively *Amphibamus* (Schoch & Milner, 2014: fig. 30A; possibly Daly, 1994: fig. 18), *Eoscopus* (Daly, 1994), *Apateon* and *Leptorophus* (Schoch & Milner, 2008), *Eoherpeton* (Panchen, 1975; Smithson, 1985), *Proterogyrinus* (Holmes, 1984), *Archeria* (Holmes, 1989), both species of *Pholiderpeton* (Panchen, 1972; Clack, 1987b), *Anthracosaurus* (Clack, 1987a), *Bruktererpeton* (Boy & Bandel, 1973), *Gephyrostegus* (Klembara et al., 2014), *Discosauriscus* and *Seymouria* (Laurin, 1996a, 2000; Klembara, 1997), apparently *Westlothiana* (Smithson et al., 1994: fig. 5B), *Adelospondylus* and *Adelogyrinus* (Andrews & Carroll, 1991; see TAB 1/SQU 4), *Sauropleura* (all three species illustrated in Bossy & Milner, 1998: fig. 53), *Lethiscus* (Pardo et al., 2017), *Capetus* (Sequeira & Milner, 1993), *Silvanerpeton* (Ruta & Clack, 2006: especially fig. 5), **Chroniosaurus* (Clack & Klembara, 2009), **Iberospondylus* (Laurin & Soler-Gijón, 2006), **Karpinskiosaurus* (Klembara, 2011), *NSM 994 GF 1.1 (Holmes & Carroll, 2010), **Spathicephalus* (Beaumont & Smithson, 1998), **Sclerocephalus* (Schoch & Witzmann, 2009a), **Glanochthon* (Schoch & Witzmann, 2009b), **Archegosaurus* (Witzmann, 2006), **Platyoposaurus* (Gubin, 1991), **Konzhukovia* (Gubin, 1991), **Lydekkerina* (Shishkin, Rubidge & Kitching, 1996; Jeannot, Damiani & Rubidge, 2006; Hewison, 2007), **Acanthostomatops* (Witzmann & Schoch, 2006a), **Erpetosaurus* (Milner & Sequeira, 2011), **Mordex* and **Branchiosaurus* (Werneburg, 2012a), **Palaeoherpeton* (Panchen, 1964), **Neopteroplax* (Romer, 1963) and **Australerpeton* (Barberena, 1998; Eltink et al., 2016).

Unknown in *Urocordylus* (Bossy & Milner, 1998: fig. 53); not preserved in **Nigerpeton* (D. M., pers. obs. of MNN MOR 69 and MNN MOR 70); damaged in MNN MOR 73 (D. M., pers. obs.), therefore unknown in **Saharastega*.

The tabulars of *Brachydectes* are so modified that this character is hard to apply (Pardo & Anderson, 2016); we have scored it as unknown.

57. TAB 7: **Parietal-parietal width smaller than (0) or greater than (1) distance between skull table posterior margin and orbit posterior margin, measured along skull midline.** We interpreted the width to mean the maximum width between the lateral margins of the parietals.

State 0 is found in *Ventastega* (Ahlberg et al., 2008), *Kotlassia* (just barely; Bulanov, 2003: fig. 30) and *Brachydectes* (barely: Pardo & Anderson, 2016: fig. 4B).

State 1 occurs in *Broiliellus* (Carroll, 1964), *Gephyrostegus* (Klembara et al., 2014) and *Scincosaurus* (Milner & Ruta, 2009).

**Saharastega* is scored 1 because it has state 1 for the left orbit and exact equality between the distances for the right orbit.

Probably subequal in *NSM 994 GF 1.1, therefore scored as unknown.

Unclear in *Hapsidopareion* due to disarticulation (CG78: fig. 13A); unclear and likely borderline in **Neopteroplax* (Romer, 1963: fig. 1, 3).

58. TAB 8: **Tabular without (0) or with (1) posteroventrally sloping occipital exposure.** See POSPAR 7 for the meaning of "sloping".

State 0 is present in *Ventastega* (Ahlberg et al., 2008), *Rhynchonkos* (Szostakiwskyj, Pardo & Anderson, 2015) and *Lethiscus* (Pardo et al., 2017).

State 1 occurs in *Oestocephalus* (Carroll, 1998a; Anderson, 2003a), *Orobates* (Berman et al., 2004) and *Tseajaia* (Moss, 1972; Berman, Sumida & Lombard, 1992), and probably in *Hapsidopareion* (CG78: fig. 13A).

Diadectes is polymorphic (Berman, Sumida & Lombard, 1992; Berman, Sumida & Martens, 1998).

Unknown for *Eocaecilia* (where it is not clear if the tabular had any occipital exposure; Jenkins, Walsh & Carroll, 2007) and *Westlothiana* (Smithson et al., 1994). The tabulars of *Brachydectes* are so modified that this character is hard to apply (Pardo & Anderson, 2016); we have scored it as unknown as well.

59. TAB 10: **Tabulars entirely on occipital surface: no (0); yes (1).**

State 0 is present in *Ventastega* (Ahlberg et al., 2008) and *Limnoscelis* (Berman et al., 2010).

60. POSORB 1: **Separately ossified postorbital: present (0); absent (1).**

State 0 is present in *Ventastega* (Ahlberg et al., 2008).

State 1 is documented in Albanerpetidae (McGowan, 2002, Venczel & Gardner, 2005).

Adelospondylus was scored 1 in RC07. Ruta, Coates & Quicke (2003: 311) cited Andrews & Carroll (1991) as their only source for this score. However, Andrews & Carroll (1991: 364, fig. 13A, B) stated that whether a small postorbital was present or absent is unknown. Carroll & Andrews (1998: 151) considered the postorbital "small or absent in *Adelospondylus*", and also noted that the adelogyrinid postorbital is in the position where the intertemporal would be expected, complicating this issue further. Not having seen the specimen, we have changed the score to unknown for the time being.

61. POSORB 2: **Postorbital without (0) or with (1) ventrolateral digitiform process fitting into deep, vertical groove along jugal lateral surface.**

State 0 is present in *Ventastega* (Ahlberg et al., 2008) and, given the assumption explained under ch. 45 (POSFRO 1), *Oestocephalus* (Anderson, 2003: fig. 2) and *Phlegethontia* (Anderson, 2002).

State 1 is found in *Eryops* (Sawin, 1941) and more weakly in *Rhynchonkos* (Szostakiwskyj, Pardo & Anderson, 2015).

Cochleosaurus (Sequeira, 2004) and *Platyrhinops* (Clack & Milner, 2010) are polymorphic; so is **Acanthostomatops* (Witzmann & Schoch, 2006a).

Unknown in *Westlothiana* (Smithson et al., 1994).

State 1 is documented in one specimen of **Glanochthon* (Schoch & Witzmann, 2009b: fig. 2J); but because this specimen is juvenile and all other illustrated specimens show state 0, we have decided to ignore it and score state 0 for **Glanochthon*.

62. POSORB 3: **Postorbital contributing to (0) or excluded from (1) orbit margin.**

State 0 is present in *Ventastega* (Ahlberg et al., 2008) and, given the assumption explained under ch. 45 (POSFRO 1), *Oestocephalus* (Anderson, 2003: fig. 2) and *Phlegethontia* (Anderson, 2002).

Acherontiscus was scored 1 in RC07. Ruta, Coates & Quicke (2003: 311) cited Carroll (1969a) as their only source for this score; however, Carroll (1969a) did not mention this question in the text, presented a reconstruction drawing where the postorbital does contribute substantially to the orbit margin, and included a specimen drawing that can be interpreted either way, depending on which faint lines are sutures and which are breaks. Carroll (1998c) did not show drawings of the skull, but stated: "In contrast with adelogyrinids, the postorbital appears to enter the margin of the orbit." Not having seen the specimen, we have changed the score to unknown for the time being.

63. POSORB 4: **Postorbital irregularly polygonal (0) or broadly crescentic and narrowing to a posterior point (1).** We have adopted a very broad interpretation of "broadly

crescentic” and concentrated on absence and presence of the pointed caudal end; RC07 emphasized that the postorbital “terminates in an acute posterior extremity” in state 1. Very likely, however, many more states – or characters – should be distinguished to represent the diversity of postorbital shapes that occur in the OTUs of this matrix.

Taking the drawings by Clack & Milner (2015: fig. 8A, C) at face value, we have scored polymorphism for *Ichthyostega*.

Ventastega (Ahlberg et al., 2008) and *Platyrhinops* (Clack & Milner, 2010) display state 0. We have kept state 0 for *Baphetes*: although the juvenile *B. orientalis* described by Milner, Milner & Walsh has a very clear case of state 1, the adult (Beaumont, 1977: fig. 25) is borderline, and in *B. kirkbyi* the caudal suture with the supratemporal is almost perfectly transverse (Beaumont, 1977: fig. 18, 21).

Megalocephalus has rather polygonal postorbitals as well, but a clear caudal point is there, the suture with the supratemporal being oblique; we have therefore scored state 1 (Beaumont, 1977). *Trimerorhachis* has very long postorbitals with a very clear caudal point (Milner & Schoch, 2013). State 1 is also found in *Chenoprosopus* (Reisz, Berman & Henrici, 2005), in **Saharastega* (D. M., pers. obs. of MNN MOR 73), and, borderline, in *Rhynchonkos* (Szostakiwskyj, Pardo & Anderson, 2015: fig. 1); note that fig. 63 of CG78 is mirrored.

Kotlassia was scored 1 in RC07, but its caudal point is far ventral (Bulanov, 2003: fig. 30), instead of dorsal as usual; we prefer to treat this unique condition as state 0.

Seymouria was scored 1 in RC07, but the caudal end of its postorbital is a straight vertical suture (Laurin, 2000: fig. 1).

State 1 is present in *Paleothyris* (Carroll, 1969b), *Petrolacosaurus* (though this may be due to the temporal fenestrae; Reisz, 1981), and *Westlothiana* (at least on the right side, and at least in the specimen drawings as opposed to the reconstruction; Smithson et al., 1994).

Batropetes fritschi and its sister-group *B. niederkirchensis* have state 0 (Glienke, 2013), but *B. palatinus* has state 1 (Glienke, 2015), making *Batropetes* polymorphic. **Micropholis* is polymorphic as well (Schoch & Rubidge, 2005: fig. 3).

Unknown in *Hapsidopareion*, where the temporal embayment is so large that it constrains the shape of the postorbital, and *Lethiscus* (see ch. 45 – POSFRO 1 – and Anderson, Carroll & Rowe, 2003: fig. 9B).

We have assigned state 0 to **Spathicephalus* because the postorbital is only crescentic insofar as it parallels the orbit margin and because it lacks a caudal point.

**Platyoposaurus* seems to be polymorphic, judging from the drawings by Efremov (1932), Konzhukova (1955) and Gubin (1991). So is **Lydekkerina* (Jeannot, Damiani & Rubidge, 2006).

**Erpetosaurus* has a clear caudal point, but the extremely long postorbitals are lozenge-shaped, being much wider in the middle than at the orbit margin; we have assigned state 0, even though this polygon is not irregular.

64. POSORB 5: **Postorbital/tabular suture: absent (0); present (1).** Unlike RC07, we have scored this character as unknown (inapplicable) when the supratemporal is present. This affects all OTUs scored 0 in RC07 except *Hyloplesion*, the diplocaulids and *Hapsidopareion*; we have scored the latter as unknown as well, however, because its temporal embayment is unusually large and acts like a supratemporal for the purpose of this character.

Microbrachis has state 1 (Olori, 2015).

65. POSORB 6: **Postorbital not wider (0) or wider (1) than orbit.** Judging from the original scores, this is meant to be measured in strict dorsal view.

State 0 occurs in *Ventastega* (Ahlberg et al., 2008). We have kept state 0 for *Limnoscelis*, which is borderline (Berman, Reisz & Scott, 2010: fig. 3A).

State 1 occurs in *Ichthyostega* (Clack & Milner, 2015: fig. 8) and *Cochleosaurus* (Sequeira, 2004).

Unknown in *Lethiscus* (see ch. 45 – POSFRO 1 – and Anderson, Carroll & Rowe, 2003: fig. 9B); *Phlegethontia*, however, has state 0 regardless of how much of the apparently compound bone consists of the postorbital (Anderson, 2002).

The condition is so borderline in the reconstruction of **Nigerpeton* (Steyer et al., 2006) and the specimen drawings of **Lydekkerina* (Jeannot, Damiani & Rubidge, 2006) that we have scored both as unknown. Pers. obs. of **Nigerpeton* (by D. M.) failed to clarify its condition: the orbit margin is only preserved on two separate pieces of MNN MOR 70.

66. POSORB 7: **Postorbital at least one-fourth of the width of the skull table at the same transverse level: absent (0); present (1).** RC07 used “skull roof” instead of “skull table”. Again, this character is evidently meant to be measured in strict dorsal view.

State 0 occurs in *Ventastega* (Ahlberg et al., 2008), *Colosteus* (Hook, 1983) and *Silvanerpeton* (Ruta & Clack, 2006) as well as **Nigerpeton* (D. M., pers. obs. of MNN MOR 70).

State 1 occurs in *Ichthyostega* (Clack & Milner, 2015: fig. 8), *Baphetes* and *Megalocephalus* (Beaumont, 1977), *Eucritta* (Clack 2001), *Edops* (Romer & Witter, 1942; D. M., pers. obs. of MCZ 1378), *Cochleosaurus* (Sequeira, 2004), *Neldasaurus* (Chase, 1965), *Trimerorhachis* (Milner & Schoch, 2013), *Eryops* (Sawin, 1941), *Amphibamus* (Schoch, 2001), *Doleserpeton* (Sigurdsen & Bolt, 2010), *Eoscopus* (Daly, 1994), *Kotlassia* (Bulanov, 2003: fig. 30), *Scincosaurus* (Milner & Ruta, 2009), all diplocaulids (A. C. Milner, 1980), and **Chroniosaurus* (Clack & Klembara, 2009).

Apateon passes from state 0 to state 1 during ontogeny (Schoch & Fröbisch, 2006). Still, *Leptorophus* retains state 0 as scored by RC07 (Boy, 1986), so we have kept the score of 0 for *Schoenfelderpeton*.

Unknown in *Adelogyrinus* (Andrews & Carroll, 1991) and *Lethiscus* (see ch. 45 – POSFRO 1 – and Pardo et al., 2017).

Microphon is polymorphic (Bulanov, 2003: fig. 16, 22), though we caution that this difference between *M. exiguus* (state 1) and *M. gracilis* (state 0) could be ontogenetic, with the known skull of *M. gracilis* being closer to maturity than that of *M. exiguus* (literally “the tiny one”).

**Acanthostomatops* is polymorphic, often within the same specimen, and all cases are close to the boundary between the states (Witzmann & Schoch, 2006a).

**Glanochthon* is likewise polymorphic, sometimes between the left and the right side of the same individual (Schoch & Witzmann, 2009b: fig. 2).

67. POSORB 8: **Anteriormost part of postorbital [dorso]me[d]ial margin with sigmoid profile in dorsal or lateral aspect: absent (0); present (1).** A clearer wording could be “ventrolateral digitiform process on the postfrontal: absent (0); present (1)”.

State 0 is seen in *Ventastega* (Ahlberg et al., 2008) and *Batropetes* (Glienke, 2013).

Some specimens of *Trimerorhachis* show state 1 on one side (Milner & Schoch, 2013); we have scored this as polymorphism.

Unknown in *Lethiscus* (see ch. 45 – POSFRO 1 – and Pardo et al., 2017); not preserved in **Nigerpeton* (D. M., pers. obs. of MNN MOR 70).

State 1 is documented in one specimen of **Glanochthon* (Schoch & Witzmann, 2009a: fig. 2I); but because this specimen is juvenile and all other illustrated specimens show state 0, we have decided to ignore it and score state 0 for **Glanochthon*.

On the right side of *Palaeoherpeton*, the process could be considered just another part of the interdigitation of the suture, but on the left side, state 1 is clearly present (Panchen, 1964: fig. 11, 12).

Australerpeton is polymorphic (Eltink et al., 2016: fig. 2–5).

68. SQU 1: **Rostral end of squamosal lying posterior to (0) or anterior to (1) parietal midlength (measured along the midline).** RC07 phrased "rostral end" as "anterior part" without defining where the caudal end of this part might lie.

Ventastega (Ahlberg et al., 2008), *Westlothiana* (Smithson et al., 1994) and *Micraroter* and *Euryodus* (CG78) show state 0, which is also borderline present in *Rhynchonkos* (Szostakiwskyj, Pardo & Anderson, 2015).

State 1 occurs in *Eucritta* (Clack, 2001), *Valdotriton* (Evans & Milner, 1996), *Batropetes* (Glienke, 2013) and *Cardiocephalus* (CG78) as well as *Adelospondylus* and *Adelogyrinus* (Andrews & Carroll, 1991).

Trimerorhachis is usually borderline; there are a few clear cases (at least on one side of certain skulls) of state 1 and a few less clear ones of state 0 (Milner & Schoch, 2013). We have scored polymorphism.

The specimen of *Archegosaurus* illustrated by Witzmann (2006: fig. 5) has state 1 when the right parietal is used for measurement and is borderline when the left one is chosen; we have scored state 1.

In *Konzhukovia* the rostral ends of the squamosals are at parietal midlength (Gubin, 1991); we have scored it as unknown.

Erpetosaurus is polymorphic and often borderline (Milner & Sequeira, 2011), much like *Trimerorhachis*.

Branchiosaurus starts out with state 1, but apparently reaches state 0 in ontogeny (Werneburg, 2012a); we have scored state 0.

SQU 2 is merged with JAW ART 1 and DEN 8, see below.

69. SQU 3: **Caudolateral edge of skull: straight or convex (0); dorsoventrally tall embayment (1); dorsally restricted notch (2) (unordered).** The original name and description of this character contradict each other: "*Squamosal without (0) or with (1) broad, concave embayment.* An embayment is widespread among early tetrapods, a deeply incised squamosal notch is seen only in some stem amniotes and, conspicuously, in temnospondyls and salientians." [italics in the original] Judging from its name, the "deeply incised squamosal notch" (found, incidentally, in few temnospondyls and no salientians!) was lumped with the complete absence of an embayment into the same state – and indeed this was reflected in the matrix. We have not ordered this character because the temporal embayment could disappear by rostrocaudal shortening (which might pass from state 2 over 1 to 0 or directly from 2 to 0) or by dorsoventral narrowing (from 1 over 2 to 0). As the criterion for distinguishing between states 1 and 2, we have primarily used whether the embayment encroaches on the quadratojugal (state 1) or has its ventral end within the squamosal (state 2).

Within state 2, a difference between a short triangular notch (e.g. *Eoherpeton*: Smithson, 1985; *Llistrofus*: Bolt & Rieppel, 2009: fig. 4) and a narrow, deep one which has almost parallel edges for much of its length (e.g. *Seymouria*: Laurin, 1996a, 2000; *Phonerpeton*: Dilkes, 1990; D. M., pers. obs. of MCZ 2313) could be recognized, but this is already included in other characters (TAB 7, ORB 5).

The temnospondyls *Capetus* (Sequeira & Milner, 1993) and *Phonerpeton* (Dilkes, 1990: state 2: D. M., pers. obs. of AMNH 7150 [7 cm skull length]: borderline state 1; MCZ 2313 [10 cm skull length]: extreme case of state 2) appear to pass from state 1 to state 2 in its

ontogeny; so does, more clearly, the seymouriamorph **Karpinskiosaurus* (Klembara, 2011). The difference between these states is not, however, size-dependent, as demonstrated by the fact that *Phonerpeton* has state 2 while *Dendrysekos* (Dendrerpetidae), at the same skull size as *Phonerpeton*, has state 1 (Holmes, Carroll & Reisz, 1998).

State 1 further occurs in *Gephyrostegus*; although not rounded, the embayment is extremely tall (Klembara et al., 2014), quite unlike what is seen in anthracosaurs. State 1 is unambiguously present in *Ecolsonia* (Berman, Reisz & Eberth, 1985; that the tabular and the quadratojugal meet caudal to the embayment does not matter), *Solenodonsaurus* (Laurin & Reisz, 1999), *Diadectes* (Berman, Sumida & Lombard, 1992; Berman, Sumida & Martens, 1998), arguably *Batropetes* (Glienke, 2015: fig. 9D, E), *Adelogyrinus* (Andrews & Carroll, 1991), *Ariekanerpeton* and *Utegenia* (Klembara & Ruta, 2004a, 2005a), *Leptoropha* (Bulanov, 2003), *Orobates* (Berman et al., 2004), *Silvanerpeton* (Ruta & Clack, 2006), and *Tseajaia* (Moss, 1972; Berman, Sumida & Lombard, 1992; Reisz, 2007; D. M., pers. obs. of CM 38033). It is also seen in **Iberospondylus* and **Acanthostomatops*, even though the greatly enlarged quadratojugal creates the appearance of state 2 in dorsal view (Laurin & Soler-Gijón, 2006; Witzmann & Schoch, 2006a).

State 2, the plesiomorphy, is seen in *Ventastega* (Ahlberg, Lukševičs & Lebedev, 1994; Ahlberg et al., 2008), *Ichthyostega* (Clack & Milner, 2015: fig. 8), *Edops* (D. M., pers. obs. of MCZ 1378), adult *Chenoprosopus* (Langston, 1953: fig. 8), *Cochleosaurus* (Sequeira, 2004), *Trimerorhachis* (Milner & Schoch, 2013), *Balanerpeton* (somewhat arguably; Milner & Sequeira, 1994), *Eryops* (D. M., pers. obs. of casts on exhibit in various museums as well as TMM, CM, USNM and MCZ specimens; very difficult to tell from publications like Sawin, 1941), *Acheloma* (Polley & Reisz, 2011), the most mature specimens of *Micromelerpeton* (Schoch, 2009b: fig. 2b), *Apateon* (throughout ontogeny: Boy, 1987; Schoch & Fröbisch, 2006), *Leptorophus* (though borderline: Boy, 1986), *Caerorhachis* (Ruta, Milner & Coates, 2002), all anthracosaurs, *Lethiscus* (Pardo et al., 2017) and *Microphon* (Bulanov, 2003).

From the available illustrations, we cannot tell if *Kotlassia* (Bulanov, 2003: fig. 30) has state 1 or 2. We have assigned the same partial uncertainty to **Pholidogaster* (Panchen, 1975: fig. 15).

Dendrerpetidae is polymorphic, with *Dendrysekos* having state 1 (see above) but *Dendrerpeton acadianum* showing state 2 instead (Milner, 1996: fig. 6A; Schoch & Milner, 2014: fig. 16B). Unfortunately the state of *Dendrerpeton confusum* is unknown (Milner, 1996: fig. 8), and *D. rugosum* probably but not certainly has state 2 (A. R. Milner, 1980: fig. 6a, c), so we cannot reconstruct the ancestral state for Dendrerpetidae.

We have scored state 0 or 2 for *Oestocephalus*: a very small notch as seen in *Lethiscus* cannot be excluded judging from Carroll (1998a: fig. 2A, 3C).

This character is not applicable to *Phlegethontia* because so much of the dermal skull roof is lost; the caudal edge of the putative squamosal (Anderson, 2002) could be called state 1 or even 2, but is most likely unrelated, lacking e.g. a caudomedial lamina.

Despite diagenetic squishing, an unusual version of state 0 is recognizable in **Saharastega* (D. M., pers. obs. of MNN MOR 73).

We have assigned state 1 to **Liaobatrachus*, although *L. zhaoi* might be said to have state 2 instead (Dong et al., 2013: fig. 6C, 7D).

In *caseasaurs the supratemporals project caudally beyond the rest of the skull table, producing the impression of state 2 (*Oedaleops*, *Eocasea*) or possibly 1 (*Eothyris*) in lateral view. We consider this an unrelated feature and have scored state 0 for *Caseasauria.

Although the reconstruction of **Bystrowiella* (Witzmann & Schoch, 2017: fig. 15) suggests state 2, the photo and specimen drawing (fig. 3A, C) strongly suggest state 1, which we have scored.

State 2 is visible in **Coloraderpeton* (Pardo et al., 2017: video).

70. SQU 5: **Squamosal without (0) or with (1) internal shelf bracing quadrate from behind.** RC07 scored state 1 for the distinctive condition found in diplocaulids and *Scincosaurus*. Therefore, we have not considered the smaller caudal exposure of the squamosal found in amniotes, diadectomorphs and possibly *Batropetes niederkirchensis* and **Crinodon* as state 1. However, state 1 is unambiguously found in **Australerpeton*, where a medial process of the squamosal meets the pterygoid dorsal to the occipital exposure of the quadrate (Barberena, 1998; Eltink et al., 2016).

Lethiscus has state 0 (Pardo et al., 2017).

Unknown in *Bruktererpeton* (Boy & Bandel, 1973); best scored as unknown in **Saharastega* (D. M., pers. obs. of MNN MOR 73).

71. JUG 1: **Separately ossified jugal: present (0); absent (1).**

State 0 is present in *Ventastega* (Ahlberg, Lukševičs & Lebedev, 1994) and *Lethiscus* (Pardo et al., 2017).

Given the seemingly bad preservation of this area in *Triadobatrachus* (all sources), we have scored it as unknown, as RC07 had already done for the quadratojugal (QUAJUG 1).

72. JUG 2-6: **Maxilla-quadratojugal contact excluding jugal from ventral edge of skull (0); jugal contributes to ventral edge of skull between maxilla and quadratojugal, but does not project laterally beyond toothrow (1); jugal laterally overlaps toothrow (2) (ordered).** We have merged two correlated characters that we interpret as parts of a continuous character. This character is not applicable to taxa with state JUG 1(1); a maxilla-quadratojugal contact can be present when there is no jugal (if the quadratojugal is present: QUAJUG 1(0)), but this is then the only possible manifestation of MAX 6(0) and thus already covered in this matrix.

State 0 is present in *Ventastega* (Ahlberg, Lukševičs & Lebedev, 1994) and *Eocaecilia* (Jenkins, Walsh & Carroll, 2007). We have kept it for *Oestocephalus*, where the lower temporal bar is interrupted but the maxilla extends much farther caudally than the jugal does (Carroll, 1998a; Anderson, 2003a); the same condition appears to be present in **Coloraderpeton* (Anderson, 2003a; Pardo et al., 2017).

Gephyrostegus has state 1 (Klembara et al., 2014), as does *Lethiscus* (Pardo et al., 2017).

State 2 is not confined to pantylids; it is shared by *Broiliellus* (Carroll, 1964: figs. 9, 10).

Schoenfelderpeton is scored as unknown due to its paedomorphosis.

Unknown in *Leptoropha* (Bulanov, 2003: fig. 12). Inapplicable to *Batropetes* due to QUAJUG 1(1), to *Hapsidopareion*, *Pelodosotis* and **Llistrofus* due to CHE EMA 1(1), and to *Rhynchonkos* due to QUAJUG 1(?) (which was already scored by RC07); see below for those characters.

Unknown in **Nigerpeton* (D. M., pers. obs. of MNN MOR 69 and MNN MOR 70).

**Lydekkerina* is polymorphic, having state 0 on the left and state 1 on the right side of the holotype (Hewison, 2007).

We have scored state 0 for **Diploradus* and **Aytonerpeton* after Clack et al. (2016: matrix).

73. JUG 3: **Jugal/pterygoid contact: absent (0); present (1).**

Eocaecilia (Jenkins, Walsh & Carroll, 2007), *Limnoscelis* (Reisz, 2007; Berman, Reisz & Scott, 2010) and *Lethiscus* (Pardo et al., 2017) have state 0. So does *Ossinodus*, in

spite of the palatal exposure of the jugal (Warren, 2007) that gives state 1 to *Cochleosaurus* (as already scored; Sequeira, 2004), **Saharastega* (Damiani et al., 2006) and **Konzhukovia* (Gubin, 1991) but not **Palatinerpeton* (Boy, 1996), **Platyoposaurus* (Gubin, 1991: drawing 3б) or **Australerpeton* (Eltink et al., 2016).

Unknown in *Edops* (Romer & Witter, 1942: fig. 3B; D. M., pers. obs. of MCZ 1378) and *Rhynchonkos* (Szostakiwskyj, Pardo & Anderson, 2015).

**Nigerpeton* has state 0 as illustrated by Steyer et al. (2006: fig. 2B), although that drawing is downright idealized (D. M., pers. obs. of MNN MOR 70, where these bones are very hard to identify; they are not preserved in the other two skulls).

Milner & Sequeira (2011) considered state 1 probably present in **Erpetosaurus*. Although this is not clear from their figures 2 and 5, we have accepted it. (Milner & Sequeira, 2011: 65, referred to a specimen number and fig. 2, but the specimen with that number is shown in fig. 5 instead, at least judging from the captions.)

Clack et al. (2012b: 22) implied state 0 for the *St. Louis tetrapod by stating: “The ectopterygoid appears to contribute to the margin of the adductor fossa.” The caudal (distal) end of the preserved right ectopterygoid, however, is broken off; fig. 2B of Clack et al. (2012b) is misleading in representing the break at the caudal end of the specimen as a line, implying a vertical surface – it is an inclined surface (D. M., pers. obs. of MB.Am.1441.1 and MB.Am.1441.2). The lingual margin does not appear to be broken, but is too irregular to represent the edge of the subtemporal fenestra; most likely, then, it is part of the sutural surface for the pterygoid. By comparison to other animals, the caudal end of the ectopterygoid would be expected far distal to the ectopterygoid fang rather than such a short distance distal to it. On the left side, most of the large Meckelian fenestra is preserved; if the mesiodistal overlap between the adductor fossa and the Meckelian fenestra was not substantially larger than in *Greererpeton* (Bolt & Lombard, 2001: fig. 5), the entire adductor fossa of the lower jaw and thus the entire subtemporal fenestra must have lain well distal (caudal) of the entire preserved fragment of the ectopterygoid. We have therefore scored the *St. Louis tetrapod as unknown.

74. JUG 4: **Maximum depth of jugal ventral to orbit greater (0) or smaller (1) than half of anteroposterior eye diameter.** One might think that this character is size-related, with larger animals having relatively smaller eyes at comparable ontogenetic stages and therefore deeper jugals ventral to them, but that is clearly not the case in our taxon samples.

Ventastega has state 1 (Ahlberg, Lukševičs & Lebedev, 1994). So do Albanerpetidae (McGowan, 2002; Venczel & Gardner, 2005) and *Lethiscus* (Pardo et al., 2017).

For *Trimerorhachis* (not including ?*T. sandovalensis*), where the jugal is excluded from the orbit margin by a strikingly long lacrimal/postorbital suture, we have combined the width of the jugal with that of the lacrimal and/or postorbital. Doing so marginally results in state 0 (Milner & Schoch, 2013).

We have scored *Eucritta* as unknown because only juvenile specimens are known and relative eye size decreases in the ontogeny of most animals.

Batropetes is polymorphic, with *B. fritschi* having state 1 while *B. niederkirchensis* and *B. palatinus* have state 0 (in lateral view) (Glienke, 2013, 2015).

Even the largest adults of **Glanochthon angusta* show both states (Schoch & Witzmann, 2009b: fig. 2A, B).

75. JUG 7: **Jugal without (0) or with (1) V-shaped indentation of its orbital margin.**

State 1 is present in *Ventastega* (Ahlberg, Lukševičs & Lebedev, 1994) and, though weakly expressed, in *Eucritta* (Clack, 2001: fig. 4, 6) and *Edops* (D. M., pers. obs. of MCZ 1378; not very well visible in Romer & Witter, 1942: fig. 1).

For *Trimerorhachis* (not including ?*T. sandovalensis*), where the jugal does not contact the orbit, we have applied this character to the lacrimal and the postorbital instead; this confirms the score of 0 (Milner & Schoch, 2013).

Unknown in *Westlothiana* (Smithson et al., 1994). Also unknown in *Baphetes* and *Megalocephalus* due to the antorbital fenestra and/or incomplete preservation (Beaumont, 1977; Milner, Milner & Walsh, 2009), as well as in **Spathicephalus*, where the jugal may not even reach the orbit proper (Beaumont & Smithson, 1998; Smithson et al., 2017).

**Sclerocephalus* is polymorphic, sometimes between the left and right sides of the same individual (Schoch & Witzmann, 2009a).

76. JUG 8: **Jugal not extending (0) or extending (1) anterior to orbit anterior margin.** This character is only applicable to OTUs with MAX 5/PAL 5(0) and applies to the (dorso)lateral surface of the skull, not to underlapping processes.

We do not count the baphetoid antorbital fenestra as part of the orbit. Although *Eucritta* nonetheless seems to have state 0 as already scored (Clack, 2001: fig. 4, 6, 8), *Baphetes* and *Megalocephalus* have state 1 (Beaumont, 1977).

Lethiscus has state 0 (Pardo et al., 2017).

State 1 is present in *Ventastega* (Ahlberg, Lukševičs & Lebedev, 1994) and *Limnoscelis* (Berman, Reisz & Scott, 2010: fig 3B).

Unknown in *Westlothiana* (Smithson et al., 1994).

77. QUAJUG 1: **Separately ossified quadratojugal: present (0); absent (1).**

State 0 is present in *Ventastega* (Ahlberg, Lukševičs & Lebedev, 1994).

Batropetes appears to have state 1 (Glienke, 2015).

Notobatrachus is polymorphic (Báez & Nicoli, 2008).

The quadratojugal is unknown in *Hapsidopareion*; CG78 interpreted this as genuine absence. However, for unknown reasons, *Hapsidopareion* has been assigned state 0 of this character ever since Ruta, Coates & Quicke (2003); neither Ruta, Coates & Quicke (2003) nor RC07 commented on this. Bolt & Rieppel (2009) pointed out that **Llistrofus* has state 0 and that the absence in *Hapsidopareion* is likely taphonomic; we have scored *Hapsidopareion* as unknown.

Most or all of the supposed squamosal of *Phlegethontia* (Anderson, 2002) is better considered the quadratojugal by comparison to other aïstopods, particularly *Lethiscus* (Pardo et al., 2017); we have scored state 0 of the present character.

The quadratojugal of **Nigerpeton* identified in Steyer et al. (2006: fig. 1B) is not reproducible; the area where this bone would be expected is ossified, but the suture between the jugal and the quadratojugal is not preserved in MNN MOR 70 and wholly covered by hard sandstone in MNN MOR 69 (D. M., pers. obs.). We have consequently scored **Nigerpeton* as unknown for all three QUAJUG characters.

All four species of **Liaobatrachus* are said to have state 0, but in three of the four species the quadratojugal is said to be fused to the quadrate, and no statement has been made about the fourth (*L. beipiaoensis*; Dong et al., 2013). The published figures do not resolve the situation. Given the shape of the supposedly compound bone and the lack of an explicit statement about fusion or lack thereof in *L. beipiaoensis*, we have scored state 0 for the time being, but this should be investigated further.

Without further comment, Schoch, Poschmann & Kupfer (2015) described their specimens of **Chelotriton* as having quadratojugals separate from their quadrates. As discussed by Marjanović & Witzmann (2015), this would be the first documented case in all of Urodela; while separate bones are clearly shown in the line drawings (Schoch, Poschmann & Kupfer, 2015: fig. 4b–d), the photograph (fig. 4a) is unclear. However, the putative quadrato-

jugals would immediately be considered quadratojugals if *Chelotriton* were compared only to other taxa in this matrix rather than to other salamandrids, having as they do a large ornamented surface that is sutured to those of the maxillae and the squamosals. We have here accepted them as such and scored state 0.

In **Bystrowiella*, the preserved margins of the jugal and the squamosal make it hard to imagine that a separate quadratojugal, or perhaps a separate-looking one as in **Chelotriton*, was absent; we have scored state 0 for this character (but not the next four, which remain unknown).

We interpret the unlabeled purple slivers in Pardo et al. (2017: ext. data fig. 4) as the articulated, though probably incomplete, quadratojugal of **Coloraderpeton*.

78. QUAJUG 2: **Quadratojugal depth less than one-fourth of squamosal depth: absent (0); present (1).**

State 0 is present in *Ventastega* (Ahlberg, Lukševičs & Lebedev, 1994), apparently *Asaphestera* (CG78) and *Lethiscus* (Pardo et al., 2017).

Unknown in *Scincosaurus* (Milner & Ruta, 2009).

Notobatrachus reigi has state 1 (Báez & Nicoli, 2008).

79. QUAJUG 3: **Quadratojugal anteroposteriorly elongate and bar-like: no (0); yes (1).**

State 0 is present in *Ventastega* (Ahlberg, Lukševičs & Lebedev, 1994) and *Lethiscus* (rather elongate, but a plate, not a bar, barely reaching the temporal fenestra: Pardo et al., 2017).

State 1 is reconstructed for *Eocaecilia* (Jenkins, Walsh & Carroll, 2007) and, though borderline so, for **Spathicephalus* (Beaumont & Smithson, 1998).

80. QUA 1: **Quadrate without (0) or with (1) dorsal process caudal to temporal embayment.** RC07 did not specify which dorsal process they meant; the one they scored as present in *Seymouria*, *Diadectes* and *Limnoscelis* is simply the ossification of the dorsal part of the shaft, rostromedial to where the embayment is or would be and clearly not homologous to the process found in dissorophoid temnospondyls.

That process is not limited to dissorophoids, however (among which it is also found in *Ecolsonia* [Berman, Reisz & Eberth, 1985: 16] and **Micropholis* [Schoch & Rubidge, 2005]): as Hook (1993) pointed out, *Chenoprosopus* has state 1, even though the process of USNM 437646 seems to have been accentuated by damage and it is at best very difficult to trace the sutures around the quadrate in that specimen (D. M., pers. obs.). State 1 further occurs in **Iberospondylus* (Laurin & Soler-Gijón, 2006) and in the stereospondyls **Lydekkerina* (Jeannot, Damiani & Rubidge, 2006: "hyoid tubercle"; Hewison, 2007: "quadrate tubercle", "quadrate boss") and **Australerpeton* (Barberena, 1998; Eltink et al., 2016: fig. 8: caudolateral to the "tympanic crest").

Clearly, this character is inapplicable in the absence of a temporal embayment (SQU 3(0)) or if the quadrate is inclined caudodorsally to rostroventrally (JAW ART 1/SQU 2/DEN 8(3/4)).

State 0 is found in *Solenodonsaurus* (Danto, Witzmann & Müller, 2012), *Discosauriscus* (Klembara, 2009), *Microphon* (Bulanov, 2014: fig. 2) and *Orobates* (Nyakatura et al., 2015: digital reconstruction).

Unknown in **Nigerpeton* (D. M., pers. obs. of MNN MOR 69 and MNN MOR 70).

81. PREOPE 1: **Preopercular: present (0); absent (1).** RC07 had exchanged the states in the text but not in the matrix.

Ventastega (Ahlberg, Lukševičs & Lebedev, 1994) and *Ossinodus* (Warren, 2007) have state 0.

So does *Whatcheeria* (Lombard & Bolt, 1995; Bolt & Lombard, 2000). Ruta, Coates & Quicke (2003), the preceding version of RC07, cited Clack (1998, 2001) for the absence of the preopercular (state 1) in all post-Devonian tetrapods, but Clack (1998) confirmed the presence of the preopercular in *Whatcheeria* (as part of the argument for the absence of this bone in *Crassigyrinus*), and Clack (2001) mentioned neither the preopercular nor *Whatcheeria*.

Unknown in *Batropetes* (Glienke, 2013, 2015), *Lethiscus* (area not preserved: Pardo et al., 2017) and **Nigerpeton* (D. M., pers. obs. of MNN MOR 69 and MNN MOR 70).

The position of the putative preopercular in **Coloraderpeton* (Pardo et al., 2017: ext. data fig. 4, video; scored as present in their matrix) excludes all alternatives rather clearly; we have scored state 0.

deleted NOS 1: **Nostrils posterolaterally expanded: absent (0); present (1).** RC07 stated: "This is a feature of branchiosaurid dissorophoids, in which the external nostril outline widens in its posterolateral portion (Boy & Sues, 2000)." They proceeded to score state 1 for their three branchiosaurids (and no other OTUs). Yet, while state 1 is clearly present in *Apateon dracyi* (Schoch & Milner, 2008: fig. 1H) and arguably in *A. pedestris* (fig. 1G), it is clearly not in *Schoenfelderpeton*, where the nostril has a narrow caudal extension instead (fig. 4A). *Leptorophus* has a wider caudal extension, but it does not seem wider than the rostral half of the nostril, at least not in dorsal view (fig. 4B). State 0 is clearly found in *Apateon gracilis* (Schoch & Fröbisch, 2006), the sister-group to most or all of the rest of *Apateon* (Schoch & Milner, 2008). Scoring *Schoenfelderpeton* as having state 0 and *Apateon* as polymorphic makes this character parsimony-uninformative regardless of the condition in *Leptorophus*, so we have deleted it. This incidentally obviates the question of correlation with NOS 3, which describes a lengthening and widening of the nostril in its caudolateral part.

State 0 is present in *Ventastega* (Ahlberg, Lukševičs & Lebedev, 1994).

Unknown in *Westlothiana* (Smithson et al., 1994).

82. NOS 3: **Nostrils: margins concave throughout (0); intermediate (1); keyhole-shaped (2) (ordered).** The intermediate state is new; it accounts for *Broiliellus* (Schoch, 2012: fig. 1F) and some, though not all, **other dissorophids (Schoch, 2012) as well as the amphibamid ***Georgenthalia* (Anderson et al., 2008b), where the dorsal and ventral margins are convex, approaching each other somewhat, but the extreme elongation of the nostril seen in state 2 is not reached. We have also assigned it to the very long nostrils of **Archaeovenator* (Reisz & Dilkes, 2003).

State 0 is present in *Ventastega* (Ahlberg, Lukševičs & Lebedev, 1994) and *Solenodonsaurus* (Danto, Witzmann & Müller, 2012).

Brachydectes appears to have state 0 or 1 (Pardo & Anderson, 2016: fig. 3A, B).

Phlegethontia seems to have state 2 (Anderson, 2007a: fig. 2).

**Saharastega* has state 0 or 1 regardless of which candidate holes actually are the nostrils (D. M., pers. obs. of MNN MOR 73).

83. NOS 4: **Nostrils elliptical, with greater axis orientated obliquely in anteromedial to posterolateral direction: absent (0); present (1).** RC07 added a further restriction on the length of the external nares in relation to the suture between the nasals, but this would make the character correlated to the length of the snout (for instance, it would require **Saharastega* to have 10-cm-long nares to qualify for state 1, which it otherwise does whether or not Damiani et al. [2006] correctly identified the nares) and inapplicable in taxa without a suture between the nasals or without nasals.

Inapplicable to greatly expanded nostrils (NOS 3(2) – *Acheloma*, *Phonerpeton*, *Ecolsonia*, **Mordex*).

State 0 is present in *Ventastega* (Ahlberg, Lukševičs & Lebedev, 1994).

State 1 is present in *Isodectes* (Sequeira, 1998), *Trimerorhachis* (Milner & Schoch, 2013), *Balanerpeton* (Milner & Sequeira, 1994), *Eryops* (though the nostrils are not very elliptical: Sawin, 1941), *Broiliellus* (Carroll, 1964; Schoch, 2012), *Eoscopus* (borderline: Daly, 1994), apparently *Platyrhinops* (Werneburg, 2012a), *Micromelerpeton* (Schoch, 2009b), albanerpetids where known (McGowan, 2002; Venczel & Gardner, 2005), *Eocaecilia* (Jenkins, Walsh & Carroll, 2007), *Caerorhachis* (other shapes would be difficult to accommodate: Ruta, Milner & Coates, 2002), *Gephyrostegus* (Klembara et al., 2014: fig. 5–7), *Diadectes* (Kissel, 2010: fig. 34–36), *Limnoscelis* (Berman, Reisz & Scott, 2010), *Petrolacosaurus* (Reisz, 1981), apparently *Asaphestera*, *Hapsidopareion*, *Micraroter*, *Pelodosotis*, *Rhynchonkos*, *Cardiocephalus* (at least *C. peabodyi*), *Euryodus* and *Hyloplesion* (CG78; Szostakiwskyj, Pardo & Anderson, 2015), *Microbrachis* (Vallin & Laurin, 2004), *Brachydectes* (an extreme case: Wellstead, 1991; Pardo & Anderson, 2016), *Lethiscus* (Pardo et al., 2017), *Capetus* (even though its nostrils are quite small: Sequeira & Milner, 1993), *Orobates* (Berman et al., 2004) and *Tseajaia* (Moss, 1972). It is also present in *Batropetes*, though the reconstructions in dorsal and lateral view by Glienke (2013: fig. 2E, F) contradict each other on the exact angle.

Unknown in *Paleothyris* (Carroll, 1969b) and *Westlothiana* (Smithson et al., 1994); unclear and likely unknown in *Saxonerpeton* and *Odonterpeton* (CG78).

Dendrerpetidae is polymorphic, with state 0 appearing in *Dendrysekos* (Holmes, Carroll & Reisz, 1998) and state 1 in at least some *Dendrerpeton* specimens (A. R. Milner, 1980, 1996); **Acanthostomatops* is likewise polymorphic, sometimes within the same individual (Witzmann & Schoch, 2006a).

We assign state 1 to **Sclerocephalus* even though its nostrils have an additional triangular extension that points dorsomedially and even though the caudal margin is pointed in some cases (Schoch & Witzmann, 2009a).

84. INT FEN 1: **Internarial fenestra or fontanelle: absent, mosaic of "postrostral" bones (0); present (1); absent, no "postrostral bones" (2) (ordered).** State 2 is new (and corresponds to all instances of the original state 0 except *Eusthenopteron* and *Panderichthys*); the order follows the scenario suggested by Ahlberg et al. (2008).

RC07 counted the presence of "median rostrals/internasals" as state 1, regardless of whether there is a fenestra (fontanelles are not mentioned) present additional to them or not. We cannot replicate this decision and find the characters independent: *Ventastega* (Ahlberg et al., 2008) and *Acanthostega* (Clack, 2003b, 2007; Porro, Rayfield & Clack, 2015) possess both a fontanelle (state 1 of the present character) and a pair of "median rostrals"; baphetids have a pair of "median rostrals" and a fully closed snout roof (state 2); *Ichthyostega* shows a single "median rostral" and a fully closed snout (state 2); **Deltaherpeton* preserves a single "median rostral/internasal" and probably had a round fontanelle or at least a deep pit (state 1; Bolt & Lombard, 2010: figs. 1, 2, 3). We have therefore split this character and coded the number of "median rostrals/internasals" as a new one, MED ROS 1 (see below).

Lethiscus has state 1 (Pardo et al., 2017; J. Pardo, pers. comm.).

Silvanerpeton has state 2 (Ruta & Clack, 2006).

Unknown in *Eucritta* (Clack, 2001) and *Edops* (Romer & Witter, 1942; D. M., pers. obs. of MCZ 1378). Also unknown in *Platyrhinops lyelli* (Clack & Milner, 2010), but *P. fritschi* almost certainly shows state 2 (Werneburg, 2012a).

States 1 and 2 occur in *Trimerorhachis*, even in *T. insignis* alone (Milner & Schoch, 2013). The same appears to hold for Dendrerpetidae, with state 1 appearing in *Dendrysekos*

(Holmes, Carroll & Reisz, 1998) while state 2 is probably present in *Dendrerpeton confusum* (Milner, 1996: fig. 7B); whether *D. acadianum* and *D. rugosum* have state 1 or 2 appears to be unknown (A. R. Milner, 1980, 1996).

Balanerpeton has state 1 or 2 (specimen drawings in Milner & Sequeira, 1994).

Apateon has states 1 and 2, with *A. gracilis* having state 2 throughout its ontogeny (Schoch & Fröbisch, 2006).

State 2 seems pretty clear in **Aytonerpeton* (Clack et al., 2016: supplementary video 2).

85. MED ROS 1: **"Median rostrals"/"medial rostrals"/"internasals": paired (0); single (1); absent (2) (unordered).** This character is split off from INT FEN 1 (see above). Pawley (2006: appendix 14) introduced a character with the same abbreviation, but only distinguished presence from absence – our state 1 would have been parsimony-uninformative in her matrix.

All three names for these bones are unsatisfying. Strictly speaking, two bones in a transversely arranged pair cannot both be median, only a single bone can be; more importantly, the homology of these bones to any part of the "postrostral" mosaic of finned sarcopterygians is an underresearched and difficult problem – several pairs of "rostrals" can be "medial". The "internasals" never lie only between the nasals, and in *Ventastega* they do not lie between them at all – they do not even contact the nasals, instead the premaxillae border the internarial fenestra, and the "internasals" lie far rostral of the "nasals" in their entirety (Ahlberg et al., 2008: fig. 3c, 4a). – The name "medial rostral" occurs only, to the best of our knowledge, in fig. 6 of Clack (2002), while the text of Clack (2002, 2003b) used "median".

We have scored *Eusthenopteron* and *Panderichthys* as unknown because homology is unclear, and *Crassigyrinus* likewise because its snout roof is very difficult to interpret. In *Eucritta*, this area of the skull is not preserved in any specimen (Clack, 2001), and the same holds for *Edops* (Romer & Witter, 1942; D. M., pers. obs. of MCZ 1378).

One specimen of *Megalocephalus* has state 1, unlike the others, which have state 0 (Beaumont, 1977: 51, 79); we have scored polymorphism.

Otherwise, state 0 occurs in *Ventastega* (Ahlberg et al., 2008), *Acanthostega* (Clack, 2002, 2003b, 2007; Porro, Rayfield & Clack, 2015) and *Baphetes* (Beaumont, 1977); 1 is restricted to *Ichthyostega*, **Deltaherpeton* and **Elginerpeton*; state 2 accounts for all other OTUs with a known snout roof.

Part of the supposed premaxilla of **Aytonerpeton* could be a "medial rostral" (Clack et al., 2016: supplementary video 2); we have scored state 0 or 2.

86. ORB 1: **Interorbital distance greater than (0), subequal to (1), or smaller than half of skull […] width at the same level (2) (ordered).** We have exchanged states 1 and 2 to make ordering of this continuous character possible and added the specification on where to measure skull width.

Ventastega has state 2 (Ahlberg et al., 2008), as do *Cochleosaurus* (Sequeira, 2004), *Brukterpeton* (Boy & Bandel, 1973), *Notobatrachus* (Báez & Nicoli, 2004), *Vieraella* (Báez & Basso, 1996) and *Tseajaia* (Moss, 1972; Berman, Sumida & Lombard, 1992 – CM 38033, seen by D. M., is too distorted for comparison).

Dendrerpetidae is polymorphic, with *Dendrysekos* reaching state 1 as already scored (Holmes, Carroll & Reisz, 1998) but *Dendrerpeton* having state 2 (A. R. Milner, 1980, 1996).

The situation is unclear in *Hapsidopareion* due to disarticulation (CG78: fig. 13A).

Diadectes is polymorphic, having states 0 and 1 (Berman, Sumida & Lombard, 1992; Berman, Sumida & Martens, 1998). So is *Batropetes*, with state 0 documented in *B. niederkirchensis* and *B. palatinus* and state 1 likely present in *B. fritschi* (Glienke, 2013, 2015).

Brachydectes has state 0 (Wellstead, 1991; Pardo & Anderson, 2016).

Danto, Witzmann & Müller (2012) claimed state 0 for *Solenodonsaurus*. This is incorrect according to their fig. 3, which shows the already scored state 2.

87. ORB 2: **Interorbital distance greater than (0), subequal to (1), or smaller than maximum orbit diameter (2) (ordered).** We have exchanged states 1 and 2 to allow ordering of this continuous character.

State 0 is found in *Acanthostega* (Porro, Rayfield & Clack, 2015) and *Brachydectes* (Pardo & Anderson, 2016). We further keep state 0 for Dendrerpetidae because it is found in *Dendrysekos* (Holmes, Carroll & Reisz, 1998), *Dendrerpeton rugosum* (A. R. Milner, 1980) and *D. confusum* (A. R. Milner, 1980, 1996); only *D. acadianum*, which is only known from considerably smaller specimens than the other two species, reaches state 1 (A. R. Milner, 1980, 1996; Schoch & Milner, 2014).

State 1 is found in *Ichthyostega* (Clack & Milner, 2015: fig. 8) and *Trimerorhachis* (Milner & Schoch, 2013).

State 2 is present in *Ventastega* (Ahlberg, Lukševičs & Lebedev, 1994; Ahlberg et al., 2008).

Tseajaia shows state 1 or arguably 2 (Moss, 1972, Berman, Sumida & Lombard, 1992); we have decided on state 1. (CM 38033, seen by D. M., is too distorted for comparison.)

Eucritta is scored as unknown for having juvenile eye size.

We have scored Albanerpetidae, *Karaurus*, *Valdotriton*, *Notobatrachus*, *Vieraella*, **Beiyanerpeton* and **Pangerpeton* as unknown, because it is unknown how much of the orbitotemporal fenestra is homologous to the orbit. (RC07 had scored *Brachydectes* as unknown, presumably for the same reason; though see above.) Only the interorbital width of *Triadobatrachus* is so small that even the smallest realistic estimates for eyeball size result in state 2, which was already scored. In several specimens of **Liaobatrachus*, the eyes are preserved as circular stains, which are about as wide as the interorbital distance; allowing for some space around the eye in the orbit proper, we have therefore scored state 2 for **Liaobatrachus*.

As for most lissamphibians, we have assigned state 1 or 2 to **Spathicephalus*.

**Cheliderpeton* passes from state 2 to state 0 in ontogeny (Werneburg & Steyer, 2002: fig. 1).

States 1 and 2 are found in the largest illustrated adults of **Glanochthon* (Schoch & Witzmann, 2009b: fig. 2).

88. ORB 3/LAC 5: **Rostroventral margin of orbit: round (0); angled (1); "antorbital vacuity" present (2) (ordered).** State 2, which corresponds to the original LAC 5(1), occurs in *Baphetes*, *Megalocephalus* and **Spathicephalus*; we count it as a state of this character because it makes state 1 inapplicable and because it may be an exaggerated version of it. Because its ontogenetic development is unknown, we have scored *Eucritta* as having state 1 (as observed) or 2.

State 1 is also observed in *Acanthostega* (Clack, 2007; Porro, Rayfield & Clack, 2015), *Eocaecilia* (Jenkins, Walsh & Carroll, 2007), *Seymouria* (both species: Laurin, 1996a, 2000; Klembara et al., 2005, 2006, 2007) and *Batropetes* (Glienke, 2013, 2015) as well as *Caseasauria (especially *Eothyris*) and **Neopteroplax* (Romer, 1963).

Dendrerpetidae is polymorphic: the previously scored state 0 is found in *Dendrerpeton rugosum* and *D. acadianum* (A. R. Milner, 1980, 1996: fig. 4A), while *Dendrysekos* reaches state 1 (Holmes, Carroll & Reisz, 1998: fig. 4C).

The condition in *Westlothiana* is unknown (Smithson et al., 1994).

*_Palaeoherpeton_ (like _Ventastega_ and _Ichthyostega_) appears to have a rostral and a ventral angle, but a negative rostroventral one, thus state 0 (Panchen, 1964).

89. ORB 4: **Orbit deeper than long: no (0); yes (1).**

State 0 is present in _Ventastega_ (Ahlberg, Lukševičs & Lebedev, 1994) and _Brachydectes_ (Pardo & Anderson, 2016) and seems highly likely in *_Aytonerpeton_ (Clack et al., 2016: especially supplementary video 2).

Eocaecilia has state 0 as already scored (Jenkins, Walsh & Carroll, 2007). However, we have scored all other modern amphibians as unknown because it is unknown how much of the orbitotemporal fenestra is homologous to the orbit.

90. ORB 5: **Orbit anteroposterior diameter shorter than (0), subequal to (1), or longer than (2) distance between orbit posterior margin and suspensorium anterodorsal margin (ordered).** We have exchanged states 1 and 2 to make ordering of this continuous character possible, and interpret the rostral end of the temporal embayment (if present) as "suspensorium anterodorsal margin". Note that the wording implies a rostrodorsally-to-caudoventrally-inclined suspensorium; when the suspensorium is inclined the other way around (ch. 146: state JAW ART1/SQU 2/DEN 8(3,4), see below), we have used its caudodorsal end as the "anterodorsal margin".

"The distribution of the different states of this character is extremely irregular and shows several instances of parallel developments and reversals", wrote RC07. On the shortest of our trees from Analysis R4, it waxes and wanes gently for a total of 39 steps; there are only five direct transitions between states 0 and 2. State 2 holds Dissorophoidea and *_Iberospondylus_ together (ambiguously also _Balanerpeton_, depending on the position of *_Palatinerpeton_); there is only a single reversal in this clade (within *_Micropholis_, an OTU which shows all three states). State 2 is further synapomorphic between _Ventastega_ and _Acanthostega_, and between _Whatcheeria_ and _Pederpes_; state 1 ties at least the latter together with everything as far crownward as Amniota, with a total of 11 reversals to state 0 (four of them in Anthracosauria). Clearly, this character carries phylogenetic signal.

Euryodus has state 0 (CG78); so does _Brachydectes_ (Pardo & Anderson, 2016).

Not counting the antorbital fenestra (see ch. 88: ORB 3/LAC 5) rostral to the lateral outgrowth of the prefrontal (ch. 15: PREFRO 7), _Baphetes_ and _Megalocephalus_ have state 0 as well (Beaumont, 1977).

Ventastega (Ahlberg et al., 2008) has state 2, as do _Acanthostega_ (Clack, 2003b, 2007; Porro, Rayfield & Clack, 2015), _Balanerpeton_ (Milner & Sequeira, 1994, with the exception of the rather small specimen in fig. 4 that may have state 1), _Phonerpeton_ (an extreme case of state 2: Dilkes, 1990; D. M., pers. obs. of AMNH 7150 and MCZ 2313), _Doleserpeton_ (Sigurdsen & Bolt, 2010) and _Gephyrostegus_ (Klembara et al., 2014).

We have scored all modern amphibians other than _Eocaecilia_ and *_Chelotriton_ as unknown because it is unknown how much of the orbitotemporal fenestra is homologous to the orbit.

All three states occur in *_Micropholis_ (Schoch & Rubidge, 2005).

While _Oedaleops_ has state 2, the larger _Eothyris_ has state 1; we have scored only the latter state for *Caseasauria.

*_Cheliderpeton_ passes from at least state 1 to state 0 in ontogeny (Werneburg & Steyer, 2002: fig. 1).

91. PIN FOR 1: **Pineal foramen present in adults (0); absent in adults (1).** Ruta, Coates & Quicke (2003) had coded the presence and the position of the pineal foramen as PIN FOR 1 and PIN FOR 2, respectively. Ruta & Coates (2007) merged them into a character they called

PIN FOR 2. We kept this in the first two preprints of this paper (Marjanović & Laurin, 2015, 2016) except for Analysis EB. Here we separate them again because the pineal foramen has a known position – PIN FOR 2(1) – in immature *Cochleosaurus* even though it is lost in adults (Milner & Sequeira, 2003; Sequeira, 2004, and references therein), showing that absence does not in fact make position inapplicable. Further, PIN FOR 2 can now simply be ordered instead of requiring a stepmatrix.

The situation in *Hapsidopareion* is unclear because of disarticulated parietals and frontals (CG78: figs. 13, 14).

State 1 is now known in *Rhynchonkos* (Szostakiwskyj, Pardo & Anderson, 2015).

Both species of *Cardiocephalus* are polymorphic, and so is *Micraroter* (CG78).

Euryodus is polymorphic, with *E. primus* having state 1 and *E. dalyae* having state 0 (CG78).

92. PIN FOR 2: **Pineal foramen caudal to (0), at the level of (1), or rostral to (2) interparietal suture midlength (ordered).**

Unknown in *Ventastega* (Ahlberg et al., 2008).

Acanthostega has state 2 (Porro, Rayfield & Clack, 2015).

Ichthyostega appears to have states 0 and 1 (Clack & Milner, 2015: 21, fig. 8).

Chenoprosopus has state 0 (Reisz, Berman & Henrici, 2005).

State 1 is found in *Cochleosaurus* (Milner & Sequeira, 2003).

Limnoscelis shows state 1 (Berman, Reisz & Scott, 2010: fig. 3A).

The situation in *Hapsidopareion* is unclear because of disarticulated parietals and frontals (CG78: figs. 13, 14).

Micraroter has state 2 (Carroll & Gaskill, 1978).

Although *Phlegethontia* lacks parietals, we have kept state 2 because the frontals reach the pineal foramen (Anderson, 2002).

*Caseasauria has states 0 and 1 (state 1 in *Eothyris*, state 0 in *Oedaleops*: Reisz, Godfrey & Scott, 2009).

States 0, 1 and 2 are documented in **Glanochthon*, apparently independent of onto-genetic age (Schoch & Witzmann, 2009b: fig. 2).

Milner & Sequeira (2011: 63) stated that state 1 is “almost” reached in **Erpetosaurus*, and reconstructed state 1 (fig. 11); their photos and drawings of specimens, however, show varying extents of state 2. Because Milner & Sequeira (2011) documented the existence of many more specimens than they figured, we have scored polymorphism.

In **Neopteroplax*, only state 2 can be excluded; the presence of the foramen (PIN FOR 1) remains unknown (Romer, 1963: fig. 1).

93. L SC SKU 1: **Lightly sculptured area (subdued ornament) adjacent to skull roof midline: absent (0); present (1).**

State 0 is present in *Ventastega* (Ahlberg et al., 2008).

State 1 makes a surprise appearance in *Ossinodus* (Warren & Turner, 2004).

Steyer et al. (2006: appendix 2: ch. 39) scored state 1 of this character for **Nigerpeton*, but did not mention it anywhere in the text and illustrated it only ambiguously (fig. 2A). Consequently, we scored state 0 in the first two preprints of this paper (Marjanović & Laurin, 2015, 2016). State 1 is in fact correct, so we have now scored it: although some-what difficult to find or to photograph, it is found on the frontals and a third of the parietals of MNN MOR 70 (D. M., pers. obs.).

Although conditions that may count as state 1 are widespread in **Chelotriton* (Schoch, Poschmann & Kupfer, 2015), MB.Am.45 has state 0, which we have scored.

94. PTF 1: **Posttemporal fossa occurring at occiput dorsolateral corner, delimited dorsally by skull table, not bordered laterally and floored by dorsolateral extension of opisthotic (0); fossa present near occiput dorsolateral corner, delimited dorsally by occipital flanges of tabular and postparietal and bordered laterally as well as ventrally by dorsolateral extension of opisthotic meeting tabular ventromedial flange (1); small fossa present near occiput ventrolateral corner, bordered laterally by tabular ventromedial flange, delimited dorsally by dorsal portion of the lateral margin of the suproccipital–opisthotic complex and floored by lateral extension of opisthotic (2); absence of fossa (3) (unordered).** It is possible that this character should be ordered or be treated according to a more complex stepmatrix, but we are not sure about this and cannot find a suggestive pattern in the data.

State 0 is present in *Panderichthys* (Brazeau & Ahlberg, 2006) and *Ventastega* (Ahlberg et al., 2008) and makes a surprise appearance in *Edops* (D. M., pers. obs. of MCZ 1378 – the short paroccipital process has an unfinished end in this huge skull).

The potentially informative specimen of *Chenoprosopus*, USNM 437646, is crushed, but state 1 is the best fit (D. M., pers. obs.).

Doleserpeton shows state 1, even though the lateral border is formed by the tabular rather than the opisthotic (Sigurdsen, 2008). This is common in stereospondyls as well, including **Australerpeton* (Barberena, 1998; Eltink et al., 2016).

State 2 fits the condition reconstructed for *Batropetes* best, though there may not have been any floor (Glienke, 2013).

Rhynchonkos shows state 2 or 3 (Szostakiwskyj, Pardo & Anderson, 2015); *Lethiscus* has state 1 or 3 (Pardo et al., 2017).

Eocaecilia (Jenkins, Walsh & Carroll, 2007), *Brachydectes* (Pardo & Anderson, 2016) and *Orobates* (Berman et al., 2004) have state 3.

Triadobatrachus has state 1 (ignoring the fact that the frontoparietal plays the roles of the absent postparietals and tabulars) or 3; despite the crushing and the preservation as a natural mold, the other states can most likely be excluded (Ascarrunz et al., 2016: 3D model 1).

**Saharastega* has state 1 or 3 (D. M., pers. obs. of MNN MOR 73).

State 1 makes a somewhat unexpected appearance in *Caseasauria (*Eothyris*; Reisz, Godfrey & Scott, 2009). While the fenestra is too small to reach the postparietal, state 1 is otherwise a perfect match; state 2, expected for very early amniotes, is not.

**Quasicaecilia* has state 0 or 3 (Pardo, Szostakiwskyj & Anderson, 2015: fig. 2).

95. SKU TAB 1: **Ratio of width to postorbital midline length of skull table: ≤ 1 (0);]1;1.5] (1);]1.5;2] (2);]2;2.5] (3); > 2.5 (4) (ordered).** We have recoded this character much like we did with PREMAX 7. Ruta, Coates & Quicke (2003) worded it as follows, under the section headline "Proportions of skull table" (italics in the original):

"SKU TAB 1. *Absence (0) or presence (1) of condition: postorbital region of skull roof abbreviated.* Although shortening of the postorbital skull roof region occurs in various degrees, several crown-group taxa are distinctly different from the outgroup and from stem-tetrapods in that their skull roof is usually wider than long, regardless of the morphology and proportions of its various constituent bones. Several temnospondyls more crownward than trimerorhachoids […] and crown-lissamphibians […] have acquired the derived state of this character independent of several basal and crown-amniotes, *Westlothiana*, microsaurs, *Scincosaurus* and derived diplocaulids […]."

Compare RC07 (italics in the original):

“SKU TAB 1. *Postorbital region of skull table abbreviated and at least one-third wider than long: absent (0); present (1).* The derived state is acquired in parallel by total group amniotes and amphibians (except the most basal taxa of both groups).”

The 2003 version was irreproducible. The 2007 version is close to unambiguous (fully so for many OTUs) if taken at face value, but the question remains whether the skull table or the skull roof was meant (see main text). As with PREMAX 7, the original scores fail to provide evidence: the width/length ratio of the skull table, the width/length ratio of the skull roof, the ratio of the width of the skull table to the length of the skull roof and the ratio of the width of the skull roof to the length of the skull table are all about equally bad matches for the scores by RC07 (Appendix-Table 3, Data S5). We have decided in favor of the skull table for both measurements (the postorbital length of the skull table is, as in TAB 7, the distance between the caudal extremities of the orbits/orbitotemporal fenestrae – averaged where necessary – and the transverse level of the caudal end of the skull table in the sagittal plane); one reason is the fact that the postorbital length of the skull roof depends on the position of the caudal extremities of the suspensoria, which is already a character in this matrix (JAW ART 1/SQU 3/DEN 8).

Like PREMAX 7, this is a continuous character which we have arbitrarily divided into states by creating a state for each interval of 0.5 (lumping the few extreme outliers above 3 into the same state as the other OTUs above 2.5).

The ratios, sources, and state changes are presented in Appendix-Table 3; they and our measurements are contained in Data S5. In salientians, the caudal margin of the orbitotemporal fenestra was taken to be the rostral margin of the otic capsule, not of the lateral process of the parietal that covers only the caudal or caudomedial part of the otic capsule.

**Llistrofus* has state 0 (Appendix-Table 3). *Hapsidopareion* appears to differ starkly in having state 2, but this could be ontogenetic: the skull the reconstruction (CG78: fig. 13B) is based on is much smaller than the **Llistrofus* skull, and indeed only about half as long as another skull which CG78 referred to *Hapsidopareion* but only illustrated in ventral view (fig. 14E); the latter skull appears to have a proportionally longer postorbital region. We therefore scored *Hapsidopareion* as having state 0, 1 or 2.

Unknown in *Acherontiscus* (Carroll, 1969a) and *Dolichopareias* (Andrews & Carroll, 1991).

Appendix-Table 3: Ratios of skull table width to postorbital (postorbitotemporal) skull table length, and changes to the scores of character 95 (SKU TAB 1). Taxa underlain in blue were scored 0 by RC07, taxa underlain in yellow were scored 1, and the remainder were scored as unknown or have been added by us; the latter are marked with an asterisk. Abbreviation: *Ph.*, *Pholiderpeton*. See Data S5 and its legend for more information.

OTU	Ratio (rounded)	New score	Measured in:
**Coloraderpeton*	0.231	0	Anderson, 2003a: fig. 3A; not measurable in Pardo et al., 2017, but looks the same and clearly does not leave state 0
**Llistrofus*	0.515	0	Bolt & Rieppel, 2009
**Pseudophlegethontia*	0.600	0	Anderson, 2003b: fig. 3
Phlegethontia	0.652	0	Anderson, 2007a
Odonterpeton	0.698	0	CG78: fig. 99B
Sauropleura	0.706	0	Bossy & Milner, 1998
Eocaecilia	0.716	0	Jenkins, Walsh & Carroll, 2007

**Erpetosaurus*	0.724	0	Milner & Sequeira, 2011
Oestocephalus	0.732	0	Anderson, 2003b
Adelogyrinus	0.745	0	Andrews & Carroll, 1991
Notobatrachus	0.813	0	Báez & Nicoli, 2004
**Sparodus*	0.826	0	Carroll, 1988
Lethiscus	0.836	0	J. Pardo, pers. comm. 2017
Adelospondylus	0.852	0	Andrews & Carroll, 1991
Captorhinus	0.906	0	Fox & Bowman, 1966: fig. 3
Neldasaurus	0.912	0	Schoch & Milner, 2014: fig. 20C
Colosteus	0.983	0	Hook, 1983
Ventastega	1.000	0	Ahlberg et al., 2008
Urocordylus	1.000	0	Bossy & Milner, 1998
Greererpeton	1.074	1	Smithson, 1982
Crassigyrinus	1.078	1	Clack, 1998
**Pholidogaster*	1.087	1	Panchen, 1975
Isodectes	1.090	1	Schoch & Milner, 2014: fig. 20E
Paleothyris	1.091	1	Carroll, 1969b: fig. 4B
Panderichthys	1.103	1	Vorobyeva & Schultze, 1991
**Deltaherpeton*	1.106	1	Bolt & Lombard, 2010: fig. 2; right side approximately doubled
**Utaherpeton*	1.111	1	Carroll, Bybee & Tidwell, 1991
Euryodus	1.119	1	CG78: fig. 37
Eusthenopteron	1.130	1	Clack, 2007
**Chroniosaurus*	1.132	1	Clack & Klembara, 2009
**Cheliderpeton*	1.155	1	Werneburg & Steyer, 2002
**Archegosaurus*	1.159	1	Witzmann, 2006: fig. 5
Baphetes	1.159	1	Beaumont, 1977: fig. 21
Chenoprosopus	1.170	1	Schoch & Milner, 2014: fig. 13E
Trimerorhachis	1.191	1	Schoch & Milner, 2014: fig. 20A
Eucritta	1.200	1	Clack, 2001: fig. 8
Ptyonius	1.200	1	Bossy & Milner, 1998
**Australerpeton*	1.206	1	Eltink et al., 2016: fig. 5
**Glanochthon*	1.214	1	Schoch & Witzmann, 2009b: fig. 2A approximately doubled
Ph. attheyi	1.228	1	Panchen, 1972
Cardiocephalus	1.228	1	CG78: fig. 69
Pantylus	1.281	1	Romer, 1969: fig. 1
Proterogyrinus	1.304	1	Holmes, 1984
Caerorhachis	1.306	1	Ruta, Milner & Coates, 2002
Silvanerpeton	1.344	1	Ruta & Clack, 2006
Ph. scutigerum	1.346	1	Clack, 1987b
**Konzhukovia*	1.348	1	Gubin, 1991: drawing 6a
**Sclerocephalus*	1.358	1	Schoch & Witzmann, 2009a
**Neopteroplax*	1.366	1	Romer, 1963: fig. 3
Cochleosaurus	1.388	1	Schoch & Milner, 2014: fig. 13D
**Palatinerpeton*	1.404	1	Boy, 1996: fig. 3
Albanerpetidae	1.414	1	*Celtedens*: McGowan, 2002
**Platyoposaurus*	1.419	1	Gubin, 1991: drawing 3a

Balanerpeton	1.458	1	Schoch & Milner, 2014: fig. 17A
Bruktererpeton	1.458	1	estimated from Boy & Bandel, 1973: fig. 7
Edops	1.459	1	Schoch & Milner, 2014: fig. 13A
Acanthostega	1.465	1	Porro, Rayfield & Clack, 2015
Dendrerpetidae	1.466	1	*Dendrysekos*: Schoch & Milner, 2014: fig. 17C
Megalocephalus	1.468	1	Beaumont, 1977: fig. 8
Capetus	1.500	1	Sequeira & Milner, 1993
Micromelerpeton	1.500	1	Beaumont, 1977: fig. 8
Westlothiana	1.500	1	Smithson et al., 1994
**Palaeoherpeton*	1.500	1	Panchen, 1964
Kotlassia	1.542	2	Bulanov, 2003: fig. 30
Keraterpeton	1.545	2	Bossy & Milner, 1998
Whatcheeria	1.586	2	Lombard & Bolt, 1995: fig. 1A
Vieraella	1.594	2	Báez & Basso, 1996: fig. 6, 7
**Mordex*	1.605	2	Schoch & Milner, 2014: fig. 32A
Gephyrostegus	1.614	2	Klembara et al., 2014
**Lydekkerina*	1.634	2	Hewison, 2007: fig. 30
Stegotretus	1.667	2	Berman, Eberth & Brinkman, 1988: fig. 10B
Hyloplesion	1.667	2	CG78: fig. 89B
Ichthyostega	1.667	2	Clack & Milner, 2015: fig. 8
Pederpes	1.674	2	Clack & Finney, 2005
**Iberospondylus*	1.685	2	Laurin & Soler-Gijón, 2006: fig. 1A left side approximately doubled
Microbrachis	1.689	2	Vallin & Laurin, 2004
Ossinodus	1.705	2	Warren, 2007
Valdotriton	1.711	2	Evans & Milner, 1996
**Micropholis*	1.750	2	Schoch & Rubidge, 2005: fig. 3B
Phonerpeton	1.778	2	Dilkes, 1990: fig. 1
Discosauriscus	1.780	2	Klembara et al., 2006
Rhynchonkos	1.825	2	CG78: fig. 63
Pelodosotis	1.829	2	CG78: fig. 48
Ariekanerpeton	1.854	2	Klembara & Ruta, 2005a
Seymouria	1.877	2	Laurin, 1996a
Archeria	1.900	2	Holmes, 1989
*NSM 994 GF 1.1	1.920	2	Holmes & Carroll, 2010
Amphibamus	1.938	2	Schoch & Milner, 2014: fig. 30
Eoherpeton	1.943	2	Panchen, 1975
Brachydectes	1.965	2	Pardo & Anderson, 2016: fig. 4b
Solenodonsaurus	1.972	2	Danto, Witzmann & Müller, 2012
**Karpinskiosaurus*	2.020	3	Klembara, 2011
Scincosaurus	2.027	3	Milner & Ruta, 2009
Doleserpeton	2.044	3	Sigurdsen & Bolt, 2010
Schoenfelderpeton	2.048	3	Boy, 1986: fig. 13
Broiliellus	2.050	3	Carroll, 1964
**Bystrowiella*	2.051	3	Witzmann & Schoch, 2017: fig. 15C
Diceratosaurus	2.061	3	Bossy & Milner, 1998
Orobates	2.092	3	Kissel, 2010: fig. 32B
**Saharastega*	2.105	3	D. M., pers. obs. of MNN MOR 73

**Nigerpeton*	2.113	3	Steyer et al., 2006
Eoscopus	2.130	3	Daly, 1994: fig. 3
Leptorophus	2.156	3	Boy, 1986: fig. 4
Eryops	2.167	3	Schoch & Milner, 2014: fig. 3C
Petrolacosaurus	2.186	3	Berman, Sumida & Lombard, 1992: fig. 11
Micraroter	2.189	3	CG78: fig. 56
Microphon	2.195	3	Bulanov, 2003: fig. 22
Asaphestera	2.326	3	CG78: fig. 7
Karaurus	2.333	3	Ivachnenko, 1978
**Acanthostomatops*	2.333	3	Witzmann & Schoch, 2006a
Limnoscelis	2.364	3	Kissel, 2010: fig. 13A[1]
Ecolsonia	2.371	3	Berman, Reisz & Eberth, 1985: fig. 5A
Anthracosaurus	2.388	3	Clack, 1987a
Acheloma	2.395	3	Polley & Reisz, 2011
Batrachiderpeton	2.397	3	Bossy & Milner, 1998
*Caseasauria	2.398	3	*Eothyris*: Reisz, Godfrey & Scott, 2009
Batropetes	2.445	3	Glienke, 2013: fig. 2
Platyrhinops	2.471	3	Clack & Milner, 2010: fig. 9
Saxonerpeton	2.471	3	CG78: fig. 22
**Carrolla*	2.563	4	Maddin, Olori & Anderson, 2011
Utegenia	2.627	4	Klembara & Ruta, 2004a
**Gerobatrachus*	2.657	4	estimated from Anderson et al., 2008a: fig. 2b
**Tungussogyrinus*	2.795	4	Werneburg, 2009
Apateon	2.860	4	Schoch & Fröbisch, 2006: fig. 1D
**Spathicephalus*	2.867	4	Beaumont & Smithson, 1998: fig. 5
Tuditanus	2.889	4	Carroll & Baird, 1968: fig. 9
**Archaeovenator*	2.963	4	Reisz & Dilkes, 2003
Tseajaia	2.976	4	Berman, Sumida & Lombard, 1992: fig. 11
**Liaobatrachus*	3.100	4	Dong et al., 2013
**Crinodon*	3.500	4	CG78: fig. 11
**Chelotriton*	3.571	4	Marjanović & Witzmann, 2015: fig. 7
Triadobatrachus	3.724	4	Roček & Rage, 2000; roof estimated
**Quasicaecilia*	3.809	4	Pardo, Szostakiwskyj & Anderson, 2015: fig. 3
Diplocaulus	5.238	4	Olson, 1951: pl. 5B left side doubled
Diploceraspis	5.849	4	Beerbower, 1963: fig. 2
Diadectes	6.280	4	Kissel, 2010: fig. 36B

[1] A line drawing identical to Berman, Reisz & Scott (2010: fig. 3A), but slightly more convenient to measure.

96. FONT 1: **Dorsal exposure of frontoparietal fontanelle: absent (0); present (1).**

State 0 is present in *Ventastega* (Ahlberg et al., 2008). This was already correctly scored by RC07, who had not known of that material. Probably they intended to score *Ventastega* for TEM FEN 1, but slipped one column to the left; *Ventastega* indeed has state 0 of that character (Ahlberg, Lukševičs & Lebedev, 1994).

97. TEM FEN 1: **Broad opening in skull postorbital region: absent (0); present (1).** RC07 had added "(aïstopod pattern)", but potential primary homologues of the aïstopod temporal fenestra can be found elsewhere.

Most obviously, the diapsid *Petrolacosaurus* has two temporal fenestrae. In principle, they could have arisen from a single fenestra that was subdivided by a contact between processes of the postorbital and the squamosal. There is no evidence that this actually happened, but, firstly, to take this into account would mean to insert assumptions about secondary homology into the determination of primary homology; secondly, the lateral temporal fenestra has subdivided itself in just such a fashion (by a contact between neomorphic processes of the jugal and the squamosal) in several **dinosaurs, e.g. ***Cryolophosaurus* (Hammer & Hickerson, 1994), giving these animals a total of three temporal fenestrae per side. We have therefore scored *Petrolacosaurus* as possessing state 1.

On the other hand, fenestrae can become confluent with other openings. Both temporal fenestrae (per side) and the orbit of Cenozoic and many Mesozoic **birds have indeed merged in such a way; at least in principle, the orbitotemporal fenestra found in *Brachydectes* and all modern amphibians in this matrix other than *Eocaecilia* might be the result of a similar merger. We have therefore scored all these OTUs as sharing state 1. This does not, however, apply to the very elongate orbit of *Batropetes*, which retains the postfrontal and postorbital bones at its caudal margin (Glienke, 2013, 2015).

Finally, states 1 of this character and the next are not mutually exclusive. In particular, the temporal fenestra of most diapsids is open ventrally, as the contact between jugal and quadratojugal has been lost. Because of the orientation of the quadratojugal in **Llistrofus* (Bolt & Rieppel, 1999: fig. 4), we have scored state 1 of the present character for that OTU.

State 0 is in any case present in *Ventastega* (see FONT 1 above).

98. CHE EMA 1: **Ventral emargination of cheek (pattern of certain tuditanomorph microsaurs): absent (0); present (1).** This pattern is not limited to *Hapsidopareion*, *Micraroter* and *Pelodosotis* (and **Llistrofus*): a very mild version occurs in *Batropetes* (Glienke, 2015), a less mild one in *Lethiscus* (Pardo et al., 2017; also scored in their matrix) and *Phlegethontia* (Anderson, 2002). We have scored *Oestocephalus* and **Coloraderpeton* as unknown because there is no non-phylogenetic way to tell if their ventrally open temporal fenestra is continuous with an emargination (Carroll, 1998a; Anderson, 2003a; Pardo et al., 2017, excluding their matrix, where state 1 was scored for **Coloraderpeton*).

State 0 is present in *Ventastega* (Ahlberg et al., 2008).

Unknown in **Nigerpeton* (D. M., pers. obs. of MNN MOR 69 and MNN MOR 70).

The cheek (jugal, quadratojugal) is absent in *Brachydectes*, possibly *Triadobatrachus*, and all salamanders except (see QUAJUG 1 above) **Chelotriton*; we have accordingly scored them as unknown.

deleted IFN 1: **Interfrontonasal: absent (0); present (1).** As RC07 explicitly mentioned, this character is parsimony-uninformative because state 1 is limited to *Eryops*. We have therefore deleted it. Our addition of **Crinodon* has made it parsimony-informative again, but there would be little point in reintroducing this character, given the fact that **Crinodon* and *Eryops* are among the least similar taxa in the entire matrix except where symplesiomorphies are concerned.

Recently an interfrontal has been discovered in most specimens of *Batropetes*, though it is absent in some of both *B. fritschi* and *B. palatinus* (Glienke, 2013, 2015; tentatively confirmed for *B. palatinus* by D. M., pers. obs. of MB.Am.1232.1). Because PAUP* cannot reconstruct ancestors as polymorphic, state 1 could not hold *Batropetes* and **Crinodon* together.

The evidence for paired interfrontonasals in any colosteids (Bolt & Lombard, 2010) is not convincing. If *Greererpeton* has a single bone between the frontals, nasals and premaxillae, it should probably be considered a "median rostral" (see MED ROS 1 above) rather than an interfrontonasal.

One specimen of **Sclerocephalus* has an interfrontonasal (Boy, 1988: 116), as does one of **Lydekkerina* (Jeannot, Damiani & Rubidge, 2006). Given the fact that so many more specimens of both (Jeannot, Damiani & Rubidge, 2006; Schoch & Witzmann, 2009a) lack it, and the fact that the **Lydekkerina* individual with an interfrontal also has an interparietal (Jeannot, Damiani & Rubidge, 2006), we have decided to ignore these cases as aberrations of development. In *Eryops*, every one of the many known skulls shows the interfrontonasal, while even its **closest relatives always lack it (Werneburg, 2007b, 2012b; Werneburg & Berman, 2012).

99. SUS 1: **Anteroposteriorly narrow, bar-like squamosal: absent (0); present (1).**

State 0 is present in *Ventastega* (Ahlberg, Lukševičs & Lebedev, 1994) and most likely *Batropetes* (Glienke, 2013: fig. 6H; contra Carroll, 1991). It is also known in *Lethiscus* (Pardo et al., 2017) and **Saharastega* (D. M., pers. obs. of MNN MOR 73).

State 1 is found in all modern amphibians in this matrix except *Eocaecilia* (0 as already scored) and *Vieraella* (unknown). We note, however, that ***Gobiates*, possibly closely related to **Liaobatrachus*, has state 0 (Roček, 2008).

Cardiocephalus was scored as sharing state 1 in RC07. The squamosal shown on both sides of the skull in CG78: fig. 30A is dorsoventrally narrow, not rostrocaudally. We have scored state 0.

Most of the squamosal of **Carrolla* is unknown, but state 1 is likely enough (Maddin, Olori & Anderson, 2011) that we have scored it as present.

100. SC 1: **Lateral-line system on skull roof totally enclosed (0), mostly enclosed with short sections in grooves (1), mostly in grooves with short sections enclosed (2), entirely in grooves (3), absent (4) (ordered).** RC07 had "skull table" instead of "skull roof". We have ordered this and the following character because the present sequence of states represents a gradual movement of the lateral-line organ from inside the bones to their surface and beyond (the organ is present in extant aquatic lissamphibians, but never leaves traces on bones).

States 0 and 4 can be difficult to distinguish from the outside. Indeed, Warren (2007) reported that *Edops*, *Chenoprosopus* and *Eryops*, previously thought to have state 4 (but see below for *Chenoprosopus*), have state 0. We have scored both as possibilities in *Edops* and *Eryops*, because it is not clear if this canal in the quadratojugal which had no connection to the outside – at least in that bone – is homologous to a lateral-line canal or rather carried nerves and/or blood vessels; Čerňanský et al. (2016) did not commit to any interpretation and called for more research. Because the quadratojugal of *Cochleosaurus* is only known in external (dorsolateral and edge-on ventral) view, we have scored it the same way.

Pawley (2006: 188) claimed that *Dendrerpeton* (Dendrerpetidae) has "pits and perforations" for the postcranial lateral line in the ventralmost row of dorsal scales, and Schoch (2013: 21; not citing Pawley, 2006) even spoke of "possible lateral line sulci", both implying that the absence of traces of cranial lateral lines in this taxon (e.g. A. R. Milner, 1980, 1996) is unexpected and may turn out to be erroneous. However, Pawley (2006) and Schoch (2013) both cited only Dawson (1882) for these observations. Although Dawson (1882: 647) mentioned "minute round pores, probably mucous or perspiratory pores" in dorsal scales ("horny scales", as opposed to the more strongly mineralized "bony" ventral ones) and mentioned that the ventralmost row of dorsal scales is "in some specimens" composed of unusually large scales "forming a sort of Vandyke edging", he gave no indication

that the pores are restricted to that row. The closest Dawson (1882: 647) came to such a statement is the following somewhat enigmatic sentence: “In front the skin projects into long pendant lappets, terminated by similar angular points, and covered with oval scales, not imbricated, and each having a pore in its centre.” No pores or pits or sulci are illustrated in plate 40; fig. 5 (p. 648) shows a pore on one scale without indicating where on the body it lay. Earlier, Dawson (1863: pl. IV: fig. 25 – not cited by Pawley, 2006, or Schoch, 2013) had drawn pores on each one of six or seven rows of scales of “*Dendrerpeton oweni*” (junior synonym of *D. acadianum*: Milner, 1996; Schoch & Milner, 2014), and the accompanying description gives no indication that the supposed pores were – one might guess – only visible on one row and extrapolated to the others; rather, “[a] limited portion of the upper, and I suppose, anterior part [of an isolated patch of scales preserved in contact with “many fragments of the skull” of *D. acadianum*] is covered with imbricated scales, which […] generally have a small spot or pore near the outer margin” (Dawson, 1863: 34). – There is another patch of scales (Dawson, 1863: pl. V: fig. 22–24) where very large scales, each with a supposed pore, form a row between much smaller dorsal scales (drawn too small to determine if pores were present) and ventral scales (drawn too small in fig. 22, not shown in fig. 23–24). It was described as follows (Dawson, 1863: 36–37): “The best preserved specimen (Fig. 22 [of pl. V]), which is about one inch in length and half an inch in breadth, is covered with very small imbricated scales. It is crossed by six or seven obscure ridges, which both at the bottom and along a mesial line, projected into points covered with larger scales. A row of large scales with round pores, connects these along the lower side (Figs. 23 and 24.) [sic] If, as seems probable, this fragment belonged to the side of the trunk or tail, it would perhaps indicate a division of the sub-cutaneous muscles into an upper and lower band, as in the newts.” This patch, which Dawson (1863) tentatively referred (not in the text, only in the legend to plate V on page 48) to the nomen dubium (Steen, 1934) **“*Hylonomus*” *wymani*, is similar to the patch drawn in p. VI, fig. 62, where a row of very large scales separates two areas of much smaller ones, but no pores are shown (only parallel striations); that patch was referred to “*Hylonomus aciedentatus*”, which is a junior synonym of *D. acadianum* (Milner, 1996; Schoch & Milner, 2014). – Given that the supposed pores are not restricted to a single row in at least one specimen, we suspect that the “pores” may be growth centers – denser than the rest of the scale, not a hole in it. In any case, Dawson (1882) cannot be cited as evidence that any dendrerpetid had a postcranial lateral line, and while one specimen described and illustrated by Dawson (1863) may in principle provide such evidence, such a conclusion requires numerous assumptions about correct anatomical identification and the referral of that specimen. At present, thus, no dendrerpetid should be thought to have had a postcranial lateral line; if Pawley (2006) and Schoch (2013) did not merely miscite Dawson (1863 or 1882), they must have relied on unpublished personal observations which they did not mention. In any case, however, it is painfully obvious that dendrerpetid scales have never been adequately described or illustrated. Dawson’s descriptions of his light-microscopic observations are superficial and confused by modern standards, and his line drawings – in stark contrast to contemporary illustrations of dinosaur or crocodyliform bones, which have been called “almost better than the bone” – are of extremely limited usefulness.

Ventastega shows state 1 (Ahlberg, Lukševičs & Lebedev, 1994). RC07 scored it as 0, but probably they intended to score *Ventastega* for SC 2, the only mandible character they did not score, and slipped one column to the left; *Ventastega* indeed has state 0 of SC 2 (Ahlberg, Lukševičs & Lebedev, 1994).

“Lateral line sulci intermittently present, but state of preservation and coarse sculpture tend to obscure their courses except anterolateral to nares and partly across quadratojugal” in *Chenoprosopus* (Langston, 1953: 365). The smaller specimen USNM 437646 lacks lateral-line sulci (Hook, 1993; D. M., pers. obs.), but this may be ontogenetic: perhaps sulci only

appeared with progressing metaplastic ossification of the dermis. We have scored state 3, which may be visible in CM 34909 (D. M., pers. obs.) as a sulcus (wide, shallow sculpture) rostral to the right naris (this area is not preserved on the left side) and possibly along the left jaw margin (this area is not preserved on the right side); the most convincing candidate for a lateral-line sulcus lies on the left jugal ventral to the orbit (this area is not preserved on the right side). Mehl's (1913) "mucus canal" must be the nasolacrimal canal and has not been mentioned in more recent literature as far as we know.

Isodectes was scored in RC07 as possessing state 1. There is, however, no clear evidence for enclosed canals anywhere on the animal; we have been cautious and scored the interrupted grooves (Sequeira, 1998) as state 2 or 3.

Trimerorhachis was scored as having state 2 or 3, yet there is no evidence for state 2 (Milner & Schoch, 2013); we have scored 3 alone.

Acheloma shows state 4 (Polley & Reisz, 2011).

Traces of the lateral lines have never been mentioned or reconstructed for *Broiliellus* (Carroll, 1964; Schoch, 2012; Schoch & Milner, 2014). Because they would be preserved and visible, we have scored state 4.

Traces of the lateral lines have never been mentioned for *Amphibamus* and are absent from all reconstructions (most recently Schoch & Milner, 2014: fig. 30A). Because the implied state 4 may be visible in Daly (1994: fig. 18), we have scored it.

Micromelerpeton has state 4 when adult (Schoch, 2009b).

Apateon was scored as unknown; the only species known from metamorphosed individuals shows state 4 – apparently throughout ontogeny (Werneburg, 1991: 85).

Albanerpetidae has state 4 according to all literature about this taxon.

Proterogyrinus was scored as possessing state 1 or 2. It has state 3; the grooves, where present at all, are shallow and not bridged, but evidently interrupted (Holmes, 1984) like in **Archegosaurus* (Witzmann, 2006) – the lateral-line organ was not deeper in the bone, but deeper in the thicker skin than in more obvious cases of state 3.

Similarly, *Archeria* was given state 2 or 3, while it has state 3 (Holmes, 1989); *Pholiderpeton attheyi* was scored 2, but shares 3 (Panchen, 1972: 288–291, fig. 4); and *Anthracosaurus*, usually claimed to have state 4 but scored 1 by RC07, shows state 3 as well (Clack, 1987a).

Discosauriscus reached state 4 when adult (Klembara, 2009). This is interpreted as part of a transition to terrestrial life. Accordingly, we have added state 4 as an option (partial uncertainty) to all seymouriamorphs for this and the following character, except of course *Seymouria* (which already had state 4 for both) and *Kotlassia* (which is unknown for both).

Batropetes was scored 1; it has state 4 – the large pits on the frontals are unrelated to lateral-line canals (Glienke, 2015).

Saxonerpeton was scored as completely unknown, but CG78: 33 probably allow only states 3 and 4; we have scored it as possessing state 3 or 4.

Microbrachis has state 3 (Olori, 2015).

Of the traces identified in *Hyloplesion* by Olori (2015), not all are convincing – notably those on the maxilla (Olori, 2015: fig. 28C) are very narrow, sharp and irregular –, but the remainder (in particular Olori, 2015: fig. 28A, B dorsal of the arrow, D) are good enough to score state 3, which is not unexpected in this apparently fully aquatic animal.

Acherontiscus was scored as having state 1 or 2. It rather clearly has state 3, with wide, long, likely interrupted rather than bridged grooves (Carroll, 1969a).

Adelospondylus, too, was scored as having state 1 or 2. Judging from Andrews & Carroll (1991: 254, fig. 13A, B, 14A, B), it is better scored as having state 3 or 4, because the possible grooves are wide and may well be interrupted rather than bridged.

Dolichopareias was scored as having state 1. In the absence of evidence that the wide grooves (Andrews & Carroll, 1991) continued inside the bone, we have changed the score to 3.

Scincosaurus is polymorphic, with most specimens having state 4 but some showing a very weak version of state 3 instead (Milner & Ruta, 2009).

Bossy & Milner (1998: 83–84, 122) stated that diplocaulids, without further qualifications, have lateral-line canals; to our surprise, all diplocaulids were originally assigned state 4. *Keraterpeton* (Bossy & Milner, 1998: 83–84, 122) and *Batrachiderpeton* (Bossy & Milner, 1998: fig. 58B) have state 3, and so do *Diceratosaurus* (D. M., pers. obs. of MB.Am.778 – very deep sculpture lies mostly, but not only around the orbits) and *Diploceraspis* (Beerbower, 1963); in the absence of evidence that *Diplocaulus* had state 4, we have scored it as unknown.

Notobatrachus has state 4 according to all figures.

Ossinodus has state 1 or 2 (Warren, 2007).

We have scored **Chroniosaurus* as possessing state 0 or 4; state 4 occurs in the specimens described by Clack & Klembara (2009), state 0 possibly in quadratojugals attributed to "*Jugosuchus*", some of which could be referable to **Chroniosaurus* (Clack & Klembara, 2009: 17).

**Nigerpeton* has state 2. The fact (best visible in MNN MOR 108; D. M., pers. obs.) that the grooves lie on top of the sculpture instead of under it excludes states 0 and 1; the fact that the infranarial canal is visible in MNN MOR 69 but not the less eroded MNN MOR 70 (D. M., pers. obs.) excludes state 3.

State 3 cannot be excluded in **Saharastega* (D. M., pers. obs. of MNN MOR 73); this means partial uncertainty between states 3 and 4.

Beaumont & Smithson (1998: 191) stated about **Spathicephalus mirus*: "In none of the skulls is there evidence of [the] lateral line canal system". In his description of **S. pereger*, Baird (1962: unnumbered table) did not mention the lateral lines at all, but showed a groove that seems to loop around the orbit and might have carried a lateral-line canal, if indeed it was continuous, which is hard to tell from the photograph. We have kept the score of the **Spathicephalus* OTU as unknown.

**Sclerocephalus* is apparently polymorphic, with some of the largest individuals showing state 3 (at least on part of the skull) and others state 4 (Schoch & Witzmann, 2009a).

Gubin (1991: drawing 6) illustrated **Konzhukovia* as having state 4, or rather did not shade the sculptured surface sufficiently to make the sulci stand out (some patterns in the sculpture vaguely suggest a few); following personal observation, Pereira Pacheco et al. (2016: appendix 2) reported state 3, which we have scored.

The lateral-line canals of **Elginerpeton* are wholly enclosed in the postorbital (Ahlberg, 1998) and almost wholly enclosed in the premaxilla (Ahlberg, 1995); the probable maxilla (Ahlberg, 1995: table 1) has not been described or illustrated, and other skull-roof bones are unknown. We have tentatively scored state 1.

101. SC 2: **Mandibular canal totally enclosed (0), mostly enclosed with short sections in grooves (1), mostly in grooves with short sections enclosed (2), entirely in grooves (3), absent (4) (ordered).**

State 0 is present in *Ventastega* (see SC 1 above).

Ichthyostega is best scored as possessing state 0 or 1 (Clack et al., 2012a).

Whatcheeria should rather be scored as 1 than as 2 (Lombard & Bolt, 2006).

Surprisingly, Milner & Lindsay (1998) did not comment on the presence or condition of lateral-line canals in the *Baphetes* specimen they described. Their fig. 4 seems to show the mandibular canal extending over most, perhaps all the length of the preserved part of the

lower jaw; we have tentatively scored state 2 or 3. The specimen described by Milner, Milner & Walsh (2009) does not contradict this, but does not narrow it down either, showing state 1, 2 or 3.

Trimerorhachis has state 1, 2, or 3 (Milner & Schoch, 2013: fig. 7A).

Langston (1953: 369) wrote about the lower jaw of *Chenoprosopus*: "Lateral line canals ill-defined: marginal sulcus [= mandibular canal] appears on ventrolateral surface of angular, continuous forward across splenials following dentary-splenial sutures, terminates anteriorly at symphysis, perhaps continuous posteriorly with a shallow groove on ventral and posterior faces of surangular; dental sulcus [= oral canal], if present, poorly developed." Because we have not been able to find a statement in the literature that explicitly contradicts this, we have scored state 3.

Isodectes was scored in RC07 as possessing state 1. There is, however, no clear evidence for enclosed canals anywhere on the animal; we have been cautious and scored the interrupted grooves (Sequeira, 1998) as state 2 or 3.

Neldasaurus has state 2 or 3 as for the skull roof (Chase, 1965).

Acheloma (Polley & Reisz, 2011) and *Ecolsonia* (D. M., pers. obs. of CM 38017 and CM 38024) have state 4.

Traces of the lateral lines have never been mentioned or reconstructed for *Broiliellus* (Carroll, 1964; Schoch, 2012; Schoch & Milner, 2014). Because they would be preserved and visible, we have scored state 4.

Schoch (2009b) did not mention the lower jaw of *Micromelerpeton*, but given the lack of lateral-line grooves on the skull in the adult stage, we have added state 4 to the observed uncertainty of state 2 or 3 of larvae (which was scored in RC07).

Albanerpetidae has state 4 according to all literature about this taxon.

Klembara et al. (2014) drew attention to a groove on the dentary of *Gephyrostegus*, wondering whether it was a lateral-line canal (state 3) or housed a blood vessel (state 4). We have kept the latter score, because the groove is discontinuous on both sides of the individual shown in their fig. 3, the rostralmost grooves ending in foramina as do several shorter grooves.

Except for *Microbrachis*, which was correctly scored as having state 3, and *Rhynchonkos*, which was correctly given state 4, all "microsaurs" were scored either as having state 0 or 1 (partial uncertainty) or as entirely unknown in RC07. This was not commented on by RC07 or Ruta, Coates & Quicke (2003), so we can only speculate that statements by CG78 about pits and grooves on lower jaws were misinterpreted as referring to lateral-line canals and led to the common score of 0 or 1. It is clear from context that these statements all refer to ornament and traces of nerves or blood vessels. CG78: 166 stated unambiguously that "*Microbrachis* is unique in having a lateral line canal groove, running along the ventral margin of the angular, the posterior splenial and the dentary." This groove is illustrated in fig. 107A, and no such groove is shown on any other "microsaur" (fig. 103, 105, 107). The correction to state 4 concerns *Batropetes* (Glienke, 2013: 81; 2015), *Tuditanus* (Carroll & Baird, 1968; CG78: fig. 4), *Pantylus* (Romer, 1969: 23), *Asaphestera* (CG78: fig. 7; D. M., pers. obs. of NMC 10041), *Saxonerpeton*, *Hapsidopareion*, *Micraroter*, *Pelodosotis*, *Cardiocephalus*, *Euryodus*, *Hyloplesion* and *Odonterpeton* (specimen drawings in CG78). Olori (2015: 39, table S3) reported "distinct pores" on the dentary of *Hyloplesion* "which may also connect to the lateral line system" and scored state 3; we are unconvinced – pores could correspond to nerves or blood vessels, or to electroreceptory organs.

Unknown in *Scincosaurus* (Milner & Ruta, 2009).

Batrachiderpeton has state 3 (Bossy & Milner, 1998: 88, fig. 60C); so do *Diplocaulus* (Douthitt, 1917) and *Diploceraspis* (Beerbower, 1963). In the absence of evidence that any other diplocaulids had the (to our surprise) originally scored state 4, and given the facts that

Bossy & Milner (1998) did not single out *Batrachiderpeton* as unusual and did not mention *Diplocaulus* or *Diploceraspis* in this context, we have scored them (i.e. *Keraterpeton* and *Diceratosaurus*) as unknown; see also SC 1 above.

Notobatrachus has state 4 (Báez & Nicoli, 2004); apparently, so does *Lethiscus* (Pardo et al., 2017).

Ossinodus has state 1 or 2; the material described by Warren (2007) is insufficient to distinguish between the two, so we have scored accordingly, but it should be mentioned that the distribution of pores and open grooves on the postsplenial hints at an ontogenetic transition from 2 to 1 (if not even 0).

**Nigerpeton* shows state 3; because not the entire lower jaw is preserved, state 2 cannot be excluded (D. M., pers. obs. of MNN MOR 69, MNN MOR 70 and MNN MOR 108), so we have scored partial uncertainty.

The mandibular canal of the *St. Louis tetrapod appears to be (D. M., pers. obs. of MB.Am.1441.2) visible for a greater length than indicated by Clack et al. (2012b); we have scored state 2 rather than 1 or 2.

Godfrey & Holmes (1989) tentatively implied state 3 for the *Parrsboro jaw; we have accepted this.

The area that would have borne the mandibular canal is not preserved in **Australerpeton*, but part of the oral canal is preserved as an open groove (Eltink & Langer, 2014), so we have scored state 2 or 3.

102. VOM 1-13: **Vomer approximately as wide as long or wider (0), intermediate (1), at least 2½ times longer than wide (2) (ordered).** This is a merger of two correlated characters that described parts of a continuous character.

Some snouts are so long and narrow that only state 2 is possible, making this character inapplicable. (The shape of the snout is not directly a character in this matrix, but correlates to varying extents with several characters.) This concerns *Acheloma* (Polley & Reisz, 2011), *Archeria* (already scored as unknown), **Archegosaurus*, **Platyoposaurus* and **Australerpeton*. Furthermore, the combination of a long snout, round interpterygoid vacuities and a vomer/pterygoid contact (states VOM 5-10/PTE 10-12-18/INT VAC 1(2 or lower) and INT VAC 3(1), which together imply short pterygoids and long vomers) likewise makes state 2 of the present character inevitable; this concerns *Chenoprosopus* (Langston, 1953), *Cochleosaurus* (Sequeira, 2004), *Neldasaurus* (where "the vomers […] are approximately four times as long as they are wide" – Chase, 1965: 172), **Nigerpeton* (Steyer et al., 2006) and, borderline, **Glanochthon* (Schoch & Witzmann, 2009b). Note that in all of these the vomers are as wide as possible, occupying the entire space between the midline, the (pre)maxillae and the choanae.

State 0 is found in *Ventastega* (Ahlberg, Lukševičs & Lebedev, 1994), *Colosteus* (Hook, 1983: fig. 1), *Eoscopus* (Daly, 1994), and *Ariekanerpeton* (Klembara & Ruta, 2005a: fig. 13).

State 1 occurs in *Baphetes* (Beaumont, 1977: fig. 19), *Trimerorhachis* (Milner & Schoch, 2013), *Caerorhachis* (Ruta, Milner & Coates, 2002: fig. 5c), *Bruktererpeton* (though almost state 2; Boy & Bandel, 1973: fig. 7), *Batropetes* (Glienke, 2013, 2015), *Asaphestera* (most likely; CG78: fig. 7), apparently *Micraroter* (CG78: fig. 53, 56), *Rhynchonkos* (CG78: fig. 63; Szostakiwskyj, Pardo & Anderson, 2015: fig. 3A), *Diceratosaurus* (Jaekel, 1903; D. M., pers. obs. of MB.Am.778), *Capetus* (Sequeira & Milner, 1993: fig. 9), *Pederpes* (as reconstructed by Clack & Finney, 2005: fig. 17), *Silvanerpeton* (Ruta & Clack, 2006: fig. 8), and *Utegenia* (Klembara & Ruta, 2004a: fig. 14) unless this is ontogenetic. CG78 (fig. 13, 14) depicted *Hapsidopareion* as having state 2, which was scored in RC07; however, the condition in **Llistrofus* (Bolt & Rieppel, 2009), which falls near the upper end of state 1, strongly

suggests that this was a misinterpretation partly due to damage to the *Hapsidopareion* specimens and that the correct score is 1.

State 2 is present in *Lethiscus* (Pardo et al., 2017) and *Orobates* (Berman et al., 2004: fig. 3B).

In *Eoherpeton* (Smithson, 1985: fig. 8) and *Proterogyrinus* (Holmes, 1984: fig. 3), the vomer is entirely unknown, but the skull is too narrow for state 0 given the shape of the palatine, so we have ascribed state 1 or 2 to both.

As reconstructed (Panchen, 1972: fig. 7), *Pholiderpeton attheyi* has state 1 on the left and 2 on the right side. We have scored it as polymorphic.

Gephyrostegus has state 2 (Klembara et al., 2014).

The condition in *Kotlassia* is unknown (Bulanov, 2003).

State 1 or 2 is present in *Cardiocephalus* (CG78: fig. 30) and *Euryodus* (CG78: fig. 37, 38).

The vomers of *Microbrachis* are so inclined (rostromedially to caudolaterally) that we cannot decide between states 0 and 1 (Vallin & Laurin, 2004: fig. 5).

Ossinodus, too, has state 0 or 1 (Warren, 2007: fig. 6).

The vomers of **Karpinskiosaurus* are so inclined (rostromedially to caudolaterally) that we cannot decide between states 1 and 2 (Klembara, 2011: fig. 3C).

**Chelotriton* has state 1 when the toothed caudal processes that frame the cultriform process, characteristic of salamandrids, are taken into account, or 0 when they are ignored (Schoch, Poschmann & Kupfer, 2015); we have gone with the latter option in order to prevent a salamandrid autapomorphy from making **Chelotriton* look less amphibamid-like.

103. VOM 3: **Vomer with (0) or without (1) fangs comparable in size to, or larger than, marginal teeth (premaxillary or maxillary).**

State 0 is present in *Ventastega* (Ahlberg, Lukševičs & Lebedev, 1994) and *Acheloma* (Polley & Reisz, 2011).

Jarvik (1996) reconstructed *Ichthyostega* with state 1. Ahlberg, Lukševičs & Lebedev (1994) demonstrated that it has state 0 instead (which is also figured in the line drawing of Blom, 2005: fig. 3). This is further confirmed by Jarvik's (1996) own pl. 26:1 and by D. M.'s observations of TMM 41224-2, AMNH 23100 and MCZ 3361 – a total of five casts of the specimen figured in that plate (MGUH VP 6055). Clack & Milner (2015) mentioned this issue and provided further evidence for state 0, but did not make clear if specimens showing state 1 are known; for the time being, we have scored state 0.

The most mature known individual of *Amphibamus* – the neotype, YPM 794 – has state 0 (if only on one side: Daly, 1994: 27). We regard this as the adult condition (see also Marjanović & Laurin, 2008: 193). Schoch & Milner (2014: 56) stated that *Amphibamus* lacks fangs on the vomer, palatine and ectopterygoid, but puzzlingly did not mention Daly (1994) in the context of *Amphibamus* at all, even though they of course mentioned YPM 794 and cited Daly (1994) as a source for *Eoscopus*.

State 1 is found in *Doleserpeton* (Bolt, 1969; Sigurdsen & Bolt, 2010), *Batropetes* (Glienke, 2013, 2015), *Hyloplesion* (Olori, 2015), *Diceratosaurus* (Jaekel, 1903; D. M., pers. obs. of MB.Am.778 and CM 34656), *Lethiscus* (Pardo et al., 2017), *Orobates* (Nyakatura et al., 2015: digital reconstruction) and *Ossinodus* (Warren, 2007). Following the matrix of Pardo et al. (2017), we have also scored it for **Coloraderpeton*.

Apparently unknown in *Broiliellus* (Schoch, 2012: fig. 2F).

The vomer is wholly unknown in *Pelodosotis* (CG78: 80–81).

104. VOM 4: **Vomer without (0) or with (1) small teeth (denticles), the basal diameter and/or height of which is less than 30% of that of adjacent marginal teeth (premaxillary**

or maxillary) and remaining vomer teeth (if present). RC07 further specified for VOM 4 that the denticles "form[…] [a] continuous shagreen or discrete, [sic] patches", but denticles occur in other arrangements as well. The purely size-based distinction of "teeth" and "denticles" (here and in other characters below) may not be satisfactory, but in any case there is no histological difference (Gee, Haridy & Reisz, 2017).

State 0 is present in *Ventastega* (Ahlberg, Lukševičs & Lebedev, 1994), *Whatcheeria* (Lombard & Bolt, 1995) and all preserved vomer fragments of *Edops* (D. M., pers. obs. of MCZ 1378 – the pterygoid fragments bear denticles throughout) as well as in *Batropetes* (Glienke, 2013, 2015), *Scincosaurus* (Milner & Ruta, 2009) and *Tseajaia* (Moss, 1972).

The entire vomer (except the rostral edge and the intervomerine fossa) of *Acheloma* (Polley & Reisz, 2011), *Broiliellus* (Carroll, 1964) and *Platyrhinops* (Clack & Milner, 2010) is covered by denticles, giving them state 1. *Limnoscelis* (Berman, Reisz & Scott, 2010) and *Ossinodus* have state 1 as well (Warren, 2007).

Although the vomerine denticles are restricted to a single ridge, they are present (state 1) in *Chenoprosopus* (Hook 1993; D. M., pers. obs. of USNM 437646).

The vomer of *Bruktererpeton* has two rows of denticles in its caudal corner (Boy & Bandel, 1973). We count this as state 1.

Hapsidopareion (CG78: fig. 14E) has state 1; note that, on the morphologically left side of the latter, the vomer has a longitudinal break, and the medial fragment with its two remaining denticles was referred to the pterygoid by CG78.

Diceratosaurus shows both states: the narrow-snouted morph, such as CM 34656, has a curled row of full-sized teeth on each vomer, while the broad-snouted morph, exemplified by CM 34670, CM 67157 and CM 81507 as well as MB.Am.778, instead has a field of denticles largely arranged in several parallel rows (D. M., pers. obs.). Ontogeny is an unlikely explanation: CM 67157 is larger than CM 34656 and CM 34670 but has the same skull length as the narrow-snouted CM 34696. In the absence of further evidence we have scored *Diceratosaurus* as polymorphic.

**Micropholis* has a few scattered non-fang teeth on the vomer, but all appear to have a width well over 30% of that of the marginal teeth (Schoch & Rubidge, 2005: fig. 2B, ?D). It thus has state 0.

Although this is not obvious from Maddin, Olori & Anderson (2011), **Carrolla* has state 0 (D. M., pers. obs. of TMM 40031-54). We have followed the matrix of Pardo et al. (2017) in scoring state 0 for **Coloraderpeton* as well.

The vomer of **Glanochthon* "is covered by numerous tubercles that could represent vestigial denticles" (Schoch & Witzmann, 2009b: 126). We have scored it as unknown, not counting the very small teeth (which may well qualify as denticles) of the parachoanal toothrow (see VOM 8).

105. VOM 5-10/PTE 10-12-18/INT VAC 1: **Pterygoids sutured to each other plus contacting parasphenoid along entire length, interpterygoid vacuities therefore absent (0); pterygoids sutured only toward their rostral ends (1); contact between pterygoids absent, pterygoid/vomer suture still present (2); pterygoids do not contact vomers, but still overlap palatines medially for part of the palatines' length (3); pterygoids entirely caudal to palatines, but still reach well mesial to subtemporal fenestrae (4); pterygoids do not extend mesially beyond their contribution (if any) to the rostral rims of the subtemporal fenestrae (5) (ordered).** This is a fusion of six redundant characters that represent parts of a single continuous character, the gradual "retreat" of the palatal ramus of the pterygoid (from mesial to distal) coupled to size increase of the interpterygoid vacuities. The palatine, if present (see PAL 8 below), is excluded from contacting the interpterygoid vacuities or the parasphenoid in states 0 to 2 (VOM 10(0)) as opposed to states 3 and 4 (VOM

10(1)). States 4 and 5 include the condition where the ectopterygoid participates in the margin of the interpterygoid vacuity, found in some temnospondyls that are not included in this matrix. State 5 is a rewording of PTE 18(1): "Pterygoid palatal ramus a robust, abbreviated, flange-like to digitiform structure, as long as or barely longer than combined length of quadrate ramus plus basicranial articulation"; note that it is not limited to caudates as originally scored, but also occurs in *Broiliellus* (borderline: Schoch, 2012: fig. 2F), *Eocaecilia* (Jenkins, Walsh & Carroll, 2007), *Triadobatrachus* (Roček & Rage, 2000; Ascarrunz et al., 2016) and even *Diploceraspis* (Beerbower, 1963: fig. 4A). (Furthermore, it does not occur in the caudates **Beiyanerpeton* and **Chelotriton* [Roček & Wuttke, 2010; Gao & Shubin, 2012; Schoch, Poschmann & Kupfer, 2015]. We have, however, assigned state 5 to **Gerobatrachus*, where the mesial end of the pterygoid is barely mesial to the mesial tip of the subtemporal fenestra, even though [much like in *Broiliellus*] the rostral rim of the fenestra is mostly formed by the ectopterygoid [Anderson et al., 2008a] – a bone caudates lack.)

We have not directly represented VOM 5, "Vomer excluded from (0) or contributing to (1) interpterygoid vacuities", although this distinction could be added to the present character as a split of state 2: it clearly depends at least in part on the width of the skull or that of the interpterygoid vacuities; if *Scincosaurus* or *Bruktererpeton* had wider skulls or interpterygoid vacuities, their vomers would end up forming the rostral margins of the interpterygoid vacuities, unless a neomorphic medial process appeared on the pterygoids. The width of the interpterygoid vacuities is coded here as INT VAC 2 and INT VAC 4.

Ventastega has state 0 (Ahlberg, Lukševičs & Lebedev, 1994).

Ichthyostega almost, but not quite, reaches state 0; it has state 1 (Jarvik, 1996: pl. 26–28; D. M., pers. obs. of TMM 41224-2 = AMNH 23100 = MCZ 3361, a total of five casts of MGUH VP 6055, which is the specimen figured in Jarvik, 1996: pl. 26:1).

Whatcheeria has state 1 (Lombard & Bolt, 1995).

Clack (2001: fig. 8) tentatively reconstructed state 1 for *Eucritta* under the assumption that the medial margin of the pterygoid (fig. 5) is complete as preserved. In seymouriamorphs, the palate closes in ontogeny (see below), so it is possible that state 0 – which was scored in RC07 – would have arisen later in *Eucritta*; state 0 is indeed seen in *Megalocephalus*, though *Baphetes* retains state 1. We have assigned state 0 or 1 to *Eucritta*.

States 2 and 3 are both found in *Trimerorhachis* (Milner & Schoch, 2013).

McGowan (2002: 9) limited the possibilities for Albanerpetidae to state 1, 2, or 3. Although McGowan's figures are coarse, his assessment is consistent with Maddin et al. (2013a: fig. 5H, I).

Gephyrostegus has state 1 (Klembara et al., 2014).

In seymouriamorphs the palate closes during ontogeny. Therefore we have assigned state 0 to *Discosauriscus* (Klembara, 1997: fig. 28, not fig. 24 or fig. 34; Klembara et al., 2006: fig. 5B), and state 0 or 1 to *Kotlassia* (instead of just the observed state 1: Bulanov, 2003: fig. 30) and also to *Leptoropha* (the pterygoid of which, not coded by RC07, is known: Bulanov, 2003: S33). *Ariekanerpeton*, *Microphon* and *Utegenia* were all given state 0, 1 or 2 in RC07; given that only state 1 is observed in *Ariekanerpeton* (Klembara & Ruta, 2005a: 64, fig. 3B, 13A), *Microphon* (Bulanov, 2003: S40, S49) and apparently *Utegenia* (Klembara & Ruta, 2004a: fig. 11A, 14A), we have restricted all three to state 0 or 1 as well.

Diadectes shows state 0 in some American species (Olson, 1947), state 1 or 2 in others (Case & Williston, 1912), and state 1 in *D. absitus* (Berman, Sumida & Martens, 1998). We have scored it as possessing states 0 and 1 because making partial uncertainty part of a polymorphism is not possible.

Limnoscelis has state 1 (Berman, Reisz & Scott, 2010).

It seems a safe assumption that *Westlothiana* possessed palatines (Smithson et al., 1994). We have kept state 1 or 2, though whether 0 and 3 can really be excluded might be arguable.

State 2 seems most likely in *Batropetes* (Glienke, 2013, 2015).

State 1 is found in *Microbrachis* (Vallin & Laurin, 2004; Olori, 2015) and *Hyloplesion* (Olori, 2015).

As reconstructed by Wellstead (1991) and Pardo & Anderson (2016), *Brachydectes* has a unique condition with an extremely broad parasphenoid that makes interpterygoid vacuities impossible. Regardless, it has state 2: there is a pterygoid/vomer contact rather than a palatine/parasphenoid contact, excluding states 3 and higher, and the pterygoids do not come anywhere near each other (being separated by the parasphenoid), excluding states 1 and 0.

The reconstruction and the specimen drawing of *Adelospondylus* (Andrews & Carroll, 1991) are not very similar to each other. We have added state 0 to the uncertainty of states 1, 2 or 3 scored in RC07.

Diceratosaurus has state 1 (D. M., pers. obs. of CM 26231; compatible with the slightly disarticulated MB.Am.778, which was reconstructed as just reaching state 2 by Jaekel, 1903: pl. III).

Lethiscus possesses state 1 or 2 (Pardo et al., 2017); *Oestocephalus* appears to have state 0, 1 or 2 (Carroll, 1998a: 153, fig. 2, 3).

In *Phlegethontia*, the vomers and the palatines are absent. Technically, only states 0 and 5 could therefore be excluded; however, given the length (rostral/mesial extent) of the "palatoquadrates" (Anderson, 2002, 2007a), we have scored state 1 or 2, because a palatine would have to be in a highly unusual position rostral to the "palatoquadrates".

Palatines (at least as separate bones) are likely also absent in *Vieraella* (Báez & Basso, 1996); given again the length of the pterygoids, states 0, 1, 2 and 5 can be excluded, so we have scored state 3 or 4. The same applies to **Chelotriton*, except that its pterygoid is so short (close to state 5, without reaching it: Roček & Wuttke 2010; Schoch, Poschmann & Kupfer, 2015) that we have also excluded state 3, leaving state 4 for this OTU.

Ossinodus has state 0 or 1 (Warren 2007).

States 3 and 4 both occur in **Micropholis* (Schoch & Rubidge, 2005: fig. 2).

We have included the condition of **Karpinskiosaurus* (Klembara, 2011: fig. 3C) in state 0.

The CT scans of **Carrolla* (only known specimen: TMM 40031-54) by Maddin, Olori & Anderson (2011) seem to only distinguish finished bone surfaces from everything else, and not to distinguish spongy bone from the matrix. Thus, fig. 4F of Maddin, Olori & Anderson (2011) fails to show some bone that is clearly present – and also misses what must be the suture between vomer and pterygoid (D. M., pers. obs. of TMM 40031-54). The lateral elements of the palate have been pushed dorsally against the skull roof, breaking the pterygoid and rotating the palatine, but D. M. confirms the reconstruction of the palate by Maddin, Olori & Anderson (2011: fig. 7B) in this respect (see below for ch. 119, PTE 3-9, however). **Carrolla* thus has state 2. It is, however, noteworthy how thin (labiolingually) the mesial process of the pterygoid is (probably shared with *Batropetes*: Glienke, 2013, 2015).

Fig. 14b of Werneburg (2012a) suggests state 3 for **Mordex*, although fig. 14a (which does not show any sutures in the articulated palate of the smallest specimen) looks like state 2 unless we assume that the palatine is split lengthwise and the lateral part alone is disarticulated. We have made this assumption and scored state 3.

Following the matrix by Pardo et al. (2017), we have scored state 1 or 2 for **Coloraderpeton*.

106. VOM 7: **Vomer/maxilla suture anterior to the choana: absent (0); present (1).**

Whatcheeria (the premaxilla participates in the margin of the choana: Lombard & Bolt, 1995), *Cochleosaurus* (Sequeira, 2004), *Trimerorhachis* (Milner & Schoch, 2013), *Acheloma* (Polley & Reisz, 2011) and *Batropetes* (Glienke, 2013, 2015) have state 0. We have also scored state 0 for *Silvanerpeton* because the reconstruction by Ruta & Clack (2006: fig. 8) seems to make state 1 impossible.

State 1 is present in *Ventastega* (Ahlberg, Lukševičs & Lebedev, 1994), *Acanthostega* (Porro, Rayfield & Clack, 2015: fig. 4C), *Balanerpeton* (Milner & Sequeira, 1994: 338), Dendrerpetidae (*Dendrerpeton acadianum*: Milner, 1996: fig. 6; in *Dendrysekos* the condition is unknown, but state 1 may well have been just barely present as in *Balanerpeton*: Holmes, Carroll & Reisz, 1998: fig. 4), apparently *Diceratosaurus* (D. M., pers. obs. of MB.Am.778), most likely *Lethiscus* (Pardo et al., 2017) and *Ossinodus* (Warren, 2007).

Phonerpeton is apparently polymorphic: the previously scored state 0 was reconstructed by Dilkes (1990: fig. 1), but is almost certainly excluded by Dilkes (1993: fig. 5) and by AMNH 7150 (D. M., pers. obs.). Dilkes (1993: fig. 4) also figured a clear case of state 0 in a specimen that cannot be referred to *Phonerpeton* with sufficient certainty (Dilkes, 1993).

The condition in *Platyrhinops* is unknown; it is possible that the palatine contacts the vomer lateral to the choana (Clack & Milner, 2010), and if so, the position of the suture between palatine and vomer lateral to the choana is unknown with respect to the maxilla (Clack & Milner, 2010).

Unknown also in **Saharastega* due to the problem of identifying the nasal passage (D. M., pers. obs. of MNN MOR 73).

Schoch & Witzmann (2009b) scored both species of **Glanochthon* as having a premaxillary contribution to the margin of the choana (state 0 of their ch. 32), which would mean state 0 of the present character. However, this contradicts their fig. 3D, which unambiguously shows state 1 in **G. angusta*. Figure 3E, for **G. latirostris*, does not show the maxilla/premaxilla suture, but if it was in about the same place as in **G. angusta*, state 1 would again result. We have scored state 1.

107. VOM 8: **Vomer with (0) or without (1) lateral (parachoanal) toothrow.** RC07 specified a “toothed lateral crest” instead of a toothrow, but, firstly, the presence of the parachoanal toothrow – not otherwise represented in this matrix – is much easier to ascertain in published line drawings than the presence of a crest, and secondly, the alveoli of toothrows are usually (among taxa in this matrix) attached to a low but sharp labial crest, so that a toothrow without a crest is unlikely to occur. Incidentally, a crest without a toothrow is documented in *Cochleosaurus* (Sequeira, 2004), *Phonerpeton* (Dilkes, 1990: fig. 1; D. M., pers. obs. of MCZ 1485) and *Eryops* (D. M., pers. obs. of MCZ 1129 and MCZ 2766), all of which were scored 1 – we conclude that RC07 did not consider a crest covered with denticles “toothed”, and have kept these scores.

We count oblique toothrows (oblique relative to the choanae) as VOM 9(0), not VOM 8(0); see VOM 9. The OTUs concerned were already scored 1 or unknown for VOM 8.

State 0 is present in *Ventastega* (Ahlberg, Lukševičs & Lebedev, 1994), *Whatcheeria* (Lombard & Bolt, 1995), *Micromelerpeton* (Boy, 1995), *Rhynchonkos* (CG78: fig. 63; Szostakiwskyj, Pardo & Anderson, 2015), *Cardiocephalus* (CG78), *Lethiscus* (Pardo et al., 2017), *Microphon* (Bulanov, 2003, 2014), *Ossinodus* (Warren, 2007) and **Karpinskiosaurus* (Klembara, 2011). We have further followed the matrix of Pardo et al. (2017) in scoring state 0 for **Coloraderpeton*.

Acheloma (Polley & Reisz, 2011), *Eoscopus* (Daly, 1994), *Bruktererpeton* (Boy & Bandel, 1973), *Gephyrostegus* (Klembara et al., 2014), *Batropetes* (Glienke, 2013, 2015) and

Diceratosaurus (at least the broad-snouted morph – in which, however, a suggestive extension of the denticle field is present: D. M., pers. obs. of MB.Am.778; idealized by Jaekel, 1903: pl. III) have state 1. Likewise, the pterygoid toothrows of *Orobates* do not continue onto the vomers (Nyakatura et al., 2015: digital reconstruction)

Trimerorhachis is polymorphic, sometimes between the left and right sides of the same individual (Milner & Schoch, 2013: fig. 3C).

In *Eocaecilia*, a single continuous toothrow extends along the labial margins of both vomers and palatines. Because the choanae lie lingual to it, we have kept the score of 1.

This character and the next are hard to apply to *Brachydectes*: a toothrow spans the length of each vomer, but the teeth are so large and the vomers so narrow that the orientation of the toothrows is wholly dictated by that of the vomers (Pardo & Anderson, 2016). We have no non-phylogenetic arguments for either taking the parachoanal orientation of the toothrows at face value (state 0 of this character) or interpreting it as a salamandrid-like homolog of the interchoanal toothrow (state 0 of the next character), so we have had to score both as unknown.

The vomer is wholly unknown in *Pelodosotis* (CG78: 80–81); the present character is also apparently unknown in *Euryodus* (CG78).

108. VOM 9: **Vomer with (0) or without (1) transverse (interchoanal) toothrow.** RC07 specified "transversely orientated, anterior crest" and did not mention any teeth. Indeed, a toothless and (especially medially) largely rounded crest is present in *Ichthyostega* (Jarvik, 1996: pl. 26:1; Clack & Milner, 2015: fig. 8C; D. M., pers. obs. of TMM 41224-2 = MCZ 3361). A similar condition is found in *Eryops*, where this ridge additionally bears relatively large denticles that continue rostromedially but not caudally (D. M., pers. obs. of AMNH 4673). A sharp ridge is found in *Phonerpeton*, where the entire vomer except for the floor of the rostral palatal vacuity bears scattered denticles (Dilkes, 1990: fig. 1; D. M., pers. obs. of MCZ 1485). Finally, *Cochleosaurus* has several denticle-bearing crests on the vomer, one of which is transverse (Sequeira, 2004). However, all four of these OTUs were scored 1 (which we have kept); we consider this evidence that RC07 actually had a crest bearing a toothrow in mind, as they stated more clearly for VOM 8. For the same reasons as in VOM 8, we have scored the toothrow alone.

Metamorphosed salamanders often have a transverse toothrow at the caudal end of the vomer; some clades instead have an S-shaped or straight toothrow that extends rostromedially to caudolaterally or caudally. These toothrows are homologous to each other and to the toothrows at the labial margin of the vomer seen in larvae and in neotenic adults that have not undergone complete metamorphosis: during metamorphosis, the part of the vomer lingual to the toothrow is destroyed, and the vomer grows on the labial side. For this reason we have scored the toothrows of *Karaurus*, *Valdotriton* (double to triple; see VOM 11), **Beiyanerpeton*, **Pangerpeton* and **Chelotriton* (Schoch, Poschmann & Kupfer, 2015 – Roček & Wuttke, 2010: 323, appear to have assigned them to the inexistent palatine) all as representing state 0. We have done the same with *Eocaecilia*, where a single continuous toothed crest extends along the labial margins of both vomers and palatines.

Because of this movement of the crest during salamander metamorphosis, and because the crest often lies about in the middle of the vomer in temnospondyls, we have scored the toothed crest of the frogs *Notobatrachus* (Báez & Nicoli, 2004), *Vieraella* (teeth unknown, but more ventral than the rest of the vomer: Báez & Basso, 1996) and **Liaobatrachus* (Dong et al., 2013) as constituting state 0 as well; in the latter, notably, the crest lies in the middle of the vomers rather than at their caudal edge (despite a general absence of paedomorphic features). The same applies to *Batrachiderpeton*, *Diplocaulus* and *Diploceraspis* (Williston,

1909; Beerbower, 1963; Bossy & Milner, 1998); *Diceratosaurus* is polymorphic between the broad-snouted and the narrow-snouted morph, see VOM 4.

State 0 is further present in *Ventastega* (Ahlberg, Lukševičs & Lebedev, 1994), *Trimerorhachis* (Milner & Schoch, 2013), *Ptyonius* (Bossy & Milner, 1998) and *Ossinodus* (Warren, 2007). We have also counted the rostralmost two teeth on the vomer of *Lethiscus* (Pardo et al., 2017) as forming a row; the vomer is too narrow for a longer transverse toothrow. Finally, we have assigned state 0 to *Doleserpeton*, see VOM 11.

Acheloma (apparently: Polley & Reisz, 2011), *Eoscopus* (Daly, 1994), *Bruktererpeton* (Boy & Bandel, 1973), *Batropetes* (Glienke, 2013, 2015), *Microphon* (Bulanov, 2003) and *Orobates* (Nyakatura et al., 2015: digital reconstruction) have state 1.

Unknown in *Edops* (D. M., pers. obs. of MCZ 1378); the vomer is wholly unknown in *Pelodosotis* (CG78: 80–81).

State 0 is clearly present in juvenile **Glanochthon angusta* (Schoch & Witzmann, 2009b: fig. 3A), but its further fate in ontogeny is unclear from the description and the illustrations; because fig. 4D might show its persistence in adult **G. latirostris*, we have provisionally scored state 0.

deleted VOM 11

RC07 called this character "**Vomer without (0) or with (1) nearly transverse patch of small teeth (denticles) lying posterome[d]ial to choana.**" They stated that state 1 "is found in *Doleserpeton* and some basal salientians", and scored it for *Doleserpeton*, the salientian *Notobatrachus* and the caudates *Karaurus* and *Valdotriton*. However, this is not defensible:

Except for the pit that bears the fang-replacing toothrow, the entire vomer of *Doleserpeton* is covered by denticles (Sigurdsen & Bolt, 2010; already correctly scored as VOM 4(1)). There is thus no transverse patch, causing us to score state 0.

Conversely, *Notobatrachus* and *Karaurus* lack denticles altogether. In *Karaurus*, the teeth that lie caudomedial to the choana have full size and are part of the transverse vomerine toothrow, far from being a patch of denticles (Ivachnenko, 1978: fig. 1b); those of *Notobatrachus* are smaller than the marginal teeth, but still rather too large for being denticles, and form a straight row borne on a crest on each vomer (Báez & Nicoli, 2004). These teeth constitute VOM 9(0).

Two to three rows of (small) teeth larger than the (tiny) marginal teeth are found on each otherwise toothless vomer of *Valdotriton*, and they lie rostromedial to the choana, which is not separate from the interpterygoid vacuity (Evans & Milner, 1996). This, too, constitutes state 0, even if the position of this double or triple toothrow would have changed in further ontogeny, corresponding as it does to what is seen halfway through metamorphosis in extant salamanders. While the toothrow shares the fact of not being single with a patch of denticles, denticles are defined in other characters of the present matrix as being much smaller than marginal teeth.

Among the OTUs scored as unknown by RC07, state 0 is now known in *Ventastega*, *Whatcheeria*, *Acheloma*, *Bruktererpeton* and *Ossinodus* (see VOM 4) as well as *Gephyrostegus* (Klembara et al., 2014) and *Capetus* (Sequeira & Milner, 1993).

State 0 was scored in RC07, but is in fact unknown, in *Neldasaurus*, for which Chase (1965: 176) specified: "The obstinate character of the matrix left no indication of palatal teeth in areas other than those described […], but the fragments of the pterygoid bones […] show that […] [parts of the pterygoid] are covered by a densely-packed shagreen of small teeth except at the medial edge of the bone." Denticles are not mentioned anywhere else in the paper. We conclude that their presence, let alone arrangement, is unknown on the vomer – as

already scored for the palatine (PAL 2) and the ectopterygoid (ECT 3). The state is also unknown in *Hapsidopareion* (CG78).

The vomers of *Archeria* and *Pelodosotis*, also originally scored 0, are entirely unknown (CG78: 80–81; Holmes, 1989).

In other words, once denticles are specified, state 1 **does not occur in this matrix at all** – except in **Gerobatrachus* (Anderson et al., 2008a). In the wording of 2007, then, this character is parsimony-uninformative – quite apart from the issue of correlation with VOM 4.

Ruta, Coates & Quicke (2003), however, did not specify denticles, and cited Bolt (1969, 1977, 1979, 1991) as support for their statement that state 1 is shared between *Doleserpeton* and various unspecified lissamphibians. This must refer to Bolt's hypothesis that the short rows of full-sized (not "small"!) teeth found on the vomer, palatine and dentary of *Doleserpeton* in the positions (indeed in recognizable pits) where other temnospondyls bear fangs are homologous to lissamphibian toothrows. While we are skeptical about this idea, we have deleted the present character but scored *Doleserpeton* as having state VOM 9(0) to avoid bias against the TH.

109. VOM 12: **Distinct posterolateral process of vomer bordering more than half of choana posterior margin: absent (0); present (1).**

State 0 is documented in *Ventastega* (Ahlberg, Lukševičs & Lebedev, 1994), *Acheloma* (Polley & Reisz, 2011), *Bruktererpeton* (Boy & Bandel, 1973), *Diceratosaurus* (D. M., pers. obs. of MB.Am.778), *Lethiscus* (Pardo et al., 2017) and *Ossinodus* (Warren, 2007). Breaks in MNN MOR 73 (D. M., pers. obs.) support the reconstruction of state 0 in **Saharastega* by Damiani et al. (2006).

State 1 must be scored for *Limnoscelis* (Reisz, 2007; Berman, Reisz & Scott, 2010). Rather than a caudal margin distinct from the medial one, the choana has a caudolateral point where the lateral edge of the vomer continues into a caudolateral process that extends all the way to the medial edge of the maxilla, excluding the palatine from the margin of the choana.

State 1 is further present in *Acanthostega* (Porro, Rayfield & Clack, 2015) and in *Batropetes* as reconstructed by Glienke (2013).

Unknown in *Scincosaurus* (Milner & Ruta, 2009); the vomer is wholly unknown in *Pelodosotis* (CG78: 80–81).

110. PAL 1: **Palatine with (0) or without (1) fangs comparable in size to or larger than marginal teeth (premaxillary or maxillary).**

Jarvik (1996) reconstructed *Ichthyostega* with state 1. Ahlberg, Lukševičs & Lebedev (1994) demonstrated that it, as well as *Ventastega*, had state 0 instead. For *Ichthyostega*, this is confirmed by Jarvik (1996: pl. 26:1) and TMM 41224-2 = AMNH 23100 = MCZ 3361 (D. M., pers. obs.; five casts of the figured specimen MGUH VP 6055). Clack & Milner (2015) mentioned this issue and provided further evidence for state 0 in *Ichthyostega*, but did not make clear if specimens showing state 1 are known; for the time being, we have scored state 0.

The most mature known individual of *Amphibamus* – the neotype, YPM 794 – has state 0 (Daly, 1994: 27). We regard this as the adult condition (see also Marjanović & Laurin 2008: 180). Schoch & Milner (2014: 56) stated that *Amphibamus* lacks fangs on the vomer, palatine and ectopterygoid, but puzzlingly did not mention Daly (1994) in the context of *Amphibamus* at all, even though they of course mentioned YPM 794 and cited Daly (1994) as a source for *Eoscopus*.

State 0 is also found in *Lethiscus* (Pardo et al., 2017), *Ossinodus* (Warren, 2007) and *Silvanerpeton* (Ruta & Clack, 2006).

Batropetes (Glienke, 2013), *Diceratosaurus* (Jaekel, 1903; D. M., pers. obs. of MB.Am.778), *Orobates* (Nyakatura et al., 2015: digital reconstruction) and *Tseajaia* (Moss, 1972: 12) have state 1.

This character is inapplicable to *Notobatrachus*, which lacks (separate) palatines (PAL 8(1)). It also has to be scored as unknown for *Vieraella*, where the presence of palatines is unknown.

111. PAL 2: **Palatine without (0) or with (1) small teeth (denticles), the basal diameter and/or height of which is less than 30% of that of adjacent marginal teeth (maxillary) and remaining palatine teeth (if present).** See VOM 4; RC07 even wrote "remaining vomer teeth" instead of "remaining palatine teeth".

Ventastega (Ahlberg, Lukševičs & Lebedev, 1994), *Whatcheeria* (Lombard & Bolt, 1995), *Batropetes* (Glienke, 2013), apparently *Oestocephalus* (Carroll, 1998a), *Orobates* (Berman et al., 2004) and *Tseajaia* (Moss, 1972: 12) have state 0. Although this is not obvious from Maddin, Olori & Anderson (2011), so does **Carrolla* (D. M., pers. obs. of TMM 40031-54). We further follow the matrix of Pardo et al. (2017) in scoring state 0 for **Coloraderpeton*.

Acheloma (Polley & Reisz, 2011), *Broiliellus* (Carroll, 1964), *Platyrhinops* (Clack & Milner, 2010), *Limnoscelis* (Fracasso, 1983: 121) and *Diceratosaurus* (at least the broad-snouted morph: Jaekel, 1903; D. M., pers. obs. of MB.Am.778) possess state 1, as does **Nigerpeton* (D. M., pers. obs. of MNN MOR 69). The smallest teeth on the palatines of *Pantylus* (Romer, 1969; CG78) and *Stegotretus* (Berman, Eberth & Brinkman, 1988) qualify for state 1 as well.

While CG78 did not mention or illustrate denticles for *Hapsidopareion*, this may be due to preservation or preparation issues; we have scored it as unknown.

Unknown in *Edops* (D. M., pers. obs. of MCZ 1378). Probably also unknown in *Kotlassia*, judging from the comments on the preservation of the toothrow and on the pterygoid and parasphenoid (Bulanov, 2003: S55).

The dots in the reconstruction of **Neopteroplax* (Romer, 1963: fig. 5) appear to represent denticles, but there is no evidence for them in the specimen drawing (fig. 2) or the text; instead, in the specimen drawing, the denticle field has a very sharp edge that appears to coincide with the suture between pterygoid and palatine. We have therefore scored state 0.

112. PAL 4: **Palatine with (0) or without (1) row of teeth (3+) comparable in size to, or greater than, marginal teeth (maxillary) and parallel to these.**

Ventastega (Ahlberg, Lukševičs & Lebedev, 1994), *Microphon* (Bulanov, 2014) and *Ossinodus* (Warren, 2007) have state 0.

Phonerpeton (D. M., pers. obs. of AMNH 7150), *Ecolsonia* (D. M., pers. obs. of CM 38017), *Brukterpeton* (Boy & Bandel, 1973: 51), *Batropetes* (Glienke, 2013), *Diceratosaurus* (at least the broad-snouted morph: D. M., pers. obs. of MB.Am.778) and *Tseajaia* (Reisz, 2007) show state 1.

Unknown in *Asaphestera* (CG78: 19). We follow the matrix of Pardo et al. (2017) in keeping the same score for *Lethiscus*, where both vomers are damaged labially (Pardo et al., 2017: fig. 1, ext. data fig. 1).

**Micropholis* is polymorphic (Schoch & Rubidge, 2005), as (apparently) is **Sclerocephalus* (Schoch & Witzmann, 2009a).

Orobates has state 1 as originally scored (Nyakatura et al., 2015: digital reconstruction); however, this is impossible to see in Berman et al. (2004).

deleted PAL 6: **Palatine articulates with maxilla only at anterior extremity of the former: absent (0); present (1).** State 1 was scored in RC07 only for branchiosaurids and *Petrolacosaurus*. However, Werneburg (2012a: 43–44) reports that it is only known from larvae in the branchiosaurid *Apateon* – the skeletally most mature individuals of *A. dracyi* (neotenic), *A. caducus* (neotenic) and *A. gracilis* (metamorphosed) have state 0. Conversely, state 1 is present in larval but not metamorphosed *Amphibamus* (Milner, 1982; Werneburg, 2012a: 44) and *Platyrhinops* (Werneburg, 2012a). It stands to reason that the observed state 1 in *Leptorophus* and *Schoenfelderpeton* (and **Tungussogyrinus*) is likewise larval or paedomorphic. This leaves state 1 to *Petrolacosaurus* alone (because of its suborbital fenestra), making this character parsimony-uninformative, so we have deleted it. We could instead have specified that state 1 only occurs in larvae – but the ontogeny of way too many taxa in this matrix is insufficiently known.

Ventastega shows state 0 (Ahlberg, Lukševičs & Lebedev, 1994), as do *Brukterer-peton* (Boy & Bandel, 1973: 51), *Lethiscus* (Anderson, Carroll & Rowe, 2003; Pardo et al., 2017) and *Ossinodus* (Warren, 2007).

Unknown in *Adelogyrinus* (Andrews & Carroll, 1991) and *Orobates* (Berman et al., 2004); inapplicable to *Phlegethontia*, which lacks palatines (Anderson, 2002).

113. PAL 7: **Palatines very wide, almost meeting in the midline (0); unremarkable (1); very narrow in any dimension (2) (ordered).** State 0 of this potentially continuous character is new and accounts for the highly unusual condition of *Eoherpeton* (Smithson, 1985), *Bruktererpeton* (Boy & Bandel, 1973), *Gephyrostegus* (Klembara et al., 2014; Carroll, 1970, even reconstructed the palatines as meeting in one specimen), *Pantylus* (Romer, 1969; CG78), *Stegotretus* (Berman, Eberth & Brinkman, 1988) and **Sparodus* (Carroll, 1988; D. M., pers. obs. of NHMW 1899/0003/0006). It does not seem to occur anywhere else in or close to our taxon sample, and there are no borderline cases we are aware of; the closest is *Crassigyrinus*, in which the palatines are roughly L-shaped and not much longer than wide, but nonetheless stay well away from the midline (Clack, 1998: fig. 4B). The distinction between states 1 and 2 (originally 0 and 1), however, is harder to define; most likely, the coding remains rather subjective – the original wording (RC07: 100) is "Palatine shaped like a slender, strut-like bone: absent (0); present (1)."

State 1 is in any case present in *Ventastega* (Ahlberg et al., 2008), *Micromelerpeton* (Boy, 1995; Schoch, 2009b: fig. 2c), *Apateon* (*A. pedestris*: Schoch & Milner, 2008; *A. caducus*: Fröbisch & Schoch, 2009b), arguably *Leptorophus* and *Schoenfelderpeton* (Boy, 1986), probably *Batropetes* (Glienke, 2013), *Lethiscus* (Anderson, Carroll & Rowe, 2003; Pardo et al., 2017) and *Ossinodus* (Warren, 2007).

State 2 makes a surprise appearance in *Acanthostega* (Porro, Rayfield & Clack, 2015).

In RC07, our state 1 was scored for *Amphibamus*. This is supported by Schoch (2001) and Schoch & Milner (2014: fig. 30B); but the individual drawn by Schoch (2002b) is not adult, because it lacks the palatal fangs noted by Daly (1994: 27) in the most mature known specimen (see VOM 7, PAL 1, ECT 2), and so does the composite reconstruction by Schoch & Milner (2014). Considering Schoch's (2002b) reconstruction of the palate of *Platyrhinops* (though see Clack & Milner, 2010: fig. 9b) and the condition seen in **Iberospondylus* (Laurin & Soler-Gijón, 2006), the shape of the palatine may depend on the presence of fangs; Daly (1994) did not illustrate it, so we have scored *Amphibamus* as unknown.

Also unknown in *Archeria* (Holmes, 1989).

We have scored state 1 or 2 for *Diceratosaurus* (D. M., pers. obs. of the crushed MB.Am.778) and for *Brachydectes*, whose palatines are arguably too short for these states to be distinguishable (Pardo & Anderson, 2016: fig. 10).

Saharastega is best scored as having state 0 or 1 (D. M., pers. obs. of MNN MOR 73).

114. PAL 8: **Separately ossified palatine: present (0); absent (1).**

Ventastega (Ahlberg, Lukševičs & Lebedev, 1994), *Batropetes* (Glienke, 2013), *Lethiscus* (Anderson, Carroll & Rowe, 2003; Pardo et al., 2017) and *Ossinodus* (Warren, 2007) have state 0.

Unknown in *Archeria* (Holmes, 1989) and *Vieraella* (Báez & Basso, 1996), apparently also in *Keraterpeton* (Huxley & Wright, 1867; Jaekel, 1903).

We accept the interpretation of Gao & Shubin (2012) and Wang, Dong & Evans (2015: 52) that a palatine is present (state 0) in the crown-group salamander **Beiyanerpeton*. A palatine is also present in another Jurassic crown-group salamander, ***Chunerpeton* (unmarked and unmentioned in Gao & Shubin, 2003: fig. 1; mentioned by Wang, Dong & Evans, 2015: 52), and has recently been reported for a third one (***Qinglongtriton*: Jia & Gao, 2016). It follows that we agree with Schoch (2014b) in rejecting Schoch's earlier (1998) interpretation that the palatine is absent even in larval salamanders in which the "palatopterygoid", homologous only to the pterygoid in that view, contacts the vomer.

115. ECT 1-4: **Ectopterygoid at least as long as palatine (0); at least about a third as long as but shorter than palatine (1); at most about a third as long as palatine (2); absent (3) (ordered).** ECT 1 concerned the presence (state 0) of an ectopterygoid, ECT 4 its size ("longer than/as long as (0) or shorter than (1) palatine"). We have merged both of them with ch. 8 of McGowan (2002) as revised by Marjanović & Laurin (2008): "Ectopterygoid at least about half as long as palatine (0), about a third as long as the palatine or shorter (1), or absent (2) (ordered)" to extract the maximum of phylogenetic signal while avoiding character correlation. The gap between states 0 and 1 of Marjanović & Laurin (2008) was an artefact of the small taxon sample; we now count this part of morphospace as part of our state 1.

Unambiguous occurrences of state 2 are limited to *Schoenfelderpeton* and **Gerobatrachus*; *Doleserpeton* (Sigurdsen & Bolt, 2010), *Triadobatrachus* (Ascarrunz et al., 2016: fig. 4, 3D model 1), *Eocaecilia* (Jenkins, Walsh & Carroll, 2007; see Marjanović & Laurin, 2008: 181, for discussion of ectopterygoids in gymnophionomorphs) and *Diploceraspis* (Beerbower, 1963) have state 2 or 3. We have scored **Quasicaecilia* the same way (Pardo, Szostakiwskyj & Anderson, 2015). **Carrolla* has small, rounded ectopterygoids like taxa with state 2, but the palatine is itself so short that we have scored **Carrolla* as possessing state 1.

Whatcheeria was scored as 0 for ECT 1 but unknown for ECT 4 in RC07. Lombard & Bolt (1995) did not mention the size of the ectopterygoid, technically excluding only state 3 of the present character; however, they mentioned its dentition which make states 0 and 1 probable, and would likely have mentioned if the ectopterygoid was unusually short. We have therefore scored state 0 or 1.

Isodectes was scored the same way, but there is not enough space for our state 0 in the reconstruction by Sequeira (1998: fig. 9), while the presence of the ectopterygoid is uncertain (Sequeira, 1998); we have scored state 1, 2 or 3.

We have accepted the score of 0 for Dendrerpetidae based on Godfrey, Fiorillo & Carroll (1987), judging from the text of which the reconstruction of the palate is probably justified even though the specimen drawing (fig. 1) only show the mere presence of the ectopterygoid. Other sources can only narrow the condition down to state 0 or 1 (Milner, 1996: 90; Holmes, Carroll & Reisz, 1998). Similar considerations hold for the other ECT characters.

Acheloma (Polley & Reisz, 2011), *Anthracosaurus* (Panchen, 1977) and *Tseajaia* (which has an unusually small palatine: Moss, 1972: 12) have state 0.

Ecolsonia has state 0 or 1 (Berman, Reisz & Eberth, 1985: fig. 6).

Broiliellus has state 1 according to Schoch (2012: fig. 2F).

We assign state 1, 2 or 3 to *Batropetes* based on Glienke (2013: fig. 4A).

Bruktererpeton has state 1 or 2; while the part of the palate that contains the suture between the ectopterygoid and the palatine is missing, enough of both bones appears to be known that the other states are impossible. The reconstruction (Boy & Bandel, 1973: fig. 7) further indicates that state 2 can be ruled out as well, but the text (p. 51) contradicts this by saying that only the caudal margin of the ectopterygoid is preserved.

Seymouria sanjuanensis has state 1 as originally scored (Klembara et al., 2005), but *S. baylorensis* has state 0 (Laurin, 2000); *Seymouria* is thus polymorphic.

Contra Marjanović & Laurin (2008: 181), *Saxonerpeton* (though borderline, if the ectopterygoid is correctly identified) and *Hapsidopareion* most likely have state 1 (CG78: figs. 14E, 21; already scored in RC07 for *Hapsidopareion*).

Olori (2015) stated that the palatine of *Hyloplesion* was shorter than reconstructed by CG78; we have therefore scored state 1 or 2 (rather than only 2).

Diplocaulus shows state 3 (Bossy & Milner, 1998), as do *Sauropleura* (Bossy & Milner, 1998), *Oestocephalus* (Carroll, 1998a) and *Phlegethontia* (Anderson, 2002).

Bossy (1976) and Bossy & Milner (1998) reconstructed a suture on the palate of *Ptyonius* that would separate the vomer from the palatine in an odd place (quite some distance caudal to the choana). Therefore, they considered the next suture (even farther caudal to the choana) to be the one between palatine and ectopterygoid. That bone, however, looks exactly like the palatine of the closely related *Sauropleura* (and several other "nectrideans") in their own reconstructions. Given the state of preservation of *Ptyonius* (Bossy, 1976), we prefer not to take the reconstruction at face value and have scored *Ptyonius* as unknown. It should be mentioned that ectopterygoids are otherwise absent in nectrideans (Bossy, 1976; Bossy & Milner, 1998) – Jaekel (1903: pl. III) reconstructed a suture between the palatine and the "transversum" (ectopterygoid) in *Diceratosaurus*, but we agree with RC07 in scoring *Diceratosaurus* as unknown because is hard to tell if MB.Am.778, the specimen on which the reconstruction is based, really possesses such a suture (D. M., pers. obs.).

Lethiscus has state 1 (Pardo et al., 2017, excluding their matrix, where state 0 is scored).

States 0 and 1 probably both occur in **Micropholis* (Schoch & Rubidge, 2005: fig. 2).

Although the suture between the palatine and the ectopterygoid cannot be found in **Ymeria*, the minimum length that includes all ectopterygoid teeth is longer than the length that remains for the palatine (Clack et al., 2012a: fig. 2), giving state 0 to **Ymeria*.

Werneburg (2012a: 12) stated that an ectopterygoid is not certainly preserved in **Branchiosaurus* but may be present in fig. 6c, d. The bone in question is indeed most likely an ectopterygoid. Unfortunately, it may not be complete, and the length of the palatine is unknown, so we have scored state 0, 1 or 2.

We have followed the matrix of Pardo et al. (2017) in scoring state 0 for **Coloraderpeton*; the ectopterygoid is present (J. Pardo, pers. comm.), it is scored as reaching the subtemporal fenestra, and the space where it would lie is so long that state 0 seems inevitable.

116. ECT 2: **Ectopterygoid with (0) or without (1) fangs comparable in size to or larger than marginal teeth (premaxillary or maxillary) and remaining ectopterygoid teeth (if present).**

Acanthostega (Clack, 1994a; Porro, Rayfield & Clack, 2015) and *Acheloma* (Polley & Reisz, 2011) show state 0, as does *Phonerpeton* (D. M., pers. obs. of MCZ 1485). We tentatively assign the same score to *Trimerorhachis* (Milner & Schoch, 2013); perhaps polymorphism would be better (Milner & Schoch, 2013: fig. 1, 5).

The most mature known individual of *Amphibamus* – the neotype, YPM 794 – has state 0 (Daly, 1994: 27) as already scored in RC07. We regard this as the adult condition (see also Marjanović & Laurin, 2008: 180). Schoch & Milner (2014: 56) stated that *Amphibamus* lacks fangs on the vomer, palatine and ectopterygoid, but puzzlingly did not mention Daly (1994) in the context of *Amphibamus* at all, even though they of course mentioned YPM 794 and cited Daly (1994) as a source for *Eoscopus*.

Lethiscus (Pardo et al., 2017) and *Tseajaia* (Moss, 1972: 12) have state 1. We have followed the matrix of Pardo et al. (2017) in scoring it for **Coloraderpeton* as well.

Unknown in *Bruktererpeton* (Boy & Bandel, 1973: 51).

Ichthyostega (Jarvik, 1996: pl. 26:1, 27:1; Blom, 2005; Clack et al., 2012a; Clack & Milner, 2015: fig. 8C) and **Lydekkerina* (Shishkin, Rubidge & Kitching, 1996: fig. 7) are polymorphic.

Orobates has state 1 as originally scored (Nyakatura et al., 2015: digital reconstruction); however, this is impossible to see in Berman et al. (2004).

We interpret the name of this character as saying that ectopterygoid fangs have to be larger than the remaining ectopterygoid teeth; therefore **Glanochthon* is polymorphic (Schoch & Witzmann, 2009b: figs. 3, 4) and **Australerpeton* has state 1 (Eltink et al., 2016: 848, fig. 6). **Platyoposaurus*, while borderline, has state 0, however (Gubin, 1991: drawing 3б); and we concur with Pereira Pacheco et al. (2016: appendix 2) in counting the borderline condition of **Konzhukovia* (Gubin, 1991: drawing 15a) as state 0 as well.

117. ECT 3: **Ectopterygoid without (0) or with (1) small teeth (denticles), the basal diameter and/or height of which is less than 30% of that of adjacent marginal teeth (maxillary) and remaining ectopterygoid teeth (if present).** See VOM 4.

Tseajaia shows state 0 (Moss, 1972: 12).

State 1 occurs in *Acheloma* (Polley & Reisz, 2011) and *Platyrhinops* (Clack & Milner, 2010).

Probably unknown in *Kotlassia*, judging from the comments on the preservation of the toothrow and on the pterygoid and parasphenoid (Bulanov, 2003: S55).

The ectopterygoid is too eroded to tell in the two **Nigerpeton* specimens, MNN MOR 69 and MNN MOR 70, that preserve it (D. M., pers. obs.).

Although this is not obvious from Maddin, Olori & Anderson (2011), **Carrolla* has state 0 (D. M., pers. obs. of TMM 40031-54). We have followed the matrix of Pardo et al. (2017) in scoring it for **Coloraderpeton* as well.

The dots in the reconstruction of **Neopteroplax* (Romer, 1963: fig. 5) appear to represent denticles, but there is no evidence for them in the specimen drawing (fig. 2) or the text; instead, in the specimen drawing, the denticle field has a very sharp edge that appears to coincide with the suture between pterygoid and ectopterygoid. We have therefore scored state 0.

118. ECT 5: **Ectopterygoid with (0) or without (1) row of teeth (3+) comparable in size to, or greater than marginal teeth (maxillary) and parallel to these.**

State 0 is found in *Greererpeton* (Smithson, 1982), *Crassigyrinus* (Clack, 1998), *Trimerorhachis* (Milner & Schoch, 2013), *Kotlassia* (Bulanov, 2003: S55, fig. 30), and *Cardiocephalus* (CG78) as well as **Coloraderpeton* (Pardo et al., 2017: matrix; J. Pardo, pers. comm.).

Acheloma (Polley & Reisz, 2011), *Phonerpeton* (D. M., pers. obs. of MCZ 1485), *Ecolsonia* (Berman, Reisz & Eberth, 1985; D. M., pers. obs. of CM 38017), *Gephyrostegus* (Carroll, 1970; Klembara et al., 2014), *Limnoscelis* (Fracasso, 1983; Reisz, 2007), *Microbrachis* (CG87; Vallin & Laurin, 2004; Olori, 2015), *Lethiscus* (Pardo et al., 2017) and *Tseajaia* (Moss, 1972: 12) show state 1.

Unknown in *Saxonerpeton* (CG78).

Orobates has state 1 as originally scored (Nyakatura et al., 2015: digital reconstruction); however, this is impossible to see in Berman et al. (2004).

From comparisons to *Colosteus* and *Greererpeton* (Smithson, 1982; Hook, 1983) and after pers. obs. of MB.Am.1441.2 by D. M., we disagree with Clack et al. (2012b: 22) that "the right ectopterygoid is preserved in such a way that additional teeth would very likely have been evident if present" and have scored the *St. Louis tetrapod as unknown.

deleted ECT 6: **Ectopterygoid/maxilla contact: present (0); absent (1).** State 1, originally scored for the branchiosaurids, *Petrolacosaurus*, *Hyloplesion* and *Odonterpeton*, can mean different things: it can mean that the subtemporal fenestra extends all the way to the middle of the palatine (as it does in larval amphibamids, see above under PAL 6) or at least reaches the caudal end of the palatine (as CG78 reconstructed for *Odonterpeton*); or it can be due to the suborbital fenestra found in diapsids (represented in this matrix by *Petrolacosaurus*), which is separated from the subtemporal fenestra by an ectopterygoid-jugal (as in *Petrolacosaurus*) or ectopterygoid-maxilla contact (as in **crocodyliforms and some **dinosaurs, where the maxilla reaches the caudal margin of the suborbital fenestra); or, at least in theory, a long caudal extension of the palatine could intervene between the ectopterygoid and the maxilla. This last possibility was reconstructed by CG78 (fig. 89) for *Hyloplesion*. It looks, however, seriously weird. We have to wonder if the supposed ectopterygoid is actually the flange of the pterygoid (see PTE 3-9) that has broken off, and the real ectopterygoid is the fragment that lies rostrolateral to it in fig. 89E. (Compare the condition in *Microbrachis*: Vallin & Laurin 2004: fig. 2C, 4B, 5B.) Olori (2015: 44) appeared to agree, stating that "the palatine terminates further anteriorly than was depicted by […] [CG78] in their figure 89H, and thus is shorter than the maxilla in anteroposterior length." (The ectopterygoid is not otherwise mentioned or indicated in a figure.) Most likely, then, state 1 is limited to *Petrolacosaurus* and *Odonterpeton*, and the primary homology of their conditions is wide open to question. Should they be assigned separate states, the character would be parsimony-uninformative.

In *Odonterpeton*, too, the situation is far from clear. First of all, if the reconstruction (CG78: fig. 99B) is correct, it may be due to skeletal immaturity, a condition that would be unsurprising in this tiny specimen, in which case it should be scored as unknown (see PAL 6 above). The specimen drawings (CG78: fig. 98A, 99A), however, do not clearly support the reconstruction. In ventral view (right part of fig. 98A and 99A) all but the medial edge of the ectopterygoid – or perhaps the caudomedial edge of the palatine – is covered by the lower jaw on one side, and nothing is exposed on the other. The description (CG78: 145) reads in full: "The ectopterygoids are small bones. The lateral margin of the left ectopterygoid, seen in dorsal view through the orbit, does not appear to reach the maxilla." It is not clear to us how this conclusion was reached; according to the specimen drawing in dorsal view (CG78: left part of fig. 98A and 99A), the lateral tip of the ectopterygoid lines up with the lateral margin of the palatine, or nearly so, and the disarticulated maxilla is only preserved in lateral view. Finally, not all of the sutures in these specimen drawings are accurate (see POSPAR 1-2 above).

Scoring *Hyloplesion* or *Odonterpeton* as anything other than unknown, thus, will have to await further inspection of specimens. (Unfortunately, D. M. had not yet considered this

character during his visit to the USNM.) This leaves state 1 only to *Petrolacosaurus*, making the character parsimony-uninformative; we have deleted it.

Acheloma (Polley & Reisz, 2011) and *Tseajaia* (Moss, 1972: 12), previously scored as unknown, show state 0.

deleted ECT 7: **Ectopterygoid narrowly wedged between palatine and pterygoid: no (0); yes (1).** RC07 explicitly ascribed state 1 to *Odonterpeton* and *Hyloplesion*, but *Odonterpeton* is reconstructed as having state 0 (CG78: fig. 99). This makes this character parsimony-uninformative (state 1 was not scored for any other OTUs, and we have not seen it in any of them), so we have deleted it. Additionally, we doubt whether state 1 occurs even in *Hyloplesion*, see ECT 6.

119. PTE 3-9: **Flange on pterygoid: absent, pterygoid margin of subtemporal fenestra concave or straight throughout (0); rostrolateral-caudomedial orientation (1); mediolateral or rostromedial-caudolateral orientation ("transverse flange"), without row of large teeth (2); same with row of large teeth (3) (ordered).** RC07 treated the "posterolateral flange" (PTE 9(1); our state 1) separately from the "transverse flange" (PTE 3(1, 2); our states 2 and 3), but we think the "transverse flange" is only an extreme of a continuum the rest of which is called "posterolateral flange". Indeed, the two flanges never occur together – except that our states 1 and 2 occur in different individuals of **Micropholis* (Schoch & Rubidge, 2005: fig. 2) and apparently **Sclerocephalus* (Schoch & Witzmann, 2009a: fig. 4, 6), which we have both scored as having states 1 and 2.

Interestingly, PTE 3 was called "Transverse flange of pterygoid absent (0), present without transverse tooth row (1), or present and carrying transverse tooth row." – the number "(2)" was omitted. This may of course be a simple typographic error; notably, however, state 2 did not occur in the matrix apart from the partial uncertainty (state 1 or 2) that was scored for *Leptoropha* and *Tseajaia*.

State 0 is present in *Ventastega* (Ahlberg, Lukševičs & Lebedev, 1994). The condition of *Caerorhachis* is borderline (Ruta, Milner & Coates, 2002), but we have kept the score of 0.

Trimerorhachis has a weak case of state 1 (Milner & Schoch, 2013), no weaker than that of *Neldasaurus* (already scored correctly; Chase, 1965). State 1 further appears in *Micromelerpeton* (close to state 2: Schoch, 2009b: fig. 2c), *Apateon* (Schoch & Milner, 2008), *Leptorophus* (Boy, 1986), *Rhynchonkos* (Szostakiwskyj, Pardo & Anderson, 2015: fig. 3A), *Lethiscus* (Pardo et al., 2017), *Oestocephalus* (Carroll, 1998a), apparently *Vieraella* (Estes & Reig, 1973: fig. 1-2; Báez & Basso, 1996: fig. 6) and *Silvanerpeton* (Ruta & Clack, 2006).

Broiliellus (Schoch, 2012: fig. 2F) and *Tseajaia* (Moss, 1972) have state 2.

If *Hyloplesion* was reconstructed correctly by CG78 (fig. 89H), this character is not applicable to it, because the pterygoid does not contribute to the subtemporal fenestra except by most of its quadrate ramus; if the unlikely positioned supposed ectopterygoid is part of the pterygoid instead (see ECT 6 above), *Hyloplesion* has state 2. We have gone with the latter option mainly because, even accepting all of the identifications by CG78, the left pterygoid (though perhaps not the right one) does go just around the corner between the medial and the rostral margin of the subtemporal fossa in one specimen (CG78: fig. 89E). This issue was not addressed or figured by Olori (2015).

State 3 of the present character (= state 2 of PTE 3) is present in *Limnoscelis* (Fracasso, 1983: fig. 2; Reisz, 2007; Berman, Reisz & Scott, 2010), *Paleothyris* (Carroll, 1969b), and *Petrolacosaurus* (Reisz, 1981).

The condition is entirely unknown in *Colosteus* (Hook, 1983: fig. 1B), *Whatcheeria* (the palate of which has not yet been described: Bolt & Lombard, 2000), *Eucritta* (the reconstruction, Clack, 2001: fig. 8, appears overly ambitious judging from the accompanying

text and specimen drawings), *Edops* (D. M., pers. obs. of MCZ 1378; contra Romer & Witter, 1942: fig. 3B), *Batropetes* (Carroll, 1991; Glienke, 2013, 2015), *Asaphestera* (CG78: fig. 6F), and apparently *Pederpes* (Clack & Finney, 2005). The palate of *Keraterpeton* appears to be entirely unknown (Huxley & Wright, 1867; Jaekel, 1903).

Neither state 0 (already scored in RC07) nor state 1 can be excluded for *Isodectes* (Sequeira, 1998). For *Scincosaurus*, figures 2B and 3B of Milner & Ruta (2009) appear to contradict each other, at least on the anatomically left side: fig. 3B shows state 0 as already scored, while fig. 2B shows state 1, unless the bulge in question was dorsal rather than lateral in life; we have scored partial uncertainty here as well.

State 0 can be ruled out for *Bruktererpeton* (Boy & Bandel, 1973: fig. 7); we have accordingly scored state 1, 2 or 3.

Amphibamus (Bolt, 1979: fig. 6B; Milner, 1982: fig. 3b; Schoch & Milner, 2014: fig. 30B) and *Odonterpeton* (CG78: fig. 99A) appear to have states 1 or 2.

**Acanthostomatops* has state 1, though it is close to state 2, at least in two dimensions (Witzmann & Schoch, 2006a).

The reconstruction of **Carrolla* by Maddin, Olori & Anderson (2011: fig. 7B) cannot be reconciled with the specimen (D. M., pers. obs. of TMM 40031-54): the drawing leaves practically no space for the subtemporal fenestra, in particular the coronoid process of the lower jaw which lies in this fenestra in the fossil, because it gives the pterygoid a large lateral extension, implying that the preserved lateral margin must be a break. In the specimen, the margin is finished and rounded, as is the cranial margin of the quadrate ramus; the margin implied by Maddin, Olori & Anderson (2011: fig. 4) should be taken at face value. Furthermore, the quadrate ramus is identified in fig. 4 and described, with its suture to the quadrate, several times in the text of Maddin, Olori & Anderson (2011), yet it is entirely absent from fig. 7B, where the pterygoid does not contact the quadrate at all! – One might wonder whether a transverse flange (state 2) might have merged with the equally transverse quadrate ramus immediately caudal to it; however, the pterygoid ramus of the quadrate articulates with the entire width of the quadrate ramus of the pterygoid, so that the flange would have to be extremely short and limited to the concave transition of the quadrate ramus to the rest of the pterygoid (D. M., pers. obs. of TMM 40031-54). **Carrolla* thus has state 0. It remains to be seen whether this is size-related, however.

"Large teeth" are not defined. Under the assumption that this simply means "not denticles", the row of teeth found in **Archaeovenator* qualifies for state 3, because the largest of those teeth are about the size of the smallest marginal teeth and distinctly larger than all denticles (Reisz & Dilkes, 2003: fig. 2).

120. PTE 7: **Pterygoid quadrate ramus orientated mostly caudally (0) or mostly laterally in ventral aspect (1); ramus absent, quadrate contacts central region of pterygoid (2) (ordered).** State 0 was originally unspecified. State 2 is new and accounts for the condition seen in *Batropetes* (Glienke, 2013), *Brachydectes* (Wellstead, 1991; Pardo & Anderson, 2016) and *Scincosaurus* (Milner & Ruta, 2009); the character is ordered because it is potentially continuous and reflects a widening of the parasphenoid and a rostral repositioning of the quadrates – although the correlation with JAW ART 1/SQU 2/DEN 8 (below) is not perfect.

State 0 is present in *Ventastega* (Ahlberg, Lukševičs & Lebedev, 1994) and *Lethiscus* (Pardo et al., 2017).

Keraterpeton was scored in RC07 as having state 0. The palate appears to be entirely unknown (Huxley & Wright, 1867; Jaekel, 1903), and indeed state 1 would be expected from the proportions of the skull (Jaekel, 1903: fig. 1, 2); we have changed the score to unknown.

State 1 is clearly found in *Diceratosaurus* (Jaekel, 1903: pl. III), *Diplocaulus* (Bossy & Milner, 1998) and *Diploceraspis* (Beerbower, 1963). In *Batrachiderpeton* (Bossy & Milner, 1998), the quadrate ramus is oriented much more laterally than caudally; we count this as state 1 as well.

State 1 is also documented in **Chelotriton* (Roček & Wuttke, 2010; Schoch, Poschmann & Kupfer, 2015); but in MB.Am.45, which is preserved in dorsal view only, state 0 would be expected because the suspensoria extend far caudal to the occiput. We have scored partial uncertainty.

We infer state 0 for the *St. Louis tetrapod because the lower jaw, and indeed its Meckelian fenestra, continues well distal to the basipterygoid process (Clack et al., 2012b).

121. PTE 11: **Pterygoid/maxilla contact: absent (0); present (1).** Because the ectopterygoid, if present, usually lies between the pterygoid and the maxilla, it would have been tempting to merge this character with ECT 1-4 (above), but Clack (1998: fig. 4B) suggests that the maxilla and the pterygoid could meet caudal to the ectopterygoid in *Crassigyrinus* (scored here as unknown), and indeed they meet caudal to the well developed ectopterygoid in *Caerorhachis* (see below) and *Micraroter* (CG78: 88; previously scored as unknown), so we have kept them separate.

Acanthostega (Clack, 1994a), *Acheloma* (Polley & Reisz, 2011), *Bruktererpeton* (Boy & Bandel, 1973: fig. 7), *Batropetes* (Glienke, 2013) and *Lethiscus* (Anderson, Carroll & Rowe, 2003; Pardo et al., 2017) have state 0.

Caerorhachis was scored 0 in RC07. Ruta, Milner & Coates (2002: fig. 4b), however, clearly showed state 1 on the right side of the specimen. On the left side of the same specimen, they reconstructed state 0 (fig. 5c); Ruta, Milner & Coates (2002) did not mention this discrepancy in the text. While it is possible that the left side had state 0, especially if the maxillary process of the pterygoid was genuinely absent and has not broken off, the left side of the palate is much too disarticulated to tell for sure; in particular, the ectopterygoid is missing on that side (fig. 3b, 5a). We have therefore scored state 1 alone.

Unknown in *Whatcheeria* (Lombard & Bolt, 1995), *Doleserpeton* and *Triadobatrachus*, together with but not entirely dependent on the existence and extent of the ectopterygoid (Sigurdsen & Bolt, 2010; Ascarrunz et al., 2016: fig. 4, 12, 3D model 1; see also character ECT 1-4 above), apparently *Keraterpeton* (Huxley & Wright, 1867; Jaekel, 1903) and *Silvanerpeton* (Ruta & Clack, 2006).

The condition in *Edops* is likewise unknown (D. M., pers. obs. of MCZ 1378); RC07 may have misplaced the question mark to the next character, the state of which is known (see below).

For **Erpetosaurus* and the *St. Louis tetrapod, see JUG 3 above.

We follow Pardo et al. (2017: matrix) in scoring state 0 for **Coloraderpeton*.

122. PTE 13: **Pterygoid without (0) or with (1) distinct, me[d]ially directed process for basipterygoid articulation.** This character is only applicable when the interpterygoid vacuities are not too small; we have scored it as inapplicable where that condition is not fulfilled (this concerns a large majority of the OTUs scored 0 by RC07), as well as in a few other OTUs that lack space for such a process like *Greererpeton* or *Limnoscelis* (Reisz, 2007: fig. 6.1; Berman, Reisz & Scott, 2010: fig. 4A).

State 1 is found in *Acanthostega* (weakly expressed: Porro, Rayfield & Clack, 2015), *Edops* (Romer & Witter, 1942: fig. 3B; D. M., pers. obs. of MCZ 1378), *Neldasaurus* (very weakly expressed: Chase, 1965), *Trimerorhachis* (weakly expressed: Milner & Schoch, 2013), *Balanerpeton* (Milner & Sequeira, 1994), *Pelodosotis* (CG78: fig. 48), *Rhynchonkos* (Szostakiwskyj, Pardo & Anderson, 2015), *Hyloplesion* (in the largest of the three specimens

drawn in CG78: fig. 89; photographed in Olori, 2015: fig. 30A), *Diceratosaurus* (Jaekel, 1903: pl. III), *Diplocaulus* (Bossy & Milner, 1998: fig. 57C) where the process is very broad rostrocaudally but no less distinct than elsewhere, *Diploceraspis* (Beerbower, 1963: fig. 4A) where the same situation prevails (partly obscured by the fact that the quadrate ramus is situated so far rostrally), *Ptyonius* (Bossy, 1976: fig. 44, 49), and *Capetus* (Sequeira & Milner, 1993). The process is apparently very short but nonetheless distinct in *Batropetes* (Glienke, 2013), so we have assigned state 1 to it as well.

The palate of *Keraterpeton* appears to be entirely unknown (Huxley & Wright, 1867; Jaekel, 1903).

State 0 makes a surprise appearance in *Lethiscus* (Pardo et al., 2017).

**Micropholis* is polymorphic (Schoch & Rubidge, 2005). So is **Sclerocephalus* (Schoch & Witzmann, 2009a: fig. 6).

Although likely shorter than implied by Maddin, Olori & Anderson (2011: fig. 7B), the process is clearly present (state 1) in **Carrolla*.

We have also assigned state 1 to the special case of **Quasicaecilia*, where the process is very long and clearly distinct, but points straight caudally because the jaw articulation is so far rostral (Pardo, Szostakiwskyj & Anderson, 2015).

Although very wide rostrocaudally, the process is present (state 1) in **Australerpeton* (Eltink & Langer, 2016).

Even though this character is inapplicable to *Hapsidopareion*, **Llistrofus* has state 1 (Bolt & Rieppel, 2009).

123. PTE 14: **Quadrate ramus of pterygoid more than (0) or at most twice as long as maximally broad (1).** The original wording was more impressionistic: "Pterygoid quadrate ramus a robust structure, indistinctly merging into basal and palatal processes: absent (0); present (1)", explained in the next sentence as the quadrate ramus being "a stout structure, slightly longer than wide and without a neat separation from the rest of the bone". We have reduced this to the length/width ratio, which we have changed because the quadrate ramus is considerably longer than broad in most OTUs that were scored 1 in RC07 (all lissamphibians, *Micromelerpeton*, and all branchiosaurids were scored 1, everything else was given state 0).

State 0 as defined by us occurs in *Micromelerpeton* (Boy, 1995), all branchiosaurids (Boy, 1986, 1987), and the lissamphibians *Eocaecilia* (Jenkins, Walsh & Carroll, 2007), *Triadobatrachus* (Roček & Rage, 2000; Ascarrunz et al., 2016; D. M. and M. L., pers. obs. of MNHN F.MAE.126), and *Valdotriton* (Evans & Milner, 1996) as well as **Liaobatrachus* (Dong et al., 2013). Less surprisingly, it is known from *Ventastega* (Ahlberg, Lukševičs & Lebedev, 1994) and *Lethiscus* (Pardo et al., 2017).

We find state 1 in *Baphetes* (Beaumont, 1977), *Eucritta* (Clack, 2001), *Isodectes* (Sequeira, 1998), *Platyrhinops* (borderline; Clack & Milner, 2010), *Caerorhachis* (Ruta, Milner & Coates, 2002), *Eoherpeton* (Smithson, 1985), *Pholiderpeton attheyi* (Panchen, 1972), *Ph. scutigerum* (Clack, 1987b), *Asaphestera*, *Micraroter* (marginally) and *Cardiocephalus* (CG78), *Scincosaurus* (Milner & Ruta, 2009), *Batrachiderpeton* (Bossy & Milner, 1998), *Diceratosaurus* (Jaekel, 1903), *Diplocaulus* (the pterygoids of which which look like those of the salamander *Karaurus*: Bossy & Milner, 1998), *Diploceraspis* (Beerbower, 1963), *Capetus* (Sequeira & Milner, 1993) and *Silvanerpeton* (Ruta & Clack, 2006) as well as, unexpectedly, **Palaeoherpeton* (Panchen, 1964: fig. 13).

Euryodus is polymorphic (CG78).

The condition is unknown in *Anthracosaurus* (Panchen, 1977), *Odonterpeton* (CG78: fig. 99A), *Adelospondylus* (Andrews & Carroll, 1991; it is too difficult to decide how to measure the reconstruction drawing and how to interpret it in relation to the specimen drawings), *Keraterpeton* (Huxley & Wright, 1867; Jaekel, 1903), and inapplicable in *Batro-*

petes, which likely lacks a quadrate ramus (Glienke, 2013), and *Brachydectes*, which clearly lacks a quadrate ramus (Wellstead, 1991; Pardo & Anderson, 2016), as well as in *Oestocephalus* (Carroll, 1998a), which lacks clear sutures between the pterygoid and other bones such as the epipterygoid and the quadrate.

Apparently borderline in the incompletely preserved **Neopteroplax* (Romer, 1963: fig. 2); we have scored it as unknown.

deleted PTE 15: **Pterygoid quadrate ramus straight, rod-like and gently tapering distally in ventral aspect: absent (0); present (1).** The ventral surface of the quadrate ramus further "is parallel-sided for most of its length and narrows smoothly in its rearmost part" in state 1, which is supposed to be present in "some dissorophoids and *Eocaecilia*". This unquantified description, which is strongly reminiscent of PTE 14, turns out to be difficult to apply to many (if not most) OTUs, or at least to the line drawings that fail to show that the quadrate ramus is a more or less vertical lamina rather than a rod. The visible tapering of the process further depends strongly on diagenetic compression. We fail to see a difference between the conditions of *Broiliellus* (scored 0 by RC07), *Platyrhinops* (0), *Eoscopus* (1), *Doleserpeton* (1), or probably even *Eocaecilia* (1). For the time being, we have therefore deleted this character.

124. PTE 16: **Pterygoid palatal ramus without (0) or with (1) distinct, anterior and unornamented digitiform process.** State 1 requires that the pterygoids meet rostral to the parasphenoid; where this is not the case (VOM 5-10/PTE 10-12-18/INT VAC 1 having a state other than 0 or 1), this character is inapplicable.

State 0 is found in *Ventastega* (Ahlberg, Lukševičs & Lebedev, 1994), *Microbrachis* (Vallin & Laurin, 2004; Olori, 2015) and *Ossinodus* (Warren 2007).

The condition of *Westlothiana* is unknown (Smithson et al., 1994) in addition to being inapplicable. The palate of *Keraterpeton* appears to be entirely unknown (Huxley & Wright, 1867; Jaekel, 1903).

We have counted the condition of **Chroniosaurus* (Klembara, Clack & Čerňanský, 2010) as state 1, although it may not be unornamented enough.

125. PTE 17: **Basal region of pterygoid immediately anterior to quadrate ramus without (0) or with (1) sharply defined, elongate longitudinal groove.**

State 0 is present in *Ventastega* (Ahlberg, Lukševičs & Lebedev, 1994) and *Lethiscus* (Pardo et al., 2017).

State 1 is known in *Euryodus* (CG78) and makes a surprise appearance in **Liaobatrachus* (Dong et al., 2013).

Edops is probably best scored as unknown (D. M., pers. obs. of MCZ 1378).

The palate of *Keraterpeton* appears to be entirely unknown (Huxley & Wright, 1867; Jaekel, 1903).

126. PTE 19: **Robust, strut-like pterygoid–squamosal process providing support for quadrate: absent (0); present (1).** This refers to the condition seen today in salamanders, where the quadrate process of the pterygoid is largely parallel to the ventrolateral process of the squamosal (= its main body), and the two together almost completely encase the (often partly or wholly unossified) quadrate for its entire length or nearly so.

State 0 is present in *Ventastega* (Ahlberg, Lukševičs & Lebedev, 1994), *Lethiscus* (Pardo et al., 2017) and, surprisingly, **Chelotriton* (Schoch, Poschmann & Kupfer, 2015).

As preserved, *Triadobatrachus* comes close to state 1; this is almost certainly due to crushing, however (Ascarrunz et al., 2016: 3D model 1), so we have kept state 0.

The palate of *Keraterpeton* appears to be entirely unknown (Huxley & Wright, 1867; Jaekel, 1903).

127. INT VAC 2: **Interpterygoid vacuities and cultriform process together occupy at least half of palatal width: absent (0); present (1).** We have added the cultriform process of the parasphenoid to make clear that we measured the distance between the lateral extremities of the vacuities; as a side-effect, this increases the applicability of this character – specifically, *Brachydectes* has state 1 despite lacking interpterygoid vacuities (Wellstead, 1991; Pardo & Anderson, 2016). On the other hand, while a width of zero could be measured and scored, it follows automatically from state VOM 5-10/PTE 10-12-18/INT VAC 1(0); we have scored these OTUs as inapplicable, as RC07 did in a few cases.

State 1 is also found in *Cochleosaurus* (Sequeira, 2004), Albanerpetidae (McGowan, 2002; Maddin et al., 2013a: fig. 5H, I), *Eocaecilia* (Jenkins, Walsh & Carroll, 2007) and *Batropetes*, the only OTU in this matrix that is known to combine state 1 with convex pterygoid margins (INT VAC 3(0)) (Glienke, 2013). *Lethiscus* appears to reach state 1 as well (Pardo et al., 2017; J. Pardo, pers. comm.).

Whatcheeria has state 0 (Lombard & Bolt 1995). So do *Bruktererpeton* (Boy & Bandel, 1973), *Diadectes* (Berman, Sumida & Martens, 1998) and *Diceratosaurus* (Jaekel, 1903; D. M., pers. obs. of MB.Am.778).

**Carrolla* shares state 0: the vacuities are probably considerably less wide than half of the palate (D. M., pers. obs. of TMM 40031-54; contra Maddin, Olori & Anderson, 2011: fig. 7B).

The palate of *Keraterpeton* appears to be entirely unknown (Huxley & Wright, 1867; Jaekel, 1903).

128. INT VAC 3: **Interpterygoid vacuities concave along their whole margins: absent (0); present (1).** We count missing parts of margins as concave (for instance in salamanders where the palatine is absent and the pterygoid does not reach the maxilla). In the absence of interpterygoid vacuities, however (VOM 5-10/PTE 10-12-18/INT VAC 1(0)), this character is inapplicable.

Batropetes is the only OTU in this matrix, with the possible exception of *Eocaecilia*, that combines convex pterygoid margins (state 0 of the present character) with vacuities that are together half as wide as the palate (INT VAC 2(1)) (Glienke, 2013).

Albanerpetidae has state 0: the only known margins are very slightly convex in their caudal half (McGowan, 2002; Maddin et al., 2013a: PDF version of fig. 5H, I at 500%). So do *Bruktererpeton* (Boy & Bandel, 1973), *Diadectes* (Berman, Sumida & Martens, 1998), *Diceratosaurus* (Jaekel, 1903; D. M., pers. obs. of MB.Am.778) and *Lethiscus* (Pardo et al., 2017).

Eocaecilia is borderline (Jenkins, Walsh & Carroll, 2007: fig. 3); we have scored it as unknown. *Phlegethontia*, too, is borderline (Anderson, 2002: fig. 8.2), so we have kept the score as unknown.

State 1 is arguably found in *Caerorhachis* (Ruta, Milner & Coates, 2002: fig. 5c), apparently in *Stegotretus* (Berman, Eberth & Brinkman, 1988), almost certainly in *Asaphestera* (CG78: fig. 6F, 7), borderline but certainly in *Ptyonius* (Bossy & Milner, 1998: fig. 75B), perhaps less certainly but not borderline in *Urocordylus* (Bossy & Milner, 1998: fig. 55A), and definitely in *Capetus* (Sequeira & Milner, 1993: fig. 7, pl. 3). It further occurs in *Tuditanus* (Carroll & Baird, 1968: fig. 5); the reconstruction (fig. 9) shows state 1 on the anatomical right and 0 on the left side, but the left pterygoid is entirely unknown (fig. 5).

**Sclerocephalus* is polymorphic (Schoch & Witzmann, 2009a: fig. 4, 6). So is apparently *Pantylus* (left and right side of the specimen reconstructed by Romer, 1969: fig. 5; copied by CG78: fig. 25).

Carrolla has state 0: the vacuity margins are partly straight (D. M., pers. obs. of TMM 40031-54; only stippled by Maddin, Olori & Anderson, 2011: fig. 7B).

Unknown in *Eoherpeton* (Smithson, 1985). The palate of *Keraterpeton* appears to be entirely unknown (Huxley & Wright, 1867; Jaekel, 1903).

129. INT VAC 4: **Interpterygoid vacuities and cultriform process together broader than long: absent (0); present (1).** We have added the cultriform process of the parasphenoid to make clear that we measured the distance between the lateral extremities of the vacuities; as a side-effect, it increases the applicability of this character – *Brachydectes* in particular has state 0 (Wellstead, 1991; Pardo & Anderson, 2016). On the other hand, while a width of zero could be measured and scored, it is already coded as state VOM 5-10/PTE 10-12-18/INT VAC 1(0); we have scored these OTUs as inapplicable for the present character, as RC07 did in some cases.

The combination of state 1 with INT VAC 3(0) does not occur in this matrix, but is known in larvae and some adults of **extant caecilians (Reiss, 1996); we have therefore not merged these characters.

Cochleosaurus has state 0 (Sequeira, 2004), as do *Bruktererpeton* (Boy & Bandel, 1973), *Diadectes* (Berman, Sumida & Martens, 1998), *Batropetes* (Glienke, 2013), *Diceratosaurus* (Jaekel, 1903; D. M., pers. obs. of MB.Am.778) and *Lethiscus* (Pardo et al., 2017).

State 1 is found in Albanerpetidae (McGowan, 2002; Maddin et al., 2013a: PDF version of fig. 5I at 500%), almost certainly in *Asaphestera* (CG78: fig. 6F, 7), and most likely in *Hyloplesion* (Olori, 2015: fig. 30C, ?A).

The palate of *Keraterpeton* appears to be entirely unknown (Huxley & Wright, 1867; Jaekel, 1903).

We have scored state 0 for **Coloraderpeton* following the matrix by Pardo et al. (2017).

130. CHO 1: **Choana wider in its anterior half than in its posterior half: no (0); yes (1).**

State 0 is found in *Crassigyrinus* (Clack, 1998), *Platyrhinops* (Clack & Milner, 2010), Albanerpetidae (Maddin et al., 2013a: PDF version of fig. 5I at 500%), *Bruktererpeton* (Boy & Bandel, 1973: 51), *Kotlassia* (Bulanov, 2003: fig. 30), *Seymouria* (Laurin, 2000; Klembara et al., 2007), *Batropetes* (most likely; Glienke, 2013, 2015), *Lethiscus* (Pardo et al., 2017), *Orobates* (Nyakatura et al., 2015: digital reconstruction) and *Silvanerpeton* (Ruta & Clack, 2006).

State 1 occurs throughout Dendrerpetidae (Godfrey, Fiorillo & Carroll, 1987; Milner, 1996; Holmes, Carroll & Reisz, 1998) and is shared by *Acheloma* (Polley & Reisz, 2011), *Ecolsonia* (Berman, Reisz & Eberth, 1985), *Amphibamus* (Schoch & Milner, 2014: fig. 30B), *Doleserpeton* (Sigurdsen & Bolt, 2010), *Limnoscelis* (Berman, Reisz & Scott, 2010), *Pantylus* (Romer, 1969; CG78) and *Stegotretus* (Berman, Eberth & Brinkman, 1988). Judging from the shape of the vomer, it is also a safe bet in *Vieraella* (Báez & Basso, 1996).

Unknown in *Saxonerpeton* (CG78: fig. 21), *Micraroter* (CG78) and *Scincosaurus* (Milner & Ruta, 2009).

**Sclerocephalus* is polymorphic (Schoch & Witzmann, 2009a: fig. 4).

131. CHO 2: **Choana expanded transversely along its medial margin: absent (0); present (1).** We cannot see a difference between the taxa that were scored 1 and most of the ones that were scored 0. We have therefore tried to define state 1 as a choana that is wide linguolabially compared to the palatine and any teeth it may bear; there do not seem to be many borderline cases.

We count the absence of a medial wall (as in *Valdotriton*) as state 1.

Platyrhinops (Clack & Milner, 2010) and *Bruktererpeton* (Boy & Bandel, 1973: 51) have state 0.

State 1 is found in *Colosteus* (Hook, 1983), *Crassigyrinus* (Clack, 1998), *Edops* (Romer & Witter, 1942; D. M., pers. obs. of MCZ 1378), *Chenoprosopus* (Langston, 1953), *Cochleosaurus* (Sequeira, 2004), *Trimerorhachis* (Milner & Schoch, 2013), *Balanerpeton* (Milner & Sequeira, 1994), Dendrerpetidae (Godfrey, Fiorillo & Carroll, 1987; Milner, 1996; Holmes, Carroll & Reisz, 1998), *Eryops* (Sawin 1941), *Acheloma* (Polley & Reisz, 2011), *Phonerpeton* (Dilkes, 1990), *Ecolsonia* (Berman, Reisz & Eberth, 1985), *Broiliellus* (even though the choana is extremely long and therefore looks narrow: Carroll, 1964: fig. 10; Schoch, 2012: fig. 2F), *Micromelerpeton* (Boy, 1995; Schoch, 2009b), Albanerpetidae (Maddin et al., 2013a: PDF version of fig. 5I at 500%), *Caerorhachis* (Ruta, Milner & Coates, 2002), *Eoherpeton* (Panchen, 1975; Smithson, 1985), *Proterogyrinus* (Holmes, 1984), *Pholiderpeton scutigerum* (Clack, 1987b), *Gephyrostegus* (Klembara et al., 2014), *Kotlassia* (Bulanov, 2003: fig. 30), *Discosauriscus* and *Seymouria* (Klembara et al., 2007; though borderline in *S. baylorensis*: Laurin, 2000), *Limnoscelis* (Berman, Reisz & Scott, 2010), *Batropetes* (Glienke, 2013, 2015), *Pantylus* (CG78), *Stegotretus* (Berman, Eberth & Brinkman, 1988), apparently *Rhynchonkos* (Szostakiwskyj, Pardo & Anderson, 2015), *Brachydectes* (Wellstead, 1991; Pardo & Anderson, 2016), *Diceratosaurus* (D. M., pers. obs. of MB.Am.778), *Diplocaulus* (Bossy & Milner, 1998), *Diploceraspis* (Beerbower, 1963), *Lethiscus* (Pardo et al., 2017), *Orobates* (Nyakatura et al., 2015: digital reconstruction) and *Silvanerpeton* (Ruta & Clack, 2006). Judging from the shape of the vomer, it is also a safe bet in *Vieraella* (Báez & Basso, 1996). **Saharastega* most likely shared it (Damiani et al., 2006; D. M., pers. obs. of MNN MOR 73).

Unknown in *Pholiderpeton attheyi*, where it may have been borderline (Panchen, 1972); further unknown in *Saxonerpeton* (CG78: fig. 21), *Micraroter* (CG78) and *Scincosaurus* (Milner & Ruta, 2009).

**Sclerocephalus* is polymorphic (Schoch & Witzmann, 2009a: fig. 4).

132. ANT VAC 2: **Anterior palatal vacuity present and single (0), present and double (1), or absent (2) (unordered).** RC07 created this character by merging ANT VAC 1 and ANT VAC 2 of Ruta, Coates & Quicke (2003). We have not ordered this character because no sequence is obvious. We interpret "vacuity" to mean "fenestra" exclusively; fossae not pierced by fenestrae are scored as state 2.

State 0 or 1 is present in *Ventastega* (Ahlberg, Lukševičs & Lebedev, 1994).

Colosteus (Hook, 1983) and *Crassigyrinus* (Clack, 1998) have state 1.

Not described or illustrated in *Whatcheeria* (Lombard & Bolt, 1995; Bolt & Lombard, 2000).

One specimen of *Megalocephalus* has state 1, unlike the others that have state 0 (Beaumont, 1977) as previously scored; we have changed the score to polymorphism.

Karaurus was scored as possessing state 2 in RC07, but has only been reconstructed (Ivachnenko, 1978) with a very large, single vacuity, thus state 0; *Bruktererpeton* shares state 0 (Boy & Bandel, 1973: 51), and apparently so does *Vieraella* (Báez & Basso, 1996: fig. 5, 7).

State 2 is known in *Batropetes* (Glienke, 2013), *Diceratosaurus* (D. M., pers. obs. of MB.Am.778) and *Orobates* (Nyakatura et al., 2015: digital reconstruction).

Lethiscus appears to have state 0 (Pardo et al., 2017; also scored in their matrix).

The condition is unknown in *Edops* (Romer & Witter, 1942; D. M., pers. obs. of MCZ 1378 – the tip of the snout consists only of plaster), *Gephyrostegus* (Klembara et al., 2014:

fig. 2), *Scincosaurus* (Milner & Ruta, 2009), *Silvanerpeton* (Ruta & Clack, 2006) and *Tseajaia* (Moss, 1972: 12).

States 0 and 2 are both known to occur in **Micropholis* (Schoch & Rubidge, 2005).

State 0 was reconstructed for **Liaobatrachus* (*L. zhaoi*: Dong et al., 2013: fig. 7B), but the photos show that the vacuity, if present, must have been smaller than reconstructed; because the text does not mention the vacuity at all, we have scored it as unknown.

For **Pholidogaster* we have followed Panchen (1975: 625), who considered state 1 "the most probable" condition. For **Coloraderpeton* we have followed the matrix of Pardo et al. (2017) in scoring state 0.

133. SUPOCC 1: **Caudal exposure of separately ossified supr[…]occipital: absent (0); present (1).** The caudal exposure has to be specified because a suproccipital – or in any case an ossification of the synotic tectum – can be present and form the roof of the braincase without being visible from the outside at all (Olson, 1941: 162, fig. 8; Bystrow, 1944; Berman, Eberth & Brinkman, 1988; Maddin, Reisz & Anderson, 2010; Polley & Reisz, 2011, and references therein – though some of these cases may represent the suprotic rather than the suproccipital: compare Grande & Bemis, 1998, and Cubbage & Mabee, 1996).

Damiani et al. (2006) cited Sequeira (1998) for the claim that *Isodectes* has a caudally exposed suproccipital (though not necessarily as a separate bone, and thus not necessarily state 1 of the present character). Not only did Sequeira (1998) not mention or illustrate such an ossification, but her fig. 4B shows a median suture between the exoccipitals dorsal to the foramen magnum, continuous with the median suture between the postparietals, leaving no space for a suproccipital. We have therefore kept the score of 0 for *Isodectes*.

State 0 is apparently present in Dendrerpetidae (Robinson, Ahlberg & Koentges, 2005) and *Diplocaulus* (Douthitt, 1917).

Bolt (1969: 889) stated explicitly that *Doleserpeton* clearly shows state 0: "There is no supraoccipital bone, and indeed no room for one, as the opisthotics cover the tops of the exoccipitals and, in maturer specimens, fuse above the foramen magnum." This is confirmed by Sigurdsen (2008) and Sigurdsen & Bolt (2010).

Eocaecilia shares state 0, as demonstrated by the median dorsal suture in the braincase (Jenkins, Walsh & Carroll, 2007) – the suproccipital is a single median bone and would make such a suture impossible. The same holds for *Notobatrachus*, at least in the reconstructions by Estes & Reig (1973) and Báez & Nicoli (2004), but apparently also in fig. 4 of the latter (a photograph of a cast of the holotype).

Although *Stegotretus* has a suproccipital (or some other ossification in that area), it appears not to have been exposed, but completely covered by the postparietals in caudal view (Berman, Eberth & Brinkman, 1988: 310). This constitutes state 0.

Euryodus was scored as unknown in RC07, but we cannot distinguish its condition from that of *Cardiocephalus* which was scored 0 (both according to CG78); we have assigned state 0 to both.

Unknown in *Eusthenopteron*, where the entire braincase is fused (Carroll & Chorn, 1995), *Megalocephalus* (similarly due to fusion: Beaumont, 1977: 65f.), *Phonerpeton* (Dilkes, 1990), *Eoscopus*, where "[b]raincase bones […] were unossified in all specimens" (Daly, 1994: 8), *Platyrhinops* (Clack & Milner, 2010), *Eoherpeton* (Holmes, 1984: fig. 4; Smithson, 1985), *Westlothiana*, where the bone interpreted as such by Smithson et al. (1994) could just as well be e.g. a part of the otic capsules (as previously noted by Laurin & Reisz, 1999), and *Lethiscus* (Pardo et al., 2017; as already scored) and *Phlegethontia* (Anderson, 2002), where the braincase roof is thin but fused.

Panchen (1964) first described state 1 in **Palaeoherpeton*, but later regarded this as "an artefact of preservation" (Clack & Holmes, 1988: 91). We have scored state 0.

134. EXOCC 2-3-4-5/BASOCC 1-5: **Basioccipital not restricting notochord (0); single occipital cotyle (with or without notochordal funnel) (1); single occipital condyle (with or without notochordal pit) (2); exoccipital articulating surfaces dorsolateral or lateral to basioccipital, basioccipital cotyle articulating with interglenoid tubercle (3); basioccipital not participating in articulation or absent, two exoccipital condyles or flat surfaces not touching each other (4); two condyles or flat surfaces touching each other, "exoccipital-basioccipital complex" without sutures (5) (stepmatrix).** We have merged six correlated characters (see below for the seventh), each of which had only one of its two states described; state 1 of each of these characters made state 1 of all five (indeed six) others impossible. State 0 of the present character is BASOCC 1(0), which was originally called "basioccipital notochordal", an imprecise term; state 5 is a modification of EXOCC 2(1) and EXOCC 5(1) and occurs in *Acheloma* (Olson, 1941: fig. 8; Polley & Reisz, 2011), *Phonerpeton* (Dilkes, 1990; D. M., pers. obs. of MCZ 2313), *Ecolsonia* (Berman, Reisz & Eberth, 1985; D. M., pers. obs. of CM 38017) and *Doleserpeton* (Sigurdsen, 2008) as well as **Konzhukovia* (with a dorsal incisure that connects to the notochordal pit) and **Platyoposaurus* (Gubin, 1991). See below for *Eryops*. The stepmatrix for this character is Appendix-Table 4.

Within state 1, it might be possible to distinguish a closed cotyle from a deep funnel as found in the aïstopods (*Lethiscus*: Pardo et al., 2017; *Oestocephalus*: Carroll, 1998a; *Phlegethontia*: Anderson, 2002, and references therein). We speculate that this distinction may have been the original point of BASOCC 6 (see below). However, incomplete ossification, bad preservation and inadequate illustration would prevent us from scoring almost any other of the OTUs currently scored 1 as having a cotyle or a funnel.

OTUs known to possess an intercotylar tubercle on the atlas (CER VER 4(1), see ch. 253) are scored as having state 1, 3, 4 or 5 of the present character if it is in fact unknown, because only these four states can occur in that case. The only occurrences of this situation are *Saxonerpeton* (made explicit by CG78: 34), **Gerobatrachus* (Anderson et al., 2008a) and **Beiyanerpeton* (Gao & Shubin, 2012: fig. 3).

Because four (and not just one) states can occur together with it, we have not merged CER VER 4 with the present character. For convenience, however, we have ignored the fact that CER VER 4(0) makes state 3 of the present character impossible (a partial uncertainty of five states is probably more trouble than it is worth, and the two states in question occur in separate areas of the tree).

State 1 is present in Dendrerpetidae (Robinson, Ahlberg & Koentges, 2005). It also appears to occur in *Ariekanerpeton* (Klembara & Ruta, 2005a) and probably *Utegenia* (Klembara & Ruta, 2004a); the same seems to hold for *Discosauriscus*, where the poorly ossified basioccipital is a caudally flat plate, but the exoccipitals appear to participate in the cotyle (Klembara, 2007).

The taphonomically crushed *Tseajaia* appears to possess state 2, as far as we can tell from Moss (1972).

We have also assigned state 2 to *Captorhinus* because it has a condyle, even though no sutures can be traced in its "exoccipital-basioccipital complex", and to *Orobates*, even though the notochordal pit, though shallow, is large and even though the digital reconstruction by Nyakatura et al. (2015) does not show sutures (generally) and does not compensate for all deformation and disarticulation.

Hapsidopareion and **Llistrofus* (CG78: 27, 28; Bolt & Rieppel, 2009) are borderline between states 3 and 4, but we have stayed conservative and assigned state 3 to both because the basioccipital does seem to have participated in the articulation.

Szostakiwskyj, Pardo & Anderson (2015) contradicted themselves: in their description of *Rhynchonkos*, they stated that only a flat eroded surface is preserved, which would mean that the range of possibilities for this character could only be narrowed down to state 1, 2, 3 or 4, while in their discussion, they stated that state 3 is present. J. Pardo (pers. comm. 2015) confirms the latter: although the area is eroded, state 3 is still visible.

State 4 is found in *Batropetes* (Carroll, 1991: fig. 5; Glienke, 2013: fig. 4, showing a ventral suture between the exoccipitals) and *Diceratosaurus* (D. M., pers. obs. of CM 72608). Outside the original taxon sample, it is standard in lissamphibians and also occurs in most *stereospondylomorph temnospondyls.

Asaphestera has a unique state (D. M., pers. obs. of NMC 10041 with J. Anderson). The drawing of the same specimen in CG78 (fig. 6E) is accurate as a two-dimensional projection in strict caudal view. In other words, state 3 (which RC07 had scored) is not reached: on its ventral side, the basioccipital sends a prong far caudal, while the dorsal side is flat until very far rostral, where it curves dorsally. To articulate with this latter surface, an interglenoid tubercle would have had to be much longer than known in any other vertebrates; realistically, only the dorsal surface of the prong could have articulated with the ventral side – not the rostral end – of such a tubercle on the atlas. We have scored state 4: although the basioccipital is present and bears the mentioned large caudal process, it does not participate in an articular surface together with the widely spaced exoccipitals.

Eryops appears to be quite variable and generally transitional. A dorsoventrally compressed, bilobed version of state 1, with a median constriction, is known to occur (Sawin, 1941; D. M., pers. obs. of the large skull TMM 40349-20 and the smaller skull MCZ 2766); however, TMM 31226-12 and MCZ 1129 have state 4, AMNH 23529 has state 5 (unless it can be interpreted as having state 2 with a very large notochordal pit), AMNH 4673 has state 5 bordering on 4, the partially encrusted AMNH 4180 most likely has state 4 or 5, as does AMNH 4186, AMNH 4183 has 5 or possibly 1, and the uncatalogued USNM specimens "Texas '84 #40" and "Texas '86 #77" have or come close to states 4 and/or 5 (all pers. obs. by D. M.). It should be investigated whether all these skulls should continue to be referred to the same species. Unfortunately we have not been able to rigorously examine whether this variation correlates with the neat division into a narrow-headed and a broad-headed morph (Werneburg, 2007b; Schoch & Milner, 2014; D. M., pers. obs. of USNM, TMM, AMNH and MCZ specimens), although there is currently no reason to think it does: for example, AMNH 4673, AMNH 4180 and AMNH 4183 are narrow-headed, while the enormous AMNH 4186 is broad-headed. Incomplete ossification and difficult preservation (such as the common presence of an ironstone crust) contribute to the confusion; unfortunately, this is the case for the type specimen of the type and possibly only species *E. megacephalus*, the narrow-headed AMNH 4189, which could have any of the three states in question (D. M., pers. obs.). For the time being, we have scored *Eryops* as possessing all three states: 1, 4 and 5.

Entirely unknown in *Baphetes* (Beaumont, 1977), apparently *Amphibamus* (Watson, 1940; Carroll, 1964; Daly, 1994), *Caerorhachis* (Ruta, Milner & Coates, 2002) and *Hyloplesion* (CG78). Contrary to Carroll (1970: 274, fig. 5), Klembara et al. (2014: 787–788) have further argued that the entire braincase is unknown in *Gephyrostegus*.

Cochleosaurus has state 1 or 3 (Sequeira, 2004).

Platyrhinops appears to have state 4 or 5 (Clack & Milner, 2010).

Diadectes may be said to change from state 1 to state 2 in ontogeny. In AMNH 4839 (D. M., pers. obs.), the convexity that defines state 2 is hard to find, only what must be the incompletely ossified exoccipitals are slightly convex in lateral view; the articulation as a whole may be interpreted as state 2 with a giant conical notochordal pit that takes almost all the convexity away. This interpretation is confirmed by the considerably larger specimen AMNH

4352 (D. M., pers. obs.), in which the notochordal pit is considerably smaller and surrounded by a thick convex rim. We have thus kept state 2 for *Diadectes*.

The preservation of *Limnoscelis* (Berman, Reisz & Scott, 2010: fig. 10, 12) is insufficient to distinguish states 1, 2, 3 and 5. We have scored partial uncertainty. Interestingly, Berman, Reisz & Scott (2010: fig. 4) reconstructed a condition intermediate between all four of those states – a flat plate with the notochordal pit that is visible in the specimen (fig. 10, 12).

Odonterpeton has state 1 or 3; CG78 (145) suggested that it has 2, but that is almost certainly incorrect – the most evident candidate for a median condyle is simply the anatomically left rim of the cotyle, against which the vertebral column has slipped to the right (D. M., pers. obs. of USNM 4465+4467). The other candidate consists of two tiny grains that are probably not part of a condyle. – As mentioned above, fig. 99A of CG78 is idealized and simplified to the extent of being unreliable.

We have scored *Ptyonius*, *Sauropleura* and *Urocordylus* as possessing state 1 or 3 based on Bossy & Milner (1998: 86, 91). Bossy & Milner (1998: 91) appear to rule out state 4 shortly after mentioning that the median part of the cranial face of the atlas centrum can be "somewhat protruding" in some or all of these taxa; probably this only means that the interglenoid tubercle (see CER VER 4) cannot be as large as it often is in "microsaurs" – it is quite small in many lissamphibians and in the "microsaur" *Odonterpeton* (see CER VER 4 below).

Silvanerpeton has state 0 or 1 (Ruta & Clack, 2006).

State 1 is almost certain in **Nigerpeton* (D. M., pers. obs. of MNN MOR 69 and MNN MOR 70).

**Iberospondylus* appears to have the version of state 1 sometimes seen in *Eryops* (D. M., pers. obs. of PU-ANF 15 with Rodrigo Soler-Gijón).

**Utaherpeton*, which has lateral facets for the exoccipitals on the basioccipital, is scored 1 or 3 because Carroll, Bybee & Tidwell (1991) did not explicitly argue against the version of state 2 sometimes seen in *Eryops*.

**Sparodus* appears to have states 3 or 4 (Carroll, 1988: fig. 1A), although a suture between the possible occipital condyles and the postparietals cannot be determined (D. M., pers. obs. of NHMW 1899/0003/0006).

We have assigned states 3, 4 or 5 to **Acanthostomatops* because its basioccipital was probably small, judging from the size of the facet for it on the parasphenoid (Witzmann & Schoch, 2006a).

The CT scans of **Carrolla* (only known specimen: TMM 40031-54) by Maddin, Olori & Anderson (2011) seem to only distinguish finished bone surfaces from everything else, and not to distinguish spongy bone from the matrix. Thus, the figures of Maddin, Olori & Anderson (2011), including the reconstruction (fig. 7B), fail to show some bone that is clearly present. This includes unfinished bone between the finished lateral extremities of the occipital articulation (D. M., pers. obs. of TMM 40031-54). As there is no interruption in the almost flat unfinished surface of this articulation, while there is a strong median constriction, the state shown by the only known specimen of **Carrolla* is best called 5, making **Carrolla* the only non-temnospondyl in this matrix to possess this state. However, it is entirely possible that further ossification would have transformed this into state 4. We have scored this as partial uncertainty. The strong constriction makes it unlikely that state 3 (expected for a "microsaur") would have developed.

For Analysis EB, this character was split as follows:
BASOCC 1-5: **Occipital articulation absent (unrestricted notochord) (0); concave (occipital cotyle) (1); convex (occipital condyle) (2) (ordered).** These states correspond to

states 0, 1+3 and 2+3+4+5 of the merged character, respectively; state 3 of the merged character is scored as partial uncertainty of the present one.

EXOCC 2-3-5: **Occipital articulation single (0); bilobed (1); double (2) (ordered).** These states correspond to states 1+2, 3+5 and 3+4 of the merged character, respectively. The present character is inapplicable to OTUs with state 0 of the preceding and thus the merged character; state 3 of the merged character is scored as partial uncertainty of the present one.

EXOCC 4: **Basioccipital participates in articulation: yes (0); no (1).** Only applicable to double occipital articulations, so that the states correspond to states 3 and 4 of the merged character.

deleted BASOCC 6: **Articulation surface of the basioccipital circular and recessed: absent (0); present (1).** This character was originally explained as follows: "In aïstopods and adelospondyls, the basioccipital has a circular outline and carries a funnel-like excavation." Specifically, state 1 was assigned to *Oestocephalus*, *Phlegethontia* and *Adelogyrinus*; the other aïstopods and adelospondyls were (correctly) scored as unknown. However, *Oestocephalus* and *Phlegethontia* (with the possible exception of one specimen: Anderson, 2002) lack sutures in the braincase, making it impossible to determine which bones make up their circular occipital cotyle; they have to be scored as unknown as well. (The same holds for *Lethiscus*, which was already scored as unknown: Pardo et al., 2017.) This leaves state 1 to *Adelogyrinus* alone (if that, given the damage mentioned by Andrews & Carroll, 1991: 250), rendering the character parsimony-uninformative. We have accordingly deleted it.

Appendix-Table 4: Stepmatrix for character 134 (EXOCC 2-3-4-5/BASOCC 1-5).

**from ↓ to →**	**0**	**1**	**2**	**3**	**4**	**5**
**0**	0	1	2	2	3	3
**1**	1	0	1	1	2	2
**2**	2	1	0	2	2	1
**3**	2	1	2	0	1	2
**4**	3	2	2	1	0	1
**5**	3	2	1	2	1	0

135. OPI 2, POSPAR 5: **Exoccipitals contact postparietals or parietals (0); opisthotics and/or suproccipital separating exoccipitals from postparietals or parietals (1); separate opisthotics absent (2) (unordered).** No sequence for ordering suggests itself.

OPI 2 was originally worded "Opisthotic[s] forming a thickened plate together with the supraoccipital, preventing the exoccipitals from contacting the skull table: absent (0); present (1)" in RC07: 102, but whether a "plate" is present depends on PTF 1, and the presence of the suproccipital is already another character (SUPOCC 1), so, in order to avoid redundancy, it should not be mentioned here.

POSPAR 5 was worded "Postparietal/exoccipital suture: absent (0); present (1)" in RC07:96. This was not applicable to taxa without postparietals (POSPAR 1-2(2)), but otherwise identical to OPI 2 with inverted state numbers: when the postparietals contact the exoccipitals (POSPAR 5(1)), OPI 2(1) is impossible, and when they do not (POSPAR 5(1)), OPI 2(1) is unavoidable – once the "thickened plate" and the suproccipital are removed from consideration – because nothing other than the opisthotics and/or the suproccipital ever intervenes between the postparietals (or parietals when postparietals are absent) and the exoccipitals.

Merging the present character with SUPOCC 1 is not an option, because *Archeria* (which was correctly scored as having state OPI 2(1), POSPAR 5(0)) possesses huge

opisthotics that separate the postparietals from the exoccipitals, but there is just a narrow unossified slit where a cartilaginous suproccipital may have been (Holmes, 1989: fig. 10A), showing that SUPOCC 1(0) and OPI 2, POSPAR 5(1) can occur together.

State 2 is new and accounts for taxa in which the opisthotics are absent or fused to the exoccipitals, including of course those with completely fused otoccipital parts of the braincase. These are *Eusthenopteron* (Carroll & Chorn, 1995), *Ichthyostega* (reconstruction drawing and photo in Jarvik, 1996, assuming the coarse-grained photo which does not show any sutures can be taken at face value; also suggested by Clack et al., 2003: fig. 3a, c; not mentioned or illustrated by Clack & Milner, 2015), Albanerpetidae (Maddin et al., 2013a), *Eocaecilia*, *Valdotriton*, *Lethiscus* (Pardo et al., 2017), *Oestocephalus*, *Phlegethontia*, and *Notobatrachus* (Estes & Reig, 1973; Báez & Nicoli, 2004, did not mention opisthotics, but mentioned that the exoccipitals usually fuse to the prootics in adults, which implies there were no opisthotics in between) as well as **Liaobatrachus*, **Carrolla* and **Chelotriton*; state 0 or 2 occurs in *Odonterpeton* (CG78: 145f.), **Sclerocephalus* and **Australerpeton*.

State 0 occurs in *Chenoprosopus* (Hook, 1993), Dendrerpetidae (Robinson, Ahlberg & Koentges, 2005), *Pelodosotis* (CG78: fig. 48), apparently *Hyloplesion* (CG78: 137), *Batrachiderpeton* if the opisthotic is correctly identified (Bossy & Milner, 1998: fig. 57B) and *Ariekanerpeton* (made explicit by Klembara & Ruta, 2005a) as well as **Lydekkerina* (Hewison, 2007: 26–27).

Greererpeton was reconstructed as having state 0 by Smithson (1982) based on crushed specimens; less crushed ones have revealed state 1 (Bolt & Lombard, 2001: 1041; Clack, 2003). State 1 is also present in *Acanthostega* and *Whatcheeria* according to the latter paper.

Eoherpeton was scored in RC07 as POSPAR 5(?) (presence of exoccipital/postparietal suture unknown), but OPI 2(1). The latter is correct, except that the presence of a suproccipital cannot be ascertained (Smithson, 1985: 338; already correctly scored as SUPOCC 1(?)); we have therefore scored state 1 of the present character.

We have assigned state 1 to *Batropetes*: although the exoccipitals and the opisthotics fuse dorsally, the suture persists ventrally, and in caudal or dorsal view it seems that the exoccipitals are growing around the foramen magnum much as in later ontogenetic stages of *Acheloma* (Maddin, Reisz & Anderson, 2010).

State 1 also occurs in *Brachydectes* (Pardo & Anderson, 2016) and *Tseajaia* (Moss, 1972).

Ptyonius appears to have state 0 or 1 on account of having separate opisthotics (Bossy & Milner, 1998: 86). The same appears to hold for **Palaeoherpeton* (compare Panchen, 1964: fig. 2, to Clack & Holmes, 1988: 91, fig. 1).

We have scored *Doleserpeton* as possessing state 0 or 2. The postparietals have tall occipital flanges that overlie the opisthotics caudally and reach the exoccipitals (Sigurdsen & Bolt, 2010; Sigurdsen & Green, 2011), corresponding to state 0; however, in the most mature specimens, the dorsal parts of the exoccipitals fuse to the opisthotics (Sigurdsen, 2008; Sigurdsen & Bolt, 2010), raising the question if *Doleserpeton* should be considered to have state 2.

Perhaps similarly, *Diplocaulus* is illustrated as having state 2 by Bossy & Milner (1998: fig. 57C), but Beerbower (1963: 59) reports that "[a]lthough Douthitt reports the exoccipital and opisthotic as fused in *Diplocaulus*, sutures can be distinguished in many specimens of that genus", frustratingly not mentioning if there are any in which fusion can be ascertained. We have scored *Diplocaulus* as having state 0 or 2.

Unknown in *Proterogyrinus* (Holmes, 1984: fig. 4) and *Pederpes* (Clack & Finney, 2005).

136. PASPHE 1: **Cultriform process gradually tapering to a rostral point (0) or parallel-sided along most of its length (1).** This is a rewording of the original name of this character and its explanation; the process cannot help being "elongate", because taxa where it does not reach the vomers are not included in this matrix. State 1 includes cases where the process is biconcave, wider at its rostral end than in the middle.

Ventastega (Ahlberg, Lukševičs & Lebedev, 1994), *Doleserpeton* (Sigurdsen & Bolt, 2010), *Solenodonsaurus* (Danto, Witzmann & Müller, 2012) and *Lethiscus* (Anderson, Carroll & Rowe, 2003) have state 0. State 0 is also found in *Leptorophus tener* (Schoch & Milner, 2008); *L. raischi* does have state 1 (Schoch, 2014a) as scored in RC07, but because it is only known from skeletally less mature (and smaller) specimens than *L. tener*, we have scored *Leptorophus* as having state 0.

Ruta, Coates & Quicke (2003) and Ruta & Coates (2007) scored all PASPHE characters of *Phlegethontia* as unknown (even PASPHE 11, which does not depend on the presence of the parasphenoid). Anderson (2002), however, maintained that the parasphenoid, although indistinguishably fused to the endochondral braincase, is present in *Phlegethontia*: in particular, there is a cultriform process (Anderson, 2002: fig. 4.2, 4.3, 8.2). Ruta, Coates & Quicke (2003) cited Anderson (2002 – as "in press") as their source for several scores of *Phlegethontia*, but did not cite it in any context involving the parasphenoid. We have therefore scored those few parasphenoid characters that do not depend on where the boundaries of the basal plate were. (Comparison to other aïstopods – Pardo et al., 2017 – suggests that the basal plate was much smaller than expected.) For the present character, *Phlegethontia* has state 0.

State 1 is present in *Greererpeton* (Smithson, 1982; D. M., pers. obs. of TMM 41574-1), *Karaurus* (biconcave: Ivachnenko, 1978: fig. 1b!), *Triadobatrachus* (Roček, 2000; Ascarrunz et al., 2016), *Valdotriton* (Evans & Milner, 1996), *Hyloplesion* (Olori, 2015: fig. 30A, table S3) and arguably *Notobatrachus* (Báez & Nicoli, 2004); **Liaobatrachus* has state 0, however (Dong et al., 2013).

Microbrachis is somewhat borderline (Olori, 2015: fig. 10); following the recommendation of Olori (2015: 56), we have scored state 0.

Not described or illustrated in *Whatcheeria* (Lombard & Bolt, 1995; Bolt & Lombard, 2000); unclear in *Hapsidopareion* (CG78: fig. 13A, 14E; Bolt & Rieppel, 2009: 475).

Given the state of preservation of **Quasicaecilia* (Pardo, Szostakiwskyj & Anderson, 2015: fig. 2, 4), we have scored this character as unknown rather than going with the reconstruction (fig. 3, which shows state 0 without indicating any uncertainty) or the statement on p. 12 that the process is "parallel-sided".

137. PASPHE 2-12: **Base of cultriform process and area between basipterygoid processes: no ridges (0); rostromedian-to-caudolateral ridges forming a V (1); state 1 plus a caudal ridge, together delimiting a raised triangular area (2) (ordered).** State 1 is the "anterior wedge-like process" of Klembara & Ruta (2004a), which can hardly be called a process. It occurs in several seymouriamorphs, but so does state 2 contrary to the scoring of RC07.

States 1 and 2 cannot be distinguished when the median depression (PASPHE 6(1)) is too large. This is the case in several taxa that (contrary to the scoring of RC07) clearly do not have state 0, namely *Proterogyrinus* (Holmes, 1984), *Archeria* (Clack & Holmes, 1988), *Pholiderpeton scutigerum* (Clack, 1987b) and *Limnoscelis* (Berman, Reisz & Scott, 2010), and also in **Chroniosaurus* (Clack & Klembara, 2009).

The raised area in state 2 usually bears denticles, and usually the rest of the parasphenoid is then toothless. *Limnoscelis* is an exception where the raised area is rough but apparently toothless (Berman, Reisz & Scott, 2010), and in *Utegenia* the area is almost

toothless while the cultriform process is (otherwise) densely denticulated (Klembara & Ruta, 2004a: fig. 10, 14). RC07 treated the raised area and its dentition as a single character (which may be the reason why they gave our state 1 to *Utegenia*); these are clearly two independent characters, so we have excluded the dentition from consideration. We have, somewhat similarly, assigned state 2 to *Trimerorhachis* where it is not known if a denticle field was present (Milner & Schoch, 2013: 107, fig. 1D), and to **Glanochthon*, where a well-defined triangular denticle field was lost in ontogeny but a well-defined raised area which we count as triangular persisted in adults (Schoch & Witzmann, 2009b: 126, fig. 4C, D). Further, we assign state 2 to *Balanerpeton* (Milner & Sequeira, 1994: fig. 1A).

State 0 is present in *Ventastega* (Ahlberg et al., 2008), apparently *Ecolsonia* (D. M., pers. obs. of CM 38017), *Eocaecilia* (Jenkins, Walsh & Carroll, 2007), *Solenodonsaurus* (Danto, Witzmann & Müller, 2012), seemingly *Kotlassia* (Bulanov, 2003: S54, fig. 30), *Diceratosaurus* (D. M., pers. obs. of several CM specimens), *Lethiscus* (Pardo et al., 2017), *Phlegethontia* (Anderson, 2002: fig. 4.2), *Microphon* (Bulanov, 2003) and *Tseajaia* (Moss, 1972).

Schoch & Milner (2014: fig. 30) reconstructed a condition for *Amphibamus* that may count as state 2, but we count the condition in the most mature known specimen (Daly, 1994: fig. 21 right side) as state 1. State 1 is furthermore found in *Caerorhachis* (Ruta, Milner & Coates, 2002), *Pholiderpeton attheyi* (Panchen, 1972) and *Gephyrostegus* (Klembara et al., 2014).

State 2 is not limited to temnospondyls. Apart from *Utegenia*, it makes surprise appearances in *Pelodosotis* (CG78: fig. 48) and *Rhynchonkos* (Szostakiwskyj, Pardo & Anderson, 2015) and shows up in later ontogenetic stages of *Discosauriscus* (Klembara et al., 2006) as well as its fellow seymouriamorphs **Karpinskiosaurus* (Klembara, 2011) and ***Biarmica* (Bulanov, 2003).

Using a generous interpretation, we have assigned state 2 to **Acanthostomatops*; some specimens fit even under a strict one (Witzmann & Schoch, 2006a). State 2 is also seen in **Platyoposaurus*: although the area is not triangular, all three ridges are present (Eltink et al., 2016: fig. 12B).

Unknown in *Colosteus* (Hook, 1983: 16) and *Anthracosaurus* (Panchen, 1977: 469).

138. PASPHE 3: **Parasphenoid without (0) or with (1) a pair of posterolaterally orientated, ventral thickenings (ridges ending in basal tubera).** This character is only applicable to OTUs whose parasphenoids are long enough caudally, i.e. those that have or may have PASPHE 9(2).

Greererpeton has state 1 (Smithson, 1982; D. M., pers. obs. of TMM 41574-1). So do the largest specimens of *Acheloma* (Olson, 1941; Maddin, Reisz & Anderson, 2010), making the scores of many other temnospondyls suspect. Indeed, MNN MOR 70 appears to have state 1, and so might MNN MOR 69 (D. M., pers. obs.); we have scored state 1 for **Nigerpeton*. State 1 further makes a surprise appearance in **Liaobatrachus* (Dong et al., 2013: fig. 6B).

State 0 is known in Albanerpetidae (Estes & Hoffstetter, 1976; Maddin et al., 2013a). In *Asaphestera*, a partial parasphenoid is preserved in NMC 10041 (D. M., pers. obs. with J. Anderson) and seems to show state 0 as well.

We have assigned state 1 to *Gephyrostegus*, which has very pronounced ridges, although they fade into flat, short triangular processes caudally and basal tubera are absent (Klembara et al., 2014).

Inapplicable to *Phlegethontia* (as already scored), where there is no space for such ridges (Anderson, 2002: fig. 4.2, 4.3).

The drawings and even the photos by Moss (1972) are not three-dimensional enough to show which state *Tseajaia* has, and the text does not mention it; in CM 38033 (D. M., pers.

obs.), most of the palate is covered by the lower jaws, matrix, and miscellaneous fragments; we have therefore scored *Tseajaia* as unknown.

The basal tubera are apparently unknown in **Archegosaurus*, but the ridges are there (Witzmann, 2006).

Incompletely ossified (Maddin, Reisz & Anderson, 2010) but present in **Erpetosaurus* (Milner & Sequeira, 2011: fig. 2C).

139. PASPHE 4: **Parasphenoid without elongate, broad posterolateral processes (0), or with processes that are less than (1), or at least half as wide as (2) the parasphenoid plate (ordered).** We have ordered this character because it is continuous. This character is only applicable to OTUs whose parasphenoids are long enough caudally, i.e. those that have or may have PASPHE 9(2). It is further inapplicable in taxa where the fenestrae ovales/vestibuli are (proportionally) too large to allow anything other than state 0, like Albanerpetidae (Estes & Hoffstetter, 1976; Maddin et al., 2013a; already scored as unknown), *Triadobatrachus* (where, in addition, the margins of the putative fenestra ovalis are unossified: Ascarrunz et al., 2016) or *Hyloplesion* (CG78: fig. 89E, H); similarly, there is no space for processes in *Eocaecilia* (Jenkins, Walsh & Carroll, 2007).

State 0 occurs in *Batropetes* (Glienke, 2013, 2015).

State 1 is found in *Greererpeton* (D. M., pers. obs. of TMM 41574-1), *Edops* (D. M., pers. obs. of USNM 23309), apparently *Cochleosaurus* (Sequeira, 2004), *Balanerpeton* (Milner & Sequeira, 1994), Dendrerpetidae (Robinson, Ahlberg & Koentges, 2005), *Eryops* (Sawin, 1941: pl. 2, 8), *Acheloma* (Olson, 1941; Maddin, Reisz & Anderson, 2010), *Broiliellus* (Schoch, 2012: fig. 2F), *Gephyrostegus* (Klembara et al., 2014), **Nigerpeton* (D. M., pers. obs. of MNN MOR 69 and MNN MOR 70), **Platyoposaurus* (Eltink et al., 2016: fig. 12B) and **Australerpeton* (Eltink et al., 2016: fig. 9A).

Microbrachis has state 2 (Vallin & Laurin, 2004: fig. 4A; Olori, 2015: fig. 12A).

The state is apparently unknown in *Megalocephalus* (Beaumont 1977: 63), *Ecolsonia* (Berman, Reisz & Eberth, 1985: fig. 6) and *Hapsidopareion* (CG78: fig. 13A); it has also not been described or illustrated in *Whatcheeria* (Lombard & Bolt, 1995; Bolt & Lombard, 2000).

**Erpetosaurus* seems to just reach state 2 (Milner & Sequeira, 2011: fig. 2, 5).

140. PASPHE 6: **Parasphenoid without (0) or with (1) single median depression.** Clack et al. (2016) merged this character with the following one (as their ch. 77), and indeed no taxon in our matrix is known to have state 1 of both; it is not clear to us whether such a condition is impossible, however, or whether paired lateral depressions can form by subdivision of a median one (or vice versa), so we prefer to keep these characters separate for now.

Albanerpetidae has state 0 (Estes & Hoffstetter, 1976; Maddin et al., 2013a), as do *Solenodonsaurus* (Danto, Witzmann & Müller, 2012), *Lethiscus* (Pardo et al., 2017) and apparently *Phlegethontia* (Anderson, 2002: fig. 4.2).

State 1 is known in *Broiliellus* (Schoch, 2012: fig. 2F), most likely *Gephyrostegus* (Klembara et al., 2014), *Seymouria* (Laurin, 1996a, 2000) and *Limnoscelis* (Berman & Sumida, 1990) as well as **Platyoposaurus* (Eltink et al., 2016: fig. 12B) and apparently **Coloraderpeton* (Pardo et al., 2017: video).

Inapplicable to *Acanthostega*, where the parasphenoid is not long enough (Porro, Rayfield & Clack, 2015); not described or illustrated in *Whatcheeria* (Lombard & Bolt, 1995; Bolt & Lombard, 2000).

We have scored state 0 for **Diploradus* after Clack et al. (2016: matrix).

141. PASPHE 7: **Parasphenoid without (0) or with (1) paired lateral depressions.**

Cochleosaurus has state 0 (Sequeira, 2004). So do Albanerpetidae (Estes & Hoffstetter, 1976; Maddin et al., 2013a), *Gephyrostegus* (Klembara et al., 2014), *Solenodonsaurus* (Danto, Witzmann & Müller, 2012) and *Lethiscus* (Pardo et al., 2017).

State 1 makes a surprise appearance in **Liaobatrachus* (Dong et al., 2013: fig. 6B).

Inapplicable to *Acanthostega*, where the parasphenoid is not long enough (Porro, Rayfield & Clack, 2015); not described or illustrated in *Whatcheeria* (Lombard & Bolt, 1995; Bolt & Lombard, 2000).

We have scored state 0 for **Diploradus* after Clack et al. (2016: matrix).

142. PASPHE 9: **Intracranial joint (0); ventral cranial suture visible in ventral view, caudal margin of parasphenoid lying rostral to or at it (1); parasphenoid extending caudal to suture (2) (ordered).** We have reworded this character to make clear what state 2, originally "Ventral cranial fissure […] not traceable", means in practice – fusion of the basisphenoid and the basioccipital is rare, and when it occurs, it is hardly ever determinable in a fossil (except by tomography), let alone described or illustrated, but the caudal extent of the parasphenoid is fairly readily observable (except in *Phlegethontia*, which we have kept as unknown even though no trace remains of any cranial fissure). Because the states of this character form a sequence of progressively firmer immobilization of the ventral cranial fissure (which forms part of a joint in *Eusthenopteron* and *Panderichthys*), we have ordered the character.

Ventastega (Ahlberg et al., 2008), *Ichthyostega* (Clack, 2000) and *Lethiscus* (Pardo et al., 2017) show state 1; although apparently not sutured, the fissure of *Lethiscus* is not a joint, so we have not scored state 0.

The suture between basisphenoid and basioccipital is still partially exposed in the smallest specimen of *Acheloma* shown by Maddin, Reisz & Anderson (2010: fig. 4, 5), as well as in one of the largest ones (fig. 4). In those specimens the parasphenoid only covers the median third of the suture; the parasphenoid completely overgrows it in some but not all of the largest specimens (fig. 4, 5). Nonetheless, the definition of state 2 is fulfilled, so we have kept the score of 2.

The braincase is probably altogether unknown in *Gephyrostegus* (Klembara et al., 2014).

Diadectes is polymorphic, having states 1 and 2 (Moss, 1972), with only state 1 being documented in *D. absitus* (Berman, Sumida & Martens, 1998).

Limnoscelis has state 1 or 2 (Berman, Reisz & Scott, 2010: fig. 4A, 10).

Asaphestera (D. M., pers. obs. of NMC 10041 with J. Anderson) and *Pelodosotis* (CG78: fig. 48) have state 2.

Unknown in *Adelospondylus*; *Adelogyrinus* appears to have state 1 or 2 (Andrews & Carroll, 1991).

Pederpes was scored 1 in RC07, but may just as well have had state 0, because the basioccipital is not preserved (Clack & Finney, 2005); we have accordingly scored partial uncertainty.

Tseajaia has state 1 (Moss, 1972: fig. 2, 4A, 15B; pl. 4; and various allusions in the text).

We have scored state 1 for **Pseudophlegethontia*: a line that may be the ventral cranial suture is visible between the two caudal processes of the parasphenoid (Anderson, 2003b: fig. 2A), much like in **Coloraderpeton* (Pardo et al., 2017), and in any case the gap between these two processes extends very far rostral.

143. PASPHE 11: **Basipterygoid processes of the basisphenoid shaped like anterolaterally directed stalks, subtriangular to rectangular in ventral view and**

projecting anterior to the insertion of the cultriform process: absent (0); present (1). This is one of the more unnerving characters: state 1 is a carefully explained combination of states of two characters (shape and orientation of basipterygoid processes) which may or may not correlate – no demonstration of this was attempted by RC07 or Ruta, Coates & Quicke (2003) –, and state 0 comprises everything else, potentially a large number of distinguishable states. More attention will have to be paid to the present character (and to its conditions of applicability) in the future.

Naturally, the basisphenoid is seldom observable; in such cases we have resorted to the parasphenoid (to which the basisphenoid very often fuses indistinguishably).

Microbrachis shows state 1 (Olori, 2015: fig. 12A), as apparently does the larger *Hapsidopareion* individual illustrated by CG78 (fig. 14E). Very mild cases are present in *Platyrhinops* (Clack & Milner, 2010) and *Batropetes* (Glienke, 2013, 2015); we have scored these as state 1 in order to retain this score for *Doleserpeton* (see Sigurdsen & Bolt, 2010: fig. 4) and especially *Karaurus*, which barely counts at best (Ivachnenko, 1978: fig. 1b).

State 0 is present in *Ventastega* (Ahlberg et al., 2008), Albanerpetidae (Maddin et al., 2013a), *Lethiscus* (Pardo et al., 2017) and *Phlegethontia* (Anderson, 2002).

Thoroughly obscured by crushing, breakage and the large fenestrae ovales in *Triadobatrachus* (Ascarrunz et al., 2016: 3D model 1).

Danto, Witzmann & Müller (2012) scored *Solenodonsaurus* as having state 0, while noting in the text that the basipterygoid processes and indeed the basisphenoid as a whole are not preserved; likewise, the pertinent parts of the parasphenoid are unknown (Danto, Witzmann & Müller, 2012: fig. 8A). Clearly, the correct score is unknown (unchanged from RC07). It is likewise unknown in *Scincosaurus* (Milner & Ruta, 2009) and inapplicable to *Brachydectes* (Pardo & Anderson, 2016).

Micraroter and *Cardiocephalus* are both polymorphic (CG78: fig. 30, 52, 55).

A very wide version of state 1 is found in **Liaobatrachus zhaoi* (Dong et al., 2013: fig. 6B – differing greatly from the reconstruction in fig. 7B); **L. macilentus* (fig. 5B), however, has a different shape, so we have scored polymorphism.

**Palaeoherpeton* appears to have a mild case of state 1 (Panchen, 1964: fig. 13).

144. PASPHE 13: **Parasphenoid much wider than long immediately posterior to level of basal articulation: absent (0); present (1).**

State 0 is found in *Ventastega* (Ahlberg et al., 2008), *Platyrhinops* (Clack & Milner, 2010), *Gephyrostegus* (Klembara et al., 2014) and *Lethiscus* (Anderson, Carroll & Rowe, 2003; Pardo et al., 2017).

Acheloma (Polley & Reisz, 2011) and Albanerpetidae (Estes & Hoffstetter, 1976; Maddin et al., 2013a) have state 1. **Nigerpeton* very clearly shares it (D. M., pers. obs. of MNN MOR 69 and MNN MOR 70).

Not applicable to *Phlegethontia* (as already scored in RC07; Anderson, 2002: fig. 4.2, 4.3), **Beiyanerpeton* (Gao & Shubin, 2012: fig. 3) and apparently **Chelotriton* (Schoch, Poschmann & Kupfer, 2015), where the area in question is taken up by the fenestrae ovales.

145. PASPHE 14: **Ventral plate of parasphenoid (caudal to basal articulations) more or less triradiate/triangular (0), rectangular (1), or rectangular with a caudal lobe (2) (unordered).** Dividing this character into states (RC07 distinguished only two: "subrectangular" and everything else) is difficult because such a wide continuum of shapes exists – notably, state 2 includes pentaradiate conditions that can look like part of an octogon, as well as hexaradiate shapes, and state 0 encompasses di-, tri-, and tetraradiate shapes; this diversity of shapes is also why we have not ordered this character.

We have ignored the relatively huge fenestrae ovales of salientians and albanerpetids for the purposes of scoring this character. This allows state 1 in *Triadobatrachus* and *Notobatrachus* (Estes & Reig, 1973; Báez & Nicoli, 2004) and 1 or 2 in Albanerpetidae (Estes & Hoffstetter, 1976; Maddin et al., 2013a). Much smaller excavations of the parasphenoid plate for the fenestrae ovales occur in *Archeria* (Holmes, 1989) and the temnospondyl ***Tersomius* (Carroll, 1964).

State 0: *Greererpeton*, *Crassigyrinus*, *Whatcheeria* (Lombard & Bolt, 1995), *Baphetes*, *Megalocephalus*, *Eucritta* if the reconstruction by Clack (2001) can be trusted, *Isodectes*, *Neldasaurus*, *Anthracosaurus* (Panchen, 1977), *Pholiderpeton scutigerum*, apparently *Gephyrostegus* (Klembara et al., 2014), *Discosauriscus*, *Seymouria*, *Captorhinus*, *Paleothyris*, *Petrolacosaurus*, *Tuditanus*, *Pantylus*, *Saxonerpeton*, *Pelodosotis*, *Cardiocephalus*, *Ariekanerpeton*, *Leptoropha*, *Microphon* (borderline; Bulanov, 2003), *Pederpes*, *Tseajaia* (Moss, 1972: fig. 2), *Utegenia*.

State 1: the four OTUs mentioned above, *Panderichthys* (though the plate is very short), *Edops* (Romer & Witter, 1942; D. M., pers. obs of USNM 23309), *Chenoprosopus* (Langston, 1953), *Cochleosaurus* (Sequeira, 2004), *Trimerorhachis* (Milner & Schoch 2013), *Eryops* (Sawin, 1941), *Phonerpeton* (Dilkes, 1990: fig. 1B), *Ecolsonia* (Berman, Reisz & Eberth, 1985), *Broiliellus* (Schoch, 2012: fig. 2F), *Amphibamus* (Daly, 1994: fig. 21 right side), *Doleserpeton*, *Micromelerpeton*, *Apateon*, *Leptorophus* and *Schoenfelderpeton* (Boy, 1987), *Eoherpeton*, *Proterogyrinus*, *Pholiderpeton attheyi*, *Micraroter*, *Rhynchonkos* (Szostakiwskyj, Pardo & Anderson, 2015), *Brachydectes*, *Scincosaurus*, *Sauropleura*.

State 2: *Platyrhinops* (Clack & Milner, 1994, 2010), *Karaurus*, *Valdotriton*, *Batropetes* (Carroll, 1991; Glienke, 2013; D. M., pers. obs. of MB.Am.1232 contradicting Glienke, 2015: fig. 3F), *Microbrachis* (Olori, 2015: fig. 12A), *Diploceraspis*, *Oestocephalus* (Carroll, 1998a).

Dendrerpetidae is polymorphic, having states 1 and 2 (Godfrey, Fiorillo & Carroll, 1987; Milner, 1996; Robinson, Ahlberg & Koentges, 2005); *Euryodus* is likewise polymorphic, with *E. dalyae* possessing state 2 and *E. primus* displaying state 0.

Acheloma has a condition intermediate between states 0 and 1 (Olson, 1941: fig. 8; Maddin, Reisz & Anderson, 2010: fig. 5I); we have scored partial uncertainty.

Similarly, *Balanerpeton* is reconstructed as intermediate between states 1 and 2 (Milner & Sequeira, 1994); we have scored partial uncertainty.

Unknown (and not already so scored in RC07): *Eusthenopteron* (inapplicable because there only are two caudal processes that are together narrower than at the basal articulation: Ahlberg, Clack & Lukševičs, 1996), *Acanthostega* (inapplicable because the parasphenoid barely extends caudally beyond the basal articulation: Clack, 1994a; Porro, Rayfield & Clack, 2015), *Ichthyostega* (inapplicable because the parasphenoid does not extend caudally beyond the basal articulation at all), *Hapsidopareion* (CG78: fig. 13A, 14E), *Adelospondylus* (Andrews & Carroll, 1991: fig. 13C), *Batrachiderpeton*, *Lethiscus* (the parasphenoid – parabasisphenoid? – is too short and caudally pointed: Pardo et al., 2017) and *Capetus* (Sequeira & Milner, 1993).

Vieraella has state 1 or 2 (Báez & Basso, 1996).

State 1, implied by Damiani et al. (2006: fig. 4B), is probably safe to score for **Saharastega* (D. M., pers. obs. of MNN MOR 73). Following the text of Gubin (1991), we have also assigned state 1 to **Konzhukovia*, though the state shown in drawing 15 is borderline to state 0.

Schoch, Poschmann & Kupfer (2015) figured state 1 in a specimen drawing, but state 2 in two reconstructions; given the state of preservation of the specimens, we have scored partial uncertainty for **Chelotriton*.

146. JAW ART 1/SQU 2/DEN 8: **Jaw articulation lying caudal to (0), level with (1), or rostral to occiput but with rostrodorsally to caudoventrally inclined or vertical caudolateral edge of squamosal (2), or caudolateral edge of squamosal caudodorsally to rostroventrally inclined (3), or even dentary shorter than half of distance between snout and occiput (4) (ordered).** State 3, the previous SQU 2(1), required JAW ART 1(2), and state 4, the previous DEN 8(1) which is limited to *Batropetes* and *Brachydectes* in the original taxon sample, required SQU 2(1), so we have merged these characters. (State 4 is also found in **Carrolla* [Maddin, Olori & Anderson, 2011] and is inevitable in **Quasicaecilia*, where the lower jaw is almost wholly unknown but the distance between the tip of the snout and the jaw joints is less than that between the jaw joints and the occiput [Pardo, Szostakiwskyj & Anderson, 2015].) Because the present character is continuous, we have ordered it and interpreted state 1 as meaning that the distance between the midpoints of the jaw joints and the occipital condyle(s)/cotyle is less than 5% of the distance between the latter and the tip of the snout; this gives state 1 to *Eoherpeton* (Smithson, 1985: fig. 8A), which was originally scored as unknown for JAW ART 1.

RC07 commented JAW ART 1 (their ch. 187) as follows: "There appears to be no clear signal associated with the distribution of different character-states, even within the same clade." Indeed, this character is somewhat labile. Still, states other than 0 occur (in the original taxon sample) only in *Eusthenopteron*, *Eoherpeton*, *Cochleosaurus*, *Trimerorhachis*, *Isodectes* and the seymouriamorph-diadectomorph-amniote-amphibian clade, augmented in the expanded taxon sample only by the temnospondyls **Saharastega*, **Lydekkerina*, **Palatinerpeton* and **Acanthostomatops* and the chroniosuchian **Bystrowiella*; states 2 and 3 are limited to amphibians, *Orobates* and *Isodectes*, and state 0 occurs at least twice in the urocordylid-aïstopod clade. Despite its five states, the present character has only 38 steps on the shortest trees from Analysis R4; reversals from state 1 to 0 are only seen six times, state 2 appears seven or eight times and reverses at most twice, state 3 or higher appears five or six times and reverses at most once, state 4 appears once and never reverses. This is far from a random distribution.

State 0 is found in *Ventastega* (Ahlberg, Lukševičs & Lebedev, 1994; Ahlberg et al., 2008), *Phonerpeton* (Dilkes, 1990), *Eoscopus* (Daly, 1994), *Brukterpeton* (Boy & Bandel, 1973: pl. 7), *Gephyrostegus* (Carroll, 1970: fig. 6A; D. M., pers. obs. of TMM 41773-1), *Westlothiana* (Smithson et al., 1994: text and fig. 2), and *Oestocephalus* (Carroll, 1998a).

Cochleosaurus (Sequeira, 2004), *Trimerorhachis* (Milner & Schoch, 2013), *Lethiscus* (Pardo et al., 2017; J. Pardo, pers. comm.) and *Tseajaia* (Moss, 1972: fig. 2; Berman, Sumida & Lombard, 1992: fig. 9; D. M., pers. obs. of CM 38033) have state 1.

Isodectes (Sequeira, 1998) and *Keraterpeton* (Bossy & Milner, 1998: fig. 57A, 78) show state 2. So does even the largest known skull of *Orobates* (Berman et al., 2004: fig. 8A).

State 3 occurs in Albanerpetidae, *Karaurus*, *Valdotriton* and *Scincosaurus*, as well as in **Beiyanerpeton*.

Batrachiderpeton, *Diplocaulus* and *Diploceraspis* possess state 2 or 3; *Diceratosaurus* has state 3 or 4.

Urocordylus has state 0, 1, or 2.

The condition is entirely unknown in *Dolichopareias* (Andrews & Carroll, 1991).

States 2 and 3 cannot be distinguished in *Phlegethontia* due to the unique shape of the squamosal (Anderson, 2002).

Many taxa go from a higher to a lower state in ontogeny. Accordingly, we have scored *Schoenfelderpeton* as possessing state 0, 1, or 2 (instead of just the observed 2). *Micromelerpeton* (Boy, 1995) and *Apateon* (Schoch & Fröbisch, 2006) are scored 0 based on the most mature specimens. *Leptoropha* and *Microphon* are scored as unknown because the halfway complete skull roofs known of them do not come from adult individuals (Bulanov, 2003).

Gerobatrachus has state 0 or 1 based on the different possibilities for where the occiput could have been in life.

In *Lydekkerina*, the occipital condyles lie rostral to the jaw joints, but the distance is less than 5% of the distance between the jaw joints and the rostral end of the skull (Shishkin, Rubidge & Kitching, 1996; Jeannot, Damiani & Rubidge, 2006); we have scored this as state 1.

State 4 can be excluded for the *St. Louis tetrapod; the other four states remain possible (Clack et al., 2012b: fig. 2A).

Bystrowiella almost certainly falls within the range of state 1 (Witzmann & Schoch, 2017: fig. 15).

147. PSYM 1: **Parasymphysial plate: present (0); absent (1).**

State 1 is found in *Acheloma* (Polley & Reisz, 2011), apparently *Schoenfelderpeton* (Boy, 1986: 154, fig. 15b), *Limnoscelis* (Berman & Sumida, 1990: fig. 5A), *Batropetes* (Glienke, 2013, 2015) and *Notobatrachus* (Báez & Nicoli, 2008).

Kotlassia was scored as unknown in RC07. Ruta, Coates & Quicke (2003) cited Bystrow (1944) as their only source for scoring *Kotlassia*; they did not mention having seen any specimens. Bystrow (1944: fig. 6) very clearly illustrated state 1; unfortunately, as we have not seen specimens either, we have no way of telling to which extent that figure can be trusted on features that are not mentioned in the text. None of the figures of Bystrow (1944) indicate any part of the skull or lower jaw as unknown; there is no indication in the legends or in the illustrations themselves as to which, if any, extent they are reconstructions or specimen drawings. – Because Bystrow (1944: fig. 6) illustrated state 1, the text (p. 389–390) does not explicitly mention any part of the lower jaw as unknown and says that “a complete description of the structural details of the lower jaw” is possible, and RC07 and Ruta, Coates & Quicke (2003) did not indicate any reason for why they scored this character as unknown instead, we have changed the score to 1. We are aware, however, that the skull roof is not as well preserved as Bystrow (1944) drew it (Bystrow, 1944: 409; Bulanov, 2003). – Bystrow (1944) did not distinguish *Kotlassia* from **Karpinskiosaurus*, but used (p. 389) the holotypes of both *Ko. prima* and **Ka. secundus* for describing the lower jaw, and the possibly composite drawing (fig. 6) labeled “Lower jaw of *Kotlassia prima*” shows clear differences to the reconstruction of **Karpinskiosaurus* by Klembara (2011: fig. 9D, E), so we infer that the lower jaw of *Kotlassia* is reasonably well known in general; furthermore, Klembara (2011) did not use the holotype of **Ka. secundus* in his reconstruction of the lower jaw, which he based on two other specimens, leading us to conclude that the holotype does not provide much information on the lower jaw.

For the time being, we accept the interpretation that the lump of bone in *Lethiscus* and **Coloraderpeton* visible not only in dorsal and lingual, but even ventral and labial views is a huge toothless parasymphysial (Pardo et al., 2017) and have therefore scored state 0 for both OTUs. However, we consider it at least as likely that this bone is a mentomandibular (an ossification of the mesial end of Meckel’s cartilage).

We have been very cautious in interpreting the lower jaw of **Carrolla*, in which some fragments are missing and several bones are distorted (Maddin, Olori & Anderson, 2011); for example, we have scored the present character as unknown.

However, we accept the “brassicate structure” at the symphysis of the *St. Louis tetrapod (Clack et al., 2012b; D. M., pers. obs. of MB.Am.1441) as evidence of state 0. Sutures around this “structure” cannot be seen, but sutures cannot be seen almost anywhere else on the specimen either.

148. PSYM 2: **Parasymphysial plate without (0) or with (1) paired fangs, comparable in size with or greater than dentary teeth.**

RC07 cited Ahlberg & Clack (1998) as saying that *Megalocephalus* has state 1, but Milner & Lindsay (1998: 220) stated that it has state 0 and cited a pers. comm. by Ahlberg & Clack. We have accordingly changed the score of *Megalocephalus* to 0.

Lethiscus shares state 0 (Pardo et al., 2017; see PSYM 1).

Bolt & Lombard (2001) stated in table 1 that **Deltaherpeton* (FM PR 1637) lacks parasymphysial fangs like all other colosteids. The text (p. 1036), however, maintains that this cannot be determined. Their fig. 7 does not help, so we have gone with the text rather than the table and have scored **Deltaherpeton* as unknown.

149. PSYM 3: **Parasymphysial plate without (0) or with (1) anteroposterior tooth row oriented subparallel to marginal dentary teeth and the basal diameter and/or height of which [= of the teeth in the parasymphysial toothrow] is 30% or greater than that of marginal teeth and twice or more that of denticles, if present.**

Lethiscus has state 0 (Pardo et al., 2017; see PSYM 1).

Bolt & Lombard (2001) stated in table 1 that **Deltaherpeton* (FM PR 1637) possesses two parasymphysial teeth like all other colosteids. The text (p. 1036), however, maintains that this cannot be determined. Figure 7 does not help, so we have gone with the text rather than the table and have scored **Deltaherpeton* as unknown.

**Ymeria* is probably polymorphic (Clack et al., 2012a).

150. PSYM 4: **Parasymphysial plate with (0) or without (1) small teeth (denticles) forming continuous shagreen or discrete patches and the basal diameter and/or height of which is less than 30% of that of adjacent marginal dentary teeth.**

The resolution of the scans published by Pardo et al. (2017; see PSYM 1) is low enough that we keep this character unknown for *Lethiscus*.

On the only known parasymphysial of **Densignathus*, there is just a single denticle (Daeschler, 2000); because denticles are usually completely absent when they are not "forming continuous shagreen or discrete patches", we have scored state 0.

deleted DEN 1: **Dentary with (0) or without (1) accessory toothrows.** According to RC07, state 0 occurs in *Captorhinus* and *Pantylus*. In fact, *Pantylus* has a single toothrow on the dentary – the additional toothrows (insofar as rows can be recognized) of the lower jaw all lie on the (single) coronoid (Williston, 1916: fig. 27; Romer, 1969: fig. 14; CG78: fig. 25 bottom middle, 25 bottom right, and 114C). This makes the character parsimony-uninformative, so we have deleted it. For the sake of completeness, we would further like to mention that state 0 is limited to one of the three species of *Captorhinus* and autapomorphic for it even if the other clade of captorhinids with multiple toothrows, not sampled in this matrix, is taken into account (Kissel, Dilkes & Reisz, 2002).

"The dorsal edge of the dentary carries a marginal row of closely spaced homodont teeth. Some, but apparently not all, individuals have a secondary row of much smaller teeth outside the principal row" in *Panderichthys* (Ahlberg & Clack, 1998: 14). By comparison to other characters in this matrix, we consider the "smaller teeth" denticles, so *Panderichthys*, originally scored as polymorphic, has only state 1 of this character. Alternatively, it may be that Ruta, Coates & Quicke (2003) intended state 0 to mean the condition of some *Panderichthys* individuals (and a few taxa not included in the matrix of RC07, like the explicitly mentioned **Elginerpeton*: Ahlberg, 1995; Ahlberg & Clack, 1998); but if so, *Captorhinus* could not be counted, and *Pantylus* would have state 1 anyway, rendering the character parsimony-uninformative again (state 0 would be restricted to part of one OTU).

Our addition of ***Elginerpeton* has not rendered this character parsimony-informative if *Panderichthys* is scored as polymorphic, because PAUP* cannot reconstruct ancestors as polymorphic: even if *Panderichthys* and ***Elginerpeton* were sister-groups, two steps (one of them within *Panderichthys*) would be needed to explain the distribution of state 1 regardless of which state their last common ancestor would have had.

151. DEN 2: **Dentary with anterior fangs generally comparable in size with, or greater than, other dentary teeth and lying close to symphysial region and lingual to marginal dentary teeth (0); with fangs/tusks/incisiforms that are part of the marginal toothrow (1); without fangs (2) (unordered).** Instead of "lingual", RC07 had "mesial". The distinction between states 0 and 1 is new; state 1 occurs in *Ichthyostega* (Jarvik, 1996: fig. 31, pl. 31; Clack et al., 2012a: fig. 5C), *Colosteus* (Hook, 1983), *Greererpeton* and ***Deltaherpeton* (Bolt & Lombard, 2001), *Diadectes*, *Limnoscelis*, *Captorhinus*, *Pantylus*, *Stegotretus* and *Orobates*. (The caniniform teeth of some *Oedaleops* specimens [Sumida, Pelletier & Berman, 2014] are too far from the symphysis to count as state 1; we have scored state 2 for *Caseasauria. The same holds for the caniniform region of *Hapsidopareion* (already scored in RC07) and ***Llistrofus* [CG78: fig. 13F; Bolt & Rieppel, 2009].)

Eusthenopteron has state 2 (Ahlberg & Clack, 1998: 15); so do *Triadobatrachus* (Ascarrunz et al., 2016), *Solenodonsaurus* (Danto, Witzmann & Müller, 2012), *Microbrachis* (CG78: fig. 80) and *Scincosaurus* (Milner & Ruta, 2009).

Baphetes has state 0 (Milner & Lindsay, 1998; Milner, Milner & Walsh, 2009), as do *Acheloma* (Polley & Reisz, 2011), *Ecolsonia* (D. M., pers. obs. of CM 38017 and CM 38024), *Eoscopus* (Daly, 1994: 10), *Platyrhinops* (Clack & Milner, 2010), *Schoenfelderpeton* (Boy, 1986: 154, fig. 15b) and *Gephyrostegus* (Klembara et al., 2014).

Archeria shows both state 0 and state 2 in different individuals (Holmes, 1989).

Anthracosaurus appears to have had state 1 and state 2 in different individuals (Panchen, 1977: 475).

Kotlassia was scored as unknown in RC07. Following Bystrow (1944: 392, fig. 6), we have changed the score to 2, but see PSYM 1.

Acherontiscus was scored as having state 0; state 2 is much more likely (Carroll, 1969a).

Unknown in *Leptoropha* (Bulanov, 2003) and *Silvanerpeton* (Ruta & Clack, 2006).

***Glanochthon* has two teeth per dentary that lie lingual to the mesialmost marginal teeth but are only as tall as the smallest (distalmost) marginal dentary teeth. Still, we count this as "generally comparable in size with […] other dentary teeth" and have assigned state 0 to ***Glanochthon*.

We accept the inference of state 0 in ***Erpetosaurus* from the holes in the ventral and the dorsal surface of the premaxillae (Milner & Sequeira, 2011).

Panchen (1975: 619) considered the inference of a tusk in ***Pholidogaster* somewhat uncertain; however, we consider the damage to the roof of the snout (Panchen, 1975: 617; compare Bolt & Lombard, 2010) evidence for state 0 or 1.

***Platyoposaurus* and ***Australerpeton* have states 0 and 1: there are tusks both in the toothrow and lingual to it (Gubin, 1991; Eltink & Langer, 2014).

152. DEN 3: **Dentary without (0) or with (1) 'chamfered' margin.**

State 0 is documented in *Cochleosaurus* (Sequeira, 2004), *Solenodonsaurus* (Danto, Witzmann & Müller, 2012), *Scincosaurus* (Milner & Ruta, 2009) and *Lethiscus* (Pardo et al., 2017), and also known in ***Nigerpeton* (D. M., pers. obs. of MNN MOR 70).

"In some *Ichthyostega* specimens, it [the chamfered margin] seems restricted to the posterior portion of the dentary" (Clack et al., 2012a: 77). This strongly implies that state 1 is always present.

Not applicable to *Phlegethontia*, where all dermal postdentary bones are absent (Anderson, 2002, and references therein).

153. DEN 4: **Dentary without (0) or with (1) U-shaped notch for premaxillary tusks.**

Karaurus has state 0 (D. M., pers. obs. of unnumbered MNHN cast of PIN 2585/2); so do *Solenodonsaurus* (Danto, Witzmann & Müller, 2012) and *Lethiscus* (Pardo et al., 2017).

**Nigerpeton* has a condition similar to but different from state 1 (D. M., pers. obs. of MNN MOR 108); we have scored this as state 0.

We accept the interpretation by Clack et al. (2012b) that the *St. Louis tetrapod has state 1, though the notch is shaped less like a U and more like an asymmetric W; a similar shallow but sharp-edged notch may even be visible in the same position on the other dentary (D. M., pers. obs. of MB.Am.1441.2).

154. DEN 7: **Dentary toothed (0) or toothless (1).**

State 0 is documented in *Chenoprosopus* (Hook, 1993), *Solenodonsaurus* (Danto, Witzmann & Müller, 2012) and *Scincosaurus* (Milner & Ruta, 2009), and also known in **Saharastega* (pers. obs. of MNN MOR 73).

Kotlassia was scored as unknown in RC07. However, Bystrow (1944: 390) stated: "There is a row of conical slightly recuved sharp teeth all along the upper edge of the dentary." We have changed the score to 0; see PSYM 1 for discussion.

155. SPL 2: **Posteriormost extension of splenial lingual lamina closer to anterior margin of adductor fossa than to anterior extremity of jaw, when the lower jaw ramus is observed in me[d]ial aspect and in anatomical connection (i.e. symphysial region orientated towards the observer): absent (0); present (1).** We follow RC07 and many other sources in homologizing the single splenial of amniotes, diadectomorphs and others with the presplenial and not the postsplenial, though we are not aware of any evidence for or against this other than the fact that this single splenial often participates in the symphysis. Unlike all other amniotes, *Petrolacosaurus* was reported to possess a (uniquely small) postsplenial (Reisz, 1981); following a pers. comm. by R. R. Reisz in about 2008, we have kept POSPL 1(0) for *Petrolacosaurus*, but we still wonder if the supposed postsplenial might actually be a fragment of the angular instead.

Whatcheeria has state 1 (Lombard & Bolt, 2006). So do *Limnoscelis* (Reisz, 2007; Berman, Reisz & Scott, 2010), *Batropetes* (Glienke, 2015), *Diplocaulus* (Douthitt, 1917) and *Lethiscus* (Pardo et al., 2017: ext. data fig. 6, contradicting their matrix).

Inapplicable (unknown) in all lissamphibians, because there is no evidence for a splenial in any of them. (The occasional reports from caecilian ontogeny in fact refer to a coronoid: Müller, Oommen & Bartsch, 2005; Müller, 2006.)

Kotlassia was scored as unknown in RC07. Following Bystrow (1944: fig. 6), we have changed the score to 1, but see PSYM 1.

No splenials can be confidently identified in *Rhynchonkos* (Szostakiwskyj, Pardo & Anderson, 2015); we have therefore scored all SPL and POSPL characters as well as PREART 5 (all below) as unknown.

Remarkably, state 0 was scored in RC07 for *Phlegethontia*, which has just two bones in the lower jaw (Anderson, 2002, and references therein): one is the dentary, the other is most likely a purely Meckelian ossification – no lamina and no splenial are remotely identifiable, leaving us no way to apply this character.

It appears (D. M., pers. obs. of MB.Am.1441.2) that the *St. Louis tetrapod had a long distal process of the splenial overlying the prearticular near the dorsomesial edge of the Meckelian fenestra, very similar to what is seen in *Greererpeton* (Bolt & Lombard, 2001: fig. 5.2). We have therefore scored state 1.

156. SPL 3-4: **Splenial separated from anterior and middle coronoids by prearticular (0); splenial contacts anterior but not middle coronoid (1); splenial contacts both anterior (if present) and middle coronoid (2) (ordered).** The splenial never contacts the middle coronoid without also contacting the anterior one (even though the latter contact lies entirely on the symphysial surface in **Platyoposaurus*: Gubin, 1991: drawing 20) unless of course if the anterior one is absent; the ordering reflects a gradual shortening of the prearticular.

We assume that the three denticle-covered areas in the coronoid series of *Platyrhinops* (Clack & Milner, 2010) correspond to the three coronoids; this allows us to keep state 2.

Milner & Schoch (2013: fig. 7B) reconstructed state 0, which was scored in RC07, for *Trimerorhachis* without mentioning this character in the text; following a pers. comm. by R. Schoch (April 2015), however, we have scored *Trimerorhachis* as unknown.

State 0 is, however, found in **Sclerocephalus* (Schoch & Witzmann, 2009a; tentatively confirmed by D. M., pers. obs. of MB.Am.1346) and in the *Parrsboro jaw: the denticulated mystery element that could be part of the prearticular or of the splenial (Sookias, Böhmer & Clack, 2014) appears continuous, except for diagenetic deformation, with the prearticular (the middle part of which has been bent deep into the Meckelian fenestra), and *Caerorhachis* (Ruta, Milner & Coates, 2002) is the only case known to us where the denticle field of the lower jaw extends, just barely, onto the splenial, while in the *Parrsboro jaw a large area is covered by denticles.

Acanthostega has state 1 (Porro, Rayfield & Clack, 2015), as does *Whatcheeria* (Lombard & Bolt, 2006). *Edops* shares state 1, though for an unusual reason: the suture between the anterior and the middle coronoid and the one between splenial and postsplenial are about at the same level, but separated by the posterior coronoid, which has a long suture with the anterior one (D. M., pers. obs. of USNM 23309, impossible to tell in MCZ 1378; not described by Romer & Witter, 1942, not visible in their fig. 3A). This condition (and thus state 1) is also found in *Eryops* (Sawin, 1941), *Pholiderpeton attheyi* (Panchen, 1972), *Anthracosaurus* (Panchen, 1977) and possibly *Proterogyrinus* (compare Holmes, 1984: fig. 15, to Panchen, 1972 and 1977), though we have kept partial uncertainty (state 1 or 2) for the last of these.

Kotlassia was scored as unknown in RC07. Following Bystrow (1944: fig. 6), we have changed the score to 2, but see PSYM 1.

Limnoscelis (Reisz, 2007; Berman, Reisz & Scott, 2010), *Ariekanerpeton* (Klembara & Ruta, 2005: fig. 6D) and *Tseajaia* (Moss, 1972: 19) show state 2 as well. We have further scored state 2 for *Lethiscus*; although the middle coronoid has not been identified, there is a large distance between the mesial end of the prearticular and the distal end of the putative anterior coronoid, all of it filled by the lingual lamina of the splenial (Pardo et al., 2017).

Acheloma has state 1 or 2 (Polley & Reisz, 2011).

**Nigerpeton* has state 1 (D. M., pers. obs. of MNN MOR 70), as does **Saharastega* (pers. obs. of MNN MOR 73).

It appears that the prearticular separates the splenial from the anterior coronoid in **Elginerpeton* (Ahlberg & Clack, 1998: fig. 12B; Ahlberg, Friedman & Blom, 2005: fig. 2B), so we have scored state 0.

Lydekkerina (Jeannot, Damiani & Rubidge, 2006: fig. 4D) and *Australerpeton* (Eltink & Langer, 2014) have a point contact between the splenial, the postsplenial and both pertinent coronoids; we have scored state 1 or 2 for both.

Aytonerpeton appears to have state 0 (Clack et al., 2016: fig. 4c, supplementary video 2).

157. POSPL 1: **Separately ossified postsplenial: present (0); absent (1).**

State 0 is, surprisingly, found in *Diplocaulus* (Douthitt, 1917). It is further said to be present in *Densignathus* (Daeschler, 2000); we have followed this, although the illustrations do not show the suture to the angular (except as a stippled line in reconstructions). *Nigerpeton* (D. M., pers. obs. of MNN MOR 70) and *Saharastega* (D. M., pers. obs. of MNN MOR 73) have state 0 as well.

Batropetes has state 1 (Glienke, 2013). We have also followed Pardo et al. (2017) in scoring it for *Lethiscus*, although fully articulated sutures are at best difficult to identify in their scans; their extended data figure 2 and the accompanying supplementary videos show a failed attempt to identify separate postsplenials (in pink). In *Coloraderpeton*, absence of the postsplenial is indicated by the long distance between the splenial (and its facet) and the angular. Note that all this contradicts the matrix by Pardo et al. (2017), where both *Lethiscus* and *Coloraderpeton* are scored as having two splenials.

Based on the figures and videos of Clack et al. (2016), we tentatively accept state 0 for *Aytonerpeton*.

158. POSPL 2: **Postsplenial without (0) or with (1) lingual lamina.**

Whatcheeria has state 0 (Lombard & Bolt, 2006).

State 1 is just barely found in *Chenoprosopus* (D. M., pers. obs. of USNM 437646) and clearly in *Isodectes* (D. M., pers. obs. of unnumbered MCZ cast of AMNH 6935 before etching), *Doleserpeton* (Sigurdsen & Bolt, 2010: fig. 3B), *Gephyrostegus* (Klembara et al., 2014), *Diplocaulus* (Douthitt, 1917), *Nigerpeton* (D. M., pers. obs. of MNN MOR 70) and *Saharastega* (D. M., pers. obs. of MNN MOR 73).

159. POSPL 3: **Postsplenial with (0) or without (1) pit line.**

State 1 is found in *Whatcheeria* (Lombard & Bolt, 2006), *Ecolsonia* (D. M., pers. obs. of CM 38024), *Doleserpeton* (Sigurdsen & Bolt, 2010: fig. 3B) and apparently *Diplocaulus* (Douthitt, 1917).

Unknown in *Panderichthys* (Ahlberg & Clack, 1998) and *Ossinodus* (Warren, 2007).

160. ANG 1: **Separately ossified angular: present (0); absent (1).**

State 0 is known in *Baphetes* (Milner & Lindsay, 1998: fig. 5; Milner, Milner & Walsh, 2009), *Diceratosaurus* (D. M., pers. obs. of CM 72608), *Diplocaulus* (Douthitt, 1917) and *Lethiscus* (Anderson, Carroll & Rowe, 2003).

State 1 is found in Albanerpetidae (Estes & Hoffstetter, 1976; Gardner, 2001; Venczel & Gardner, 2005).

Unclear in *Triadobatrachus* (Ascarrunz et al., 2016).

Schoch, Poschmann & Kupfer (2015) described a bone in *Chelotriton* that they identified as the surangular. According to their reconstruction (Schoch, Poschmann & Kupfer, 2015: fig. 4f), however, this bone lies unambiguously in the position of an angular, at the ventral edge of the lower jaw, entirely ventral to the prearticular and the articular. “The angular and surangular” are mentioned in the text (p. 82), but there is no “angular” in the illustrations, the list of abbreviations, or anywhere in the text outside the above quote. Personal communication of D. M. with R. Schoch has not so far clarified this matter; for the

time being, we therefore interpret **Chelotriton* as possessing an angular – unique though this is among urodeles, as discussed by Marjanović & Witzmann (2015) – and lacking a surangular; we have scored state 0 of the present character and have also scored **Chelotriton* for other ANG characters based on the supposed "surangular".

Judging from the starburst ornamentation (supplementary video 2), the suture between the angular and the postsplenial of **Aytonerpeton* postulated in fig. 4b of Clack et al. (2016) goes straight through the angular.

161. ANG 2-3: **Angular-prearticular contact: entirely at caudoventral edge of jaw (0); absent, Meckelian bone or cartilage continuously exposed from the jaw joint to the splenial (1); on the lingual side, where the angular has a lingual lamina (2) (unordered).** The contact (explicitly called a suture) and the lingual ("mesial") lamina of the angular were treated as separate characters by RC07, but the latter never seems to occur without the former; we therefore follow Ahlberg, Clack & Blom (2005) and the matrix of Ahlberg et al. (2008), Callier, Clack & Ahlberg (2009) and Clack et al. (2012a) in merging these characters.

State 0 is now known in *Lethiscus* (Pardo et al., 2017).

RC07 claimed that the absence of a contact (ANG 3(1)) was limited to *Acanthostega* in their taxon sample; had that been correct, the character would have been parsimony-uninformative. However, state 1 does occur in *Eusthenopteron* (Zupiņš, 2008: fig. 4) and *Whatcheeria* (Lombard & Bolt, 2006), where the surangular, the angular and the postsplenial are wholly separated from the prearticular by exposed Meckelian bone continuous with the articular, and in *Pederpes*, where the prearticular, "[a]s its ventral border is very simple and delicate, it clearly did not suture with the surangular or angular and possibly not with the postsplenial" (Clack & Finney, 2005). Incidentally, state 1 appears plesiomorphic for Sarcopterygii (Worobjewa, 1975: fig. 3; Long, Barwick & Campbell, 1997: fig. 38; Friedman, 2007: fig. 5).

State 2 is known to occur in *Chenoprosopus* (Hook, 1993; D. M., pers. obs. of USNM 437646, though the sutures are difficult to trace), *Isodectes* (D. M., pers. obs. of unnumbered MCZ cast of AMNH 6935 before etching, though the sutures are nigh impossible to trace), *Doleserpeton* (Sigurdsen & Bolt, 2010; Sigurdsen & Green, 2011: appendix 2), *Gephyrostegus* (Klembara et al., 2014), *Batropetes* (Glienke, 2013, 2015), *Cardiocephalus* (CG78) and *Diplocaulus* (Douthitt, 1917).

Inapplicable in Albanerpetidae, see ANG 1; "always unclear" (Bossy & Milner, 1998: 87) in *Sauropleura* (which was already scored as unknown for ANG 2, but not for ANG 3).

Following Ahlberg, Friedman & Blom (2005), we have scored state 1 for **Elginerpeton*.

There may not be a prearticular in **Coloraderpeton* or **Pseudophlegethontia*: the supposed fusion of the prearticular and the Meckelian bone in the former (Pardo et al., 2017) is probably just Meckelian bone, and the supposed suture at the cranial end of the supposed left prearticular of the latter (absent on the right side; Anderson, 2003b: fig. 2A) could be a break in the Meckelian bone. However, the angular clearly lacks a lingual lamina, so we have scored state 0 or 1 for both.

162. ANG 4: **Angular not reaching (0) or reaching (1) lower jaw posterior end.**

Baphetes has state 0 (Milner, Milner & Walsh, 2009). So do *Chenoprosopus* (Hook, 1993) and *Platyrhinops* (Clack & Milner, 2010).

State 1 is found in *Batropetes* (Glienke, 2015), *Diceratosaurus* (D. M., pers. obs. of CM 72608), *Diplocaulus* (Douthitt, 1917) and *Lethiscus* (Pardo et al., 2017).

Kotlassia was scored as unknown in RC07. Following Bystrow (1944: fig. 6), we have changed the score to 0, but see PSYM 1.

The condition of *Westlothiana* is probably not known, judging from the specimen drawings in Smithson et al. (1994).

163. SURANG 1: **Separately ossified surangular: present (0); absent (1).**

State 0 is documented in *Whatcheeria* (Lombard & Bolt, 2006), *Baphetes* (Milner, Milner & Walsh, 2009), *Batropetes* (Glienke, 2015), *Diceratosaurus* (D. M., pers. obs. of CM 72608), and *Lethiscus* (Anderson, Carroll & Rowe, 2003; Pardo et al., 2017).

Diplocaulus and *Diploceraspis* have state 1; we have, however, kept the scores of SURANG 3 and 5 for the latter and scored the former (which was scored as unknown in RC07 for most of the lower jaw) because at least part of the unitary "articular" must be homologous to the surangular (Douthitt, 1917; Beerbower, 1963: 68, fig. 7).

Schoch, Poschmann & Kupfer (2015) described a bone in **Chelotriton* that they identified as the surangular. According to their reconstruction (Schoch, Poschmann & Kupfer, 2015: fig. 4f), however, this bone lies unambiguously in the position of an angular, at the ventral edge of the lower jaw, entirely ventral to the prearticular and the articular. "The angular and surangular" are mentioned in the text (p. 82), but there is no "angular" in the illustrations, the list of abbreviations, or anywhere in the text outside the above quote. Personal communication of D. M. with R. Schoch has not clarified this matter; for the time being, we therefore interpret **Chelotriton* as possessing an angular – unique though this is among urodeles, as discussed by Marjanović & Witzmann (2015) – and lacking a surangular like all other lissamphibians; we have, in short, scored state 1 of the present character, and have correspondingly scored the other SURANG characters as unknown.

Like Witzmann & Schoch (2017), we infer the presence of the surangular in **Bystrowiella* from the serrated, apparently sutural dorsal margin of the angular.

164. SURANG 3: **Surangular with (0) or without (1) pit line.** Ahlberg, Friedman & Blom (2005) suggested that the surangular pit line was homologous with the oral sulcus of the lateral-line system; this is clearly not the case, because *Eusthenopteron* possesses both (Schultze & Reed, 2012: fig. 10A).

Whatcheeria has state 1 (Lombard & Bolt, 2006), as do *Baphetes* (Milner, Milner & Walsh, 2009), *Batropetes* (Glienke, 2015), *Diplocaulus* (Douthitt, 1917), apparently *Lethiscus* (Pardo et al., 2017; though scored as unknown in their matrix) and *Ossinodus* (Warren, 2007: fig. 8A) – judging from their fig. 7P, the "pit line" mentioned by Warren & Turner (2004: 158) refers to the two pores that are part of the lateral-line system in the surangular figured by Warren (2007: fig. 8A) and do not lie in a position where a pit line would be expected. We have followed the matrix of Pardo et al. (2017) in scoring state 1 for **Coloraderpeton*.

Kotlassia was scored as unknown in RC07. Following Bystrow (1944: fig. 6), we have changed the score to 1, but see PSYM 1.

Unknown in *Westlothiana* where the surface of the bone is unknown (Smithson et al., 1994).

165. SURANG 5: **Surangular lateral exposure much smaller than angular lateral exposure: no (0); yes (1).**

Whatcheeria has state 0 (Lombard & Bolt, 2006), as do *Baphetes* (Milner, Milner & Walsh, 2009), *Batropetes* (Glienke, 2015), *Brachydectes* (Wellstead, 1991; Pardo & Anderson, 2016), *Diplocaulus* (Douthitt, 1917) and *Lethiscus* (Anderson, Carroll & Rowe, 2003; Pardo et al., 2017). Following the illustrations by Berman et al. (2004) against the text, we have also scored *Orobates* as possessing state 0.

State 1 is found in *Cochleosaurus* (Sequeira, 2004) and *Diploceraspis* (Beerbower, 1963: 68).

Kotlassia was scored as unknown in RC07. Following Bystrow (1944: fig. 6), we have changed the score to 0, but see PSYM 1.

166. PREART 5: **Prearticular/splenial suture: present (0); absent (1).** The possible correlations with SPL 3-4 and POSPL 1 remain to be investigated; more states of this character and/or SPL 3-4 should perhaps be distinguished.

State 0 is found in *Whatcheeria* (Lombard & Bolt, 2006), *Diplocaulus* (Douthitt, 1917) and *Lethiscus* (Pardo et al., 2017). We have also scored it for **Coloraderpeton* (Pardo et al., 2017), though there it seems likely that the prearticular is altogether absent rather than fused to Meckel's bone (Pardo et al., 2017: video).

Sigurdsen & Green (2011) reported state 1 in *Doleserpeton*. State 1 also occurs in **Nigerpeton* (D. M., pers. obs. of MNN MOR 70) and **Saharastega* (D. M., pers. obs. of MNN MOR 73).

Cochleosaurus bohemicus was scored in RC07 as having state 0, possibly after the reconstruction of *C. florensis* by Godfrey & Holmes (1995: fig. 5); however, the text (p. 17) and the specimen drawing (fig. 2) make clear that the area in question is not preserved. In her description of the skull of *C. bohemicus*, Sequeira (2004) showed a clear case of state 1 (fig. 12D) in a specimen that appears to be split through the bone, so it may not show the sutures at the lingual surface (fig. 12B); in another specimen, Sequeira (2004: fig. 13) showed a point contact if the dashed suture between the splenial and the postsplenial is taken for granted, did not elaborate on this in the reconstruction (fig. 8C), and mentioned in the text (p. 30) that "[a]n elongate prearticular extends beneath [= ventral to] the short posterior coronoid and continues forward for some distance beneath the middle coronoid, apparently terminating close to the common splenial suture [between splenial and postsplenial] in a subadult specimen (Fig. 13)". We have changed the score of the *Cochleosaurus* OTU to unknown.

Following a pers. comm. by R. Schoch (April 2015), we have scored *Trimerorhachis* as unknown.

167. ANT COR 1: **Separately ossified anterior coronoid: present (0); absent (1).**

Acheloma has state 0 (Polley & Reisz, 2011), as do *Lethiscus* (apparently: Pardo et al., 2017), **Nigerpeton* (D. M., pers. obs. of MNN MOR 70) and **Saharastega* (D. M., pers. obs. of MNN MOR 73).

Hook (1983) stated: "The sutures subdividing the coronoid series cannot be traced and the tusk-bearing anterior coronoid of *Greererpeton* is not preserved [in *Colosteus*]." Nevertheless, we agree with RC07 that all three coronoids were probably present: the mesial end of the coronoid series as preserved, whether complete or not, is very far mesial, easily far enough to belong to the anterior coronoid, while the distal end of the lower-jaw fragment in Hook (1983: fig. 3A) is too far distal to belong to the anterior coronoid by comparison to e.g. *Greererpeton* (Bolt & Lombard, 2001); another lower-jaw fragment shows that the coronoid series reached the adductor fossa (Hook, 1983: fig. 9), so that the posterior coronoid should have been present; and a complete lower jaw (Hook, 1983: fig. 6B) does not show any interruption in the middle of the coronoid series.

State 1 is found in *Limnoscelis* (Reisz, 2007; Berman, Reisz & Scott, 2010) and apparently *Batropetes* (Glienke, 2013, 2015).

Daly (1994: 11) described the situation in *Eoscopus* as follows: "Sharp, recurved denticles cover the dorsomedial part of the jaw and extend from the region of the symphysis to the presumed position of the adductor fossa. The coronoid bones that bear the denticles cannot be described because of their position." To be on the safe side, we have kept only the scores for the presence and the denticles of the posterior coronoid of *Eoscopus*, and have changed all other coronoid characters to unknown.

All ANT COR and MID COR characters as well as POST COR 2, 3 and 4 were scored as unknown in *Kotlassia*. However, Bystrow (1944: 390) described “three toothless coronoids” and added (p. 414): “All endeavors to find traces of teeth on any of the coronoids of *Kotlassia* have yielded no results.” We have scored all eleven characters accordingly; see PSYM 1.

Szostakiwskyj, Pardo & Anderson (2015) identified two coronoids in *Rhynchonkos*, separated by a large gap and a break in the specimen. It remains possible that all three fragments belonged to a single bone (J. Pardo, pers. comm., 2015); however, if they do represent two separate coronoids, it is not clear if the mesial one is the anterior coronoid or the middle coronoid. We have accordingly scored all ANT COR and MID COR characters as unknown.

Douthitt (1917: 17) stated that the anterior and middle coronoids of *Diplocaulus* were most likely absent. Considering how unusually wide the dentary is in dorsal view (Douthitt, 1917: fig. 2.5) and that the area is damaged in all specimens (Douthitt, 1917: 17), we prefer to keep ANT COR 1 and MID COR 1 as unknown: maybe part of the “dentary” actually belongs to a coronoid or several, and the suture is too eroded to remain visible.

Unknown in Dendrerpetidae (Godfrey, Fiorillo & Carroll, 1987; Holmes, Carroll & Reisz, 1998), *Doleserpeton* (Sigurdsen & Bolt, 2010) and *Orobates* (Berman et al., 2004).

We have scored all three coronoids as present in **Ymeria* as suggested by Clack et al. (2012a).

Similarly, while the illustrations of **Densignathus* (Daeschler, 2000) do not show the sutures (except as stippled lines in the reconstructions), the text is so confident about their locations that we have scored all three coronoids as present in **Densignathus*. Daeschler (2000: 304) even stated: “*Coronoids.*—There are three coronoids. The posterior coronoid is the longest and […]” (italics in the original).

The same may hold for **Mordex*, where the sutures are not shown in the specimen drawings (there are no reconstructions) by Milner & Sequeira (2003) or Werneburg (2012a), but the latter (p. 27) stated that all three coronoids bear denticles all over; we have thus scored all three as present, consistent with the fact that the denticle field extends from the adductor fossa to the symphysis (Werneburg, 2012a: fig. 19c).

Clack et al. (2012b: 22) confidently stated that “a fragment of the first coronoid, bearing denticles, is visible” in the *St. Louis tetrapod, but their fig. 2C only labels it “?coronoid”, and no denticles are indicated. It appears (D. M., pers. obs. of MB.Am.1441.2) that the labial (as well as the lingual) margin of the parasymphysial is visible. Labial to it, the ragged edge of the “?coronoid” (Clack et al., 2012b: fig. 2C) actually consists of spikes that may well be denticles, although the pterygoid denticles are hemispherical; this morphology is readily interpretable by comparison to *Greererpeton* (Bolt & Lombard, 2001: fig. 5.2, 5.3). We have thus scored state 0 of the present character and of ANT COR 3.

In **Coloraderpeton* it seems to be impossible to tell if there were sutures in the “long coronoid series” (Pardo et al., 2017: 643, ext. data fig. 6, video); however, the series is so long that we have scored all three coronoids as present (as Pardo et al., 2017, did in their matrix). We have done the same for the similar situations of **Doragnathus* and **Diploradus* (Smithson, 1980; Clack et al., 2016).

“Cor 2 tooth” of **Aytonerpeton* (Clack et al., 2016: fig. 4c) is the fang on the fairly well discernible anterior, not middle, coronoid; we have scored state 0 of this and the next character.

168. ANT COR 2: **Anterior coronoid with (0) or without (1) fangs comparable in size to or larger than marginal dentary teeth.**

State 0 makes a surprise appearance in *Leptorophus* (Boy, 1986: 144, fig. 7b).

Acheloma (Polley & Reisz, 2011), *Platyrhinops* (Clack & Milner, 2010), *Lethiscus* (Pardo et al., 2017), **Nigerpeton* (D. M., pers. obs. of MNN MOR 70) and **Saharastega* (D. M., pers. obs. of MNN MOR 73) have state 1.

Best scored as unknown in *Colosteus*, where the pertinent part of the anterior coronoid may have broken off (Hook, 1983).

The part of the coronoid where the fangs would be expected is not preserved in MB.Am.1441 (D. M., pers. obs.), unless a mysterious multipartite hole is part of an alveolus; we have scored the *St. Louis tetrapod as unknown.

169. ANT COR 3: **Anterior coronoid with (0) or without (1) small teeth (denticles) forming continuous shagreen or discrete patches and the basal diameter and/or height of which is less than 30% of that of adjacent marginal dentary teeth.**

Clack et al. (2016: 6) wrote: "Some colosteids lack coronoid teeth, and instead bear shagreen, a variable condition among individuals", citing Bolt & Lombard (2001), who in fact reported no such individual variation, indeed no variation at all between the specimens they studied except for the presence of "denticles" on the middle coronoid in **Deltaherpeton* and their absence in *Greererpeton* (Bolt & Lombard, 2001: table 1: ch. 89); "teeth" on the middle coronoid are present in both taxa (ch. 87), and in both taxa the "smallest teeth" on that bone are arranged in more than one row (ch. 88). While RC07 used 30% of the size of the marginal teeth as the cutoff point between "teeth" and "denticles", Bolt & Lombard (2001) used 10%. Accepting, like RC07, the inference (Bolt & Lombard, 2001, based on comparison to **Deltaherpeton*) that *Greererpeton* had both middle and posterior coronoids, we find that RC07 scored the denticles and toothrows of *Greererpeton* accurately except for missing the rows of very small teeth, which nonetheless seem to count as such under their/our definition, on the middle and posterior coronoids (see MID COR 4 and POST COR 4 below).

We have kept state 1 for the toothrow of *Colosteus*; although the teeth are small enough to count as denticles when compared to the dentary teeth, many or all of the upper marginal teeth would be denticles by comparison to the dentary teeth as well. Because the dentary teeth of *Colosteus* are unusually large, we have ignored the size criterion in this instance.

State 0 is known in *Acheloma* (Polley & Reisz, 2011), *Platyrhinops* (Clack & Milner, 2010) and **Saharastega* (D. M., pers. obs. of MNN MOR 73).

Whatcheeria has state 1 (Lombard & Bolt, 2006), as does *Trimerorhachis* (Milner & Schoch, 2013).

Eryops is polymorphic (Werneburg, 2007b).

The coronoid teeth of **Sigournea*, **Diploradus* and arguably **Doragnathus* are (just barely) large enough not to be considered denticles (Bolt & Lombard, 2006: fig. 2; Clack et al., 2016: fig. 3d; Smithson, 1980: fig. 2); we have scored absence of denticles and presence of a toothrow (see below) for all three OTUs and all three coronoids, even though **Diploradus* and, for a short stretch on the middle coronoid, **Sigournea* have two rows instead of the usual one.

170. ANT COR 4: **Anterior coronoid with (0) or without (1) anteroposterior tooth row orientated sub-parallel to marginal dentary teeth and the basal diameter and/or height of which is 30% or greater than that of marginal teeth and twice or more that of denticles, if present.**

State 1 is now documented in *Trimerorhachis* (Milner & Schoch, 2013), *Acheloma* (Polley & Reisz, 2011), *Platyrhinops* (Clack & Milner, 2010) and *Lethiscus* (Pardo et al., 2017) and is also preserved in **Nigerpeton* (D. M., pers. obs. of MNN MOR 70) and **Saharastega* (D. M., pers. obs. of MNN MOR 73).

Jeannot, Damiani & Rubidge (2006: 831) stated that **Lydekkerina* has state 0; Hewison (2007: 35) quoted that, but illustrated state 1 in fig. 34. Given the polymorphism that **Lydekkerina* shows in many other characters (such as POST COR 4), we have tentatively scored polymorphism here as well.

171. MID COR 1: **Separately ossified middle coronoid: present (0); absent (1).**

Baphetes has state 0 (Milner, Milner & Walsh, 2009), as do **Nigerpeton* (D. M., pers. obs. of MNN MOR 70) and **Saharastega* (D. M., pers. obs. of MNN MOR 73).

Batropetes apparently has state 1 (Glienke, 2013, 2015), as does *Orobates* (Berman, Reisz & Scott, 2010).

Unknown in Dendrerpetidae (Godfrey, Fiorillo & Carroll, 1987; Holmes, Carroll & Reisz, 1998); see ANT COR 1 for *Diplocaulus*.

Pardo et al. (2017) reconstructed only one coronoid (here interpreted as the anterior one, see above) in *Lethiscus*, followed distally by a long gap. This gap may of course have been filled in life by further coronoids which may well be too crushed to identify in the CT scan; we have therefore kept the middle and the posterior coronoid as unknown. Note that Pardo et al. (2017) scored *Lethiscus* as having three coronoids without discussing this decision.

172. MID COR 2: **Middle coronoid with (0) or without (1) fangs comparable in size to or larger than marginal dentary teeth.**

State 0 makes a surprise appearance in AMNH 4565, the type specimen of *Trimerorhachis insignis* (D. M., pers. obs.), and most likely in AMNH 4572. This was scored as MID COR 4(0) in RC07, but does not fulfill its definition: the fang is not larger than the marginal teeth. – At the same time, Milner & Schoch (2013) strongly implied the absence of fangs in a different specimen; we have scored *Trimerorhachis* as polymorphic. The mention of “no coronoid fangs” in their diagnosis of *Trimerorhachis* (p. 115) may assume a different definition of “fang” than the strictly size-based one used by RC07.

Accepting, like RC07, that the middle coronoid is present in *Colosteus* (see ANT COR 1 above), state 1 is strongly suggested by Hook (1983: fig. 3A, 6B).

Balanerpeton (Milner & Sequeira, 1994), *Doleserpeton* (Sigurdsen & Bolt, 2010), **Nigerpeton* (D. M., pers. obs. of MNN MOR 70) and **Saharastega* (D. M., pers. obs. of MNN MOR 73) have state 1 in any case.

Ahlberg & Clack (1998: fig. 10A) explicitly indicated a pair of fangs on the middle coronoid of **Elginerpeton*, but stated in the text (p. 27): “The middle and posterior coronoids do not carry distinct fang pairs.” Because not only the two teeth indicated in the figure, but the entire toothrow (MID COR 4) would count as fangs under the definition used here, we have scored state 0; we have scored POST COR 2 as unknown, because the part of the posterior coronoid where fangs would be expected is not known (Ahlberg, Friedman & Blom, 2005).

173. MID COR 3: **Middle coronoid with (0) or without (1) small teeth (denticles) forming continuous shagreen or discrete patches and the basal diameter and/or height of which is less than 30% of that of adjacent marginal dentary teeth.**

Baphetes has state 0 (Milner, Milner & Walsh, 2009). So do *Trimerorhachis* (Milner & Schoch, 2013; D. M., pers. obs. of AMNH 4565) and **Saharastega* (D. M., pers. obs. of MNN MOR 73).

Eryops is polymorphic (Werneburg, 2007b).

"The middle coronoid of *Doleserpeton* has either large denticles or small teeth", so Sigurdsen & Green (2011: appendix 2: 14) recommended to score it as unknown; we have followed this.

174. MID COR 4: **Middle coronoid with (0) or without (1) anteroposterior tooth row orientated subparallel to marginal dentary teeth and the basal diameter and/or height of which is 30% greater than that of marginal teeth and twice or more that of denticles, if present.**

State 0 is apparently found in *Greererpeton*, in that some of the teeth in the row fulfill the size criterion (Bolt & Lombard, 2001: fig. 1, 2, 5). We have also scored it in *Colosteus* (Hook, 1983: fig. 3A, 6B; see ANT COR 1, ANT COR 3 and MID COR 2 above).

Trimerorhachis has state 1, see MID COR 2. So do *Balanerpeton* (Milner & Sequeira, 1994), **Nigerpeton* (D. M., pers. obs. of MNN MOR 70) and **Saharastega* (D. M., pers. obs. of MNN MOR 73).

"The middle coronoid of *Doleserpeton* has either large denticles or small teeth", so Sigurdsen & Green (2011: appendix 2: 14) recommended to score it as unknown; we have followed this.

175. POST COR 1: **Separately ossified posterior coronoid: present (0); absent (1).**

Baphetes has state 0 (Milner, Milner & Walsh, 2009), as do *Trimerorhachis* (Milner & Schoch, 2013; D. M., pers. obs. of AMNH 4565), *Brachydectes* (Pardo & Anderson, 2016) and likely *Diplocaulus* (Douthitt, 1917: 17), although we prefer to keep all POST COR characters unknown for the latter.

Batropetes apparently has state 1 (Glienke, 2013, 2015).

The coronoid of *Pantylus* is so large that it may well represent a fusion of all three coronoids, or the crowded teeth may simply obscure the sutures between two or three coronoids; but because it has not been possible to trace sutures (Romer, 1969: 24; CG78) and because the coronoid participates in the coronoid process, we here homologize it with the posterior coronoid and have scored the other two coronoids as absent (keeping the scores of RC07 for ANT COR 1, MID COR 1 and POST COR 1).

The evidence for any coronoids in **Sparodus* is limited to a pair of tusks (Fig. 4; Carroll, 1988) and a probable suture between the bone that bears them and the dentary (D. M., pers. obs. of NHMW 1899/0003/0006; Fig. 4). Parsimoniously, we assume that these tusks are homologous to those of *Pantylus*, so we have scored **Sparodus* as possessing a posterior coronoid and fangs on it (see POST COR 2 below) and have scored all other coronoid characters as unknown.

As *Stegotretus* had coronoid tusks that look homologous to those of *Pantylus* and **Sparodus* (see POST COR 2), we have assigned state 0 to *Stegotretus* as well.

It is not clear which coronoid(s) is/are preserved in the *Goreville microsaur (Lombard & Bolt, 1999), but the posterior one is probably the safest bet because it is the most common one to occur in complete specimens of other OTUs.

Similarly, *Diceratosaurus* has two toothrows in each lower jaw ramus, and sutures are not visible (D. M., pers. obs. of CM 29593, CM 29876 and CM 34668); because they extend equally far caudally, we assign the lingual one to the posterior coronoid.

176. POST COR 2: **Posterior coronoid with (0) or without (1) fangs comparable in size to or larger than marginal dentary teeth.**

RC07 copied the scores of Ahlberg & Clack (1998), but used a different definition of "fangs". Ch. 15 of Ahlberg & Clack (1998: 43) reads: "Fangs on posterior coronoid recognisable because much bigger than marginal teeth: yes (0); no (1)." Under the definition

of RC07, *Ventastega* has state 0, because the largest two alveoli on the posterior coronoid are much larger than most and larger than most or all alveoli on the dentary (Ahlberg & Clack, 1998: fig. 14B), in addition to being much larger than all other alveoli on the posterior coronoid itself. They are part of the toothrow on that bone, but this is also the case for the considerably larger fang pairs on the other two coronoids, which were scored as fangs.

State 0 makes a surprise appearance in AMNH 4565, the type specimen of *Trimerorhachis insignis* (D. M., pers. obs.): the caudalmost member of the denticle field reaches the size of a dentary tooth. This is not apparent from the illustration of this specimen – or at least a specimen with the same number – by Milner & Schoch (2013: fig. 7C), where the entire field is only shown as black spots symbolizing alveoli, or in their reconstruction (fig. 7B). As explained under MID COR 2, we have scored *Trimerorhachis* as possessing state 0.

Boy (1986: fig. 15b) showed one tooth on the posterior coronoid of *Schoenfelderpeton*; he called it “a large denticle” on p. 154, but added that it is “not smaller than the dentary teeth”. It is thus large enough to count as state 0 of this character. Unfortunately it is not clear if the preservation of that specimen is good enough to rule out a toothrow (POST COR 4(0)), but in the drawing the sutures between the dentary, the posterior coronoid and the prearticular are shown as unbroken lines well anterior (mesial) to the tooth, implying that the space where a toothrow would have one or two alveoli is preserved and does not bear teeth. We have scored state 0 for *Schoenfelderpeton*.

State 0 is moreover found in *Pantylus*, *Stegotretus* and **Sparodus* (see POST COR 1 above).

Whatcheeria (Lombard & Bolt, 2006) and *Doleserpeton* (Sigurdsen & Bolt, 2010) have state 1, as do *Brachydectes* (Pardo & Anderson, 2016) and *Diceratosaurus* (see POST COR 1).

Under the present definition, at least the first two teeth on the posterior coronoid of **Densignathus* are fangs (Daeschler, 2000: fig. 2).

We have scored **Elginerpeton* as unknown (see MID COR 2), although most of the toothrow (POST COR 4) counts as fangs under the definition used here.

177. POST COR 3: **Posterior coronoid with (0) or without (1) small teeth (denticles) forming continuous shagreen or discrete patches and the basal diameter and/or height of which is less than 30% of that of adjacent marginal dentary teeth.**

Baphetes has state 0 (Milner, Milner & Walsh, 2009). So do Dendrerpetidae (Godfrey, Fiorillo & Carroll, 1987) and *Pantylus* (see POST COR 1 above).

Doleserpeton (Sigurdsen & Bolt, 2010), *Diadectes* (Berman, Sumida & Martens, 1998: fig. 11), *Rhynchonkos* (Szostakiwskyj, Pardo & Anderson, 2015) and *Brachydectes* (Pardo & Anderson, 2016) show state 1.

Colosteus may have a double toothrow like **Diploradus*; whether additional denticles were present is not clear from the published drawing (Hook, 1983: fig. 9). Ignoring the size criterion (see ANT COR 3 above), we have therefore scored *Colosteus* as unknown.

178. POST COR 4: **Posterior coronoid with (0) or without (1) anteroposterior tooth row orientated sub-parallel to marginal dentary teeth and the basal diameter and/or height of which is 30% or greater than that of marginal teeth and twice or more that of denticles, if present.**

Greererpeton has a row of three fairly large teeth and thus state 0 (Bolt & Lombard, 2001).

Colosteus lacks that row, but may have a double row of smaller teeth like **Diploradus* (Hook, 1983: fig. 9; see POST COR 3 immediately above); we have scored it as unknown.

Trimerorhachis (Milner & Schoch, 2013; D. M., pers. obs. of AMNH 4565) and *Doleserpeton* (Sigurdsen & Bolt, 2010) have state 1, as do *Anthracosaurus* (Panchen, 1977: fig. 10), *Limnoscelis* (Reisz, 2007; Berman, Reisz & Scott, 2010), *Rhynchonkos* (Szostakiwskyj, Pardo & Anderson, 2015) and *Brachydectes* (Pardo & Anderson, 2016).

Pantylus has state 0 (Romer, 1969; CG78 – at least the labialmost seven teeth may be considered a row), as do *Batrachiderpeton* (Bossy & Milner, 1998 – two rows surrounding a denticle field) and *Diceratosaurus* (see POST COR 1 above).

**Glanochthon* is polymorphic, with **G. latirostris* having state 1 and **G. angusta* having state 0 (Boy, 1993; Schoch & Witzmann, 2009b).

**Lydekkerina* appears to be polymorphic as well (Hewison, 2007).

179. POST COR 5-7: **Posterior coronoid without posterodorsal process (0); with a process that does not contribute to the tallest point of lateral margin of adductor fossa (1); or with a process that does (2) (ordered).** This is a continuous character (gradual enlargement of the posterodorsal process).

Whatcheeria has state 2 (Lombard & Bolt, 2006), and so do *Isodectes* (D. M., pers. obs. of unnumbered MCZ cast of AMNH 6935 before etching), *Ecolsonia* (D. M., pers. obs. of CM 38024), *Doleserpeton* (Sigurdsen & Bolt, 2010: fig. 3C), *Brachydectes* (Pardo & Anderson, 2016) and **Nigerpeton* (D. M., pers. obs. of MNN MOR 70).

Cochleosaurus has state 1 (Sequeira, 2004), as do *Limnoscelis* (Reisz, 2007) and *Rhynchonkos* (Szostakiwskyj, Pardo & Anderson, 2015).

State 0 is found in *Trimerorhachis* (Milner & Schoch, 2013; D. M., pers. obs. of AMNH 4565).

Gephyrostegus has state 1 or 2 (Klembara et al., 2014: fig. 3, 6).

We follow the text (p. 509) rather than the label in fig. 4D of Schoch & Rubidge (2005) in scoring state 2 for **Micropholis*.

Hewison (2007) claimed state 0 for **Lydekkerina*. The accompanying reconstruction drawing (Hewison, 2007: fig. 34), however, shows state 2, even though the process is very low.

The lower jaw of **Neopteroplax* is so strongly C-shaped that the meaning of "tallest point" is unclear. Measuring more or less along the curve, we have scored state 2, in keeping with the fact that the surangular crest is unusually low for an anthracosaur (Romer, 1963: 429, fig. 6).

180. POST COR 6: **Posterior coronoid exposed in lateral view: no (0); yes (1).**

Baphetes has state 0 (Milner, Milner & Walsh, 2009). So do *Edops* (as far as D. M. can tell from pers. obs. of MCZ 1378, where the area is partly covered by upper-jaw fragments), *Cochleosaurus* (Sequeira, 2004), *Limnoscelis* (Reisz, 2007; Berman, Reisz & Scott, 2010), *Rhynchonkos* (Szostakiwskyj, Pardo & Anderson, 2015) and *Tseajaia* (Berman, Reisz & Scott, 2010).

Phonerpeton (D. M., pers. obs. of MCZ 1414), *Ecolsonia* (D. M., pers. obs. of CM 38024), *Doleserpeton* (Sigurdsen & Bolt, 2010: fig. 3C), *Pantylus* (Romer, 1969: 24), *Brachydectes* (Pardo & Anderson, 2016), *Orobates* (Berman, Reisz & Scott, 2010) and **Nigerpeton* (D. M., pers. obs. of MNN MOR 70) show state 1.

We follow the text (p. 509) rather than the label in fig. 4D of Schoch & Rubidge (2005) in scoring state 2 for **Micropholis*.

181. ADD FOS 1: **Adductor fossa facing dorsally (0) or lingually (1).** This is actually a continuous character, and it is not clear where the line is supposed to be drawn. Fairly much is visible of the adductor fossa of *Whatcheeria* in medial view, yet Lombard & Bolt (2006: 26f.)

stated that “[t]he adductor fossa opens mostly dorsally, as in other early tetrapods and the osteolepiform sarcopterygians related to them”, in other words counting *Whatcheeria* as possessing state 0 (as scored by RC07).

Even so, state 1 is found in *Greererpeton* (Bolt & Lombard, 2001), *Baphetes* (Milner, Milner & Walsh, 2009), *Doleserpeton* (Sigurdsen & Bolt, 2010), *Lethiscus* (Pardo et al., 2017 – contrary to their matrix), and *Eocaecilia* (Jenkins, Walsh & Carroll, 2007), although we wonder if the latter should be scored as inapplicable because its subtemporal fenestra faces labioventrally rather than just ventrally.

Yet, state 0 occurs in Albanerpetidae (*Albanerpeton inexpectatum*: Estes & Hoffstetter, 1976: pl. VII; Gardner, 2001: fig. 5), *Batropetes* (Glienke, 2013, 2015), *Pantylus* (Romer, 1969: fig. 15), *Rhynchonkos* (Szostakiwskyj, Pardo & Anderson, 2015: fig. 5D – the labial rim is drawn out into a tall crest, but the lingual rim is straight rather than embayed ventrally), *Brachydectes* (Pardo & Anderson, 2016), *Batrachiderpeton* (Bossy & Milner, 1998), *Diplocaulus* (Douthitt, 1917), **Carrolla* (Maddin, Olori & Anderson, 2011) and **Chelotriton* (Schoch, Poschmann & Kupfer, 2015).

Following the reconstructions in CG78: fig. 114, we have also tentatively assigned state 0 to *Hapsidopareion* (which was scored as unknown in RC07), *Micraroter*, *Cardiocephalus* and *Euryodus*, where the labial rim of the adductor fossa is much taller than the lingual one only because of the tall coronoid process, while the lingual rim is more or less on the same level with the articular and the toothrow.

Bystrow (1944: fig. 6) showed state 0 in *Kotlassia* as well. Note that this is not shared by **Karpinskiosaurus* (Klembara, 2011: fig. 9D, E).

Unknown in *Stegotretus* (Berman, Eberth & Brinkman, 1988).

**Lydekkerina* appears to change from state 1 (Jeannot, Damiani & Rubidge, 2006: fig. 4D) to state 0 as seen in *Whatcheeria* (Hewison, 2007: fig. 34) in ontogeny. We have scored state 0.

182. TEETH 1: **Marginal tooth pedicely at any point in ontogeny: absent (0); present (1).** This character is usually difficult to score in small animals unless sections and electron micrographs are made – or unless the tooth crowns have fallen off post mortem, leaving the pedicels in place (a very common occurrence in lissamphibian fossils). When the tooth crowns are in place, ambiguity is common. As an example, there is a consensus (Clack & Milner, 2010: 288, fig. 6, 8; and references therein) that *Platyrhinops* shows state 0 (as scored by RC07 – Pardo, Small & Huttenlocker [2017: supplementary information part E] mentioned state 1 in passing, but this is an error). Yet, the lingual side of CM 72646 (a natural mold of the mesial part of a lower jaw; D. M., pers. obs.) has two mesiodistal breaks, one distal to the other, that run through several teeth each, giving the impression of pedicely at first glance. These breaks, however, continue through the matrix between the teeth and have crooked, jagged, parallel edges instead of straight or rounded smooth ones. They probably follow, more or less, the labial jaw margin, which is much taller than the lingual one; the labial side of the specimen shows no breaks and preserves only the tips of the teeth. In a smaller specimen, where the edges of the breaks would be more difficult to see and/or there would be less space between the teeth, it might not be possible to determine whether the teeth were pedicellate.

Most OTUs that are potentially relevant to lissamphibian origins remain to be investigated.

We have added the specification about ontogeny because pedicely often appears during lissamphibian ontogeny and is commonly absent in neotenic salamanders.

Bolt (1979: 545) wrote about the teeth of *Amphibamus*: “Some appear to be pedicellate (see Fig. 8 and discussion below), although it is difficult to be sure of this on the basis of a cast.” The cited fig. 8 shows what looks at least as much like a break as like an

unmineralized zone. We therefore follow Sigurdsen & Bolt (2010) and Sigurdsen & Green (2011: appendix 2) in scoring *Amphibamus* as unknown.

Apateon and *Schoenfelderpeton* were scored as unknown, but the only published report of pedicely in any branchiosaurid specimen (*Apateon*: Schoch & Carroll, 2003) looks very much like a preservational artefact to us. We have therefore scored both taxa as having state 0.

We have kept state 0 for *Diceratosaurus*, but it is noteworthy that each of its teeth is ringed by a constriction near the tip; postmortem breakage at this point, while by no means universal within an individual, appears to be common (D. M., pers. obs. of several CM specimens). Indeed, all marginal teeth of MB.Am.778 (D. M., pers. obs.) are broken at what seems to be the same height, making at least the anatomical left side look like most jaw specimens of fossil lissamphibians or *Doleserpeton*.

The mode of preservation suggests state 0 in *Lethiscus* and **Coloraderpeton* (Pardo et al., 2017).

Carroll (1998a: fig. 4B, 8A) illustrated a dentary and maxillae of *Oestocephalus* where many teeth appear to be broken at the same level, as if the crowns had fallen off of pedicels, and mentioned this similarity on p. 158. We have scored *Oestocephalus* as unknown, even though other specimens (such as CM 29596, CM 29891 and CM 68353; D. M., pers. obs.) do not show evidence of pedicely.

Germain (2008b) cautiously suggested on the basis of electron micrographs that the teeth of *Phlegethontia* could be pedicellate, interpreting two possible breaks as the separation between tip and pedicel and between tooth and jawbone. We have therefore scored it as unknown.

Notobatrachus has state 0 (Báez & Nicoli, 2004), a likely reversal also found in the **pipoids and ***Ascaphus*. We have further assigned state 0 to **Liaobatrachus*, where all tooth crowns appear to be preserved and there is no evidence for pedicels (figures in Dong et al., 2013), although Dong et al. (2013), surprisingly, did not mention this question at all.

Anderson et al. (2008a) reported pedicely in **Gerobatrachus*. However, as pointed out previously (Marjanović & Laurin, 2008), not one of the teeth is preserved as a lone pedicel – there are only complete teeth and empty alveoli. Given the facts that the resolution of the photo (fig. 3a) is too low to tell, that we have not seen the specimen (the forthcoming detailed description by Anderson et al. will doubtless provide additional information) and that the single known specimen does not provide ontogenetic information, we have scored it as unknown, even though pedicellate teeth would not be surprising in a close relative of *Doleserpeton* (where they are indeed present, as scored by RC07 [Bolt, 1991: fig. 4; Sigurdsen & Bolt, 2010]) – and even though Sigurdsen & Bolt (2010), who had seen the specimen, considered it to lack pedicely. – Very recently, Pardo, Small & Huttenlocker (2017: supplementary information part E) reported that "[p]edicellate morphology, but no clear dividing zone between pedicel and crown, is seen in […] *Gerobatrachus*", which – in the absence of ontogenetic information – confirms that the correct score is unknown.

Although most tooth crowns are not preserved, **Saharastega* has state 0 (D. M., pers. obs. of MNN MOR 73).

We interpret the complete teeth and empty alveoli of **Sparodus* (Carroll, 1988) as the unsurprising absence of pedicely in this "microsaur".

We follow Maddin, Olori & Anderson (2011) in interpreting the teeth of **Carrolla* as possibly pedicellate, i.e., unknown for this character.

One of the two lower jaws known from **Diploradus* was not figured by Clack et al. (2016), so we do not know if it preserves complete teeth, which are shown in the reconstruction drawing (bottom of fig. 3d). The lower jaw of the type specimen, drawn above

the reconstruction, seems to show all teeth broken at a similar level. We have scored the present character (as well as the other tooth-tip characters: TEETH 3, 6, 7, 10) as unknown.

183. TEETH 3: **Maximum number of labiolingually arranged cusps per tooth at any point in ontogeny: one cusp (0); a ridge (1); two or three separate cusps (2) (ordered).** We have ordered this partially meristic, partially continuous character.

The original wording was: “Marginal teeth without (0) or with (1) two cuspules labiolingually arranged.” RC07 went on to state that the mesiodistally arranged cusps of *Batropetes* and Albanerpetidae “cannot be treated as an alternative state, as they are not readily comparable”. The two OTUs in question were scored 0. We do not understand why this condition was not treated as a third state of the same character (as done by Marjanović & Laurin, 2008); however, given the diversity of cusp arrangements among the OTUs of this matrix (especially the OTUs we have added), we have instead split this character, treating the number of labiolingually (TEETH 3) and mesiodistally arranged cusps (the new character TEETH 10) separately. Taxa that have two or more cusps in both directions do not occur in this matrix, but exist – “HOMO *nofce Te ipsum*”, as Linnaeus (1758) wrote (“**human, learn to know yourself”; capitals and italics in the original).

State 1 occurs in “cf. *Broiliellus* sp.” according to Bolt (1977). Because Carroll (1964) explicitly reported state 0 for *Broiliellus brevis*, we think that the specimen Bolt described does not belong to the latter species, for which we have therefore kept the score of 0. State 1 is, however, found in *Cardiocephalus* (Anderson & Reisz, 2003; Anderson, 2007b); as Anderson & Reisz (2003) pointed out, this state may be much more widespread but widely overlooked.

State 2 is found in *Diadectes* (which has three cusps in a labiolingual row: Berman et al., 2004) and *Orobates* (Berman et al., 2004).

Lethiscus has state 0 (Pardo et al., 2017).

Unknown in *Pederpes* (Clack & Finney, 2005: 322).

**Saharastega* has state 0 (D. M., pers. obs. of MNN MOR 73).

**Carrolla* has two cusps per teeth; according to Maddin, Olori & Anderson (2011), it is not clear if they were arranged labiolingually or mesiodistally in life. As for character TEETH 10, we have therefore assigned state 0 or 2 to **Carrolla*.

Because crown-group salamanders replace mono- by bicuspid teeth during metamorphosis, we have scored the neotenic **Beiyanerpeton*, which has monocuspid teeth, as unknown.

184. TEETH 4: **Conspicuous peak involving one or more anterior maxillary teeth: absent (0); present (1).**

Acanthostega has state 1: a clear caniniform region is preserved in the holotype of the only species, *A. gunnari* (D. M., pers. obs. of TMM 41766-1, a cast of MGUH VP 6033). This may also have been noted by Clack & Milner (2015: 18), who noted that the maxillary teeth are “largest at positions 7 to 11” (even though that contradicts their fig. 3A and 6A), and possibly by Porro, Rayfield & Clack (2015: 10). Given the (already scored) presence of state 1 in *Ventastega*, *Ichthyostega* and *Whatcheeria*, this is less surprising than it might look at first glance.

Chenoprosopus shares state 1 (Hook, 1993), and so do *Cochleosaurus* (Sequeira, 2004), *Acheloma* (Dilkes & Reisz, 1987: fig. 3), and *Phonerpeton* (Dilkes, 1990: fig. 1) as well as *Discosauriscus* (borderline: Klembara, 1997), *Ariekanerpeton* and *Utegenia* (Bulanov, 2003; figures in Klembara & Ruta, 2004a, 2005a). *Brukterpeton* (Boy & Bandel, 1973) and *Hapsidopareion* (CG87: fig. 14D) have a weak version of state 1.

Cardiocephalus has state 0 (CG78), as do *Lethiscus* (Anderson, Carroll & Rowe, 2003; Pardo et al., 2017) and *Vieraella* (Báez & Basso, 1996).

Euryodus is polymorphic; whether the caniniform tooth of one of the two species counts as "anterior" is debatable, but the number of maxillary teeth rostral to it is low.

Scincosaurus is unknown for this character (Milner & Ruta, 2009).

We have assigned state 0 to **Nigerpeton* because its huge maxillary tusks are not part of the marginal toothrow in MNN MOR 70 (D. M., pers. obs.). This amounts, however, to an ontogenetic hypothesis: the maxillary tusks are part of the toothrow in the smaller MNN MOR 69 and not preserved in the intermediate-sized MNN MOR 108 (D. M., pers. obs.).

Judging from the reconstruction by Klembara (2011: fig. 3B), **Karpinskiosaurus* had a very prominent caniniform region (state 1) in the adult stage; unfortunately, neither Klembara (2011) nor Bulanov (2003) mentioned this character at all, and no photographs or specimen drawings that would show it appear to have been published – the published illustrations (Bulanov, 2003; Klembara, 2011) all show younger individuals which had state 0. We have provisionally scored state 1.

States 0 and 1 appear to be found in different individuals of **Lydekkerina* (Jeannot, Damiani & Rubidge, 2006; Hewison, 2007).

Unclear in the *St. Louis tetrapod; it is even possible that there was a caniniform region on the left but not the right side of the only known specimen (Clack et al., 2012b: fig. 2A; D. M., pers. obs. of MB.Am.1441.2). We have scored it as unknown.

185. TEETH 5: **Dentary teeth larger than maxillary teeth: no (0); yes (1).**

We have scored *Crassigyrinus* as having state 1 because almost all dentary teeth are larger than all maxillary ones (Clack, 1998). A very similar condition, which we have also scored as state 1, is found in Dendrerpetidae (Godfrey, Fiorillo & Carroll, 1987) and *Caerorhachis* (Ruta, Milner & Coates, 2002).

In *Neldasaurus*, only ⅓ to ½ of its dentary teeth are larger than the maxillary teeth (Chase, 1965: fig. 2, 6), but we count this as state 1 because the larger teeth do not form a specialized caniniform (or other) region.

Trimerorhachis has state 1 (Case, 1935: fig. 13, pl. VII; Milner & Schoch, 2013), as does *Platyrhinops* (Clack & Milner, 2010).

"The individual teeth are definitely longer and have a greater diameter than those of the upper jaw" (CG78); we have accordingly scored state 1 for *Micraroter*.

State 0 is shown for *Bruktererpeton* in table 7 of Boy & Bandel (1973). It is also present in *Solenodonsaurus* (where the maxillary teeth are indeed larger than the dentary ones: Danto, Witzmann & Müller, 2012), *Scincosaurus* (from comparing fig. 2A and fig. 4 of Milner & Ruta, 2009), *Diceratosaurus* (D. M., pers. obs. of CM 67169), *Lethiscus* (Pardo et al., 2017), **Nigerpeton* (D. M., pers. obs. of MNN MOR 70) and **Saharastega* (D. M., pers. obs. of MNN MOR 73).

Following Bystrow (1944) and DEN 7 (see above), we have scored state 0 for *Kotlassia*.

Orobates was scored as polymorphic in RC07, presumably due to its long incisiform dentary teeth that are longer than the maxillary teeth (and oppose similarly long incisiform teeth on the premaxilla). We exclude incisiform teeth (a very rare specialization in this matrix) from consideration and therefore score *Orobates* as possessing only state 0.

Sequeira & Milner (1993) reconstructed *Capetus* with a mild version of state 1 (in more than half of the toothrow).

**Crinodon* is apparently borderline; we have scored it as unknown.

The dentary teeth of **Palatinerpeton* (Boy, 1996) vary in size, being smaller opposite the caniniform region of the maxilla and premaxilla but larger elsewhere. Many are larger

than most of its right maxillary teeth, but this may not hold for the left side; we have scored *Palatinerpeton* as unknown.

The few exposed dentary teeth of **Erpetosaurus* are no larger than the tiny maxillary teeth (Milner & Sequeira, 2011); however, the dentary teeth of the same region of *Isodectes* are no larger than the maxillary teeth either, while the remaining ones are easily twice as large (Sequeira, 1998), so we have scored **Erpetosaurus* as unknown.

State 1 is strongly suggested for **Pholidogaster* by Romer (1964: fig. 3, alone and in comparison with pl. 1); we have scored **Pholidogaster* accordingly, even though Romer (1964) nowhere mentioned this character in the text.

Unless the distalmost teeth in the right maxilla are all much less far erupted than the dentary teeth opposite them, the *St. Louis tetrapod has state 1; the discrepancy is larger than shown by Clack et al. (2012b: fig. 2A, B), because those are ventral and ventrolabial views rather than strictly labial ones (D. M., pers. obs. of MB.Am.1441.2). However, the discrepancy is still noticeably smaller than in *Greererpeton* (let alone *Colosteus*; Smithson, 1982; Hook, 1983); in addition, the dentary teeth are distinctly smaller than the ectopterygoid fang next to them, quite unlike in *Greererpeton* and *Colosteus*. In order to emphasize this difference, we have accepted the claim of "no clear discrepancy in tooth size between upper and lower jaw rami" (Clack et al., 2012b: 22) and scored state 0.

186. TEETH 6: **Marginal tooth crowns chisel-tipped: no (0); yes (1).**

Lethiscus has state 0 (Pardo et al., 2017), as does **Saharastega* (D. M., pers. obs. of MNN MOR 73).

Oestocephalus was scored in RC07 as having state 0 or 1; it has 0 and 1, respectively, in different specimens (species?) from different sites (Carroll, 1998a), so we have scored polymorphism.

187. TEETH 7: **Marginal tooth crowns without (0) or with (1) 'dimple'.**

**Saharastega* has state 0 (D. M., pers. obs. of MNN MOR 73).

188. TEETH 8: **Marginal tooth crowns robust and conical: absent (0); present (1).** Such teeth require so much space that not many of them fit into a maxilla (apparently never more than 12 or perhaps 15), so that state 1 makes TEETH 9 inapplicable. Simply adding this state to TEETH 9, however, is not an option: in *Batropetes*, each maxilla bears 4 to 6 mostly cylindrical teeth (mostly or entirely individual variation) and is small enough to only have space for 10, yet the teeth are thin and cylindrical except for an expanded, tricuspid tip (Glienke, 2013, 2015); in *Brachydectes* and **Carrolla*, each maxilla holds only 5–8 relatively enormous teeth that are pointed but recurved and much taller (thus thinner) than in taxa with state 1 (Maddin, Olori & Anderson, 2011; Pardo & Anderson, 2016); in *Scincosaurus*, there are 8 slender, stalked teeth per maxilla (Bossy & Milner, 1998; Milner & Ruta, 2009); *Keraterpeton*, *Batrachiderpeton* and *Diceratosaurus* have similarly few teeth of entirely plesiomorphic shape and size (Bossy & Milner, 1998; D. M., pers. obs. of *Diceratosaurus* specimens), while those of *Diploceraspis* are large and have an intermediate shape (Beerbower, 1963) – when teeth are very few in number, they are not necessarily robust and conical.

Carroll (1969a) reconstructed *Acherontiscus* as having 15 maxillary teeth; the preserved teeth – the last 4 – are robust and bluntly conical. We count this as state 1.

189. TEETH 9: **Number of maxillary teeth greater than 40 (0), between 30 and 40 (1), between 13 and 29 (2); smaller than 13 (3) (ordered).** As this is a meristic (practically continuous) character, we have ordered it. Indeed, unambiguous changes between states 0 and

2 are seen only three times in the shortest trees from Analysis R4, and state 3 only changes to (once) and from state 2 (twice), while the character as a whole has 48 steps.

Even apart from this, the distribution of the states is nowhere near random. State 1 or higher is an autapomorphy (reversed a minimum of nine times) of the smallest clade that contains **Ymeria* and the crown-group; state 2 is rare in temnospondyls, and state 3 is limited to two clades of amphibians, while almost all members of the seymouriamorph-diadectomorph-amniote-amphibian clade have state 2 or 3 (where applicable – see above). Clearly, the claim that this character contains "little phylogenetic signal" (RC07) is not tenable.

State 3 is new, and found in *Batropetes*, *Brachydectes*, *Scincosaurus*, *Keraterpeton*, *Batrachiderpeton*, *Diceratosaurus*, *Diploceraspis* and **Carrolla* (see TEETH 8); state 2 of RC07 was simply "smaller than 30". The cutoff is chosen so that *Albanerpeton inexpectatum* retains state 2; it has 15 to 23 teeth per maxilla (Estes & Hoffstetter, 1976: 312), while no OTU in this matrix seems to have 13 or 14.

Colosteus has at least 34 maxillary teeth (Hook, 1983: fig. 1), giving it state 0 or 1.

Isodectes seems to have state 1 or 2 (Sequeira, 1998: 252 and fig. 9B, C).

Trimerorhachis has state 0 (Milner & Schoch, 2013).

Acheloma seems to have had 30 maxillary teeth at the most (Polley & Reisz, 2011), giving it state 1 or 2.

Micromelerpeton was scored as unknown in RC07. Boy (1972: 25) reported state 1, agreeing with the reconstruction (fig. 5) and possibly with a specimen drawing (fig. 4p) in the same paper, so we have scored state 1.

Apateon, too, was scored as unknown. Schoch & Milner (2008: fig. 4E) reconstructed *A. pedestris* with 25 teeth on one maxilla and 26 on the other; this resolves to state 2. Boy (1987: 84; translated by D. M.), however, ascribed "maximally 23–35 teeth" to *A. pedestris*, spanning states 1 and 2. State 2 was also reported for *A. dracyi* (as "*A. dracyiformis*") by Boy (1986: 158, 1987: 90), although this may be due to skeletal immaturity (the maxilla is very short); state 1 was confirmed for *A. caducus* by Boy (1987: 88 – "Long, slender maxilla […] bears more than 30 teeth"; translated by D. M.). Metamorphosed *A. gracilis*, however, has state 0 with about 50 maxillary teeth (Werneburg, 1991: 82); indeed, the maxilla grows with positive allometry (Werneburg, 1991: 82). We have scored state 0, because *A. gracilis* is the only species of *A.* known to undergo metamorphosis.

Leptorophus tener has state 0 or 1: "maxilla with maximally 37–42 teeth" (Boy, 1986: 139; translated by D. M.). *L. raischi* has "space for about 30 teeth" in the maxilla (Schoch, 2014a: 231), which would translate to state 1 or 2. Because the shorter snout of *L. raischi* compared to *L. tener* is a sign of relative skeletal immaturity and because polymorphism with partial uncertainty is impossible in PAUP*, we have ignored the possibility of state 2 and have scored *Leptorophus* as having states 0 and 1; Schoch (2014a) pointed out, however, that the teeth of *L. raischi* are larger than those of *L. tener*, so if the former continued to grow to the latter's snout length, it would still have fewer teeth.

Bruktererpeton has "ca. 40 teeth" in the maxilla, giving it state 0 or 1 (Boy & Bandel, 1973: 50, translation by D. M.).

In *Kotlassia*, "the number of marginal teeth remains uncertain" due to incomplete preservation and incompetent preparation (Bulanov, 2003); *Kotlassia* was accordingly scored as unknown. However, a few maxillary teeth remain in the holotype (Bulanov, 2003); we therefore trust Bystrow's (1944: fig. 2) reconstruction of state 1 even though the exact number of maxillary teeth is uncertain.

State 2 is found in *Cochleosaurus* (Sequeira, 2004), *Valdotriton* (Evans & Milner, 1996), apparently *Lethiscus* (Pardo et al., 2017) and *Leptoropha* (Bulanov, 2003).

**Micropholis* is polymorphic, showing states 0 and 1 (Schoch & Rubidge, 2005).

We estimate 20 to 25 maxillary teeth, thus state 2, for the *Goreville microsaur (Lombard & Bolt, 1999: fig. 2).

We have scored **Sigournea* as possessing state 0. The maxilla is unknown, but the dentary has so many tooth positions (88) that any other state would require the maxillary teeth to be implausibly large and/or widely spaced (Bolt & Lombard, 2006). The same holds for the 98 to 100 tooth positions in the dentary of **Doragnathus* (Smithson, 1980) and the about 90 to 100 known tooth positions in the dentary of **Elginerpeton* (Ahlberg, 1995; Ahlberg & Clack, 1998; Ahlberg, Friedman & Blom, 2005).

Because of its paedomorphically short maxilla, we have scored **Beiyanerpeton* as having state 0 or the observed uncertainty of state 1 or 2.

States 1 and 2 are known in adult **Glanochthon* (Schoch & Witzmann, 2009b: figs. 3, 4).

Panchen (1964: fig. 13) reconstructed space for 32 teeth in **Palaeoherpeton*; we have cautiously scored state 1 or 2.

190. TEETH 10: **Maximum number of mesiodistally arranged cusps per marginal tooth at any point in ontogeny: 1 (0), lozenge-shaped crown with mesial and distal ridges that lead to the mesial and distal corners or very short cusps (1), 3 (2), more (3) (ordered).** This character is ordered for the same reasons as TEETH 3, from which it is split.

State 0 is the plesiomorphy, found in all OTUs that preserve tooth crowns (including **Saharastega*: D. M., pers. obs. of MNN MOR 73) except for the following:

State 1 is found in *Discosauriscus* (at least in the tooth in the third alveolus in fig. 10 of Klembara 1997) and *Scincosaurus* (Milner & Ruta, 2009).

State 2 occurs in *Batropetes* (Carroll, 1991; Glienke, 2013, 2015) and **Tungussogyrinus* (Werneburg, 2009).

State 3 is limited to *Leptoropha* and *Microphon* (Bulanov, 2003) in the present matrix.

Albanerpetidae is polymorphic, with *Anoualerpeton* possessing state 1 (Gardner, Evans & Sigogneau-Russell, 2003) and its sister-group, composed of *Celtedens* and *Albanerpeton*, showing state 2 (e.g. Estes & Hoffstetter, 1976; Fox & Naylor, 1982; McGowan, 2002).

Westlothiana shows partial uncertainty between states 0 and 1 (Smithson et al., 1994).

**Carrolla* has two cusps per tooth; according to Maddin, Olori & Anderson (2011), it is not clear if they were arranged labiolingually or mesiodistally in life. As for character TEETH 3, we have therefore assigned state 0 or 2 to **Carrolla* (3 cusps are the closest condition to 2).

191. CLE 1: **T-shaped dorsal expansion of cleithrum: absent (0); present (1).**

State 0 is known in *Ventastega* (Ahlberg et al., 2008), *Baphetes* (Milner & Lindsay, 1998), *Cochleosaurus* (Sequeira, 2009), *Isodectes* (D. M., pers. obs. of CM 81512 and CM 81430) and *Doleserpeton* (Sigurdsen & Bolt, 2010).

CG78 (p. 60, fig. 31) identified an L- to C-shaped bone as the cleithrum in *Cardiocephalus*. We do not understand why and have kept the scores of all three CLE characters as unknown. It might be a clavicle (if so, we still cannot score the only CLA character) or an unrelated part of the fissure-fill taphocoenosis at Fort Sill.

192. CLE 2: **Cleithrum with (0) or without (1) postbranchial lamina.** Probably unlike RC07, we count everything that is primarily homologous to a postbranchial lamina as state 0; this includes laminae that may have the wrong size or shape to function as a postbranchial lamina, so we do not hypothesize on which of the taxa we have scored 0 actually possessed internal gills in life. What sizes and shapes are possible for a functional postbranchial lamina

has not, to the best of our limited knowledge, ever been investigated; conversely, the lamina is clearly not necessary for internal gills to function – even the lamina "of *Eusthenopteron* is very narrow and poorly differentiated from the cleithral blade" (Ahlberg, 1998: 113). Moreover, cleithra of limbed vertebrates are almost never illustrated or described in cranial or caudal view (the postbranchial lamina is impossible to see in lateral view, and difficult or impossible to recognize in medial view at least in drawings); sometimes, like in the description of the postcranium of *Greererpeton* by Godfrey (1989), the lamina has been illustrated but not recognized (as previously noted by Lebedev & Coates, 1995, and Coates, 1996). We suspect therefore that state 0 is more widespread than we have been able to score.

Ch. 43 of Coates (1996) was called "Post-branchial lamina: present (0); absent (1)" in the character list (p. 417), but in the text Coates (1996: 400) specified "a broad postbranchial lamina (ch. 43)" (without attempting to define "broad"). This supports our interpretation that RC07 scored laminae that do not fulfill unspecified criteria of size and/or shape as state 1.

Under our possibly expanded definition, state 0 is present in *Ventastega* (Ahlberg et al., 2008: fig. 2b, 3e – contradicting the text which evidently uses a stricter definition), *Baphetes* (Milner & Lindsay, 1998; Milner, Milner & Walsh, 2009), and *Archeria* (Pawley, 2006: fig. 70-2.4); compare the conditions of *Ichthyostega* (Jarvik, 1996: pl. 45) and *Greererpeton* (Godfrey, 1989: fig. 17b), which were already scored 0.

Ossinodus has a candidate lamina. Warren & Turner (2004) identified that lamina as the sutural surface for the clavicle; it is clear from Bishop (2014), however, that this can only be true for the ventralmost part, leaving the rest to function as a postbranchial lamina and us to score it as state 0. The similarity between the cleithra of *Ossinodus* (Warren & Turner, 2004: fig. 9B, C; Bishop, 2014: fig. 8B, E) and *Archeria* (Pawley, 2006: fig. 70-2.4) is striking; contrast *Eryops* (Pawley & Warren, 2006: 4), which has state 1 as already scored.

Crassigyrinus was scored as unknown in RC07. Laurin (2011: 57) wrote that it had a postbranchial lamina, citing Coates (1996) as his source, yet Coates (1996) did not mention *Crassigyrinus* in his discussion of the postbranchial lamina and scored it in his data matrix as lacking the lamina. Given that our definition of the postbranchial lamina is less strict than that of Coates (1996), we have kept the score as unknown.

State 1 is preserved in *Cochleosaurus* (Sequeira, 2009: fig. 6, 7) and *Doleserpeton* (Sigurdsen & Green, 2011: appendix 2). In *Gephyrostegus*, state 1 seems to be preserved in TMM 41773-1, so we have scored state 1 as present, though crushing and the fact that this specimen is a cast leave doubts about this, in particular about the interpretation of the cleithrum as being preserved in medial view.

Clack & Finney (2005) stated that the postbranchial lamina is absent in *Pederpes*, and illustrated this with a cross-section of the cleithrum (fig. 11 B). It is not at all obvious from the figure (including the photo: fig. 11 A) or the text if the very tall ridge is medial (thus potentially a postbranchial lamina) or lateral; however, personal observation by D. M. of the only known specimen (GLAHMS 100815) on exhibit in the Hunterian Museum, Glasgow, shows that it is lateral, meaning that there is no postbranchial lamina, so the score of 1 in RC07 is correct despite the occurrence of state 0 in *Whatcheeria* and *Ossinodus*.

Unknown (or at least not sufficiently well illustrated and described) in *Proterogyrinus* (Holmes, 1984), *Pholiderpeton scutigerum* (Clack, 1987b), *Brukterpeton* (Boy & Bandel, 1973), *Kotlassia* (Bystrow, 1944) and *Adelogyrinus* (Andrews & Carroll, 1991: 252).

Perhaps surprisingly, **Casineria* has state 0 (Fig. 7), which we have therefore scored. The condition appears to be quite similar to that seen in *Archeria* (Pawley, 2006: fig. 70-2.4); see Discussion: Phylogenetic relationships: The interrelationships of Anthracosauria, Silvanerpeton, Caerorhachis, Gephyrostegidae, Casineria and Temnospondyli.

193. CLE 3: **Cleithrum co-ossified with scapulocoracoid: yes (0); no (1).**

State 0 is present in *Ventastega* (Ahlberg et al., 2008).

Whatcheeria is polymorphic (Coates, 1996: 409). Surprisingly, so is *Eryops* (Pawley & Warren, 2006).

Baphetes has state 1 (Milner & Lindsay, 1998; Milner, Milner & Walsh, 2009), as do *Cochleosaurus* (Sequeira, 2009) and *Isodectes* (D. M., pers. obs. of CM 81430).

Inapplicable to salamanders, which lack cleithra entirely, and to aïstopods, which lack scapulocoracoids entirely.

"In late adult individuals, the two bones are probably fused to each other" in **Sclerocephalus* (Meckert, 1993: 123; translation by D. M.); we have stayed conservative and scored **Sclerocephalus* as possessing state 1.

194. CLA 3: **Clavicles meet anteriorly: yes (0); no (1).**

State 0 occurs in *Diadectes* (Case, 1911: 79, fig. 26) and *Orobates* (Berman et al., 2004: fig. 2B). It is most likely shared by *Chenoprosopus*, judging from the disarticulated clavicle of USNM 437646 (Hook, 1993: fig. 2; D. M., pers. obs.).

State 1 is present in *Ventastega* (Ahlberg, Lukševičs & Lebedev, 1994; Ahlberg et al., 2008). *Baphetes* shares state 1 (Milner & Lindsay, 1998), as do *Cochleosaurus* (Sequeira, 2009), *Isodectes* (D. M., pers. obs. of USNM 4471, CM 81512 and CM 81430), *Leptorophus* (Werneburg, 2007a), *Ossinodus* (Bishop, 2014) and, judging from the shapes of clavicle and interclavicle, *Doleserpeton* (Sigurdsen & Bolt, 2010) and **Sparodus* (Carroll, 1988). Clack & Milner (2010) implied it for *Platyrhinops*.

Unclear in *Kotlassia* (Bystrow, 1944: fig. 15); best scored as unknown in **Chroniosaurus* and **Bystrowiella* (Witzmann & Schoch, 2017).

Apparently unknown in *Notobatrachus* (Báez & Nicoli, 2004); **Liaobatrachus* has state 0 (Dong et al., 2013).

**Lydekkerina* is polymorphic (Shishkin, Rubidge & Kitching, 1996; Pawley & Warren, 2005; Hewison, 2007: 42).

195. INTCLA 1-2: **Interclavicle posterior margin not drawn out into parasternal process (0), with parasternal process that is not parallel-sided (1), or with elongate, slender process that is parallel-sided for most of its length (2) (unordered).** Although called INTCLA 1 by RC07, this character is a composite of INTCLA 1 and INTCLA 2 of Ruta, Coates & Quicke (2003), which were still kept separate by Pawley (2006: 350). For the time being, we have not ordered it because we have yet to compare the data on its changes in ontogeny and phylogeny.

State 0 is found in *Cochleosaurus* (Sequeira, 2009) and *Doleserpeton* (Sigurdsen & Bolt, 2010).

Ventastega (Ahlberg et al., 2008) and *Batropetes* (Glienke, 2013, 2015) have state 1.

Limnoscelis (Kennedy, 2010) and *Tseajaia* (Moss, 1972) show state 2; the process has a unique club-shaped expansion at the caudal end, but is otherwise parallel-sided.

Baphetes (Milner, Milner & Walsh, 2009: fig. 3A, 4B) and *Hyloplesion* (Olori, 2015: 57) have state 1 or 2.

196. INTCLA 3: **Interclavicle wider than long (excluding parasternal process, if present): absent (0); present (1).**

Ventastega (Ahlberg et al., 2008), *Cochleosaurus* (Sequeira, 2009) and *Bruktererpeton* (Boy & Bandel, 1973: fig. 10) have state 0.

As reconstructed by Pawley & Warren (2006) and mentioned in the text of their publication, *Eryops* shows state 1. The fimbriate rostral margin indicates that growth may have continued till state 0 was reached, and indeed state 0 is not far away, but there is no

evidence that this happened, the known individuals all seem well advanced in age, and the fimbriation is much weaker than in *Microbrachis* or *Hyloplesion*. We have therefore scored *Eryops* as indeed possessing state 1.

State 1 is further present in *Limnoscelis* (Kennedy, 2010), *Hyloplesion* (CG78: fig. 87B; Olori, 2015) and *Orobates* (Nyakatura et al., 2015: digital reconstruction).

We have scored *Schoenfelderpeton* as unknown due to its pronounced paedomorphosis.

State 1 was scored for *Ossinodus* in RC07, but as preserved (Warren & Turner, 2004) the incomplete interclavicle shows state 0; because of its broken margins we have scored it as unknown.

197. INTCLA 4: **Caudal part of interclavicle (excluding parasternal process, if present) longer (0) or shorter (1) than cranial part.** The original wording was: "Interclavicle rhomboidal with posterior part longer (0) or shorter (1) than anterior part", but it is usually possible to distinguish the parts even when the interclavicle lacks the plesiomorphic deltoid shape, the boundary being the mediolateral line along which the interclavicle is widest. We have added the mention of the parasternal process.

Ventastega (Ahlberg et al., 2008) and *Limnoscelis* (Kennedy, 2010) have state 0, as do *Tuditanus* and *Pantylus* (CG78) and *Orobates* (Nyakatura et al., 2015: digital reconstruction).

Cochleosaurus shows a mild case of state 1 (Sequeira, 2009), as, surprisingly, *Doleserpeton* (Sigurdsen & Bolt, 2010) seems to. *Eryops* is an unambiguous example of state 1, with the caudal part only about 2/3 as long as the cranial part (Pawley & Warren, 2006). State 1 is further found in *Archeria* (Romer, 1957: fig. 1C), *Kotlassia* (Bystrow, 1944: fig. 15) and *Seymouria* (White, 1939: fig. 21).

**Sclerocephalus* passes from state 0 to state 1 in ontogeny (Meckert, 1993: fig. 1, 4; Schoch & Witzmann, 2009a: fig. 6A, D, E).

198. INTCLA 5: **Transversely elongate grooves and ridges on central part of interclavicle ventral surface: absent (0); present (1).**

Ventastega (Ahlberg et al., 2008), *Chenoprosopus* (Hook, 1993; D. M., pers. obs. of USNM 437646), *Cochleosaurus* (Sequeira, 2009), *Trimerorhachis* (Pawley, 2007) and *Doleserpeton* (Sigurdsen & Bolt, 2010) have state 0, as do *Solenodonsaurus* (Danto, Witzmann & Müller, 2012) and *Limnoscelis* (Kennedy, 2010).

Whatcheeria was scored 0 in RC07. We have changed it to unknown, because ornamentation is entirely absent not only on the skull but also on the shoulder girdle of *Whatcheeria* (Lombard & Bolt, 1995: 483–484), making state 0 predictable and thus this character inapplicable.

State 1 is, surprisingly, found in *Eryops* (Pawley & Warren, 2006).

Panchen (1975) described an interclavicle as belonging to **Pholidogaster* even though it was isolated and not found at the type locality. Clack & Milner (2015: 49) pointed this out and added that this interclavicle "is unlike undisputed colosteid interclavicles in shape", casting doubt on its referral. This refers to the fact that it has state INTCLA 4(0) (see above), the plesiomorphy, while the two other colosteids that preserve interclavicles – *Colosteus* and *Greererpeton* – have state 1. However, the interclavicle of the holotype, which is partially covered by the clavicles and partially preserved as an impression, does have state 0 of that character (Romer, 1964). This is not surprising for a colosteid that lies outside the smallest clade formed by *Colosteus* and *Greererpeton*. For the moment, we accept Panchen's (1975) referral and have used the interclavicle he described to score **Pholidogaster* where that of the holotype does not suffice; this concerns only the present character (state 0).

199. SCACOR 1: **Separate scapular ossification: absent (0); present (1).** As previously pointed out (e.g. Marjanović & Laurin, 2008: 185), ontogenetic fusion makes this character difficult to interpret. Also, the taxon sample is perhaps somewhat unfortunate – the salamanders in this matrix (*Karaurus*, *Valdotriton*, **Beiyanerpeton*, **Pangerpeton* and **Chelotriton*) all have state 0, but ***Kokartus*, a close relative of *Karaurus*, shows state 1 in an apparently immature specimen (Averianov et al., 2008: 480, fig. 7B), as do adults of the extant paedomorphic salamanders ***Amphiuma* and ***Siren* (Goodrich, 1930).

Nonetheless, we have scored *Ventastega* (Ahlberg et al., 2008), *Platyrhinops* (implied by Carroll, 1964, and Clack & Milner, 2010) and *Micromelerpeton* (implied by Boy, 1995: 444) as possessing state 0.

In contrast, we have not been able to find any mention of the endochondral shoulder girdle of *Amphibamus* in the literature (Carroll's [1964] "*Amphibamus lyelli*" is *Platyrhinops*) and have therefore scored it as unknown.

Where known (*Celtedens*: McGowan, 2002), albanerpetids have state 1. So do *Eocaecilia* (Jenkins, Walsh & Carroll, 2007), *Diplocaulus* (Douthitt, 1917) and *Orobates* (Nyakatura et al., 2015: digital model).

In *Bruktererpeton* the condition is unknown because the coracoid region is entirely unossified (Boy & Bandel, 1973).

Captorhinus was scored 0 in RC07. Fox & Bowman (1966) insisted several times that it has state 1, but provided no evidence other than a notch which supposedly marked the otherwise obliterated suture between the procoracoid and the metacoracoid. It is quite likely that *Captorhinus* changed from state 1 to state 0 in ontogeny, but in the absence of a reference for state 1, we have kept state 0.

Unknown in *Trimerorhachis*, where of the entire scapulocoracoid only the central part of the scapula ever ossifies (Pawley, 2007), and in *Brachydectes* (Wellstead, 1991), where the same seems to be the case. Also unclear in *Microbrachis*, where a break in an unusually well ossified specimen may or may not correspond to a suture (Olori, 2015).

Danto, Witzmann & Müller (2012) identified a small bone of *Solenodonsaurus* that, as far as preserved, does not participate in the glenoid as a coracoid would; despite this uncertainty, they scored *Solenodonsaurus* as having state 1 of this character. We side with the uncertainty of the text and fig. 4 and have scored *Solenodonsaurus* as unknown.

Based on the report of a longitudinal groove which may be "a partially co-ossified suture" (Schoch & Rubidge, 2005: 511), we have assigned state 1 to **Micropholis*.

200. SCACOR 2: **Glenoid subterminal: yes (0); no (1).** This character describes whether the coracoid part of the scapulocoracoid is at least partly ventral (state 1) or purely medial to the glenoid (state 0); it is not applicable to taxa with unossified coracoids, unless the clavicles and the interclavicle allow a 3D reconstruction.

State 0 occurs in *Ventastega* (Ahlberg et al., 2008), *Cochleosaurus* (Sequeira, 2009), *Doleserpeton* (Sigurdsen & Bolt, 2010), *Eoscopus* (Daly, 1994: 16) and *Diplocaulus* (Williston, 1909), likely also in *Apateon* (Werneburg, 1991: fig. 5b).

State 1 is found in *Eryops* (Pawley & Warren, 2006; D. M., pers. obs. of TMM 31227-14), Albanerpetidae (McGowan, 2002), *Eocaecilia* (Jenkins, Walsh & Carroll, 2007), *Scincosaurus* (Milner & Ruta, 2009), *Orobates* (Nyakatura et al., 2015: digital model) and *Ossinodus* (Warren & Turner, 2004: fig. 9I; Bishop, 2014: fig. 12).

Glienke (2013: fig. 5K) reconstructed state 1 for *Batropetes niederkirchensis*; Glienke (2015: fig. 5B) reconstructed state 0 for *B. palatinus*; Glienke (2015: fig. 8G) reconstructed state 1 for *B. appelensis*. In *B. niederkirchensis* and *B. palatinus*, the coracoid portion is preserved and shows state 1, which may be due to diagenetic pressure; in *B. appelensis*, the coracoid portion was unossified, but the clavicles and the interclavicle allow a 3D

reconstruction. It is not clear from Glienke (2015: fig. 5) or from MB.Am.1232 (D. M., pers. obs.) whether this is also the case for *B. palatinus*; for the time being, we have scored *Batropetes* as polymorphic.

201. SCACOR 3: **Enlarged glenoid foramen: absent (0); present (1).** "Loss of an enlarged glenoid foramen occurs sporadically and does not show any clear phylogenetic signal" (RC07: 105, 106) – it only happens at most once on the shortest trees from Analysis R6 (in *Pantylus*; unknown in *Stegotretus*) and at most twice in R4 and R5 (*Pantylus* and *Batropetes* + Lissamphibia; unknown in *Brachydectes* and many other "microsaurs"), for a constant total of four steps under the LH, the TH and the PH. Presence of the enlarged foramen, however, may keep several "microsaurs" together with each other (*Asaphestera*, *Pelodosotis*, **Trihecaton*; unknown in most others) and with *Diplocaulus*.

Doleserpeton (Sigurdsen & Bolt, 2010, Sigurdsen & Green, 2011: appendix 2), apparently *Apateon* (Werneburg, 1991: fig. 5b), *Eocaecilia* (Jenkins, Walsh & Carroll, 2007), apparently *Triadobatrachus* (Ascarrunz et al., 2016: 3D model 1), *Valdotriton* (Evans & Milner, 1996: fig. 11a), *Batropetes* (Carroll, 1991; Glienke, 2013, 2015), *Notobatrachus* (Báez & Nicoli, 2004: fig. 3B) and *Orobates* (Nyakatura et al., 2015: digital model) have state 0; the notch cranial of the glenoid of *Notobatrachus* should correspond to the coracoid foramen, not the glenoid foramen, judging from Pawley & Warren (2006: fig. 5).

State 1 is present in *Ventastega*, assuming this is what "glenoid canal" means (Ahlberg et al., 2008). The condition in *Diplocaulus* (Williston, 1909: pl. 4a) may also count.

Unknown in *Trimerorhachis* (Pawley, 2007), *Scincosaurus* (Milner & Ruta, 2009) and *Ossinodus* (Warren & Turner, 2004: 166).

202. SCACOR 4: **Ventrome[d]ially extended infraglenoid buttress: absent (0); present (1).**

State 0 is present in *Ventastega* (Ahlberg et al., 2008).

Doleserpeton has state 1 (Sigurdsen & Bolt, 2010, Sigurdsen & Green, 2011: appendix 2). State 1 is also known in *Eocaecilia* (most likely: Jenkins, Walsh & Carroll, 2007: fig. 40), *Triadobatrachus* (Ascarrunz et al., 2016: 3D model 1), *Westlothiana* (Smithson et al., 1994: 392), *Diplocaulus* (Williston, 1909: pl. 4a) and *Orobates* (Nyakatura et al., 2015: digital model).

203. ANOCLE 1: **Anocleithrum: present (0); absent (1).**

State 0 is present in *Ventastega* (Ahlberg et al., 2008).

State 1 looks like a safe interpretation of *Cochleosaurus* (Sequeira, 2009: fig. 2, 4, 6).

Complete articulated skeletons of *Celtedens*, with even the scales in place, demonstrate state 1 for Albanerpetidae (McGowan, 2002).

The dorsal end of the cleithrum of *Tseajaia* is known and lacks a contact surface for an anocleithrum (Moss, 1972), so we have taken the absence of a preserved anocleithrum at face value and scored *Tseajaia* as possessing state 1.

Only a small part of the cleithrum has ever been found in any of the many specimens of *Trimerorhachis* (Pawley, 2007); we therefore cannot (except by phylogenetic bracketing) feel safe about taking the lack of an anocleithrum at face value and have changed the score to unknown.

204. HUM 1: **Latissimus dorsi process offset anteriorly relative to the ectepicondyle (0) or aligned with the latter (1).**

This character and HUM 4 are inapplicable to *Ichthyostega*, where the attachment surface for the latissimus dorsi isn't much of a process. Ahlberg (2011) stated that the M.

latissimus dorsi probably attached to process 1; however, even if so, process 1 can hardly be considered homologous with the latissimus dorsi process or ridge in other OTUs in this matrix (Callier, Clack & Ahlberg, 2009: supp. inf.; Ahlberg, 2011: fig. 1; Clack et al., 2012a). It is confusing, though, that Ahlberg is a coauthor of Callier, Clack & Ahlberg (2009) as well as of Clack et al. (2012a), and that his 2011 paper was both submitted and accepted while the manuscript of Clack et al. (2012a) was in review.

State 0 is now known in *Ossinodus* (Bishop, 2014). It also appears to be present in *Panderichthys* (Boisvert, Mark-Kurik & Ahlberg, 2008: supplementary movie 4); a possible process for the M. latissimus dorsi can be seen in a location similar to where the process is in *Acanthostega* (Coates, 1996: fig. 16d), distal to a foramen – the feature labeled "ldp" by Coates (1996: fig. 35i) is instead a part of the ectepicondyle ridge. Instead of a process, Ahlberg (2011) figures several ridges as the attachment area for the M. latissimus dorsi; these are offset anteriorly (preaxially) from the ectepicondyle as well.

State 1 is documented in all but the smallest specimens of *Trimerorhachis* (Pawley, 2007) and appears to occur in *Isodectes* (D. M., pers. obs. of USNM 4474) and in *Karaurus* (D. M., pers. obs. of unnumbered MNHN cast of PIN 2585/2).

205. HUM 2: **Distinct "supinator" (brachioradialis) process projecting anteriorly: absent (0); present (1).** See Bishop (2014) for the homology of this feature.

State 0 occurs in *Baphetes* (Milner & Lindsay, 1998), *Doleserpeton* (Sigurdsen & Bolt, 2009; D. M., pers. obs. of AMNH 29466) and *Eocaecilia* (Jenkins, Walsh & Carroll, 2007).

Lebedev & Coates (1995: 316) described a not particularly pointed, but large "supinator" process in *Tulerpeton*; it even appears to be visible in distal view (Lebedev & Coates, 1995: fig. 5D). A pathetic version of this is seen in the most ossified specimen of *Archeria* (Romer, 1957: fig. 4B, D, 5E). Chase (1965: 200) reported the presence of a "supinator" process (state 1) in *Neldasaurus*. *Trimerorhachis* shows state 1 in presumably subadult and adult specimens (Pawley, 2007); similarly, state 1 is seen in the most mature specimens of *Micromelerpeton* (Boy, 1995). Notably, it is shared by *Ariekanerpeton* (Klembara & Ruta, 2005b: fig. 6A).

Danto, Witzmann & Müller (2012: 49) stated that the "supinator" process is absent in *Solenodonsaurus*. Even as preserved, the process is clearly present (Danto, Witzmann & Müller, 2012: fig. 1, 4, 5A); it may have been longer, with the rest of the impression being part of the lost counterpart.

State 1 is further found in *Captorhinus* (Fox & Bowman, 1966: fig. 28).

CG78 figured a corner in the position where the "supinator" process would be expected (from comparison to Smithson et al., 1994: fig. 12) in *Tuditanus* (CG78: fig. 5D) and *Asaphestera* (CG78: fig. 8E). For *Tuditanus*, this appears to be confirmed by the photograph in Carroll & Baird (1968: fig. 4 right side) – the specimen drawing (fig. 5) disagrees, but is clearly reconstructed in several places. We have tentatively scored state 1 for both *Tuditanus* and *Asaphestera*; the specimens of both should clearly be restudied, however.

Like those of *Apateon*, *Leptorophus*, *Schoenfelderpeton* (Boy, 1986, 1987; Werneburg, 2007a; Schoch, 2014a) and *Discosauriscus* (Klembara & Bartík, 2000), the humerus of **Chroniosaurus* (Clack & Klembara, 2009: fig. 9) and that of **Micropholis* figured by Schoch & Rubidge (2005: fig. 6A) are too poorly ossified to possess a "supinator" process (Pawley & Warren, 2006); we have scored all OTUs mentioned in this paragraph as unknown. Similarly, we have scored *Amphibamus* as unknown: Gregory (1950: 850) described a specimen where the ends of the humeri "are imperfectly ossified"; Daly (1994: fig. 18) presented a photograph of the largest *Amphibamus* specimen, which clearly has completely ossified humeri (with a distal end much wider than in the closely related *Doleserpeton*), but it

remains impossible to determine from this photograph whether "supinator" processes were present.

Preservation and ossification conspire in *Balanerpeton* to make it impossible to determine the state of this character, at least from the published figures (Milner & Sequeira, 1994); we have scored *Balanerpeton* as unknown.

Carroll (1967) stated that his dendrerpetid material lacked the "supinator" process. However, a minimally ossified process might even be present in the incompletely ossified humerus drawn in his fig. 15D, judging from comparison to the less incompletely ossified one of *Ariekanerpeton* which was labeled by Klembara & Ruta (2005b: fig. 6A) as possessing the process. A more or less fully ossified humerus is preserved in the specimen described by Holmes, Carroll & Reisz (1998), but there the area where the process would be has not been prepared out of the matrix. We have scored Dendrerpetidae as unknown.

About *Broiliellus*, Carroll (1964: 200) stated: "The left humerus is complete except for the ectepicondylar process distal to the middle of the articulating surface for the radius." His fig. 10B, however, suggests that the distal end is quite incompletely ossified. We have scored *Broiliellus* as unknown.

Probably not visible in *Eoscopus* (Daly, 1994: fig. 11, 14).

Diplocaulus was scored in RC07 as having state 0; the only figured humerus (Williston, 1909; Douthitt, 1917) is not quite well enough ossified to tell, however, and the ectepicondyle projects so far that state 1 actually seems quite likely. We have changed the score to unknown.

We have kept state 1 for *Orobates*, although the process is very small indeed (Nyakatura et al., 2015: digital model).

The area where the process would be – indeed the entire proximal, preaxial and ventral edge of the humerus – is not ossified in *Pederpes* (Clack & Finney, 2005: fig. 13). We have therefore changed the score to unknown.

The area is preserved in **Casineria*, and the humerus is well ossified, but the specimen is split through the bone so that the "supinator" process, if present, is deeply buried in matrix (D. M., pers. obs. of NMS G 1993.54.1) and cannot be scored.

206. HUM 3: **Sharp-edged ventral humeral ridge: present (0); absent (1).** RC07 had a comma after "sharp-edged", but the ventral humeral ridge is present and blunt in *Edops* (D. M., pers. obs. of MCZ 1781) and *Eryops* (Pawley & Warren, 2006), whose scores we keep as 1.

State 0 is known from *Tulerpeton* and *Eoherpeton* (Milner & Lindsay, 1998), *Whatcheeria* and *Ossinodus* (Bishop, 2014) and *Pederpes* (Clack & Finney, 2005).

Isodectes (D. M., pers obs. of USNM 4471 and USNM 4474), *Trimerorhachis* (Pawley, 2007), *Acheloma* (Polley & Reisz, 2011), *Doleserpeton* (Sigurdsen & Bolt, 2009), *Platyrhinops* (Clack & Milner, 2010), Albanerpetidae (McGowan, 2002), *Eocaecilia* (Jenkins, Walsh & Carroll, 2007), *Diplocaulus* (Williston, 1909; Douthitt, 1917), *Orobates* (Nyakatura et al., 2015: digital model) and *Tseajaia* (Moss, 1972: fig. 9A) have state 1.

Unknown or at least not illustrated or described in *Kotlassia* (Bystrow, 1944: fig. 16).

207. HUM 4: **Latissimus dorsi process confluent with (0) or distinct from (1) deltopectoral crest.**

State 1 is found in *Panderichthys* (Boisvert, 2009), *Isodectes* (D. M., pers. obs. of USNM 4474) and *Ossinodus* (Bishop, 2014).

208. HUM 5: **Entepicondyle foramen: present (0); absent (1).**

State 0 is known in *Hyloplesion* (Olori, 2015) and *Ossinodus* (Bishop, 2014). *Diplocaulus* was scored in RC07 as having state 1, in keeping with the claim that the humerus of *Scincosaurus* "is unique among nectrideans in having an entepicondylar foramen" (Bossy & Milner, 1998: 97); it has state 0 instead (Williston, 1909; Douthitt, 1917). We note that Bossy & Milner (1998) did not mention or illustrate the humerus of *Diplocaulus* at all.

Orobates was correctly (Nyakatura et al., 2015: digital model) scored as having state 0 in RC07, even though the condition was neither described nor illustrated by Berman et al. (2004) and RC07 (appendix 1) did not claim to have seen specimens.

Unknown in *Colosteus* (Hook, 1983).

209. HUM 6: **Ectepicondyle foramen: present (0); absent (1).** See Bishop (2014) for the homology of this feature; the foramen found in many amniotes happens not to occur in the present matrix.

The idea that this character might be size-dependent (loss of the foramen at a certain size: Sequeira, 2009) seems unlikely to us, because very small amphibamids including branchiosaurids lack this foramen as well as the much larger *Eryops*, *Trimerorhachis* and **Sclerocephalus*.

State 1 is found in *Scincosaurus* (Milner & Ruta, 2009) and *Ossinodus* (Bishop, 2014).

Orobates was correctly (Nyakatura et al., 2015: digital model) scored as having state 1, even though the condition was neither described nor illustrated by Berman et al. (2004) and RC07 (appendix 1) did not claim to have seen specimens.

We keep the score of *Cochleosaurus* as unknown; the foramen is present in small humeri, while in the largest one it is absent – if, that is, the quality of the plaster cast that is all which remains of that specimen can be trusted (Sequeira, 2009).

210. HUM 7-8: **Ectepicondyle ridge present, not reaching distal humeral end (0); present, reaching distal end (1); absent (2) (unordered).** State 0, originally HUM 8(0), was originally scored only for *Eusthenopteron*, *Notobatrachus* and *Vieraella* – but the latter two have state 2 instead, which limited state 0 to *Eusthenopteron* and thus made character HUM 8 uninformative. However, *Panderichthys* shares state 0 (Boisvert, 2009).

Platyrhinops has state 1 (Hook & Baird, 1984: fig. 1).

State 2 is known in *Eocaecilia* (Jenkins, Walsh & Carroll, 2007), *Batropetes* (Glienke, 2015: fig. 6) and *Microbrachis* (Olori, 2015).

Unclear in *Eoscopus* (Daly, 1994).

We have changed the score of *Isodectes* from entirely unknown to state 0 or 1. This must be based on D. M.'s pers. obs. of the specimens mentioned for other characters here, but if so, D. M. forgot to document this. The area where the ridge should be is preserved in several specimens, and the distal end is never ossified (D. M., pers. obs.), so the new score is most likely correct.

We have tentatively kept the score of 2 for *Micromelerpeton* and all branchiosaurids, because there is no evidence of another state in the literature; however, we wonder if this could be due to insufficient ossification. Unfortunately, fig. 5b of Werneburg (1991) is not clear enough to tell if it can be known which state the skeletally most mature individual of *Apateon* had.

211. HUM 9: **Distal extremity of ectepicondyle ridge: aligned with ulnar articulation (0); between ulnar articulation and radial condyle (1); aligned with radial condyle (2) (ordered).** This continuous character is inapplicable when the ectepicondyle ridge is absent (HUM 7-8(2)); RC07 already scored accordingly. We use the term "ulnar articulation" instead of "ulnar condyle" because the articular surface for the ulna on the humerus is a trochlea

rather than a condyle in most OTUs of this matrix (see Sigurdsen & Bolt, 2009) to the extent that it is even ossified.

Eucritta (Clack, 2001: fig. 7, in comparison with *Baphetes*: Milner & Lindsay, 1998: fig. 9), *Isodectes* (D. M., pers. obs. of USNM 4471 and USNM 4474), *Platyrhinops* (Hook & Baird, 1984), *Diplocaulus* (Williston, 1909; Douthitt, 1917) and *Silvanerpeton* (Ruta & Clack, 2006) show state 2.

Bishop (2014: 217, fig. 12) reconstructed state 0 in *Ossinodus*.

We tentatively score state 1 or 2 for the incompletely ossified **Erpetosaurus* (Milner & Sequeira, 2011: fig. 8).

Unknown in *Eoscopus* (Daly, 1994: fig. 11, 14).

212. HUM 10: **Humerus without (0) or with (1) waisted shaft.**

Ossinodus has state 0 (Bishop, 2014).

Cochleosaurus (Sequeira, 2009) and *Isodectes* (D. M., pers. obs. of USNM 4471, USNM 4474, USNM 4555, and CM 81430) have state 1, and so do *Cardiocephalus* (CG78: fig. 31), *Microbrachis* (CG78; Olori, 2015) and, somewhat surprisingly, *Diplocaulus* (Williston, 1909; Douthitt, 1917).

Sigurdsen & Green (2011: appendix 2) stated, in agreement with Romer (1946), that *Limnoscelis* lacks a humeral shaft, and changed the score from 1 to 0. The humerus is still, however, strongly constricted in the middle, where the proximal expansion (the head with the deltoid and pectoral processes) and the distal expansion (with the epicondyles and the "supinator" process) meet at a right angle in the widespread tetrahedral shape (Romer, 1946; Berman & Sumida, 1990: 331, fig. 12A; Kennedy, 2010), so we regard *Limnoscelis* as combining state 1 with an unusually short humerus (in particular HUM 12-15(0), see below); humeri with state 0 look quite different. This is also in accordance with the femur, which Berman & Sumida (1990) described as follows: "The proximal and distal ends are widely expanded and joined by an extremely short and moderately stout shaft, giving the femur a deeply waisted appearance."

Sigurdsen & Green (2011: appendix 2) went on to state that "*Seymouria* has a very porrly [sic] developed shaft", which they scored as polymorphic – they appear not to have distinguished polymorphism from partial uncertainty, although we still wonder why they would have wanted to score "partial" uncertainty for a binary character –; White (1939: fig. 23) clearly figured a short waisted shaft, so we retain state 1 here as well.

213. HUM 11: **Position of radial condyle: terminal (0); ventral (1).**

"It seems very likely" that *Ossinodus* had state 0 (Bishop, 2014: 217).

State 1 is documented in *Doleserpeton* (Sigurdsen & Bolt, 2009; D. M., pers. obs. of AMNH 29466), *Eocaecilia* (Jenkins, Walsh & Carroll, 2007), *Archeria* (Romer, 1957: fig. 4D), *Kotlassia* (Bystrow, 1944: fig. 16), *Westlothiana* (Smithson et al., 1994: fig. 12), *Hyloplesion* (Olori, 2015) and *Orobates* (Nyakatura et al., 2015: digital model). Sigurdsen & Green (2011: appendix 2) further reported it for *Triadobatrachus*; this seems at least plausible from the inclination of the ossification front shown in Sigurdsen, Green & Bishop (2012: fig. 7C) and Ascarrunz et al. (2016: 3D model 1). It also seems to be present in *Isodectes* (D. M., pers. obs. of USNM 4555). In *Balanerpeton*, the quite small attachment area for the condyle is well visible on the ventral side (Milner & Sequeira, 1994: fig. 12A, B), and the forelimb of *Dendrysekos* (Dendrerpetidae) described by Holmes, Carroll & Reisz (1998: fig. 8) is preserved in such a strongly flexed position that the probably unossified condyle must have been on the ventral side; we have therefore scored state 1 for both *Balanerpeton* and Dendrerpetidae.

The entire area did not ossify in the known specimens of *Neldasaurus* (Chase, 1965), *Trimerorhachis* (Pawley, 2007; D. M., pers. obs. of TMM 40031-80 and TMM 40031-81), *Discosauriscus* (Klembara & Bartík, 2000), *Ariekanerpeton* (Klembara & Ruta, 2005b) and apparently *Tuditanus* (Carroll & Baird 1968, CG78). Even in *Eoherpeton*, comparison to the best-ossified specimen of *Archeria* (Romer, 1957: 118, fig. 4, 5E; Smithson, 1985: fig. 25) makes it likely that the articular surface for the humerus was incompletely ossified, so we have scored it as unknown as well. The area is furthermore not exposed in *Silvanerpeton* (Ruta & Clack, 2006).

214. HUM 12-15: **Humerus L-shaped, postaxial margin proximal to entepicondyle shorter than or subequal to length of proximal margin of entepicondyle (0); intermediate (1); slender and elongate, total length more than three times maximum width of distal end (2) (ordered).**

HUM 15 was originally called “Width of entepicondyle greater (0) or smaller (1) than half humerus length”. Ruta, Coates & Quicke (2003) did not explain how these measurements should be taken, though the name of the character seems obvious enough. RC07 (p. 106) provided fairly precise instructions: “The entepicondyle width is measured in the plane of the entepicondyle flattening, as the distance between its free margin and a line parallel to the humerus greater axis and passing through the point of attachment of the entepicondyle posterior margin into the general surface of the bone. The humerus length is the maximum distance between its proximal and distal extremities.” If defined this way, state 0 may not occur in this matrix at all. All OTUs that were scored HUM 15(0) have HUM 15(1) according to the usual sources, except apparently *Tulerpeton* (Lebedev & Coates, 1995; but note the incomplete ossification of the head, while the entepicondyle is fully ossified) and maybe *Keraterpeton* (Jaekel, 1903: fig. 3).

Among those previously scored as unknown, HUM 15(0) has not shown up either, while HUM 15(1) is found in *Panderichthys* (Coates, 1996: fig. 35i; Boisvert, Mark-Kurik & Ahlberg, 2008; Boisvert, 2009), *Cochleosaurus* (Sequeira, 2009), *Isodectes* (D. M., pers. obs. of USNM 4471, USNM 4474 and USNM 4555), *Karaurus* (D. M., pers. obs. of unnumbered MNHN cast of PIN 2585/2), *Archeria* (Romer, 1957), *Paleothyris* (D. M., pers obs. of TMM 45955-2, a cast of MCZ 3482), *Ossinodus* (unless the ossification was very unequally incomplete: Bishop, 2014) and *Tseajaia* (Moss, 1972: 32).

Rather than deleting HUM 15 as uninformative, we strongly suspect that the explanation by RC07 is wrong and Ruta, Coates & Quicke (2003) actually measured something else, perhaps the length-to-width ratio of the humerus (width including but not limited to the entepicondyle) or the ratio of the lengths of the proximal margin of the entepicondyle and the adjacent postaxial margin of the humerus proximal to the entepicondyle. For ease of scoring, we have decided in favor of the latter, drawing the line close to a ratio of 1 : 1. State 0 of the present character thus occurs in *Acanthostega* and *Ichthyostega* (Callier, Clack & Ahlberg, 2009), *Tulerpeton* (Lebedev & Coates, 1995), *Greererpeton* (Godfrey, 1989), *Crassigyrinus* (Panchen, 1985), *Whatcheeria* (Lombard & Bolt, 1995), *Baphetes* (Milner & Lindsay, 1998), *Eoherpeton* (Smithson, 1985), *Proterogyrinus* (Holmes, 1984), *Limnoscelis* (Kennedy, 2010), *Keraterpeton* (Jaekel, 1903: fig. 3), *Diceratosaurus* (D. M., pers. obs. of MB.Am.776; note that the proximal end of the humerus is better preserved than drawn by Jaekel, 1903: pl. IV), *Orobates* (Nyakatura et al., 2015: digital model), *Ossinodus* (Bishop, 2014), *Pederpes* (Clack & Finney, 2005) and *NSM 994 GF 1.1 (Holmes & Carroll, 2010). “Defined” this way, HUM 15 had to be merged with HUM 12 (which was defined as absence/presence of state 2 of the present potentially continuous character), because states 0 and 2 of the present character cannot occur in the same humerus. However, the present character remains independent of HUM 16 both in theory (unlike state 0

of the present character, HUM 16(0) does not require a wide entepicondyle, and unlike state 1 of the present character, HUM 16(1) does not require a narrow one) and in practice.

Of the OTUs that were originally scored as unknown for HUM 12 and are not listed above as having state 0, state 1 of the present character is found in *Cochleosaurus* (Sequeira, 2009) and *Isodectes* (D. M., pers. obs. of USNM 4471, USNM 4474, USNM 4555, CM 81512 and CM 81430). *Cardiocephalus* has state 1 or 2 (CG78: fig. 31).

Because of incomplete ossification (Clack, 2001), we cannot determine if *Eucritta* has state 0 or 1.

215. HUM 13: **Posterolateral margin of entepicondyle lying distal relative to plane of radial and ulnar facets: yes (0); no (1).**

Trimerorhachis shows state 0 in large specimens (Pawley, 2007), highlighting the fact that this character can only be scored for well ossified humeri.

We have also assigned this state to *Edops*, because MCZ 1781 appears to show a weak case of it (less weak in extensor view) despite being incompletely ossified (D. M., pers. obs.), and tentatively to *Greererpeton* based on the incompletely ossified humerus pictured in Godfrey (1989: fig. 19g, h). It is further found in *Acanthostega* (Coates, 1996: fig. 15, 16 contra fig. 35j!; Callier, Clack & Ahlberg, 2009: fig. 2B, note that 2C = 4E is reconstructed), at least marginally in *Whatcheeria* (Lombard & Bolt, 1995: fig. 7B), *Ecolsonia* (Berman, Reisz & Eberth, 1985: fig. 11D), *Eoherpeton* (Smithson, 1985: fig. 25), *Proterogyrinus* (Holmes, 1984: fig. 26), *Archeria* (Romer, 1957), *Pholiderpeton scutigerum* (borderline, but incompletely ossified: Clack, 1987b: fig. 30), apparently *Solenodonsaurus* (Danto, Witzmann & Müller, 2012: fig. 5A), *Kotlassia* (Bystrow, 1944: fig. 16), *Seymouria* (White, 1939: fig. 23), *Diadectes* (Berman, Sumida & Martens, 1998), *Limnoscelis* (Berman & Sumida, 1990; Kennedy, 2010), *Captorhinus* (just barely: Fox & Bowman, 1966: fig. 28), *Sauropleura* (Bossy & Milner, 1998: fig. 66C) and *Orobates* (Nyakatura et al., 2015: digital model).

State 1 is found in *Panderichthys* (Coates, 1996: fig. 35i; Boisvert, Mark-Kurik & Ahlberg, 2008; Boisvert, 2009) and *Karaurus* (D. M., pers. obs. of unnumbered MNHN cast of PIN 2585/2). We have kept the score of 1 for *Ichthyostega* because, although the entepicondyle projects distal to the ulnar facet, the radial facet is so much more proximal – much like in *Panderichthys* (Ahlberg, 2011) – that the entepicondyle at most reaches the oblique plane in which the two facets lie (Callier, Clack & Ahlberg, 2009: fig. 1, 4D); this situation is in fact quite similar to that seen in *Paleothyris* (Carroll, 1969b: fig. 7E).

Unknown due to insufficient ossification (i.e. absence of the posterolateral margin of the entepicondyle) in *Crassigyrinus* (Panchen, 1985: fig. 22), *Baphetes* (Milner & Lindsay, 1998), *Eucritta* (Clack, 2001), *Neldasaurus* (Chase, 1965: fig. 12), *Balanerpeton* (Milner & Sequeira, 1994), Dendrerpetidae (Carroll, 1967; Holmes, Carroll & Reisz, 1998), *Apateon* (Werneburg, 1991: fig. 5b), *Triadobatrachus* (Sigurdsen, Green & Bishop, 2012: fig. 7C; Ascarrunz et al., 2016: 3D model 1), *Discosauriscus* (Klembara & Bartík, 2000), *Brachydectes* (Wellstead, 1982), *Diplocaulus* (Williston, 1909; Douthitt, 1917), *Ariekanerpeton* (Klembara & Ruta, 2005b), *Pederpes* (Clack & Finney, 2005), *Silvanerpeton* (Ruta & Clack, 2006) and *Utegenia* (Klembara & Ruta, 2004b).

Unclear (at least from published illustrations) in *Ptyonius* and *Urocordylus* (Bossy & Milner, 1998: fig. 66A, B) as well as *Tseajaia* (where the text and the illustration of Moss [1972] appear to contradict each other, and the illustration is at an oblique angle).

216. HUM 14: **Posterolateral margin of the entepicondyle markedly concave: yes (0); no (1).** Apparently this describes whether the distal margin is straight or forms a distal hook. If so, *Panderichthys* has state 0 (Boisvert, 2009).

Cochleosaurus (Sequeira, 2009), *Eoscopus* (Daly, 1994: fig. 11, 14) and *Karaurus* (D. M., pers. obs. of unnumbered MNHN cast of PIN 2585/2) have state 1.

Insufficiently ossified to score in *Triadobatrachus* (Ascarrunz et al., 2016: 3D model 1).

217. HUM 16: **Portion of humerus […] length proximal to entepicondyle smaller (0) or greater (1) than humerus head width.**

State 0 looks very likely in *Ossinodus* (Bishop, 2014).

State 1 is found in *Eusthenopteron* (just barely: Coates, 1996: fig. 35h), *Panderichthys* (Coates, 1996: fig. 35i; Boisvert, Mark-Kurik & Ahlberg, 2008), *Baphetes* (perhaps just barely: Milner & Lindsay, 1998), *Edops* (D. M., pers. obs. of MCZ 1781), *Isodectes* (D. M., pers. obs. of USNM 4471, USNM 4474 and USNM 4555), *Balanerpeton* (Sequeira & Milner, 1994: fig. 12), Dendrerpetidae (Holmes, Carroll & Reisz, 1998: fig. 8), *Eryops* (Pawley & Warren, 2006), *Karaurus* (D. M., pers. obs. of unnumbered MNHN cast of PIN 2585/2), *Solenodonsaurus* (Danto, Witzmann & Müller, 2012), *Kotlassia* (Bystrow, 1944: 409), *Paleothyris* (D. M., pers obs. of TMM 45955-2, a cast of MCZ 3482), *Cardiocephalus* (CG78: fig. 31), *Diplocaulus* (Williston, 1909; Douthitt, 1917) and *Orobates* (Nyakatura et al., 2015: digital model).

We have scored *Eucritta* as unknown because the measurements are about equal (Clack, 2001) and the proximal end of the humerus seems incompletely ossified.

The state in the largest specimen of *Cochleosaurus* is unclear; smaller ones are rather borderline (Sequeira, 2009). We have therefore kept its score as unknown. *Proterogyrinus* is likewise borderline (Holmes, 1984: fig. 26), so we have scored it as unknown as well; taking the width in fig. 26(c) at face value, *P.* would just barely have state 1, rather than 0 as originally scored.

Unknown in *Scincosaurus* (Milner & Ruta, 2009).

218. HUM 17: **Accessory foramina on humerus: present (0); absent (1).**

State 1 occurs in *Tulerpeton* (Lebedev & Coates, 1995), *Cochleosaurus* (Sequeira, 2009), *Paleothyris* (D. M., pers obs. of TMM 45955-2, a cast of MCZ 3482) and *Ossinodus* (Bishop, 2014). Germain (2008a) correctly scored state 1 in *Isodectes* (D. M., pers. obs. of USNM 4471, USNM 4474, and USNM 4555), but did not mention this in the text; instead he intended (Germain, 2008a: 185) to change the score of the next character from unknown to 1, but did not do this in the matrix. Evidently, the right score ended up in the wrong column.

219. HUM 18/DIG 1: **Forelimb absent (0); humerus present, length smaller (1) or greater (2) than combined length of two and a half mid-trunk vertebrae (ordered).** State 0 corresponds to part of state DIG 1(0) of RC07; for the other part see state DIG 1-2-3-4(0) in our ch. 276. States 1 and 2 correspond to states HUM 18(0,1) of RC07 – who had exchanged the states in the text but not in the matrix, except maybe for the taxa they added.

State 2 is found in *Whatcheeria* (strongly implied by Lombard & Bolt, 1995), *Baphetes* (Milner & Lindsay, 1998 – the longest intercentrum is 22 mm long, the humerus somewhere around 85; this should ensure state 2 even if the vertebrae were very rhachitomous), *Eucritta* (Clack, 2001), *Edops* (Romer & Witter, 1942), *Cochleosaurus* (Sequeira, 2009), *Isodectes* (Sequeira, 1998; Germain, 2008a: 185 – see HUM 17; D. M., pers. obs. of CM 81512), *Acheloma* (Polley & Reisz, 2011), *Phonerpeton* (Dilkes, 1990), *Ecolsonia* (Berman, Reisz & Eberth, 1985), *Doleserpeton* (Sigurdsen & Bolt, 2010), *Leptorophus* (Boy, 1986; Werneburg, 2007a), *Triadobatrachus* (all literature and pers. obs.), *Eoherpeton* (Smithson, 1985: compare fig. 19, 24, 25), *Pholiderpeton scutigerum* (Clack, 1987b), *Diadectes* (all literature), *Cardiocephalus* (CG78: fig. 31), *Notobatrachus* and *Vieraella* (Estes & Reig,

1973; Báez & Basso, 1996), *Orobates* (Berman et al., 2004: fig. 1), *Pederpes* (Clack & Finney, 2005), *Silvanerpeton* (Ruta & Clack, 2006), and *Tseajaia* (Moss, 1972: pl. 2; D. M., pers. obs. of CM 38033). We have kept state 1 or 2 for the very poorly ossified *Schoenfeld-erpeton* (Boy, 1986).

RC07 scored *Acherontiscus* and all adelogyrinids as unknown for the presence of limbs; Ruta, Coates & Quicke (2003: 263) commented on the adelogyrinids (there is no comment specifically on *Acherontiscus*): "Limb absence may well be a preservational artifact, especially because of the very few specimens known." However, we find it difficult to imagine that the forelimbs and, notably, the endochondral shoulder girdle would have just fallen off the specimen of *Adelogyrinus* drawn by Andrews & Carroll (1991: fig. 6), where the ribs, the dermal shoulder girdle and even the hyobranchial apparatus are hardly disarticulated and well preserved. Andrews & Carroll (1991: 252) commented on that specimen: "No unidentified bone is present in the area of the dermal shoulder girdle and none could possibly be confused with the endochondral girdle or fore limbs. All bone present is well preserved, although it has suffered surface damage. The specimen is broken through the area of the shoulder girdle so that a cross-section of the bones is visible. If the endochondral shoulder girdle had been ossified, it is difficult to imagine how it could fail to be preserved with the rest of the girdle, ribs and vertebrae. One can only assume that these bones were unossified (cartilaginous) or missing in the living animal. The scapulocoracoid is slow to ossify in small labyrinthodonts and microsaurs, but the remainder of the skeleton suggests that this specimen is mature. The dermal elements are typical of other early tetrapods in their proportions and configuration." We have therefore scored *Adelogyrinus* as limbless (state 0), although we have kept the question mark for the less well articulated *Acherontiscus*, the much less well articulated *Adelospondylus*, and of course *Dolichopareias* which is exclusively known from skull bones.

**Utaherpeton* changes from state 2 to state 1 in ontogeny (Carroll & Chorn, 1995: table 1). We have only considered the adult condition.

**Sclerocephalus* has state 2, but just barely (Schoch & Witzmann, 2009a: fig. 9D), at least as far as the ossified part of the humerus is concerned.

In spite of having only 13 presacral vertebrae, **Chelotriton* has state 1 (Schoch, Poschmann & Kupfer, 2015), showing that state 1 does not always correlate with trunk elongation.

220. HUM 19: **Process '2' on humerus: absent (0); present (1).** Sigurdsen & Green (2011) suspected that this process is homologous to the latissimus dorsi process, but they are both present and lie on different sides of the ectepicondyle ridge in *Acanthostega* (Coates, 1996: fig. 16) and *Ossinodus* (Bishop, 2014) for example; Pawley (2006: fig. 72.2.1) figured and labeled them both on a humerus of *Proterogyrinus*.

Isodectes has state 0 (D. M., pers. obs. of USNM 4471 and USNM 4474).

Panderichthys and even *Eusthenopteron* have a candidate process (figures in Boisvert, 2009, and Ahlberg, 2011) which we have scored as state 1.

In *Acanthostega*, process 2 seems to disappear during ontogeny (Callier, Clack & Ahlberg, 2009: fig. 2), which is not known to happen in any other taxon; we have kept state 1 for the time being because process 2 is not mentioned in the figures, text or supplementary information of Callier, Clack & Ahlberg (2009) and because the drawing is not completely unambiguous, but we would like to draw attention to this question.

Ichthyostega was scored in RC07 as having state 0; but the term "process 2" was first invented for *Ichthyostega* (Jarvik, 1996: 62). In *Ichthyostega*, unlike in the other OTUs that have it, process 2 is only identifiable in postaxial/posterior view (Jarvik, 1996: fig. 45), hiding between the ectepicondyle ridge ("dorsal ridge" of Jarvik, 1996) and the entepicondyle; in

extensor/dorsal view, the large process 1 covers it, while in flexor/ventral view, it lies behind the oblique ridge that forms the proximal edge of the entepicondyle (compare Jarvik, 1996: fig. 45F and Callier, Clack & Ahlberg, 2009: fig. 1 left side for two slightly different interpretations of exactly which angle is "ventral view"). It is not common for the humeri of early tetrapods to be figured in postaxial/posterior view; Callier, Clack & Ahlberg (2009) and Ahlberg (2011), for example, did not bother. – In short, *Ichthyostega* has state 1.

Tulerpeton was scored as unknown, and RC07 stated without further comment that the presence of process 2 was "uncertain". Ruta & Clack (2006), however, claimed that *Tulerpeton* had state 1 and cited Lebedev & Coates (1995). The latter did not mention process 2; from their fig. 5 it seems that a rather indistinct process is present. We have therefore changed the score to 1.

The process of *Crassigyrinus* that Panchen (1985) identified as the insertion point of M. subscapularis and Lebedev & Coates (1995: fig. 6F) labeled as the insertion point of M. latissimus dorsi appears to be process 2 by comparison to *Acanthostega* (Coates, 1996; Callier, Clack & Ahlberg, 2009) and *Ossinodus* (Bishop, 2014).

It is unclear from Milner & Lindsay (1998: fig. 9) whether *Baphetes* had a small process 2; we have changed the score to unknown.

Not preserved in *Eucritta*, apart from the early ontogenetic stage of the individual (Clack, 2001: fig. 7).

Fig. 13 of Romer & Witter (1942) is a reconstruction; the area where process 2 would be is not preserved in any known specimen of *Edops* (D. M., pers. obs.), which we have therefore scored as unknown.

State 1 is further known in *Ossinodus* (Bishop, 2014); *Silvanerpeton* has a possibly slightly less weak version of state 1 than *Tulerpeton* (Ruta & Clack, 2006).

About *Pederpes*, Clack & Finney (2005: 330) stated: "There is no equivalent to 'process 2' of *Ichthyostega* (Jarvik, 1996) or *Acanthostega* (Coates, 1996)." We do not think, however, that this can be stated with reasonable certainty when the area where the process would be has not been fully prepared and the humerus was even less well ossified (Clack & Finney, 2005: fig. 13) than that of *Ossinodus* (Bishop, 2014). We have therefore changed the score to unknown.

221. RAD 1: **Radius or radioulna longer (0) or shorter (1) than humerus.**

Baphetes has state 1 (Milner & Lindsay, 1998), as do *Edops* (D. M., pers. obs. of MCZ 1769), *Cochleosaurus* (Sequeira, 2009), *Isodectes* (D. M., pers. obs. of USNM 4471, USNM 4474, USNM 4555 and CM 81430), *Doleserpeton* (Sigurdsen & Bolt, 2009, 2010), *Cardiocephalus* (CG78: fig. 31), *Notobatrachus* (Báez & Nicoli, 2004) and *Vieraella* (Báez & Basso, 1996).

222. RAD 2: **Radius longer than (0), as long as (1), or shorter than (2) ulna (ordered).** This is a continuous character.

State 1 is found in *Doleserpeton* (Sigurdsen & Green, 2011: appendix 2), Albanerpetidae (McGowan, 2002), *Eocaecilia* (Jenkins, Walsh & Carroll, 2007) and apparently *Cardiocephalus* (CG78: fig. 33C).

Edops has state 2 (D. M., pers. obs. of MCZ 1769), as do *Cochleosaurus* (Sequeira, 2009), *Isodectes* (D. M., pers. obs. of USNM 4471 and CM 81430), *Trimerorhachis* (Pawley, 2007), *Triadobatrachus* (Ascarrunz et al., 2016: 3D model 1), *Valdotriton* (Evans & Milner, 1996: fig. 7), *Microbrachis* (Olori, 2015: fig. 20C, D, 27C) and *Diplocaulus* (Williston, 1909; Douthitt, 1917). Radius and ulna remain distinguishable in a fused radioulna (RAD 3(1)); state 2 is shared by *Notobatrachus* (Báez & Nicoli, 2004) and probably *Vieraella* (Báez & Basso, 1996: fig. 6).

Olori (2015: 58, table S3) has stated that *Microbrachis* and *Hyloplesion* have state 1. This must reflect a more generous definition of that state than what we have used. As mentioned, we consider three photographs of *Microbrachis* in Olori (2015) to show state 2. The most mature forearm of *Hyloplesion* depicted in that study, Olori (2015: fig. 33C), indeed shows state 1, but the ulna has almost no olecranon, and the caption states: "Beyond this [ontogenetic] stage, the olecranon becomes a rounded, distinct process." This strongly implies that state 2 was reached later in ontogeny. For this reason we have kept state 2 for *Hyloplesion*, which is unambiguously depicted in the drawings in CG78: fig. 90D, E.

Unknown in *Solenodonsaurus* (Danto, Witzmann & Müller, 2012) and *Euryodus* (CG78).

As preserved, **Erpetosaurus* has state 1 (Milner & Sequeira, 2011: fig. 8), but scoring it that way would assume that the olecranon process is fully ossified, which is unlikely in the light of the clearly incompletely ossified humerus. We have therefore scored state 1 or 2.

From Paton, Smithson & Clack (1999: fig. 3) it may seem that **Casineria* has state 2; however, radius and ulna are so incompletely ossified and preserved (split lengthwise; D. M., pers. obs. of NMS G 1993.54.1p) that we have to keep the score as unknown.

223. RAD 3: **Compound radio-ulna: absent (0); present (1).**

Baphetes is known to have state 0 (Milner & Lindsay, 1998), as are *Edops* (D. M., pers. obs. of MCZ 1769), *Cochleosaurus* (Sequeira, 2009), *Isodectes* (D. M., pers. obs. of USNM 4471, USNM 4474 and CM 81430) and *Cardiocephalus* (CG78: fig. 31).

224. ULNA 1: **Olecranon process: absent (0); present (1).**

Incompletely ossified proximal ends of ulnae that do not show a process should of course not be scored 0, but unknown. This affects *Crassigyrinus* (Panchen, 1985), *Schoenfelderpeton* (Boy, 1986: fig. 10), *Pederpes* (Clack & Finney, 2005), *Silvanerpeton* (Ruta & Clack, 2006: fig. 6), and even the most mature known specimens of *Discosauriscus* (Klembara & Bartík, 2000) and *Utegenia* (Klembara & Ruta, 2004b) that preserve an ulna. In *Leptorophus*, a rudimentary process might sometimes be present (Boy, 1986: fig. 10), but more likely the ulna is again too incompletely ossified to tell (supported for *Leptorophus* by Werneburg, 2007a, and Schoch, 2014a). The same holds for *Micromelerpeton* (Boy, 1972: 37, fig. 20) and *Odonterpeton* (CG78: 147, fig. 98, 99). We have scored all OTUs mentioned in this paragraph as unknown.

Edops (D. M., pers. obs. of MCZ 1769 and MCZ 7143), *Cochleosaurus* (Sequeira, 2009), *Isodectes* (D. M., pers. obs. of USNM 4471 and CM 81430), *Apateon* (metamorphosed *A. gracilis*: Fröbisch & Schoch, 2009b), Albanerpetidae (*Celtedens ibericus*: McGowan, 2002: fig. 6C), *Eocaecilia* (Jenkins, Walsh & Carroll, 2007), *Microbrachis* (Olori, 2015), *Diplocaulus* (Williston, 1909; Douthitt, 1917) and *Ariekanerpeton* (Klembara & Ruta, 2005b) have state 1. Apparently, so do the incompletely ossified *Valdotriton* (Evans & Milner, 1996: fig. 7, 8) and the incompletely preserved *Cardiocephalus* (CG78: fig. 31).

"In *Acanthostega* the ulna lacks an olecranon process" (Coates, 1996: 386), and indeed *Acanthostega* was scored as having state 0 in RC07. But on the same page (Coates, 1996: fig. 17g–l), the left ulna of *Acanthostega* is illustrated as having a quite large process that continues the large flange proximal to the ossification front of the proximal articular end. Comparison to the right ulna of *Greererpeton* (Godfrey, 1989: fig. 20g–l) and to skeletal restorations (Godfrey, 1989: fig. 1c; Coates, 1996: fig. 18) does not show any reason not to consider this process homologous to an olecranon process. The only clear difference to *Greererpeton* (scored 1 in RC07) is that, in *Acanthostega*, the proximal surface of this process is entirely unfinished (it looks like a cross-section) and continuous with the equally unfinished surface of the articulation for the humerus; this is not a sufficient reason to score state 0. We

thus agree with Ahlberg (2011) that the olecranon process is present in *Acanthostega* and have changed the score to 1.

Somewhat similarly, *Notobatrachus* should be considered to have state 1 (Báez & Nicoli, 2004: 167, fig. 2, 4, 5).

CG78 stated on p. 32 that there is no "distinct olecranon" in **Llistrofus*. The base of an olecranon process is, however, shown in their fig. 15, so we have scored state 1. Given the wide-open skull sutures, the neurocentral sutures, the absence of preserved carpals and the apparently rather featureless radius, we consider the lack of further ossification of the olecranon to be most likely a juvenile feature.

225. ILI 3: **Dorsal iliac process: absent (0); present (1).** Judging from various "microsaurs" like *Tuditanus* and *Sparodus*, or even dinosaurs like *Bagualosaurus* (Pretto, Langer & Schultz, 2018), this process is homologous to the preacetabular process of (many) amniotes.

Frogs generally have only one process which is directed cranially. We wonder if the tuber superius of *Triadobatrachus*, *Notobatrachus*, ***Gobiates* and **some other salientians (Roček & Rage, 2000; Ascarrunz et al., 2016; Báez & Nicoli, 2004; Roček, 2008) – though not all others; **Liaobatrachus* lacks it (Dong et al., 2013) – is homologous to the caudal process, and have therefore scored the two mentioned OTUs as having state 1.

Ventastega shares state 1 (Ahlberg et al., 2008). So do *Edops* (D. M., pers. obs. of MCZ 6489; also implied by Romer & Witter, 1942), *Chenoprosopus* (Hook, 1993; D. M., pers. obs. of USNM 437646), *Isodectes* (D. M., pers. obs. of USNM 4474), *Neldasaurus* (Chase, 1965), *Trimerorhachis* (well developed: Pawley, 2007), Dendrerpetidae (less distinct, but still clear: Milner, 1996; Holmes, Carroll & Reisz, 1998; Pawley, 2006: 183, fig. 58), *Acheloma* (Olson, 1941), *Broiliellus* (D. M., pers. obs. of MCZ 3272; also suggested by the illustration of that specimen in Carroll, 1964: fig. 11), *Doleserpeton* (Sigurdsen & Bolt, 2010), *Eoscopus* (Daly, 1994), *Platyrhinops* (Hook & Baird, 1984; Werneburg, 2012a; D. M., pers. obs. of AMNH 2002), *Apateon* (Werneburg, 1991: fig. 8d), *Leptorophus* (Werneburg, 2007a: fig. 3), *Batropetes* (Glienke, 2013, 2015), *Saxonerpeton*, *Hapsidopareion*, *Micraroter* and *Euryodus* (CG78: fig. 126), *Pelodosotis* (CG78: 85), *Hyloplesion* (in later ontogeny: Olori, 2015), *Scincosaurus*, *Diceratosaurus*, *Ptyonius*, *Sauropleura* and *Urocordylus* (Bossy & Milner, 1998: fig. 67), and *Ariekanerpeton* and *Utegenia* (Klembara & Ruta, 2004b, 2005b). An extremely short but otherwise well developed dorsal process is found in **Chroniosaurus* (Clack & Klembara, 2009: fig. 8, left side); we have scored this as state 1 as well.

Eryops was scored as having state 0 in RC07. However, if anything, the caudal process is absent, not the dorsal one (Romer, 1957: 117; Pawley, 2006: 183, fig. 58; Pawley & Warren, 2006; D. M., pers. obs. of specimens in USNM, TMM, AMNH and elsewhere), leading us to score state 1. The caudal process may well be present as the corner called "sacral flange" by Pawley (2006: fig. 58) and Pawley & Warren (2006).

The albanerpetid *Celtedens* appears to have a dorsal process and maybe a small caudal one (McGowan, 2002: fig. 9); we have therefore assigned state 1 to Albanerpetidae.

The unusual-looking fig. 8 of Watson (1940), recommended by Daly (1994), suggests that *Amphibamus* may have had state 1 rather than 0 as scored by RC07, especially when compared to *Eoscopus* (Daly, 1994: fig. 13); we have scored it as unknown. "[T]he rear parts of the body are missing" in all known specimens of *Schoenfelderpeton* (Boy, 1986: 155; translated by D. M.), almost the entire ilium is unknown in *Valdotriton* (Evans & Milner, 1996), and the situation is unclear (probably the ilium is too poorly ossified) in *Keraterpeton* (Bossy & Milner, 1998: fig. 67).

The ilium of *Cochleosaurus* (Sequeira, 2009) apparently has an unusual ontogeny which begins at a shape very similar to that of *Eryops* but ends at a long, almost parallel-

sided, caudodorsally directed rod as seen in *Isodectes* and **Erpetosaurus*. However, as we count both of those conditions as state 1, we have assigned state 1 to *Cochleosaurus* as well.

In **Gerobatrachus*, "[t]he ilium lacks the posterior process common to temnospondyls but the presence of an anterior process, a salientian character, is obscured by an overlying fragment of the femur" (Anderson et al., 2008a: 516). Because the caudal process is absent, we have tentatively scored state 1.

The situation in **Micropholis* is borderline, but we interpret the low dorsal bulge (Schoch & Rubidge, 2005: fig. 6G) as the dorsal process and have thus scored state 1.

226. ILI 4: **Caudal iliac process tapers to a single point or is rounded (0); ends in two corners (1).** The original wording, "Posterior iliac process subhorizontal, stout, abbreviated posteriorly and tapering rearward in lateral aspect: absent (0); present (1)", is a garbled hash of four statements on size and shape, using "abbreviated" to indicate that the process is metaphorically 'shortened' by being metaphorically 'cut off', giving it a more or less caudal edge (sometimes vertical as in *Limnoscelis* and *Discosauriscus*) with two corners instead of one. Notably, caudal processes with state 1 usually do not taper, but have a more or less constant width throughout. – Judging from various "microsaurs" like *Tuditanus* and *Sparodus*, this process is homologous to the postacetabular process of (many) amniotes.

State 0 is present in *Chenoprosopus* (Hook, 1993; D. M., pers. obs. of USNM 437646), *Cochleosaurus* (Sequeira, 2009), *Isodectes* (D. M., pers. obs. of USNM 4474), *Platyrhinops* (Hook & Baird, 1984), *Leptorophus* (Werneburg, 2007a) and *Kotlassia* (Bystrow, 1944: 409).

State 1, at least under our definition, is found in *Petrolacosaurus* (Reisz, 1981), probably *Westlothiana* (Smithson et al., 1994: fig. 11A, 14A, B, 15B), and, more surprisingly, *Acheloma* (Olson, 1941: fig. 12A), *Archeria* (Romer, 1957), *Sauropleura* (Bossy & Milner, 1998), **Archegosaurus* (Witzmann & Schoch, 2006), **Mordex* (lectotype: Werneburg, 2012a: fig. 31f) and **Australerpeton* (Eltink & Langer, 2014). By comparison to younger individuals of **Mordex* (Werneburg, 2012a: fig. 31b–d), we have scored **Branchiosaurus* (Werneburg, 2012a: fig. 30b, d, f) as unknown.

Batropetes appears to be polymorphic (Glienke, 2015: fig. 5).

Unknown in *Ecolsonia* (Berman, Reisz & Eberth, 1985: fig. 12A); almost the entire ilium is unknown in *Valdotriton* (Evans & Milner, 1996). If the tuber superius of *Triadobatrachus* is homologous to the caudal iliac process, it is nonetheless too small and possibly incompletely ossified for this character to be applicable (Ascarrunz et al., 2016: 3D model 1).

227. ILI 6: **Supr[…]acetabular iliac buttress less (0) or more (1) prominent than postacetabular buttress.**

State 0 is found in *Orobates* (Nyakatura et al., 2015: digital model).

Cochleosaurus has state 1 (Sequeira, 2009), as do *Doleserpeton* (D. M., pers. obs. of BEG 40882-25), apparently *Platyrhinops* (Hook & Baird, 1984) and (weakly expressed) *Triadobatrachus* (Ascarrunz et al., 2016: 3D model 1). We have also kept this state for *Eoscopus*, because Daly (1994: 17, fig. 13) mentioned and possibly drew "projecting rims dorsally and ventrally" on the acetabulum, with no indication of a caudal one; Daly (1994: 17) went on to state that "there is no supra-acetabular buttress", but clearly such a buttress and the projecting dorsal rim are homologous.

Eryops was scored as having state 1 in RC07. Pawley & Warren (2006: figs. 8.2 and 8.4, based entirely on specimens not mentioned below) reconstructed state 0 instead. This is not likely to be ontogenetic, because the large specimens TMM 31225-3 and TMM 31225-9 show state 1 while the likewise large MCZ 1126, MCZ 1536, MCZ 2588 and MCZ 2638 share state 0 with the middle-sized MCZ 2682 and the small TMM 31225-33, though other

differences between the large specimens leave open the possibility of sexual or similar dimorphism. Such dimorphism is well known for the skulls, which are not preserved in these specimens: there is a narrow-headed and a round-headed morph independently of absolute size (Werneburg, 2007b; D. M., pers. obs. of USNM, TMM, AMNH and MCZ specimens; considered two species by Schoch & Milner, 2014). In any case, we have scored *Eryops* as polymorphic.

Diagenetic crushing can further make this character difficult to code; the left side of the *Diadectes* specimen AMNH 23761 shows (if somewhat borderline) state 1 as scored, while on the right side the ilium is bent ventromedially, creating state 0. (We have kept state 1 for *Diadectes*.)

The postacetabular buttress is unossified in *Chenoprosopus* (Hook, 1993: fig. 3B; D. M., pers. obs. of USNM 437646) and *Trimerorhachis* (Pawley, 2007). The same appears to be the case in **Sclerocephalus* (Schoch & Witzmann, 2009a: fig. 8F), **Archegosaurus* (Witzmann & Schoch, 2006b: fig. 7), **Erpetosaurus* (Milner & Sequeira, 2011: fig. 10A), and to a lesser extent **Australerpeton* (Eltink & Langer, 2014).

228. ILI 7: **Transverse pelvic ridge: absent (0); present (1).**

State 0 is found in *Ventastega* (Ahlberg et al., 2008) and *Cochleosaurus* (Sequeira, 2009).

State 1 is documented in *Edops* (D. M., pers. obs. of MCZ 6489), in at least some large specimens of *Trimerorhachis* (Pawley, 2007) and in *Acheloma* (Polley & Reisz, 2011), and was apparently also present in *Chenoprosopus* (Hook, 1993; D. M., pers. obs. of USNM 437646), *Kotlassia* (Bystrow, 1944: fig. 17) and *Tuditanus* (D. M., pers. obs. of CM 29592). We further agree with Romer (1957) that the transverse ridge of *Ichthyostega* (Jarvik, 1996) constitutes state 1.

229. ILI 9: **Ilium shaped like an elongate rod directed anteriorly/anterodorsally: absent (0); present (1).** We have deliberately kept this character independent of the putative homology of the rod, in particular the question of whether it is the dorsal or the caudal process (ILI 3), in order to allow the homology hypotheses to compete.

State 0 is present in *Ventastega* (Ahlberg et al., 2008), *Edops* (D. M., pers. obs. of MCZ 6489), *Chenoprosopus* (Hook, 1993), *Cochleosaurus* (Sequeira, 2009), *Isodectes* (D. M., pers. obs. of USNM 4474), *Platyrhinops* (Hook & Baird, 1984; Clack & Milner, 2010), *Leptorophus* (Werneburg, 2007a) and probably *Cardiocephalus* (CG78: fig. 31).

"[T]he rear parts of the body are missing" in all known specimens of *Schoenfelderpeton* (Boy, 1986: 155; translated by D. M.); almost the entire ilium is unknown in *Valdotriton* (Evans & Milner, 1996).

**Tungussogyrinus* has an intermediate condition that we count as state 1, following Werneburg (2009). The same appears to be the case in **Beiyanerpeton* (Gao & Shubin, 2012: fig. 2).

deleted ILI 10: **Acetabulum directed posteriorly/posterolaterally (0) or laterally (1).** As RC07 pointed out, this character is parsimony-uninformative, so we have deleted it.

Boisvert (2005) stated three times that *Panderichthys*, which was scored as unknown in RC07, has state 0, referring twice to her fig. 1d. That, however, is a line drawing which does not show the acetabulum, but instead shows the whole area as damaged. The photograph (Boisvert, 2005: fig. 1c) which the drawing interprets appears to show that the entire surface of the pelvis and of the proximal half or so of the femur is damaged; no acetabulum can be discerned. We therefore keep the score as unknown; state 0 thus remains restricted to *Eusthenopteron*, and this character remains parsimony-uninformative.

"[T]he rear parts of the body are missing" in all known specimens of *Schoenfelderpeton* (Boy, 1986: 155; translated by D. M.).

deleted ISC 1: **Ischium contributing to pelvic symphysis: no (0); yes (1).** As RC07 pointed out, this character is parsimony-uninformative, so we have deleted it.

230. PUB 1: **Number of pubic obturator foramina: multiple (0), single (1), or absent (2) (ordered).** We have ordered this meristic character.

Eryops (Pawley & Warren, 2006), *Acheloma* (Polley & Reisz, 2011), *Phonerpeton* (D. M., pers. obs. of MCZ 1548), *Doleserpeton* (Sigurdsen & Bolt, 2010), *Eoscopus* (Daly, 1994) and *Cardiocephalus* (CG78: fig. 31) display state 1.

The three pubes catalogued as MCZ 7158 have been assigned to *Edops*. Only one of them clearly shows a foramen. Right next to it (craniodorsally), there is another hole of about the same size; however, we interpret it as damage, because it seems to have a solid floor of spongy bone (unlike the more likely foramen, which is filled with matrix to at least a deeper level) and because the bone surface around it, including the area between the two holes, is damaged (D. M., pers. obs.). We have therefore scored state 1 for *Edops*.

Platyrhinops has state 1 or 2; we can exclude the possibility of state 0 (D. M., pers. obs. of AMNH 2002).

Batropetes palatinus shows states 1 and 2 in different specimens (Glienke, 2015). Because only state 1 is documented in the other three species (Glienke, 2013, 2015), we have kept the score of 1, although this may yet turn out to be an artefact: *B. niederkirchensis* and *B. appelensis* are known from a single specimen each, and the specimens of *B. fritschi* and *B. appelensis* are less well preserved and ossified than those of the other two species (Glienke, 2013, 2015).

Clearly, the absence of pubic bones in the **Lydekkerina* specimens described by Hewison (2008) is due to immaturity, while the specimen described by Pawley & Warren, (2005) is more mature and has a short but ossified pubis which bears an obturator foramen on its ventral surface (Pawley & Warren, 2005: fig. 6F–H). We have scored state 1.

231. FEM 1-2-6: **Internal trochanter does not project (0); projects proximally, enclosing (with the head) an indentation (often rounded) in the "ventral" margin of the femur (1); projects only "ventrally", so that its proximal edge forms an angle of at least 90° with the shaft (2) (unordered).** It may be further possible to distinguish lack of projection (which appears to occur e.g. in *Ichthyostega*; Pierce, Clack & Hutchinson, 2012: supplementary movies) from complete absence, but it appears that absence is only documented in *Eusthenopteron* in the present taxon samples (Coates, 1996: fig. 36i).

State 2 is our reinterpretation of FEM 6(1), which was scored only for *Eocaecilia* and *Valdotriton* in RC07. In those two OTUs (Evans & Milner, 1996; Jenkins, Walsh & Carroll, 2007), the internal trochanter is very large in relation to the femoral head, projects "ventrally", and is not continued distally by an adductor blade, making it even more conspicuous; but the lack of the adductor blade seems to be the only distinction to the condition found in *Eryops* (Pawley & Warren, 2006; D. M., pers. obs. of TMM 31227-11 and TMM 31227-14), *Diadectes* (Berman, Sumida & Martens, 1998: fig. 18A) or *Ossinodus* (Warren & Turner, 2004), to all of which we have therefore also assigned state 2. We note that FEM 6(0) was redundantly scored instead of FEM 6(?) for all OTUs with FEM 1(0) – "Internal trochanter raised as a distinct protuberance: absent" – except *Notobatrachus*.

The original FEM 2 was confusing: its name was "Internal trochanter separated from the general surface of the femur shaft by a distinct, trough-like space: absent (0); present (1)", but the explanation (RC07: 107) referred to "a deeply notched web of bone", strongly

implying that the "trough-like space" lies proximodorsal to the trochanter, between it and the head, rather than "dorsal" or "ventral" from it. Complete absence of this notch is indistinguishable from FEM 1(0), while conversely the notch can occupy the entire space where the web would be, which is always the case in taxa with state 2 of the present merged character – including *Eocaecilia* and *Valdotriton* (see above). Clearly, the three characters had to be merged. Neither the sequence 0-1-2 nor 0-2-1 represents increasing size as far as we can tell, so we have not ordered this character.

State 0 is present in *Edops* (D. M., pers. obs. of MCZ 6493) and *Saxonerpeton* (CG78: 38).

State 1 is found in *Cochleosaurus* (Sequeira, 2009: fig. 11), the largest specimen of *Trimerorhachis* (Pawley, 2007), *Pholiderpeton attheyi* (Panchen, 1972: fig. 14b) and *Gephyrostegus* (D. M., pers. obs. of MB.Am.641). Following Kennedy (2010: fig. 9D), we have also scored state 1 for *Limnoscelis*; the internal trochanter is in any case present (Kennedy, 2010: 217).

Kotlassia (Bystrow, 1944: fig. 19) and *Seymouria* (D. M., pers. obs. of BEG 30966-176) are probably best scored as showing state 2, which they share with *Microphon* (Bulanov, 2014). State 2 is further seen in Dendrerpetidae (Carroll, 1967: fig. 18), *Acheloma* (Olson, 1941: fig. 12; Polley & Reisz, 2011: fig. 14), *Ecolsonia* (Berman, Reisz & Eberth, 1985: 23, fig. 12D), *Doleserpeton* (Sigurdsen & Bolt, 2010: fig. 11B), *Platyrhinops* (Hook & Baird, 1984: fig. 1), *Archeria* (Romer, 1957), *Batropetes* (Glienke, 2013: fig. 8Q; D. M., pers. obs. of MB.Am.1232; unclear if 1 or 2 from Glienke, 2015), *Diplocaulus* (Williston, 1909; Douthitt, 1917), *Orobates* (Berman et al., 2004: fig. 15; Nyakatura et al., 2015: digital model), *Tseajaia* (Moss, 1972) and **Beiyanerpeton* (Gao & Shubin, 2012), although we caution that in some of these cases it is not certain whether the trochanter was fully ossified. In contrast, an unambiguous case of state 2 occurs in **Lydekkerina* (Pawley & Warren, 2005: fig. 6).

Urocordylus seems to have state 0 or 2 (Bossy, 1976: 228).

It appears from comparisons that the supposedly left femur of **Platyoposaurus* shown by Gubin (1991: drawing 35) is a right one, and that the supposed fourth trochanter (*tr*) is actually the entire adductor blade. Under this assumption, it is not quite clear whether state 1 or 2 is present, but state 2 appears more likely. The text does not mention the internal trochanter.

Incomplete ossification prevents us from determining whether *Neldasaurus* (Chase, 1965), *Proterogyrinus* (Holmes, 1984), *Bruktererpeton* (Boy & Bandel, 1973), *Tuditanus*, *Micraroter*, *Pelodosotis*, *Microbrachis* (CG78: 99, 124, fig. 5I, 50F, 83, 127; D. M., pers. obs. of MB.Am.840.2, the specimen figured in Olori, 2015: fig. 24F) and *Brachydectes* (Wellstead, 1991) had state 1 or 2; we have scored partial uncertainty. *Broiliellus* is apparently not well enough preserved to decide between the same two states (Carroll, 1964). *Amphibamus* may be well enough preserved, but Daly's (1994) fig. 18 is unclear, and no better reference appears to exist, so we have scored the same uncertainty; similarly, we have assigned state 1 or 2 to *Petrolacosaurus* (Reisz, 1981: fig. 22). Being unaware of a sufficient illustration of a femur of *Euryodus*, we have also scored it the same way; the only *Scincosaurus* specimen that appears to preserve a femur, MB.Am.29, is damaged in that area (D. M., pers. obs.), so we have scored it the same way as well.

Wholly inapplicable to the insufficiently ossified femora of *Discosauriscus* (Klembara & Bartík, 2000: fig. 25).

232. FEM 3: **Fourth trochanter of femur with distinct rugose area: no (0); yes (1).** We have not investigated whether this character is size-related or how distinct the area can be.

Phonerpeton shows state 0 (D. M., pers. obs. of MCZ 1771 and MCZ 2474, several small and incompletely ossified femora), as do *Doleserpeton* (Sigurdsen & Bolt, 2010), *Diplocaulus* (Williston, 1909; Douthitt, 1917) and *Microphon* (Bulanov, 2014).

We have scored state 1 for **Nigerpeton* because there is a distinct process; whether it was rugose before erosion is impossible to tell (D. M., pers. obs. of MNN MOR 82).

233. FEM 4: **Proximal end of femur adductor crest reaching midshaft length: no (0); yes (1).**

Diplocaulus has state 0 (Williston, 1909; Douthitt, 1917).

State 1 is found in *Edops* (D. M., pers. obs. of MCZ 7264), *Chenoprosopus* (Hook, 1993), *Cochleosaurus* (Sequeira, 2009), *Trimerorhachis* (Pawley, 2007), *Phonerpeton* (D. M., pers. obs. of MCZ 1771 and MCZ 2474), *Diadectes* (Case, 1911: fig. 30a; Berman, Sumida & Martens, 1998: fig. 18A; D. M., pers. obs. of BEG 31222-56), *Microphon* (Bulanov, 2014) and *Orobates* (Berman et al., 2004).

234. FEM 5: **Femur shorter than (0), as long as (1), or longer than humerus (2) (ordered).** This is a continuous character.

State 0 is found in *Panderichthys* (Boisvert, 2005) and *Ichthyostega* (Coates & Clack, 1995, and references therein; Pierce, Clack & Hutchinson, 2012: fig. 1, supplementary text 1.1, supplementary movies), making the distinction between states 0 and 1 parsimony-informative. In RC07, state 0 was restricted to *Eusthenopteron*.

Acanthostega (Coates, 1996: 389: "The femur is about 25% longer than the humerus"!), *Cochleosaurus* (Sequeira, 2009), *Isodectes* (D. M., pers. obs. of CM 81430 and USNM 4474), *Trimerorhachis* (Pawley, 2007), *Doleserpeton* (Sigurdsen & Bolt, 2010: fig. 1; Sigurdsen & Green, 2011: appendix 2) and *Leptorophus* (Werneburg, 2007a) have state 2.

State 1 makes surprise appearances in *Acheloma* (specimen WM 1756: Olson, 1941: fig. 11D, E, 12B–D) and **Lydekkerina* (Pawley & Warren, 2005: fig. 6).

Batropetes was scored as having states 1 and 2 in RC07. State 2 is limited to the left side of one specimen (Glienke, 2015: appendix 1). On the right side, the same specimen has state 1; state 1 is further found in one or two other specimens of *B. palatinus* and maybe one of *B. fritschi*. All others have state 0 (Glienke, 2013: appendix; 2015: appendix 1). Because state 0 is so common, we have assigned it to *Batropetes* without polymorphism.

235. TIB 6: **Outline of tibia medial margin shaped like a distinct, subsemicircular embayment contributing to interepipodial space and the diameter of which is less than one-third of bone length: absent (0); present (1).**

Since almost any condition is by definition state 0, it is not surprising that state 0 is known to occur in *Panderichthys* (Boisvert, 2005), *Edops* (D. M., pers. obs. of MCZ 7162), *Cochleosaurus* (Sequeira, 2009: fig. 11), *Isodectes* (D. M., pers. obs. of USNM 4474), *Doleserpeton* (Sigurdsen & Bolt, 2010; D. M., pers. obs. of AMNH 24969), *Platyrhinops* (Clack & Milner, 2010), Albanerpetidae (McGowan, 2002) and *Karaurus* (Ivachnenko, 1978; D. M. and M. L., pers. obs. of unnumbered MNHN cast of PIN 2585/2).

Strangely, Warren (2007: fig. 11) did not color the tibia of *Ossinodus* in her skeletal reconstruction to mark it as known. The shape of the reconstructed tibia, however, agrees with the better preserved one of the two described by Warren & Turner (2004), so we assume the lack of color is unintentional and have kept the scores for this and the following character.

236. TIB 7: **Tibia without (0) or with (1) flange along its posterior edge.**

Like *Panderichthys* (Boisvert, 2005), *Cochleosaurus* (Sequeira, 2009: fig. 11), *Isodectes* (D. M., pers. obs. of USNM 4474), *Doleserpeton* (Sigurdsen & Bolt, 2010; D. M.,

pers. obs. of AMNH 24969) and *Karaurus* (D. M., pers. obs. of unnumbered MNHN cast of PIN 2585/2), *Orobates* has state 0 (Nyakatura et al., 2015: digital model).

We count the crest of **Archegosaurus* (Witzmann & Schoch, 2006b: fig. 9B), and by extension *Edops* (D. M., pers. obs. of MCZ 7162 and MCZ 7259), as state 1.

237. FIB 1: **Fibula waisted: no (0); yes (1).**

State 0 is found in *Panderichthys* (Boisvert, 2005).

Edops possesses state 1 (D. M., pers. obs. of MCZ 1782 and 7258), and so do *Cochleosaurus* (Sequeira, 2009: fig. 11), *Isodectes* (D. M., pers. obs. of CM 81430), *Doleserpeton* (Sigurdsen & Bolt, 2010; D. M., pers. obs. of AMNH 29470), *Platyrhinops* (Hook & Baird, 1984; Clack & Milner, 2010) and *Leptorophus* (Werneburg, 2007a). We have also assigned state 1 to *Notobatrachus* because its tibiofibula is strongly waisted (Báez & Nicoli, 2004).

The fibula is entirely unknown in *Ossinodus* (Warren & Turner, 2004; Warren, 2007: fig. 11).

All of the bones lying around in fig. 10A of Milner & Sequeira (2011) are waisted; judging from their sizes and positions, one of them is bound to be the fibula. We have therefore scored state 1 for **Erpetosaurus*.

238. FIB 3: **Ridge near posterior edge of fibula flexor surface: absent (0); present (1).**

Orobates has state 0 (Berman et al., 2004; Nyakatura et al., 2015: digital model).

Trimerorhachis (Pawley, 2007: fig. 15.2.3; D. M., pers. obs. of TMM 40998-39) and *Doleserpeton* (Sigurdsen & Bolt, 2010) show state 1. (The *Doleserpeton* specimen AMNH 29470 has state 0 [D. M., pers. obs.], but this could be ontogenetic.) State 1 further shows up in **Australerpeton* (Eltink & Langer, 2014).

Sigurdsen & Green (2011: appendix 2) recommended to score *Valdotriton* as unknown; we have followed this.

The fibula is entirely unknown in *Ossinodus* (Warren & Turner, 2004; Warren, 2007: fig. 11).

239. FIB 4: **Rows of tubercles near posterior edge of fibula flexor surface: absent (0); present (1).**

Doleserpeton (Sigurdsen & Bolt, 2010) and *Orobates* (Berman et al., 2004; Nyakatura et al., 2015: digital model) have state 0.

State 1 is found in *Eryops* (Pawley & Warren, 2006).

The fibula is entirely unknown in *Ossinodus* (Warren & Turner, 2004; Warren, 2007: fig. 11).

240. TAR 2: **Separate tibiale, intermedium and/or centrale 4 (0); astragalus (1).** The original wording counted the "[p]roximal tarsal ossifications: absent (0); presence of single ossification (1); presence of more than two ossifications (2)". This did not distinguish incomplete ossification of the tarsus from fusion of individual tarsals. The degree of ossification depends both on ontogeny and on lifestyle (with aquatic taxa ossifying the proximal tarsus later and/or to a lesser degree than terrestrial ones). Furthermore, incomplete ossification and incomplete preservation can only be distinguished in articulated skeletons. Finally, the astragalus usually comes with a calcaneum (= fibulare), so there are two "[p]roximal tarsal ossifications" – yet no state was available between "single" and "more than two"! Of the OTUs with an astragalus, *Captorhinus*, *Paleothyris* and *Petrolacosaurus* were scored as having state 2 in RC07, while *Diadectes*, *Tuditanus* and *Pantylus* were scored as having state 1 or 2! We do not consider this tenable and have redefined the character to consider fusion only.

Of the OTUs previously scored as unknown or partially uncertain, Albanerpetidae has state 0 (McGowan, 2002), as do *Eocaecilia* (Jenkins, Walsh & Carroll, 2007), *Triadobatrachus* (Roček & Rage, 2000; Ascarrunz et al., 2016), *Kotlassia* (Bystrow, 1944: 406), most likely *Limnoscelis* (Kennedy, 2010), *Batropetes* (Glienke, 2013), *Microbrachis* (CG78: 124; Olori, 2015) and *Scincosaurus* (Milner & Ruta, 2009). Apparently, so does *Platyrhinops* (Hook & Baird, 1984; Clack & Milner, 2010: fig. 1a). We have also scored state 0 for *Ossinodus*, where Warren (2007: fig. 11) figured a tibiale in a skeletal restoration without mentioning it in the text (the possible fibulare of Warren & Turner [2004], which has a quite different shape, is not shown), and **Pholidogaster*, where the largest preserved tarsal (on both sides), which is clearly proximal, is much smaller than expected for an astragalus (Romer, 1964: fig. 1B).

We count *Gephyrostegus* as possessing state 1: the tibiale and the intermedium are (although incompletely) fused, and the lateralmost centrale in fig. 9 of Carroll (1970) should be the centrale 3, not 4.

In *Euryodus* the condition is unknown (CG78: 65).

241. TAR 3: **L-shaped proximal tarsal element: absent (0), present (1).**

Albanerpetidae and *Eocaecilia* have state 0 (McGowan, 2002; Jenkins, Walsh & Carroll, 2007), as do *Limnoscelis* (Kennedy, 2010), *Microbrachis* (Olori, 2015) and *Tseajaia*, assuming that Moss (1972) has interpreted the tarsus correctly (the shapes of the tibiale and the intermedium are rather unusual).

Tuditanus shows state 1 (Carroll & Baird, 1968: fig. 10B).

242. TAR 4: **Distal tarsal ossifications between fibulare and digits: absent (0); present (1).** RC07 (p. 108) stated that this character and TAR 5 have the same distribution. We would have merged the characters in that case; however, *Scincosaurus* and *Orobates* break the pattern (see below).

Platyrhinops (D. M., pers. obs. of AMNH 2002) and Albanerpetidae (McGowan, 2002) have state 1.

Remarkably, *Scincosaurus* shows state 0 (Milner & Ruta, 2009).

243. TAR 5: **Distal tarsal ossifications between tibiale and digits: absent (0); present (1).**

State 1 is known in *Platyrhinops* (D. M., pers. obs. of AMNH 2002), Albanerpetidae (McGowan, 2002), *Batropetes* (Glienke, 2015) and *Scincosaurus* (Milner & Ruta, 2009).

Orobates has state 0; of all distal tarsals only the fourth is ossified (Berman et al., 2004).

244. RIB 1: **Anterior and posterior process of ribs: both absent (0); anterior process present, posterior process absent (1); both present, ribs k-shaped in at least part of the trunk (2) (ordered).** RC07 compared the shape to a capital K, but the ventrolateral ramus is distinctly longer than the others, so we follow the comparison by Anderson (2002, 2003a, b) and Anderson, Carroll & Rowe (2003). While RC07 only contrasted state 2 with the others in a binary character, we prefer to code the fact that *Lethiscus* has half of the k-shape (Anderson, Carroll & Rowe, 2003). State 1 is not known elsewhere (the anterior process may be homologous with the capitulum, but differs in not articulating with a vertebra); ribs with a posterior but no anterior process appear to be entirely unknown, justifying ordering of this character.

Cochleosaurus (Sequeira, 2009), *Isodectes* (D. M., pers. obs. of USNM 4555, CM 81430 and CM 81512), *Archeria* (D. M., pers. obs. of MCZ 2049 and others) and *Cardiocephalus* (CG78: fig. 31) have state 0.

245. RIB 2: **"Cervical" ("pectoral") ribs with (0) or without (1) flattened distal ends.**

Unknown in *Ossinodus* (Warren & Turner, 2004; Warren, 2007).

Cochleosaurus has state 0 (Sequeira, 2009), and so do *Isodectes* (D. M., pers. obs. of USNM 4474 and CM 81430), *Doleserpeton* (Sigurdsen & Bolt, 2010), *Cardiocephalus* (CG78: fig. 31) and *Ptyonius* (D. M., pers. obs. of MCZ 3721, a cast of "AMNH 6871 (85466)").

Trimerorhachis has state 1 where known (Milner & Schoch, 2013). So do *Oestocephalus* (Anderson, 2003a: fig. 4B) and *Phlegethontia* (Anderson, 2002). We further follow Pardo et al. (2017: matrix) in scoring state 1 for *Lethiscus* and **Coloraderpeton*.

246. RIB 3: **Ribs mostly straight (0) or ventrally curved in at least part of the trunk (1).**

The "first dorsal rib" of *Tseajaia* has "cervical rib" morphology (Moss, 1972); this may be why it (and it alone) was scored as polymorphic by RC07. The definition refers to "at least part of the trunk", however, meaning that *Tseajaia* has state 1.

So do *Isodectes* (D. M., pers. obs. of USNM 4474, CM 81430 and CM 81512) and *Cardiocephalus* (CG78).

State 0 is found in *Cochleosaurus* (Sequeira, 2009). We follow Pardo et al. (2017: matrix) in scoring state 0 for **Coloraderpeton*.

Schoch & Rubidge (2005: figs. 5B, 7A) showed curvature in **Micropholis*, but did not explain if it is ventral or only caudal in direction. We have scored **Micropholis* as unknown.

247. RIB 4: **Broad rectangular flanges in at least some trunk ribs: absent (0); present (1).**

Like *Cochleosaurus* (Sequeira, 2009), *Archeria* (D. M., pers. obs. of MCZ 2049 and others), *Kotlassia* (Bystrow, 1944) and *Cardiocephalus* (CG78: fig. 31), *Lethiscus* has state 0 (Anderson, Carroll & Rowe, 2003).

**Nigerpeton* has state 1 (D. M., pers. obs. of MNN MOR 83). In **Platyoposaurus*, the flanges are somewhat narrow, but clearly present as well (Konzhukova, 1955; Gubin, 1991).

248. RIB 5: **Triangular spur-like posterodorsal process in at least some trunk ribs: absent (0); present (1).**

Baphetes shows state 1 (Milner & Lindsay, 1998), as do *Gephyrostegus* (Godfrey & Reisz, 1991) and *Kotlassia* (Bystrow, 1944).

Like *Cochleosaurus* (Sequeira, 2009), *Archeria* (D. M., pers. obs. of MCZ 2049 and others) and *Cardiocephalus* (CG78: fig. 31), *Lethiscus* has state 0 (Anderson, Carroll & Rowe, 2003), and so does **Nigerpeton* (D. M., pers. obs. of MNN MOR 83).

249. RIB 6: **Elongate posterodorsal triangular flange in the midtrunk ribs: absent (0); present (1).**

Like *Cochleosaurus* (Sequeira, 2009), *Archeria* (D. M., pers. obs. of MCZ 2049 and others) and *Cardiocephalus* (CG78: fig. 31), *Lethiscus* has state 0 (Anderson, Carroll & Rowe, 2003), and so does **Nigerpeton* (D. M., pers. obs. of MNN MOR 83).

250. RIB 7: **Trunk ribs longer (0) or shorter (1) than three successive articulated vertebrae in adults.** The measured vertebrae should be from the same region of the trunk as the vertebrae.

RC07 added the unquantified terms "poorly ossified" and "slender" to the definition of state 1 and did not test if all three traits are correlated. We have reduced the character to length alone, making it identical to McGowan's (2002) ch. 1 as modified by Marjanović & Laurin (2008: 177f.). Thus, we have scored *Micromelerpeton* as possessing state 0 which,

according to R. Schoch (pers. comm. to Marjanović & Laurin, 2008: 178), is observed in the metamorphosed specimens announced by Lillich & Schoch (2007) and Schoch (2009b), and we have scored *Balanerpeton* (Milner & Sequeira, 1994), Dendrerpetidae (Holmes, Carroll & Reisz, 1998), *Acheloma* (Case, 1911: fig. 46), *Cardiocephalus* (borderline; CG78: fig. 31), *Odonterpeton* (CG78), *Lethiscus* (the longest preserved rib seems not to reach state 0, although it comes close: Wellstead, 1982: fig. 8B), *Oestocephalus* (Carroll, 1998a; Anderson, 2003a) and *Phlegethontia* (both species; Anderson, 2002: fig. 10) as having state 1.

Importantly, *Acheloma* (Case, 1911: fig. 46) demonstrates that this character is not correlated to absolute body size, even though **Utaherpeton* adds to the already known sample of taxa which change from state 1 to (barely) state 0 in their ontogeny (Carroll, Bybee & Tidwell, 1991; Carroll & Chorn, 1995).

State 0 is further found in *Baphetes* (Milner & Lindsay, 1998), *Cochleosaurus* (Sequeira, 2009) and *Isodectes* (D. M., pers. obs. of CM 81512) as well as **Nigerpeton* (D. M., pers. obs. of MNN MOR 83). It furthermore occurs (just barely) in *Adelospondylus* and *Adelogyrinus* (Andrews & Carroll, 1991; contra Marjanović & Laurin, 2009), while *Acherontiscus*, in which the ribs are as long as 2½ vertebrae, has state 1 (Carroll, 1969a).

Unknown in *Phonerpeton* (Dilkes, 1990) and in *Westlothiana* where it is too borderline to tell (Smithson et al., 1994).

251. CER VER 1: **Halves of atlas neural arch unfused (0) or fused (1).**

Edops has state 0 (D. M., pers. obs. of MCZ 7197), as do *Cochleosaurus* (Sequeira, 2009), *Trimerorhachis* (Pawley, 2007; Milner & Schoch, 2013), *Acheloma* (Polley & Reisz, 2011), *Triadobatrachus* (Ascarrunz et al., 2016) and *Batropetes* (Glienke, 2013: 81; 2015).

Euryodus is polymorphic, with *E. dalyae* having state 0 as scored, but *E. primus* showing state 1 (CG78: fig. 115).

State 1 is found in *Diplocaulus* (Williston, 1909: pl. 3; Douthitt, 1917: fig. 4). We have scored **Gerobatrachus* as possessing state 1, following the matrix by Anderson et al. (2008a), surprising though this is (*Doleserpeton* has state 0, as was correctly scored: Bolt, 1991: fig. 5; Sigurdsen & Bolt, 2010).

The situation in *Microbrachis* is unclear; in the other vertebrae the halves seem to have fused in later ontogeny (Olori, 2015). We have scored it as unknown.

We follow Pardo et al. (2017: matrix) in scoring state 1 for *Lethiscus* and **Coloraderpeton*.

252. CER VER 3: **Axis arch not fused (0) or fused (1) to axis (pleuro)centrum.**

Trimerorhachis (Pawley, 2007) shows state 0, as does *Acheloma* (Polley & Reisz, 2011).

Eocaecilia has state 1 (Jenkins, Walsh & Carroll, 2007), as do *Triadobatrachus* (Ascarrunz et al., 2016: fig. 7), *Kotlassia* (Bystrow, 1944: 394, fig. 8), apparently *Batropetes* (Glienke, 2013) and *Cardiocephalus* (there is only “a trace of suture”: CG78: 58). We have also scored it for *Hyloplesion*, following Olori (2015: table S3), coherent with a statement on p. 57 of that paper but apparently contradicting another on p. 46.

We also ascribe state 1 to Albanerpetidae because we strongly suspect that the entirely arch-less “axis”, which often fuses to the “third cervical”, is in fact the axis intercentrum, and the “third cervical” is the axis pleurocentrum + neural arch (Material and methods: Modifications to individual cells: The albanerpetid neck).

In *Brachydectes*, however, we have scored state 0: while we have not been able to find a statement concerning specifically the axis, neurocentral sutures are ubiquitous in both the atlas and other vertebrae (Wellstead, 1991; Pardo & Anderson, 2016: fig. 15), so state 1 would be rather surprising.

State 0 is documented in subadult specimens of *Doleserpeton*, but the condition in adult individuals is apparently unknown; given state TRU VER 11(1), it is possible that they had state 1 of this character (Sigurdsen & Bolt, 2010), so we follow Sigurdsen & Green (2011) in keeping *Doleserpeton* scored as unknown.

State 0 is also seen in the only known axis of *Microphon* (Bulanov, 2014); given the immature or paedomorphic (Bulanov, 2003) status of that specimen, we retain the original score of unknown.

Given that the arch is fused to the centrum both in the atlas and in the trunk (Pardo et al., 2017: ch. 111 and 132 in their matrix), state 1 seems likely enough in *Lethiscus* and **Coloraderpeton* for us to score it.

253. CER VER 4: **Odontoid process, or tuberculum interglenoideum, on anterior surface of atlas body: absent (0); present (1).** It is a good question if this process – also called "intercotylar tubercle"; not homologous to the odontoid process of mammals, which consists of the entire atlas pleurocentra that are fused to the axis – should be considered homologous regardless of whether the "atlas body" consists of pleuro- or intercentra. Unfortunately, whether the atlantes of, say, lissamphibians consist of pleuro- or intercentra is itself a difficult question, so we have followed RC07 in considering all such processes primarily homologous.

We have scored this character as unknown for OTUs which are known to have any state of EXOCC 2-3-4-5/BASOCC 1-5 (ch. 134) other than 4 or 5, because the process does not (as far as known) and probably cannot occur together with states 0, 1 or 2 of EXOCC 2-3-4-5/BASOCC 1-5-6 and by definition occurs with state 3 (where the cotyle of the basioccipital articulates with it). The great exception to this rule is *Phlegethontia*, which has state 1 of that character, but nonetheless displays a long odontoid process; this is made possible by the surprising gap between the skull and the atlas that is bridged by the long proatlas dorsally and, at least in part, by the odontoid process ventrally (Anderson, 2002; D. M., pers. obs. of USNM 17097, where the proatlas is not preserved). Although the process is an extension of the ventral surface in *Phlegethontia*, it has a more dorsal, more lissamphibian-like location in the closely related **Pseudophlegethontia* (Anderson, 2003b), so we see no reason not to consider it primarily homologous and have scored state 1.

Furthermore, this character is inapplicable to *Doleserpeton*, in which the area where a tubercle could lie is occupied by the huge notochordal canal (Bolt, 1991: fig. 5; Sigurdsen & Bolt, 2010). Similarly, the presacral centra of **Sclerocephalus* are very incompletely ossified (Schoch & Witzmann, 2009a); **Platyoposaurus* (Gubin, 1991: drawing 27) shows a well-defined but huge notochordal notch similar to that of *Doleserpeton* despite being a much larger animal.

The illustrations of *Acheloma* supplied by Polley & Reisz (2011) are, unfortunately, not three-dimensional enough to tell; however, because Polley & Reisz (2011) did not mention the presence of a process, we have scored *Acheloma* as possessing state 0.

We cannot find a description or illustration of the atlantes of *Amphibamus* or *Eoscopus*; the most likely source, Daly (1994), did not describe any atlas centra, except for mentioning the very existence of one in *Platyrhinops* (which RC07 already scored as unknown). We have therefore scored both as unknown.

The condition is further unknown in *Hyloplesion* (CG78: 131).

Odonterpeton was scored as unknown in RC07. CG78: 167 implied state 0, but this refers to a large process as commonly found in "microsaurs"; the process can be very small in lissamphibians, and indeed CG78: fig. 99C (repeated as fig. 116L) depicted state 1. Personal observation by D. M. of USNM 4465+4467 (the holotype and probably only known specimen) shows that this is correct – although only as an outline drawing; the process is not

part of the ventral surface of the atlas, but has a considerably more dorsal position, making the atlas much less unusual than implied by CG78.

State 1 is found in *Diplocaulus* (Williston, 1909: pl. 3), as well as in *Triadobatrachus* (very weakly ossified: Ascarrunz et al., 2016: 3D model 1), *Notobatrachus* (Báez & Basso, 1996; Báez & Nicoli, 2004), **Liaobatrachus* (figures in Dong et al., 2013) and other frogs like ***Gobiates* (Roček, 2000: fig. 16; 2008: fig. 2E). We further follow Pardo et al. (2017: matrix) in scoring it for *Lethiscus* and **Coloraderpeton*.

254. TRU VER 1: **Extra articulations above zygapophyses in at least some trunk and caudal vertebrae: absent (0); present (1).**

Cochleosaurus (Sequeira, 2009), *Isodectes* (D. M., pers. obs. of CM 81512) and *Cardiocephalus* (CG78: fig. 31) have state 0.

255. TRU VER 2: **Neural and haemal spines rectangular to fan-shaped in lateral view: no (0); yes (1).**

Chenoprosopus shows state 0 (D. M., pers. obs. of USNM 437646), as do *Cardiocephalus* (CG78: fig. 31), *Phlegethontia* (Anderson, 2002), *Ossinodus* (Warren, 2007) and **Nigerpeton* (D. M., pers. obs. of MNN MOR 83).

Pardo et al. (2017: supp. inf.) scored **Coloraderpeton* as having state 0 of their ch. 115: "Neural spine shape in lateral view (HPSA 153): (0) anterior and posterior sides parallel, forming a rectangular surface; (1) non-parallel, triangular". We interpret this as our state 1.

256. TRU VER 3: **Neural and haemal spines aligned dorsoventrally: absent (0); present (1).**

Hyloplesion (Olori, 2015) and *Ossinodus* (Warren, 2007) have state 0.

The entire tail is unknown in *Colosteus* (Hook, 1983), *Crassigyrinus* (Panchen, 1985), *Neldasaurus* (Chase, 1965), *Broiliellus* (Carroll, 1964), *Eoherpeton* (Smithson, 1985), *Pholid-erpeton scutigerum* (Clack, 1987b), *Gephyrostegus* (Carroll, 1970; Godfrey & Reisz, 1991), *Solenodonsaurus* (Laurin & Reisz, 1999; Danto, Witzmann & Müller, 2012), *Stegotretus* (Berman, Eberth & Brinkman, 1988), and *Ariekanerpeton* (Klembara & Ruta, 2005b). Similarly, no hemal arches are preserved in *Triadobatrachus* (Roček & Rage, 2000), and none were ossified in described specimens of *Apateon* or *Leptorophus*.

Doleserpeton has state 0 as scored, but one wonders how this was done before Sigurdsen & Bolt (2010) described (any part of) the tail for the first time in a publication; Ruta, Coates & Quicke (2003) did not claim to have seen specimens and did not cite Bolt's unpublished thesis (which dates to 1964).

257. TRU VER 4: **Haemal spines not fused (0) or fused (1) to caudal centra.** According to RC07, state 1 is "observed almost exclusively in nectrideans", but this statement does not seem defensible to us.

Importantly, hemal arches are not homologous to intercentra or parts thereof (contra, e.g., Williston, 1912: 466; Carroll, 1968: 1177, 1188–1189, 1969a: 545; Carroll & Chorn, 1995: 49; Palci, Caldwell & Nydam, 2013: 1339; Olori, 2015: 57). This is demonstrated by the separate hemal arches and intercentra of animals like the temnospondyls **Acanthostoma-tops* (Witzmann & Schoch, 2006a), **Lydekkerina* (Pawley & Warren, 2005) and ***Trematolestes* (Schoch, 2006: fig. 6H) or the anthracosaurs **RM 206859 (Holmes & Carroll, 2010) and apparently **CM 34638 (Clack, 2011a). Therefore, this character cannot be interpreted as "hemal spines not/fused to caudal pleurocentra". It is also, unfortunately, not a cheap way of determining whether an animal has intercentra (see TRU VER 7, 8, 9, 13-14).

State 1 is thus present at a minimum in *Eusthenopteron* and *Acanthostega* (Coates, 1996), probably *Ichthyostega* (Jarvik, 1996: pl. 38), *Greererpeton* (Godfrey, 1989), *Edops* (D. M., pers. obs. of MCZ 7128), *Trimerorhachis* (Pawley, 2007), Dendrerpetidae (Holmes, Carroll & Reisz, 1998: fig. 1), *Eryops* (Moulton, 1974: fig. 6–8), *Acheloma* (Case, 1911: 135), *Phonerpeton* (Dilkes, 1990: fig. 10), *Ecolsonia* (Berman, Reisz & Eberth, 1985), *Amphibamus* and *Eoscopus* (Daly, 1994), *Platyrhinops* (Hook & Baird, 1984), *Karaurus* (Ivachnenko, 1978: 366; D. M., pers. obs. of unnumbered MNHN cast of PIN 2585/2), *Caerorhachis* (Ruta, Milner & Coates, 2002), *Proterogyrinus* (Holmes, 1984), *Archeria* (Holmes, 1989), *Pholiderpeton attheyi* (Panchen, 1972), *Bruktererpeton* (Boy & Bandel, 1973: fig. 8), *Kotlassia* (Bystrow, 1944), *Discosauriscus* (Klembara & Bartík, 2000), *Seymouria* (White, 1939: 356), *Diadectes* (Berman, Sumida & Martens, 1998: 78), *Limnoscelis* (Williston, 1912: 466, fig. 25; Berman & Sumida, 1990: 326), *Captorhinus* (Dilkes & Reisz, 1986: 1294), *Petrolacosaurus* (Reisz, 1981: 36), *Westlothiana* (Smithson et al., 1994), *Micraroter* (CG78: 97, fig. 58), *Orobates* (Nyakatura et al., 2015: digital model), *Ossinodus* (Warren, 2007), *Silvanerpeton* (Ruta & Clack, 2006) and *Utegenia* (Klembara & Bartík, 2000: fig. 30). It is also suggested for *Balanerpeton* by fig. 10C of Milner & Sequeira (1994); we have accepted this at face value.

Hyloplesion has state 0 (Olori, 2015).

A large number of taxa where the tail is poorly or not known were scored as having state 0 by RC07. This includes *Colosteus* (Hook, 1983), *Crassigyrinus* (Panchen, 1985), *Neldasaurus* (Chase, 1965), *Broiliellus* (Carroll, 1964), *Eoherpeton* (Smithson, 1985), *Pholiderpeton scutigerum* (Clack, 1987b), *Gephyrostegus* (Carroll, 1970; Godfrey & Reisz, 1991), *Solenodonsaurus* (Laurin & Reisz, 1999; Danto, Witzmann & Müller, 2012), *Stegotretus* (Berman, Eberth & Brinkman, 1988) and *Ariekanerpeton* (Klembara & Ruta, 2005b).

Further unknown in *Baphetes* (Milner & Lindsay, 1998: fig. 8), Albanerpetidae (McGowan, 2002) and *Paleothyris* (Carroll, 1969b); inapplicable to *Triadobatrachus* which does not preserve any hemal arches (Roček & Rage, 2000; Ascarrunz et al., 2016).

Doleserpeton has a truly remarkable tail in which all elements of each vertebra – neural arch, pleurocentrum, intercentrum, and hemal arch – are fused into a single bone (Sigurdsen & Bolt, 2010). This constitutes state 1; it is probably not even possible to distinguish if the hemal arches are fused to the intercentra alone or to both inter- and pleurocentra, though Sigurdsen & Bolt (2010) suggested that the latter has happened.

We interpret **Archaeovenator* (Reisz & Dilkes, 2003: fig. 1) as having fused hemal arches and intercentra, thus state 1.

258. TRU VER 5: **Extra articulations on haemal spines: absent (0); present (1).**

State 0 is now known in *Hyloplesion* (Olori, 2015), *Orobates* (Nyakatura et al., 2015: digital model) and *Ossinodus* (Warren, 2007).

State 1 is found in *Scincosaurus* (Milner & Ruta, 2009).

The entire tail is unknown in *Colosteus* (Hook, 1983), *Crassigyrinus* (Panchen, 1985), *Neldasaurus* (Chase, 1965), *Broiliellus* (Carroll, 1964), *Eoherpeton* (Smithson, 1985), *Pholiderpeton scutigerum* (Clack, 1987b), *Gephyrostegus* (Carroll, 1970; Godfrey & Reisz, 1991), *Solenodonsaurus* (Laurin & Reisz, 1999), *Stegotretus* (Berman, Eberth & Brinkman, 1988) and *Ariekanerpeton* (Klembara & Ruta, 2005b). Similarly, no hemal arches are preserved in *Triadobatrachus* (Roček & Rage, 2000; Ascarrunz et al., 2016).

Doleserpeton has state 0 as scored, but one wonders how this was done before Sigurdsen & Bolt (2010) described (any part of) the tail for the first time in a publication; Ruta, Coates & Quicke (2003) did not claim to have seen specimens and did not cite Bolt's unpublished thesis (which dates to 1964).

259. TRU VER 7: **Ossified pleurocentra: absent (0); present (1).** Under the assumption that loss of ossification does not equal loss of the element, we have not treated this character or TRU VER 13-14 (see below) as irreversible in Analyses R7–R12.

Like RC07, we have scored this and the next two characters as unknown in all taxa where the vertebrae are monospondylous, because in them there is no way – other than phylogenetic reconstruction – to tell whether the single centrum is the inter- or the pleurocentrum (i.e. the fused pair of left and right inter- or pleurocentra); see TRU VER 4 (above) on the homology of hemal arches. We have correspondingly scored TRU VER 13-14 as state 0 (intercentrum forms complete ring) or 2 (trunk intercentra absent), an option that was not available before we merged those two characters. The only cases RC07 overlooked are *Batropetes* (Carroll, 1991; Glienke, 2013, 2015), *Microbrachis* (CG78; Olori, 2015) and *Scincosaurus* (Bossy & Milner 1998; Milner & Ruta 2009) and, in the case of TRU VER 13 but not TRU VER 7, 8 and 9, *Triadobatrachus* (Ascarrunz et al., 2016). The only exceptions, for which we have scored state 1, are Albanerpetidae (already so scored by RC07 for unclear reasons), which may have axis intercentra (Material and methods: Modifications to individual cells: The albanerpetid neck), and **Utaherpeton*: the tail of the immature specimen of **Utaherpeton* of demonstrates that the only ossified centra are pleurocentra because the last few are broad dorsally but narrow to a point ventrally (Carroll & Chorn, 1995) – the opposite would be expected for intercentra.

There is evidence from **extant frogs that their centra are pleurocentra: the tadpoles of certain megophryids ossify caudal centra that begin as paired ossifications dorsolateral to the notochord, with the distalmost centra not progressing beyond this stage before they are osteoclastically destroyed during metamorphosis (Handrigan & Wassersug, 2007). However, there are no megophryids, indeed no clear crown-group frogs at all, in this matrix; we have kept *Notobatrachus*, *Vieraella* and **Liaobatrachus* as unknown.

In the adelogyrinids, it is likewise difficult or impossible to determine the homology of the monospondylous, fully ossified centra; the fact that the neural arches are positioned dorsal to the cranial halves of the centra and even articulate with two successive centra in part of the trunk of ***Palaeomolgophis* (Andrews & Carroll, 1991: 243) rather strongly suggests that the centra are intercentra and all adelogyrinids are fully stereospondylous, but in order to avoid potential bias against the traditional hypothesis that the adelogyrinids are “lepospondyls”, we have scored them as unknown like the abovementioned other taxa with monospondylous centra.

State 1 is found in *Cochleosaurus* (Sequeira, 2009), *Cardiocephalus* (CG78: fig. 31) and *Utegenia* (Klembara & Ruta, 2004b). Pierce et al. (2013) have confirmed it in *Ichthyostega* (contra Ahlberg, Clack & Blom, 2005; though note that Jarvik, 1996, was right for the wrong reasons).

Clear occurrences of state 0 are thus limited to *Panderichthys* and *Crassigyrinus*, and certainly not homologous between the two. State 0 in *Crassigyrinus*, at least, may be correlated to its generally very low degree of ossification in the endochondral skeleton; scoring *Crassigyrinus* as unknown would render this character parsimony-uninformative.

260. TRU VER 8: **Trunk pleurocentra fused midventrally: no (0); yes (1).**

Cochleosaurus apparently has state 0 (Sequeira, 2009).

Cardiocephalus (CG78: fig. 31) and *Orobates* (Berman et al., 2004: fig. 11; Nyakatura et al., 2015: digital model) have state 1.

The vertebrae of **Casineria* are preserved in strict lateral view. We have scored state 1 because the pleurocentra reach the ventral side of the vertebral column and have a flat surface rather than a point there (Fig. 5). The following character remains unknown for **Casineria*.

261. TRU VER 9: **Trunk pleurocentra fused middorsally: no (0); yes (1).**

Cochleosaurus apparently has state 0 (Sequeira, 2009), as does *Trimerorhachis* (Pawley, 2007).

Cardiocephalus (CG78: fig. 31) and *Orobates* (Berman et al., 2004: fig. 10B; Nyakatura et al., 2015: digital model) have state 1.

In *Proterogyrinus*, the pleurocentra "are tightly appressed dorsally" (Sigurdsen & Green, 2011: 18), but not fused or apparently even sutured; we have therefore kept state 0.

State 0 is observed in the tail and possibly the last presacral vertebra of the immature specimen of **Utaherpeton*, but the condition is unknown in the other vertebrae (which should be more advanced ontogenetically) of that specimen and entirely unknown in the adult specimen (Carroll & Chorn, 1995). We have therefore scored **Utaherpeton* as unknown for this character.

Ariekanerpeton and *Utegenia* were scored as polymorphic. Given the ontogenetic progression from 0 to 1 in better-known seymouriamorphs, we have scored *Ariekanerpeton* as unknown following Klembara & Ruta (2005b: 80) and have kept only state 1 for *Utegenia*, for which state 1 was mentioned to exist by Klembara & Ruta (2004b: 79), but not illustrated in their fig. 3C which is cited there.

262. TRU VER 10: **Neural arches without (0) or with (1) distinct convex lateral surfaces.**

Chenoprosopus (D. M., pers. obs. of USNM 437646), *Cochleosaurus* (Sequeira, 2009) and *Isodectes* (D. M., pers. obs. of CM 81512) have state 0; apparently, so do *Batropetes* (Glienke, 2013) and *Cardiocephalus* (CG78: fig. 31, 33).

Petrolacosaurus was scored as polymorphic in RC07, presumably because "[t]he neural arches are slightly swollen above the postzygapophyses, but only in the dorsal vertebrae" (Reisz, 1981: 34). We think, however, that this character should be considered inapplicable to the greatly elongate cervical vertebrae and have therefore scored state 1 alone. Similarly, we have kept state 1 for *Limnoscelis* and *Orobates*, which have swollen neural arches in the trunk but not the tail vertebrae (Kennedy, 2010; Nyakatura et al., 2015: digital model). – In principle, presence of different states in different parts of the vertebral column should be considered an intermediate state; polymorphism implies that different individuals or indeed subclades have different states, or that the left and right sides of the same individual do.

State 1 makes a surprise appearance in *Diplocaulus* (Williston, 1909: pl. 3).

We have scored *Utegenia* as unknown because the observed state 0 is also found in larvae but not "postmetamorphic" individuals of *Discosauriscus*, in which the appearance of state 1 is interpreted as part of the transition to terrestrial life (Klembara & Bartík, 2000; Klembara, 2009).

263. TRU VER 11: **Neural arches of trunk vertebrae fused to centra: no (0); yes (1).** According to RC07, this character "does not appear to be simply related to […] inferred degree of specimen maturity", yet there is evidence that *Batropetes* and *Microbrachis* (see below) changed from state 0 to state 1 in ontogeny, and in amniotes this is a very widely used marker for skeletal maturity (Irmis, 2007); among OTUs with more or less holospondylous vertebrae, it seems to us that only the most paedomorphic ones keep state 0 throughout life, and the same may hold for seymouriamorphs (Laurin, 2000).

Centra are altogether unknown in *Leptorophus* (Boy, 1987; Werneburg, 2007a); they probably only ossified during metamorphosis (if metamorphosis ever occurred in *Leptorophus*). We have accordingly scored it as unknown.

State 0 is found in *Cochleosaurus* (Sequeira, 2009), *Platyrhinops* (Hook & Baird, 1984), *Saxonerpeton*, *Hapsidopareion* and *Micraroter* (CG78) and *Hyloplesion* (Olori, 2015: 46, apparently contradicting 57).

State 1 is documented in Albanerpetidae (Estes & Hoffstetter, 1976; Fox & Naylor, 1982; McGowan, 1996), *Eocaecilia* (Jenkins, Walsh & Carroll, 2007), *Cardiocephalus* (there is only "a trace of suture"; CG78: p. 60) and *Batrachiderpeton* (Bossy & Milner, 1998: fig. 63A–D).

Discosauriscus, *Ariekanerpeton* and *Utegenia* were scored as having state 0. This is probably ontogenetic (Laurin, 2000); we have changed their scores to unknown.

Following the skeletally most mature specimens (Carroll, 1991; Glienke, 2013; Olori, 2015), we have scored *Batropetes* and *Microbrachis* as possessing state 1.

264. TRU VER 12: **Bicipital rib-bearers on trunk centra: absent (0); present (1).**

Chenoprosopus (Hook, 1993; D. M., pers. obs. of USNM 437646), *Cochleosaurus* (Sequeira, 2009), *Isodectes* (D. M., pers. obs. of CM 81512), *Broiliellus* (Carroll, 1964: 198), *Cardiocephalus* (CG78) and apparently *Batrachiderpeton* (Bossy & Milner, 1998: fig. 63A) have state 0.

Centra are altogether unknown in *Leptorophus* (Boy, 1987; Werneburg, 2007a); they probably only ossified during metamorphosis (if metamorphosis ever occurred in *Leptorophus*). We have accordingly scored it as unknown.

The rib-bearers of **Pangerpeton* are not bicipital, because the ribs are single-headed (an autapomorphy of Cryptobranchoidea: Gao & Shubin, 2012), but because they are still rib-bearers as seen in salamanders and almost nowhere else, we have scored state 1 for **Pangerpeton*.

We have kept state 0 for all diadectomorphs for the time being, but should point out that the condition of at least *Orobates* (Nyakatura et al., 2015: digital model) is remarkably similar to state 1 and should likely be counted as such.

265. TRU VER 13-14: **Trunk intercentra: fused middorsally (0), separate middorsally (1), absent (2) (ordered).** Intercentra in state 1 have a wide range of sizes; usually they are crescent-shaped, and their dorsal tips do not touch. Evidently, this is a single continuous character, with the degree of intercentrum ossification gradually decreasing from state 0 to state 2 (though something in the middle of state 1 is the plesiomorphy). We assign state 0 or 2 to taxa with single-piece centra throughout the column that cannot (without phylogenetic bracketing) be identified as pleuro- or intercentra (see TRU VER 7).

Chenoprosopus (Hook, 1993; D. M., pers. obs. of USNM 437646) and *Cochleosaurus* (Sequeira, 2009) have state 1, as does *Cardiocephalus* (CG78: fig. 31).

Isodectes has state 0 or 1 (D. M., pers. obs. of CM 81430).

Albanerpetidae seems to have axis intercentra (Material and methods: Modifications to individual cells: The albanerpetid neck), but lacks trunk intercentra, giving it state 2.

Solenodonsaurus is known (Carroll, 1970: 294–295; Danto, Witzmann & Müller, 2012) to have very small intercentra that are very far from reaching the dorsal edge of the pleurocentra (or rather the notochord) and thus cannot possibly have been fused middorsally; this means state 1.

Orobates was scored as unknown by RC07. A photo of a specimen containing the first six vertebrae shows that only the first four, counted as "neck" rather than "trunk", have intercentra (Berman et al., 2004: fig. 11); this suggests state 2. In the digital model of Nyakatura et al. (2015), all vertebrae behind the sixth have intercentra (state 1); they are, however, modeled after an isolated neural arch + pleurocentrum because the vertebrae in the articulated specimens were apparently too crushed to reconstruct. Given the presence of state

1 in *Diadectes* (as already scored) and the presence of intercentra in caudal, but not cranial or middle, trunk vertebrae in *Eocasea* (*Caseasauria), which we have also counted as state 1, we have tentatively assigned state 1 to *Orobates*. This is probably not the best score; John Nyakatura (pers. comm. March 2018) thinks this character is most likely unknown in *Orobates*.

Unexpectedly, **Nigerpeton* has state 0 (D. M., pers. obs. of MNN MOR 69).

266. TRU VER 15: **Anteroposteriorly elongate, lateral and ventral carinae on trunk centra: absent (0); present (1).** We have assigned state 0 in cases where ventral carinae occur but lateral ones do not (e.g. *Westlothiana*: Smithson et al., 1994: fig. 9B, C; **Archegosaurus*: Witzmann & Schoch, 2006; **Lydekkerina*: Pawley & Warren, 2005 – though see Hewison, 2008).

Chenoprosopus (Hook, 1993; D. M., pers. obs. of USNM 437646) and *Cochleosaurus* have state 0 (Sequeira, 2009), and so do *Cardiocephalus* (CG78: fig. 31) and *Orobates* (Berman et al., 2004: fig. 11).

RC07 scored only *Brachydectes* and the adelogyrinids as having state 1, and all other OTUs with preserved centra except the above as having state 0. It is possible that they meant to restrict this character to taxa with monospondylous centra; but neither would that make sense, nor did they score diplospondylous OTUs as unknown. We have ascribed state 1 to any inter- or pleurocentra that have the described carinae. Thus, weak but clear cases of state 1 – where the ventral carinae are not fully distinct from each other but more or less form a very wide median carina – are found in *Colosteus* (Hook, 1983), *Greererpeton* (only in caudal dorsals: Godfrey, 1989: 85, fig. 6d, f), *Neldasaurus* (Chase, 1965) and *Trimerorhachis* (Chase, 1965; Pawley, 2007). State 1 is also found in *Eoherpeton* (Smithson, 1985: fig. 16; “horizontal grooves” on p. 349), *Proterogyrinus* (Holmes, 1984), *Archeria* (Holmes, 1989), *Pholiderpeton attheyi* (Panchen, 1966), apparently *Ph. scutigerum* (Clack, 1987b: especially fig. 21f), and – a particularly striking case – *Acherontiscus* (Carroll, 1969a). Given the additional occurrence in **Pholidogaster* (Romer, 1964), we strongly suspect that state 1 is universal in anguilliform swimmers; its strong phylogenetic signal (all colosteids, all anthracosaurs, all adelospondyls, *Brachydectes*, both dvinosaurs – scored as unknown for *Isodectes*, which D. M. forgot to check during his collection visits, and for **Erpetosaurus* [Milner & Sequeira, 2011]; five steps, no reversals) may well be entirely ecological. All aïstopods seem to have state 0 as already scored for the three in the original sample, which may be additional evidence that some of them were terrestrial (Anderson, 2002, 2003a; Germain, 2008a, b); notably, we could not score **Coloraderpeton*.

Unclear in *Bruktererpeton* (Boy & Bandel, 1973).

Centra are altogether unknown in *Leptorophus* (Boy, 1987; Werneburg, 2007a); they probably only ossified during metamorphosis (if metamorphosis ever occurred in *Leptorophus*). We have accordingly scored it as unknown.

267. TRU VER 16: **Strong proximal emargination along anterior and posterior margins of haemal spines: absent (0); present (1).** RC07 added “of tail vertebrae” at the end; that is redundant.

The entire tail is unknown in *Colosteus* (Hook, 1983), *Crassigyrinus* (Panchen, 1985), *Neldasaurus* (Chase, 1965), *Broiliellus* (Carroll, 1964), *Eoherpeton* (Smithson, 1985), *Pholiderpeton scutigerum* (Clack, 1987b), *Gephyrostegus* (Carroll, 1970; Godfrey & Reisz, 1991), *Solenodonsaurus* (Laurin & Reisz, 1999; Danto, Witzmann & Müller, 2012), *Stegotretus* (Berman, Eberth & Brinkman, 1988) and *Ariekanerpeton* (Klembara & Ruta, 2005b). Similarly, no hemal arches are preserved in *Triadobatrachus* (Roček & Rage, 2000; Ascarrunz et al., 2016), and none have been described in *Leptorophus*.

Doleserpeton has state 0 as scored, but one wonders how this was done before Sigurdsen & Bolt (2010) described (any part of) the tail for the first time in a publication; Ruta, Coates & Quicke (2003) did not claim to have seen specimens and did not cite Bolt's unpublished thesis (which dates to 1964).

Platyrhinops has state 0 (Hook & Baird, 1984; Werneburg, 2012a: fig. 9a); so do *Hyloplesion* (Olori, 2015), *Orobates* (Nyakatura et al., 2015: digital model) and *Ossinodus* (Warren, 2007).

268. TRU VER 18: **Striated ornament on vertebral centra: absent (0); present (1).** State 1 does in fact exist and occurs where it was scored in RC07 (Williston, 1909; Bossy & Milner, 1998). For this reason we have ignored the original description of this character (RC07: 109) which contradicts its name: "A 'pleated' or unevenly striated surface sculpture characterises the neural spines [!] of some of the more derived keraterpetontid [= diplocaulid] nectrideans."

State 0 is found in *Edops* (D. M., pers. obs. of MCZ specimens), *Chenoprosopus* (Hook, 1993; D. M., pers. obs. of USNM 437646), *Cochleosaurus* (Sequeira, 2009), *Isodectes* (D. M., pers. obs. of CM 81430), *Cardiocephalus* (CG78: fig. 31), *Euryodus* (CG78), *Hyloplesion* (Olori, 2015) and *Ossinodus* (Warren, 2007).

Centra are altogether unknown in *Leptorophus* (Boy, 1987; Werneburg, 2007a); they probably only ossified during metamorphosis (if metamorphosis ever occurred in *Leptorophus*). We have accordingly scored it as unknown.

269. TRU VER 19: **Tallest ossified part of neural arch in posterior trunk vertebrae situated above (aligned vertically with) posterior half of vertebral centrum: no (0); yes (1).** State 1 is much more widespread than RC07 scored it, at least if we assume that the neural spine counts as part of the neural arch, and if "entirely behind the centrum" still counts as "above […] posterior half" as opposed to the middle or the anterior half. In the absence of evidence for variation among the dorsal vertebrae, we have resorted to middle or anterior ones if no posterior ones are preserved.

Specifically, state 1 is present in *Eusthenopteron* and *Acanthostega* (Coates, 1996; Pierce et al., 2013), *Greererpeton* (Godfrey, 1989), *Whatcheeria* (Lombard & Bolt, 1995), *Cochleosaurus* (Sequeira, 2009), *Isodectes* (D. M., pers. obs. of CM 81430), *Neldasaurus* (Chase, 1965), *Trimerorhachis* (Pawley, 2007), *Balanerpeton* (Milner & Sequeira, 1994: fig. 9), Dendrerpetidae (Holmes, Carroll & Reisz, 1998), *Eryops* (Moulton, 1974), *Ecolsonia* (Berman, Reisz & Eberth, 1985), *Amphibamus* (Daly, 1994: fig. 22), *Eoscopus* (Daly, 1994: fig. 6, 7), *Platyrhinops* (Hook & Baird, 1984), *Micromelerpeton* (Boy, 1995), Albanerpetidae (Estes & Hoffstetter, 1976), *Eocaecilia* (Jenkins, Walsh & Carroll, 2007: fig. 37A), *Karaurus* (Ivachnenko, 1978), *Triadobatrachus* (Roček & Rage, 2000; Ascarrunz et al., 2016: fig. 9, 3D model 1), *Valdotriton* (Evans & Milner, 1996), *Caerorhachis* (Ruta, Milner & Coates, 2002), *Proterogyrinus* (Holmes, 1984), *Archeria* (somewhat borderline: Holmes, 1989), *Brukter-erpeton* (Boy & Bandel, 1973), *Gephyrostegus* (Godfrey & Reisz, 1991), *Solenodonsaurus* (Danto, Witzmann & Müller, 2012), *Discosauriscus* (Klembara & Bartík, 2000: fig. 4h), *Diadectes* (Berman, Sumida & Martens, 1998: fig. 13C), *Cardiocephalus* (CG78: fig. 31, 33), *Scincosaurus* (Bossy & Milner, 1998: fig. 63L), *Lethiscus* (Wellstead, 1982), *Ariekanerpeton* (Klembara & Ruta, 2005b), apparently *Vieraella* (Báez & Basso, 1996: fig. 6), *Ossinodus* (Warren & Turner, 2004) and *Pederpes* (Clack & Finney, 2008; Pierce et al., 2013). In **Australerpeton*, Dias & Schultz (2003: fig. 8) reconstructed state 0, but their photo and drawing of an articulated specimen (fig. 2, 3) clearly show state 1, which we have therefore scored.

We have retained state 0 for the unusual lumbar vertebrae of *Ichthyostega* (Pierce et al., 2013: fig. 1d); for *Batrachiderpeton* and *Diploceraspis* (Bossy & Milner, 1998: fig. 63) as well as, somewhat tentatively, *Diplocaulus* (Williston, 1909: pl. 3); for *Eoherpeton*, where the highest point of the neural spine is between the inter- and the pleurocentrum, in the middle of the centrum as a whole (Smithson, 1985: fig. 19); for *Pholiderpeton attheyi*, where the highest point is at least that far cranial in middle trunk vertebrae (more caudal trunk vertebrae are insufficiently preserved; Panchen, 1966); very tentatively for *Ph. scutigerum*, where at least some neural arches show state 0 even though it is less clear where in the column they come from (Clack, 1987b); and for neural spines with a horizontal flat top where the entire dorsal edge is the tallest "point" and covers at least part of the cranial half of the centrum (*Keraterpeton*, *Diceratosaurus*, *Ptyonius*, *Sauropleura*, *Urocordylus*: Bossy & Milner, 1998: fig. 61, 63, 76; **Karpinskiosaurus*: Bystrow, 1944: fig. 22). State 0 further occurs in **Nigerpeton* (D. M., pers. obs. of MNN MOR 83) and *NSM 994 GF 1.1 (Holmes & Carroll, 2010).

Unknown in *Colosteus* (Hook, 1983), *Kotlassia* (Bystrow, 1944: 409) and *Utegenia* (Klembara & Ruta, 2004b). **Llistrofus* has state 1 in cranial to midtrunk vertebrae, but the preservation makes the condition of caudal trunk vertebrae unclear (CG78).

270. TRU VER 20-21-22-23-24-25: **Zygapophyses absent throughout, or nearly so (0), present only on trunk and proximal tail vertebrae (1), or present throughout (2) (ordered).** These originally six characters concerned the presence of prezygapophyses (TRU VER 20, 22 and 24) and postzygaphopyses (TRU VER 21, 23 and 25) in the trunk (TRU VER 20 and 21) and the proximal (TRU VER 22 and 23) and distal tail (TRU VER 24 and 25).

One would at first think (as Pawley [2006: 205] did) that pre- and postzygapophyses only occur together because they articulate with each other. The reason given by RC07 for keeping them as separate characters was that *Crassigyrinus* and *Trimerorhachis* had been reported to possess pre- but lack postzygapophyses.

In *Trimerorhachis*, however, the postzygapophyses merely happen not to be visible in lateral view in tail and caudal trunk vertebrae because the neural spine is continuous with them (much like in **Acanthostomatops*: Witzmann & Schoch, 2006a); they are unambiguously present throughout the trunk and tail, like the prezygapophyses (Pawley, 2007), so we have assigned state 2 to *Trimerorhachis*.

In *Crassigyrinus*, the situation is less clear. Panchen (1985: 534) described two neural arch pairs as showing "no sign" of a postzygapophysis and then stated: "Postzygapophyses, formerly thought of (with prezygapophyses) as an autapomorphous feature of tetrapods, appear to be absent." Two pages earlier, however, we find the statement: "The neural arches of *Crassigyrinus* are remarkable for their primitive or degenerate condition. All those visible occur as separate bilateral halves with no sign of suture or fusion in the middle. They lack clear zygapophyses of tetrapod type and they are very small for the size of the animal. None has been found in natural articulation with a centrum and in the absence of clearly defined matching articular facets on each it is not clear precisely what their mutual orientation was." This implies quite strongly that prezygapophyses are also absent – yet, prezygapophyses (though unusually small ones) are illustrated and described as present. In the light of this, it must be wondered if postzygapophyses were likewise present – not necessarily as processes visible in lateral view, but at least as articular facets. Indeed, fig. 17(*e*), which shows the presumed left atlas arch in left lateral and cranial views, shows a convex surface that would fit into the concave craniodorsal margin of the prezygapophysis of the same arch. This convex surface is overhung by the craniocaudally very broad neural spine. We consider it likely that *Crassigyrinus* simply has a less well ossified version of the condition seen in *Trimerorhachis*.

(The vertebrae of *Crassigyrinus* are indeed as poorly ossified as the quote above implies; fig. 17(*a*)–(*c*) shows three pairs of incompletely fused left and right intercentra, the broadest pair measuring more than 2 cm from side to side.) At least, this interpretation is more probable than the occurrence of prezygapophyses that have nothing to articulate with. The tail of *Crassigyrinus* being unknown, we have scored *Crassigyrinus* as showing state 1 or 2.

The reason given for keeping the zygapophyses of the trunk, the proximal tail and the distal tail as three separate pairs of characters was "to account for the possibility that acquisition of fully developed and ossified zygapophyses occurred in a 'stepwise' fashion along the backbone (e.g. tail of certain Devonian taxa, notably *Acanthostega* and *Ichthyostega*; trunk of *Crassigyrinus* and *Trimerorhachis*)" (RC07: 110). First, to the best of our knowledge, it is never observed, and would be unexpected from functional considerations, that prezygapophyses occur in the distal but not the proximal part of the tail. This confirms the suspicion of the "'stepwise' fashion" by RC07. Second, stepwise evolution can only be represented by an ordered multistate character (a meristic character, more precisely). Thus, TRU VER 22/23 and 24/25 should have been merged already by Ruta, Coates & Quicke (2003). Third, to the best of our knowledge, no animal is known that has zygapophyses in the trunk but nowhere in the tail (the urostyle of frogs perhaps excepted); the distinction between TRU VER 20/21 and 22/23 is therefore useless in this matrix. For these reasons we feel obliged to perform this megamerger of six characters into one.

Chenoprosopus (Hook, 1993; D. M., pers. obs. of USNM 437646), *Cochleosaurus* (Sequeira, 2009), *Isodectes* (D. M., pers. obs. of CM 81512 and CM 81430) and *Cardiocephalus* (CG78) have state 1 or 2.

Doleserpeton has state 2 as already scored for TRU VER 22 through 25, but one wonders how this was done before Sigurdsen & Bolt (2010) described (any part of) the tail for the first time in a publication; Ruta, Coates & Quicke (2003) did not claim to have seen specimens and did not cite Bolt's unpublished thesis (which dates to 1964).

Triadobatrachus has state 1 or 2: the distalmost tail vertebrae are poorly preserved and poorly ossified (Ascarrunz et al., 2016).

We have scored *Bruktererpeton* (Boy & Bandel, 1973) as possessing state 1 or 2 because the distal tail is not described and only visible in one illustration (the plate), the resolution of which is insufficient to determine whether zygapophyses are present. About the same holds for *Leptorophus* (Werneburg, 2007a: fig. 2, 3).

We have assigned the same score to *Westlothiana*, the distal part of whose tail is entirely unknown (Smithson et al., 1994). Similar things hold for Albanerpetidae (McGowan, 2002) and for *Microphon* (only an axis is known: Bulanov, 2014), so we have scored them the same way.

271. TRU VER 26: **Capitular facets situated on posterior rim of vertebral midtrunk centra: absent (0); present (1).** State 0 will need to be divided; the capitular facet often sits on the intercentrum when inter- and pleurocentra are both present, but it can sit in the center of a pleurocentrum in gastrocentral vertebrae. State 1 includes cases where the facet straddles two successive centra.

Gephyrostegus has state 0 (Godfrey & Reisz, 1991), as do *Cardiocephalus* (CG78) and *Orobates* (Nyakatura et al., 2015: digital model).

State 1 is found in *Scincosaurus* (Milner & Ruta, 2009). We have also assigned it to *Eocaecilia*, where the facets commonly straddle two neighboring centra (Jenkins, Walsh & Carroll, 2007).

State 1 was scored for *Batropetes* in RC07; however, capitular facets are altogether absent in *Batropetes* (Glienke, 2015), so we have changed the score to unknown.

272. TRU VER 27: **Height of the ossified portion of the neural arch in midtrunk vertebrae greater (0) or smaller (1) than the distance between pre- and postzygapophyses.**

Chenoprosopus has state 0 (D. M., pers. obs. of USNM 437646). So does **Nigerpeton* (D. M., pers. obs. of MNN MOR 83).

State 1 is found, at least, in *Cochleosaurus* (Sequeira, 2009: fig. 12), *Trimerorhachis* (Pawley, 2007: fig. 5.1), *Amphibamus* (Daly, 1994: fig. 22), *Doleserpeton* (Sigurdsen & Bolt, 2010), *Eoscopus* (Daly, 1994: fig. 6, 7), Albanerpetidae (Estes & Hoffstetter, 1976), *Eocaecilia* (Jenkins, Walsh & Carroll, 2007), *Karaurus* (M. L., pers. obs. of unnumbered MNHN cast of PIN 2585/2), *Valdotriton* (judging from cranial dorsals and proximal caudals: Evans & Milner, 1996: fig. 9a, 10), *Cardiocephalus* (CG78: fig. 31, 33), *Scincosaurus* (Bossy & Milner, 1998: fig. 63; Milner & Ruta, 2009), *Diplocaulus*, *Diploceraspis* and *Ptyonius* (Williston, 1909: pl. 3; Bossy & Milner, 1998: fig. 61, 63), *Urocordylus* (borderline: Bossy, 1976), all aïstopods (Wellstead, 1982; Anderson, 2002, 2003a) and *Orobates* (Nyakatura et al., 2015: digital model).

Tseajaia is given state 0 because that state is found in the vertebrae with the dorsoventrally longest neural spines (Moss, 1972).

Unknown in *Kotlassia* (Bystrow, 1944: 409); borderline and probably not completely prepared in the photo of **Chroniosaurus* (Clack & Klembara, 2009: fig. 8).

We have scored state 0 for **Coloraderpeton* because the neural spines are described as "tall" or "high" in two of the three accessible sources (Carroll, 1998b; Anderson, 2003a). The third, however, scored them as "low" as opposed to "high" without quantifying these states (Anderson, Carroll & Rowe, 2003: table A1).

273. TRU VER 28: **Crenulations or fimbriate sculpture along dorsal margin of ossified portion of neural spines: absent (0); present (1).**

Edops (D. M., pers. obs. of MCZ 7136) has state 0, as do *Cochleosaurus* (Sequeira, 2009), *Isodectes* (D. M., pers. obs. of CM 81430), *Triadobatrachus* (Roček & Rage, 2000), *Cardiocephalus* and *Euryodus* (CG78), *Hyloplesion* (pers. obs. of NHMW specimens; Olori, 2015) and *Ossinodus* (Warren & Turner, 2004).

Inapplicable to *Diplocaulus*, the neural spines of which are too small to be ornamented (Williston, 1909; Douthitt, 1917).

274. TRU VER 29: **Intravertebral foramina for spinal nerves in at least some trunk vertebrae: absent (0); present (1).**

Cardiocephalus has state 0 (CG78: fig. 31).

Sauropleura is polymorphic (Bossy & Milner, 1998; Milner & Ruta, 2009: matrix).

We have scored state 1 for **Pseudophlegethontia* following the matrix of Anderson, Carroll & Rowe (2003).

275. TRU VER 30: **Transverse processes stout and abbreviated, the length of which is less than 30% of neural arch height: absent (0); present (1).** State 0, which D. M. has observed in *Isodectes* (CM 81512), probably hides some phylogenetically informative diversity. For instance, Carroll & Chorn (1995: 49f.) mentioned that adelogyrinids (scored 1 in RC07) "are unique among lepospondyls in having very *long* transverse processes […] as in primitive labyrinthodonts" (emphasis ours), implying that the other "lepospondyls" have an intermediate condition (in terms of length) between those seen in "primitive labyrinthodonts" and adelogyrinids on the one hand and seymouriamorphs and diadectomorphs (which were scored as having state 0) on the other hand.

Anyway, *Adelospondylus* and *Adelogyrinus* have state 0 (Andrews & Carroll, 1991), and the condition in *Acherontiscus* is unknown (Carroll, 1969a). State 0 further seems to be known in *Cardiocephalus* (CG78: fig. 31). This leaves state 1 exclusively to *Colosteus* and *Greererpeton*.

276. DIG 1-2-3-4: **"Independent radials" (0); polydactyly (1); pentadactyly (2); tetradactyl forelimb (3); tridactyl forelimb (4) (ordered).** RC07 treated the presence/absence of digits (DIG 1), the presence/absence of four or fewer fingers per hand (DIG 2), the presence/absence of five or fewer fingers per hand (DIG 3), and the presence/ absence of three or fewer fingers per hand (DIG 4) as completely independent characters. It goes without saying that, if a taxon has three or fewer fingers per hand, it also has fewer than four and fewer than five, yet RC07 did not even provide for these cases by scoring inapplicability. We have therefore merged all these characters, except for splitting DIG 1 to differentiate the mere absence of digits (state 0 of the present character) from the wholesale absence of limbs (state HUM 18/DIG 1(0), see ch. 219).

The present character differs from DIG 5 of Germain (2008a) by being ordered, containing partial uncertainty, and defining state 0 of this character and of ch. 219 (his states DIG 5(0) and DIG 5(5)) morphologically where Germain (2008a) had called them "primary absence of digits" and "secondary absence of digits", which should be an inference from the results of the analysis and not an assumption of coding. It differs from DIG 1 of Ruta & Bolt (2006) in not splitting polydactyly into two states (eight and six fingers per hand, each only present in a single OTU), in providing for OTUs with three fingers per hand (which do not occur in the matrix of Ruta & Bolt, 2006), and again in being ordered.

We have not counted the prepollex/-hallux, where identifiable as such, as a digit, because it is not homologous to an "independent radial" (Johanson et al., 2007) and because it is so common in otherwise four-fingered lissamphibians (if only, in most cases, as something like a distal carpal). The postminimus of *Tulerpeton* does count, but the possibly homologous pisiform bone does not, because its homology is unclear, because it is only a carpal without a digit, and because it is common in less-than-pentadactyl hands.

In relation to a pentadactyl limb, a tetradactyl one can have digits I–IV or II–V. We have ignored this issue, treating all tetradactyl hands as primarily homologous, but see Marjanović & Laurin (2013a) for discussion.

Greererpeton has often been thought to have tetradactyl hands, but Coates (1996: 415) mentioned and illustrated a well preserved hand with five fingers and mentioned another that preserves four, one of which is the distinctively small fifth. Accordingly, we have scored *Greererpeton* as having state 2, even though the closely related *Colosteus* really does seem to have only four fingers per hand as scored by RC07 (the third is the longest, as common in tetradactyl limbs, not the fourth as would usually be expected in a pentadactyl one; Hook, 1983). – D. M. has not been able to find the fourth finger in the articulated hand of AMNH 6917 (pers. obs.), but there is sufficient space for it.

Only state 0 can be ruled out for *Crassigyrinus* (Panchen, 1985; Panchen & Smithson, 1990), *Baphetes* (Milner & Lindsay, 1998), *Edops* (D. M., pers. obs. of MCZ 7126 and MCZ 7274), *Ecolsonia* (Berman, Reisz & Eberth, 1985 – inferred from the hindlimb), *Eocaecilia* (Jenkins, Walsh & Carroll, 2007), *Pholiderpeton scutigerum* (Clack, 1987b), *Kotlassia* (Bystrow, 1944 – inferred from the hindlimb), *Stegotretus* (Berman, Eberth & Brinkman, 1988), *Saxonerpeton* (CG78: 38), *Asaphestera*, *Pelodosotis*, *Cardiocephalus*, *Euryodus* (CG78), *Ossinodus* (Warren, 2007) and *Pederpes* (Clack & Finney, 2005); we have thus scored them all, and several OTUs which we added, as having state 1, 2, 3, or 4.

The same appears to hold for *Whatcheeria* (Lombard & Bolt, 1995: 483; Bolt & Lombard, 2000: 1049), even though the latter source makes state 4 appear unlikely.

In *Eucritta*, the same holds. The hindlimb does appear to be pentadactyl, which would strongly suggest five or fewer fingers in the forelimb, but we do not think polydactyly – especially a small postminimus like in the hand of *Tulerpeton* – can be ruled out.

Cochleosaurus has state 3 (Sequeira, 2009). So does *Isodectes* (D. M., pers. obs. of USNM 4471, USNM 4555, and CM 81430).

Trimerorhachis has state 2 or 3 (Case, 1935; Pawley, 2007).

Dendrysekos (Dendrerpetidae) preserves four distal carpals (Holmes, Carroll & Reisz, 1998). That most likely means four or five fingers (state 2 or 3).

No metacarpals or fingers are preserved in *Acheloma cumminsi*, but there appear to have been five distal carpals (Olson, 1941), of which the tiny preaxialmost one could belong to a prepollex; this means four or five fingers and thus state 2 or 3. Dilkes (2015a) described only four distal carpals (all set in a plaster sculpture), but did not explicitly exclude (or mention) the possibility of a fifth beyond the absence of an articulation facet for one on distal carpal 4. – *A. dunni* only preserves two fragments of pedal phalanges (Polley & Reisz, 2011).

Doleserpeton has state 3 (Sigurdsen & Bolt, 2009, 2010), as do *Platyrhinops* (Carroll, 1964) and *Leptorophus* (judging from the drawings in Werneburg, 2007a).

Brukterpeton can safely be given state 2 (Boy & Bandel, 1973: 63 and fig. 14).

Solenodonsaurus has at least four metacarpals (Carroll, 1970; Danto, Witzmann & Müller, 2012), giving it state 1, 2, or 3.

Westlothiana preserves parts of four fingers (Smithson et al., 1994) and may have had more, meaning state 1, 2, or 3.

Keraterpeton has state 3 (A. C. Milner, pers. comm., September 2009) as scored by RC07. This agrees with Bossy & Milner (1998), contradicting Bossy (1976) and possibly Jaekel (1903: fig. 2), probably agreeing with pl. XIX of Huxley & Wright (1867), and contradicting the text of Huxley & Wright (1867) which mentions five metacarpals and fingers.

Diceratosaurus, however, has state 2 (A. C. Milner, pers. comm., September 2009; D. M., pers. obs. of MB.Am.776, “Dicey 2-hands” = CM 34617, CM 81504, CM 81508, CM 25468, and AMNH 6933, the type specimen), agreeing with Jaekel (1903: three times explicitly, and pl. IV-6, which shows MB.Am.776) and with Bossy (1976) but contradicting Bossy & Milner (1998).

Diplocaulus has state 2 or 3 (Douthitt, 1917).

In **Erpetosaurus*, four incomplete fingers are preserved next to a break (Milner & Sequeira, 2011: fig. 8). We have scored state 1, 2 or 3.

deleted DOR FIN 1: **Ossified lepidotrichia in dorsal fin: present (0); absent (1).** As RC07 pointed out, this character is parsimony-uninformative, so we have deleted it.

277. CAU FIN 1: **Ossified lepidotrichia in caudal fin: present (0); absent (1).**

State 0 is probably present in *Ventastega* (Ahlberg et al., 2008); we have tentatively scored it accordingly.

State 1 is found in *Isodectes* (D. M., pers. obs. of MCZ 6044, a cast of USNM 4481) and seems to be a safe inference for *Platyrhinops* (Clack & Milner, 2010; Werneburg, 2012a: fig. 9a).

Although there is no evidence for lepidotrichia or endoskeletal radials around the 34 preserved tail vertebrae of *Brukterpeton*, in all but the first few very little is known beyond the centra, and what is known does not seem to preclude a tail fin skeleton (Boy & Bandel, 1973); we have kept the score as unknown.

Complete tails of *Hyloplesion*, preserving state 1, are known (CG78).

We have scored both *Proterogyrinus* and *Archeria* as unknown, because at least the 20 distalmost tail vertebrae in the latter (Holmes, 1989) and more in the former (Holmes, 1984) are unknown. Some discussion is provided by Clack (2011a).

Further unknown in *Broiliellus* (the entire tail is unknown; Carroll, 1964), *Doleserpeton* (as already scored; although Sigurdsen & Green [2011: appendix 2] reported state 1, and although a tail fin is of course unexpected in this terrestrial or at most amphibious animal, the middle and distal parts of the tail are unknown: Sigurdsen & Bolt, 2010), *Gephyrostegus* (the entire tail is unknown: Carroll, 1970), *Acherontiscus* (the tail tip, and possibly the entire tail, is unknown: Carroll, 1969a), apparently *Silvanerpeton* (the known tails are very poorly preserved: Clack, 1994b: 375, fig. 1, 2; Ruta & Clack, 2006: fig. 9C) and *Tseajaia* (almost the entire tail is unknown; Moss, 1972).

We have also scored *Caerorhachis* as unknown: only the first 16 or so caudal vertebrae are preserved (Holmes & Carroll, 1977; Ruta, Milner & Coates, 2002), and the first caudal with a supraneural radial in **CM 34638 appears to be around number 17 (Clack, 2011b).

deleted BAS SCU 1: **Basal scutes: present (0); absent (1).** As RC07 pointed out, this character is parsimony-uninformative, so we have deleted it.

Additional references

References not listed here are cited in the main text. In some cases, “a” cited here is absent there (e.g. “Schoch (2002a)” is cited as “Schoch (2002)” in the main text, Schoch (2002b) is cited as such here because it is not cited in the main text).

Ahlberg PE, Clack JA, Blom H. 2005. The axial skeleton of the Devonian tetrapod *Ichthyostega*. *Nature* 437:137–140. DOI 10.1038/nature03893

Ahlberg PE, Clack JA, Lukševičs E. 1996. Rapid braincase evolution between *Panderichthys* and the earliest tetrapods. *Nature* 381:61–64. DOI 10.1038/381061a0

Anderson JS, Reisz RR. 2003. A new microsaur (Tetrapoda: Lepospondyli) from the Lower Permian of Richards Spur (Fort Sill), Oklahoma. *Canadian Journal of Earth Sciences | Revue canadienne des Sciences de la Terre* 40:499–505. DOI 10.1139/e02-066

Averianov A, Martin T, Skutschas PP, Rezvyi AS, Bakirov AA. 2008. Amphibians from the Middle Jurassic Balabansai Svita in the Fergana depression Kyrgyzstan (Central Asia). *Palaeontology* 51:471–485. DOI 10.1111/j.1475-4983.2007.00748.x

Blom H. 2005. Taxonomic revision of the Late Devonian tetrapod *Ichthyostega* from East Greenland. *Palaeontology* 48:111–134. DOI 10.1111/j.1475-4983.2004.00435.x

Bolt JR. 1969. Lissamphibian origins: possible protolissamphibian from the Lower Permian of Oklahoma. *Science* 166:888–891. DOI 10.1126/science.166.3907.888

Bolt JR. 1974. Osteology, function, and evolution of the trematopsid (Amphibia: Labyrinthodontia) nasal region. *Fieldiana: Geology* 33:11–30.

Bolt JR. 1977. Dissorophoid relationships and ontogeny, and the origin of the Lissamphibia. *Journal of Paleontology* 51:235–249. Available at https://www.jstor.org/stable/1303603

Bolt JR. 1979. *Amphibamus grandiceps* as a juvenile dissorophid: evidence and implications. In: Nitecki MH, ed. *Mazon Creek Fossils*. New York/San Francisco/London: Academic Press/Harcourt Brace Jovanovich, 529–563.

Bolt JR. 1991. Lissamphibian origins. In: Schultze H-P, Trueb L, ed. *Origins of the Higher Groups of Tetrapods—Controversy and Consensus*. Ithaca: Cornell University Press, 194–222.

Boy JA. 1988. Über einige Vertreter der Eryopoidea (Amphibia: Temnospondyli) aus dem europäischen Rotliegend (? höchstes Karbon – Perm). 1. *Sclerocephalus*. *Paläontologische Zeitschrift* 62:107–132. DOI 10.1007/BF02989838

Brough MC, Brough J. 1967. Studies on early tetrapods. III. The genus *Gephyrostegus*. *Philosophical Transactions of the Royal Society* B 252:147–165. DOI 10.1098/rstb.1967.0006

Carroll RL. 1968. The postcranial skeleton of the Permian microsaur *Pantylus*. *Canadian Journal of Zoology* 46:1175–1192. DOI 10.1139/z68-168

Carroll RL. 1998c. Order undesignated. In: Carroll RL, Bossy KV, Milner AC, Andrews SM, Wellstead CF, ed. *Lepospondyli* – part 4 of Wellnhofer P, ed. *Handbuch der Paläoherpetologie | Encyclopedia of Paleoherpetology.* Stuttgart: Gustav Fischer, 183–185.

Carroll RL, Andrews SM. 1998. Order Adelospondyli WATSON 1929. In: Carroll RL, Bossy KV, Milner AC, Andrews SM, Wellstead CF, ed. *Lepospondyli* – part 4 of Wellnhofer P, ed. *Handbuch der Paläoherpetologie | Encyclopedia of Paleoherpetology.* Stuttgart: Gustav Fischer, 149–162.

Carroll RL, Baird D. 1968. The Carboniferous amphibian *Tuditanus* [*Eosauravus*] and the distinction between microsaurs and reptiles. [Brackets in the original.] *American Museum Novitates* 2337:1–50. Available at http://hdl.handle.net/2246/2547

Case EC. 1935. Description of a collection of associated skeletons of *Trimerorhachis*. *Contributions from the Museum of Paleontology, University of Michigan* IV:227–274. Available at https://deepblue.lib.umich.edu/handle/2027.42/48206

Čerňanský A, Witzmann F, Klembara J, van Heteren AH. 2016. Computed tomography and the morphological variability of foramina and canals in the quadratojugal of basal tetrapods. *The Anatomical Record* 299:1073–1079. DOI 10.1002/ar.23373

Clack JA. 1994a. *Acanthostega gunnari*, a Devonian tetrapod from Greenland; the snout, palate and ventral parts of the braincase, with a discussion of their significance. *Meddelelser om Grønland, Geosciences* 31:1–24.

Clack JA. 1994b. *Silvanerpeton miripedes*, a new anthracosauroid from the Viséan of East Kirkton, West Lothian, Scotland. *Transactions of the Royal Society of Edinburgh: Earth Sciences* 84:369–376. DOI 10.1017/S0263593300006179

Clack JA. 2000. The origin of tetrapods. In: Heatwole H, Carroll RL, ed. *Amphibian Biology*. Chipping Norton: Surrey Beatty & Sons, 979–1029.

Clack JA. 2002. The dermal skull roof of *Acanthostega gunnari*, an early tetrapod from the Late Devonian. *Transactions of the Royal Society of Edinburgh: Earth Sciences* 93:17–33. DOI 10.1017/S0263593300000304

Clack JA. 2003b. A revised reconstruction of the dermal skull roof of *Acanthostega gunnari*, an early tetrapod from the Late Devonian. *Transactions of the Royal Society of Edinburgh: Earth Sciences* 93:163–165. DOI 10.1017/S0263593300000390

Clack JA. 2007. Devonian climate change, breathing, and the origin of the tetrapod stem group. *Integrative and Comparative Biology* 47:510–523. DOI 10.1093/icb/icm055

Clack JA, Holmes R. 1988. The braincase of the anthracosaur *Archeria crassidisca* with comments on the interrelationships of primitive tetrapods. *Palaeontology* 31:85–107. Available at https://mail.palaeo-electronica.org/publications/palaeontology-journal/archive/31/1/article_pp85-107 (HTTPS certificate expired as of 13 November 2018)

Clack JA, Milner AR. 1994. *Platyrhinops* from the Upper Carboniferous of Linton and Nýřany and the family Amphibamidae (Amphibia: Temnospondyli). In: Heidtke U, ed. *New Research on Permo-Carboniferous Faunas*. Bad Dürheim: Pollichia-Buch 29:185–191.

Clack JA, Ahlberg PE, Finney SM, Dominguez Alonso P, Robinson J, Ketcham RA. 2003. A uniquely specialized ear in a very early tetrapod. *Nature* 425:65–69. DOI 10.1038/nature01904

Cubbage CC, Mabee PM. 1996. Development of the cranium and paired fins in the zebrafish *Danio rerio* (Ostariophysi, Cyprinidae). *Journal of Morphology* 229:121–160. DOI 10.1002/(SICI)1097-4687(199608)229:2<121::AID-JMOR1>3.0.CO;2-4

Dawson JW. 1882. On the results of recent explorations of erect trees containing animal remains in the coal-formation of Nova Scotia. *Philosophical Transactions of the Royal Society of London* 173:621–659. DOI 10.1098/rstl.1882.0013

Dilkes DW. 1993. Biology and evolution of the nasal region in trematopid amphibians. *Palaeontology* 36:839–853. Available at https://mail.palaeo-electronica.org/publications/palaeontology-journal/archive/36/4/article_pp839-853 (HTTPS certificate expired as of 13 November 2018)

Dilkes DW, Reisz RR. 1986. The axial skeleton of the Early Permian reptile *Eocaptorhinus laticeps* (Williston). *Canadian Journal of Earth Sciences | Revue canadienne des Sciences de la Terre* 23:1288–1296. DOI 10.1139/e86-124

Dilkes DW, Reisz RR. 1987. *Trematops milleri* Williston, 1909 identified as a junior synonym of *Acheloma cumminsi* Cope, 1898, with a revision of the genus. *American Museum Novitates* 2902:1–12. Available at http://hdl.handle.net/2246/5185

Fox RC, Bowman MC. 1966. Osteology and relationships of *Captorhinus aguti* (Cope) (Reptilia: Captorhinomorpha). *Paleontological Contributions from the University of Kansas* 11:1–79.

Gee BM, Haridy Y, Reisz RR. 2017. Histological characterization of denticulate palatal plates in an Early Permian dissorophoid. *PeerJ* 5:e3727. DOI 10.7717/peerj.3727

Godfrey SJ. 1989. The postcranial skeletal anatomy of the Carboniferous tetrapod *Greererpeton burkemorani* Romer, 1969. *Philosophical Transactions of the Royal Society* B 323:75–133. DOI 10.1098/rstb.1989.0002

Godfrey SJ, Holmes R. 1995. The Pennsylvanian temnospondyl *Cochleosaurus florensis* Rieppel, from the lycopsid stump fauna at Florence, Nova Scotia. *Breviora of the Museum of Comparative Zoology* 500:1–25.

Good DA, Wake DB. 1992. Geographic variation and speciation in the torrent salamanders of the genus *Rhyacotriton* (Caudata: Rhyacotritonidae). *University of California Publications in Zoology* 126:xi–xii + 1–91.

Goodrich ES. 1930. *Studies on the structure and development of vertebrates* (1st ed. Vol. 1). London: Macmillan.

Gregory WK. 1950. Tetrapods of the Pennsylvanian nodules from Mazon Creek, Illinois. *American Journal of Science* 248:833–873.

Hammer WR, Hickerson WJ. 1994. A crested theropod dinosaur from Antarctica. *Science* 264:828–830. DOI 1126/science.264.5160.828

Handrigan GR, Wassersug RJ. 2007. The metamorphic fate of supernumerary caudal vertebrae in South Asian litter frogs (Anura: Megophryidae). *Journal of Anatomy* 211:271–279. DOI 10.1111/j.1469-7580.2007.00757.x

Heaton MJ. 1979. Cranial anatomy of primitive captorhinid reptiles from the Late Pennsylvanian and Early Permian Oklahoma and Texas. *Bulletin of the Oklahoma Geological Survey* 127:1–84. Available at http://ogs.ou.edu/docs/bulletins/B127.pdf

Huttenlocker AK, Small BJ, Pardo JD. 2007. *Plemmyradytes shintoni*, gen. et sp. nov., an Early Permian amphibamid (Temnospondyli: Dissorophoidea) from the Eskridge Formation, Nebraska. *Journal of Vertebrate Paleontology* 27:316–328. DOI 10.1671/0272-4634(2007)27[316:PSGESN]2.0.CO;2

Huxley TH, Wright EP. 1867. On a collection of fossil Vertebrata, from the Jarrow Colliery, county of Kilkenny, Ireland. *Transactions of the Royal Irish Academy* XXIV:351–368.

Irmis RB. 2007. Axial skeleton ontogeny in the Parasuchia (Archosauria: Pseudosuchia) and its implications for ontogenetic determination in archosaurs. *Journal of Vertebrate Paleontology* 27:350–361. DOI /10.1671/0272-4634(2007)27[350:ASOITP]2.0.CO;2

Jarvik E. 1967. The homologies of frontal and parietal bones in fishes and tetrapods. *Problèmes actuels de Paléontologie—Evolution des Vertébrés, Colloques Internationaux du Centre National de la Recherche Scientifique, Paris* 163:181–213.

Kissel RA, Dilkes DW, Reisz RR. 2002. *Captorhinus magnus*, a new captorhinid (Amniota: Eureptilia) from the Lower Permian of Oklahoma, with new evidence on the homology of the astragalus. *Canadian Journal of Earth Sciences | Revue canadienne des Sciences de la Terre* 39:1363–1372. DOI 10.1139/e02-040

Laurin M. 2011. Paleontological evidence: origin and early evolution of limbed vertebrates. In: Bels V, Casinos A, Davenport J, Gasc J-P, Jamon M, Laurin M, Renous S, ed. *How vertebrates moved onto land* (*Mémoires du Muséum national Histoire naturelle* 201). Paris: Muséum national d'Histoire naturelle, 33–73.

Lebedev OA, Clack JA. 1993. Upper Devonian tetrapods from Andreyevka, Tula region, Russia. *Palaeontology* 36:721–734. Available at https://mail.palaeo-electronica.org/publications/palaeontology-journal/archive/36/3/article_pp721-734

Linnaeus C. 1758. *Caroli Linnæi [...] systema naturæ per regna tria naturæ, secundum classes, ordines, genera, species, cum characteribus, differentiis, synonymis, locis* (10th ed. Vol. 1). Stockholm: Laurentius Salvius.

Long JA, Gordon MS. 2004. The greatest step in vertebrate history: a paleobiological review of the fish-tetrapod transition. *Physiological and Biochemical Zoology* 77:700–719. DOI 10.1086/425183

Long JA, Barwick RE, Campbell KSW. 1997. Osteology and functional morphology of the osteolepiform fish *Gogonasus andrewsae* Long, 1985, from the Upper Devonian Gogo Formation, Western Australia. *Records of the Western Australian Museum* (Suppl. 53): 1–89.

McGowan GJ. 1996. Albanerpetontid amphibians from the Jurassic (Bathonian) of Southern England. In: Morales M, ed. *The Continental Jurassic*. *Museum of Northern Arizona Bulletin* 60:227–234.

Mehl MG. 1913. A description of *Chenoprosopus milleri*, gen. et sp. nov.. In: Case EC, Williston SW, Mehl MG, ed. *Permo-Carboniferous vertebrates from New Mexico*. *Carnegie Institute of Washington Publication* 181:11–16.

Milner AC. 1980. A review of the Nectridea (Amphibia). In: Panchen AL, ed. *The Terrestrial Environment and the Origin of Land Vertebrates*. London: Academic Press, 377–405.

Milner AR. 1982. Small temnospondyl amphibians from the middle Pennsylvanian of Illinois. *Palaeontology* 25:635–664. Available at https://mail.palaeo-electronica.org/publications/palaeontology-journal/archive/25/3/article_pp635-664

Moodie RL. 1912. The skull structure of *Diplocaulus magnicornis* Cope and the amphibian order Diplocaulia. *Journal of Morphology* 23:31–44. DOI 10.1002/jmor.1050230104

Moulton JM. 1974. A description of the vertebral column of *Eryops* based on the notes and drawings of A. S. Romer. *Breviora* 428:1–44.

Müller H. 2006. Ontogeny of the skull, lower jaw, and hyobranchial skeleton of *Hypogeophis rostratus* (Amphibia: Gymnophiona: Caeciliidae) revisited. *Journal of Morphology* 267:968–986. DOI 10.1002/jmor.10454

Müller H, Oommen OV, Bartsch P. 2005. Skeletal development of the direct-developing caecilian *Gegeneophis ramaswamii* (Amphibia: Gymnophiona: Caeciliidae). *Zoomorphology* 124:171–188. DOI 10.1007/s00435-005-0005-6

Olson EC. 1951. *Diplocaulus* – a study in growth and variation. *Fieldiana: Geology* 11:57–154.

Palci A, Caldwell MW, Nydam RL. 2013. Reevaluation of the anatomy of the Cenomanian (Upper Cretaceous) hind-limbed marine fossil snakes *Pachyrhachis*, *Haasiophis*, and *Eupodophis*. *Journal of Vertebrate Paleontology* 33:1328–1342. DOI 10.1080/02724634.2013.779880

Panchen AL. 1967. The nostrils of choanate fishes and early tetrapods. *Biological Reviews* 42:374–420. DOI 10.1111/j.1469-185X.1967.tb01478.x

Pretto FA, Langer MC, Schultz CL. 2018. A new dinosaur (Saurischia: Sauropodomorpha) from the Late Triassic of Brazil provides insights on the evolution of sauropodomorph body plan. *Zoological Journal of the Linnean Society* online early: 29 pp. DOI 10.1093/zoolinnean/zly028

Reiss J. 1996. Palatal metamorphosis in basal caecilians (Amphibia: Gymnophiona) as evidence for lissamphibian monophyly. *Journal of Herpetology* 30:27–39. DOI 10.2307/1564703

Roček Z. 2000. Mesozoic anurans. In: Heatwole H, Carroll RL, ed. *Amphibian Biology*. Chipping Norton: Surrey Beatty & Sons, 1295–1331.

Roček Z. 2008. The Late Cretaceous frog *Gobiates* from Central Asia: its evolutionary status and possible phylogenetic relationships. *Cretaceous Research* 29:577–591. DOI 10.1016/j.cretres.2008.01.005

Romer AS. 1946. The primitive reptile *Limnoscelis* restudied. *American Journal of Science* 244:149–188.

Romer AS. 1969. The cranial anatomy of the Permian amphibian Pantylus. [Lack of italics in the original.] *Breviora of the Museum of Comparative Zoology* 314:1–37.

Schoch RR. 1998. Homology of cranial ossifications in urodeles: Significance of developmental data for fossil basal tetrapods. *Neues Jahrbuch für Geologie und Paläontologie* Monatshefte 1998:1–25.

Schoch RR. 2001. Can metamorphosis be recognised in Palaeozoic amphibians? *Neues Jahrbuch für Geologie und Paläontologie* Abhandlungen 220:335–367. DOI 10.1127/njgpa/220/2001/335

Schoch RR. 2002b. The evolution of metamorphosis in temnospondyls. *Lethaia* 35:309–327. DOI 10.1111/j.1502-3931.2002.tb00091.x

Schoch RR. 2014a. First evidence of the branchiosaurid temnospondyl *Leptorophus* in the Early Permian of the Saar-Nahe Basin (SW Germany). *Neues Jahrbuch für Geologie und Paläontologie* Abhandlungen 272:225–236. DOI 10.1127/0077-7749/2014/0401

Schoch RR 2014b. Amphibian skull evolution: the development and functional context of simplification, bone loss and heterotopy. *Journal of Experimental Zoology (Molecular and Developmental Evolution)* 322B:619–630. DOI 10.1002/.22599

Schoch RR, Carroll RL. 2003. Ontogenetic evidence for the Paleozoic ancestry of salamanders. *Evolution & Development* 5:314–324. DOI 10.1046/j.1525-142X.2003.03038.x

Schultze H-P, Reed JW. 2012. A tristichopterid sarcopterygian fish from the upper Middle Devonian of Nevada. *Historical Biology* 24:425–440. DOI 10.1080/08912963.2012.673599

Skutschas P, Martin T. 2011. Cranial anatomy of the stem salamander *Kokartus honorarius* (Amphibia: Caudata) from the Middle Jurassic of Kyrgyzstan. *Zoological Journal of the Linnean Society* 161:816–838. DOI 10.1111/j.1096-3642.2010.00663.x

Steen MC. 1934. The amphibian fauna from the South Joggins, Nova Scotia. *Proceedings of the Zoological Society of London* 104:465–504. DOI 10.1111/j.1096-3642.1934.tb01644.x

Wang Y, Dong L, Evans SE. 2015 (printed 2016)**.** Polydactyly and other limb abnormalities in the Jurassic salamander *Chunerpeton* from China. *Palaeobiology and Palaeoenvironments* 96:49–59. DOI 10.1007/s12549-015-0219-7

Watson DMS. 1940. The origin of frogs. *Transactions of the Royal Society of Edinburgh* 60:195–231.

Williston SW. 1912. Restoration of *Limnoscelis*, a cotylosaur reptile from New Mexico. *American Journal of Science* 34:457–468. DOI 10.2475/ajs.s4-34.203.457

Williston SW. 1916. Synopsis of the American Permocarboniferous Tetrapoda. *Contributions from Walker Museum* 1:193–236.

Worobjewa [Vorobyeva] **EJ. 1975.** Formenvielfalt und Verwandtschaftsbeziehungen der Osteolepidida (Crossopterygii, Pisces). *Paläontologische Zeitschrift* 49:44–55. DOI 10.1007/BF02988065

Zupiņš I. 2008. A new tristichopterid (Pisces, Sarcopterygii) from the Devonian of Latvia. *Proceedings of the Latvian Academy of Sciences* B 62:40–46. DOI 10.2478/v10046-008-0007-0
