# Supplementary material for: Phylogeny of Paleozoic limbed vertebrates reassessed through revision and expansion of the largest published relevant data matrix
Source: PeerJ. 2019 Jan 4;6:e5565. doi: 10.7717/peerj.5565 (PMC6322490; doi:10.7717/peerj.5565)
Supplement: Supplemental Information 2 [file peerj-07-5565-s002.pdf]

Appendix S2: Human-readable version of the revised data matrix (the computer-readable version is contained in Data S3). Polymorphism in parentheses, partial uncertainty in curly braces. The order of the original OTUs (*Eusthenopteron* through *Utegenia*; underlain in yellow as opposed to purple) is unchanged from one version of the matrix of RC07. The deleted characters and the changes to them (67 red, five green, three blue; see below) are not shown, but are documented and justified in App. S1 and counted in Data S4. Note that the added OTU Caseasauria is exclusively scored after *Eothyris*, *Oedaleops* and *Eocasea*. Abbreviations: micro., microsaur; *Ph.*, *Pholiderpeton*; *Pseudophleg.*, *Pseudophlegethontia*.

Scores unchanged from RC07 are black; state numbers that have changed as a result of mergers or changes in the order of states remain black. Green font marks new scores when a state is new, redefined or possibly redefined (in cases where we are not sure of the meaning intended by RC07) and is also used to mark all but the most trivial newly recognized cases of inapplicability (usually changes away from nonadditive binary coding) as well as the scores changed to unknown following the removal of all postcranial material from *Rhynchonkos* (Material and methods: Treatment of OTUs: *Rhynchonkos*). Blue: score changed in order to account for ontogeny (Material and methods: Treatment of characters: “Ontogeny discombobulates phylogeny”); red: score changed at face value. The distinction between green and red should not be mistaken for different degrees of certainty; those are instead discussed in App. S1. Only green is shown when red and green apply, only blue is shown when red or green and blue apply; blue font is also used for the added OTUs where appropriate (their other scores are all black). In cases of merged characters, if an OTU was scored correctly for one of the characters but incorrectly for another, only the correct one is shown (in black); the total number of 4129 colored scores for the original taxon sample (2430 red, 1620 green excepting the postcranium of *Rhynchonkos*, 79 blue; counted in Data S4) is therefore an underestimate.

	1–5	6–10	11–15	16–20	21–25	26–30	31–35	36–40	41–45
Eusthenopteron	00300	00???	?0000	00000	00000	?000?	00000	00000	000?0
Panderichthys	00110	00?0?	10000	00000	00000	1000?	00000	00100	000?0
Ventastega	00000	0?10?	?0000	0001?	00000	1{12}000	?010?	00?00	10000
Acanthostega	00200	0210?	00101	0001?	00000	12001	02111	0?100	00(01)00
Ichthyostega	00111	0010?	00010	0101?	00000	12000	02101	100(01)0	00100
Tulerpeton	2????	?????	?????	?????	?????	?????	?????	?????	?????
Colosteus	1020?	??11?	10000	??00(01)	00000	12001	02210	01(01)00	00010
Greererpeton	10101	??11?	10100	{02}1000	00000	12001	02210	01100	00010
Crassigyrinus	20001	11101	00000	200(01)0	00000	1{12}000	00211	0??00	00000
Whatcheeria	00?0?	??101	00000	000?0	00000	12001	00111	00000	10000
Baphetes	00(01)00	10101	00(01)?1	0?0??	00000	12000	00211	01100	(01)0100
Megalocephalus	00200	10101	00101	1?0??	00000	12000	(01)1211	00000	00(01)00
Eucritta	0010?	1?101	000(01)0	000??	0000?	11000	00211	00000	00000
Edops	?11?0	11100	00000	0001?	00000	12000	002?1	01100	00100

	1–5	6–10	11–15	16–20	21–25	26–30	31–35	36–40	41–45
Chenoprosopus	01100	10101	00100	1001?	00000	12000	00211	00000	00000
Cochleosaurus	01000	10100	00000	0001?	00001	12000	00201	00000	00000
Isodectes	10200	10101	10000	200?0	02000	10000	00210	00100	00000
Neldasaurus	00300	1?100	00100	10000	0000{01}	10000	00210	00000	00000
Trimerorhachis	10100	10101	10000	0000(01)	00000	11(01)00	00210	01100	00100
Balanerpeton	10100	1?101	00010	00000	00000	1(12)000	00211	01100	10000
Dendrerpetidae	10000	11101	00000	00000	00000	10000	002(01)1	11100	10100
Eryops	10000	11101	00000	0001?	10000	12000	01111	00000	00(01)00
Acheloma	10200	11100	00000	21000	00000	12100	01211	00000	00100
Phonerpeton	30200	10100	00010	21000	0(02)001	12100	01211	00000	00000
Ecolsonia	1020?	11100	00000	20000	02000	11100	01201	01000	00000
Broiliellus brevis	?020?	1?101	00010	01000	00010	10100	01201	00000	00000
Amphibamus	10100	1?101	10010	00000	0200{01}	12000	01201	00001	00000
Doleserpeton	10100	11101	00010	01000	02012	12100	01211	00001	00000
Eoscopus	30100	1{12}101	00010	0?000	01000	11100	01211	00001	00100
Platyrhinops	10100	1{12}101	00010	00000	01000	1(12)000	01201	00000	10000
Micromelerpeton	10200	11101	00000	0?0??	02000	11100	(01)1201	00000	00000
Apateon	10100	1?101	10010	0(01)000	02002	12100	01201	01000	00000
Leptorophus	?0300	1?101	10010	0?000	0{12}002	1{12}100	01211	01?00	00000
Schoenfelderpeton	?0200	1?101	10010	00000	{01}??02	1?100	01211	01100	00000
Albanerpetidae	20100	1?101	10100	01001	{01}0002	0(01)?01	?{34}211	00?2?	????1
Eocaecilia	20200	10101	10100	{02}01??	01000	11000	?3201	10000	10000
Karaurus	10000	1?101	10011	2001?	{01}?101	11?00	?{34}{12}01	10?2?	????1
Triadobatrachus	?????	??1??	?00?0	?????	0{01}?0?	???1?	?{34}???	???2?	0?0?1
Valdotriton	30200	1?100	100?0	{02}????	{01}?10{12}	10?00	?{34}?11	?0?2?	????1
Caerorhachis	0?0??	??101	0????	?????	0?000	1??00	00211	01100	00000
Eoherpeton	00200	1?101	00000	0001?	10000	12000	10211	00000	00000
Proterogyrinus	00300	1?10?	00100	010?0	00000	12000	10211	00000	10000
Archeria	00300	1?101	00100	0001?	10000	12000	10211	00100	10000
Ph. attheyi	20300	1?101	00000	1001?	00000	12000	10211	00000	10000

	1–5	6–10	11–15	16–20	21–25	26–30	31–35	36–40	41–45
Anthracosaurus	20200	1?101	00100	00000	{01}0000	12000	10211	00000	10000
Ph. scutigerum	20300	1?101	00000	{01}?01?	10?00	1??0?	10?11	0??00	10000
Bruktererpeton	2?30?	1?101	00?0?	0?0??	0?000	12100	10211	000{01}0	00000
Gephyrostegus	20200	10101	00100	00000	00000	12000	10201	01001	00000
Solenodonsaurus	??2??	1?101	00100	00000	10000	12000	11211	01100	00000
Kotlassia	??3??	??101	10010	00000	??0?0	10000	10211	00000	?00?0
Discosauriscus	00300	10101	(01)0010	00000	10000	11000	10211	00000	00100
Seymouria	20200	10100	00000	00000	10000	12000	10211	00000	00100
Diadectes	30200	10101	00000	00000	10000	12000	12211	0111(01)	1(01)100
Limnoscelis	30310	11101	00000	00000	10000	12000	12211	01111	01100
Captorhinus	20310	10101	00000	00000	10000	12100	?2211	00002	00100
Paleothyris	?0200	10101	00000	00000	10000	12100	12211	01002	00000
Petrolacosaurus	20300	1?101	00000	00000	10000	12100	12211	10002	00000
Westlothiana	20200	1011?	(01)0???	?1???	{01}??00	1200(01)	12211	00000	0?000
Batropetes	20310	?0101	10100	(02)0000	10000	1(01)100	?32(01)1	0(01)?2?	1?1?0
Tuditanus	2?3??	1?10?	00000	?0001	{12}?000	12000	?4201	0000?	???00
Pantylus	20410	10111	10000	00000	10012	10000	?4211	01100	00000
Stegotretus	20310	10101	?0000	00000	00010	11000	?4211	00000	01000
Asaphestera	20200	1?101	10000	00000	10000	12000	?4211	00000	10000
Saxonerpeton	20200	1?101	10000	00000	1?000	12?00	?4201	00001	00000
Hapsidopareion	20100	11101	00000	00000	10000	12000	?4201	01?00	01000
Micraroter	20210	11101	00000	0100(01)	1?000	11000	?4211	00000	11000
Pelodosotis	20210	1?101	00000	00001	10000	12000	?4211	00000	11000
Rhynchonkos	20210	11101	00000	00000	11000	12000	?4201	00000	01000
Cardiocephalus	20310	10101	10100	00001	11000	10000	?4211	00000	01000
Euryodus	(02)0210	10101	10000	00001	00000	10000	?4211	00000	01000
Microbrachis	20300	12101	10000	00001	10000	10000	?3211	10000	00000
Hyloplesion	20300	1?101	10000	01000	1?000	10000	?3211	11102	00000
Odonterpeton	20400	1?111	10000	00000	00000	11?00	?{34}211	10?2?	0?0?0
Brachydectes	{12}0000	1?101	10100	20000	1{01}100	10?00	?{34}21(01)	01100	11111

	1–5	6–10	11–15	16–20	21–25	26–30	31–35	36–40	41–45
Acherontiscus	?????	1?111	?0100	?0000	{01}0000	1?00?	?{34}???	?????	?????
Adelospondylus	?????	??1??	?0100	?00??	0?00?	11000	?{34}210	?1100	00010
Adelogyrinus	20?0?	1?1?1	?0000	?00??	{01}100?	10?00	?{34}?10	?11?{01}	00010
Dolichopareias	10?0?	1?1?1	?0100	200??	{01}?000	1?000	??210	?11?{01}	0?010
Scincosaurus	20300	1?100	10000	20000	20000	10000	?4211	01?2?	????0
Keraterpeton	0020?	1?100	10000	20000	{12}0000	10000	?3211	11100	00010
Batrachiderpeton	00400	1?101	10000	00000	11000	10000	?3211	11000	00110
Diceratosaurus	2030?	1?101	10100	20000	{12}1000	10000	?3211	11000	00110
Diplocaulus	20400	1?0??	?0000	0(01)01?	20000	0010?	?3211	10000	001?0
Diploceraspis	20400	1?0??	????0	{02}11??	2000?	0010?	?3211	10001	001?0
Ptyonius	00100	1?100	00000	20001	00000	10001	12210	01100	10000
Sauropleura	00200	1?1(01)0	(01)0100	20001	01000	02001	12210	01100	(01)0000
Urocordylus	?030?	1????	?????	?0001	{01}0000	?????	?????	?????	?????
Lethiscus	30100	1210?	10?10	2?000	01000	12100	12200	011{01}0	?0011
Oestocephalus	{23}0200	1?101	10100	20000	0{12}000	10000	12210	01100	11111
Phlegethontia	{23}1000	12100	?0000	{02}01??	{12}{12}000	0?101	?{34}2??	???2?	????1
Ariekanerpeton	00300	10101	00010	00000	10000	11000	10211	00000	00100
Leptoropha	?????	??1??	00000	?0???	?0001	11000	10211	00000	00?00
Microphon	?0200	10101	00000	(01)0000	10001	10000	10211	00000	00100
Capetus	10100	1?101	00000	01000	00000	12000	00201	01100	10100
Notobatrachus	00300	121?1	?1???	??1??	0?002	???1?	?{34}???	0??2?	????1
Vieraella	00100	1?1??	?1???	??1??	1??0{01}	???1?	?{34}???	???2?	????1
Orobates	30110	10101	00000	00000	10000	12000	12211	0101(12)	11100
Ossinodus	??201	?????	?0000	??000	00001	1??0?	0{02}210	00000	?01?0
Pederpes	?030?	1?1?1	?0000	01000	00000	1?0?0	00???	??100	00000
Silvanerpeton	2020?	??101	00000	00000	00000	12000	10211	00000	00000
Tseajaia	2030?	1?101	000?0	0000?	10000	12000	02211	01002	11?00
Utegenia	00300	10101	00000	00000	10000	1200(01)	10211	00001	00000
Gerobatrachus	?0200	11101	000?0	0000?	??0?0	12100	0{012}201	?11{01}{01}	??000
Chroniosaurus	3020?	1?101	00000	0001?	00000	12100	11211	00000	10000

	1–5	6–10	11–15	16–20	21–25	26–30	31–35	36–40	41–45
Micropholis	30100	11101	00010	00000	02000	11100	01201	01100	(01)0100
Nigerpeton	01100	10100	0010?	1?01?	00000	12?00	00200	01100	?0100
Saharastega	??{123}00	??101	00000	1001?	00000	10000	00100	01100	00000
Iberospondylus	10100	?0100	00000	00000	00001	12000	0{01}2?1	0??00	10100
Tungussogyrinus	30200	??101	00010	00000	???02	10000	01211	01100	00000
Utaherpeton	1030?	??11?	10100	21001	010??	11000	??1??	01???	????0
Caseasauria	3031?	1?10?	00100	00000	0(01)00(12)	12100	12210	011(01)(12)	11000
Goreville microsaur	???0?	??1??	?00??	?0???	?????	1?1??	?????	?????	?????
Sparodus	??4?0	?????	?????	?????	?????	?????	?4???	0?100	000?0
Liaobatrachus	00200	121?1	?1???	??1??	0?002	???1?	?{34}???	0??2?	1???1
Acanthostomatops	(12)0100	1(01)101	000(01)0	00000	10000	12000	012(01)1	11100	00(01)00
Deltaherpeton	2020?	??1??	?01?0	?1000	00000	1?00?	02211	01110	10000
Karpinskiosaurus	20300	10101	10000	00000	00000	10000	00211	00000	00100
Carrolla	20210	11101	10000	20001	{01}0000	1110?	?{34}211	?0?2?	1???0
NSM 994 GF 1.1	?????	?????	?0???	?????	0?0??	1?00?	10211	00100	101?0
Crinodon	00300	?010?	00100	01001	10000	12000	?4201	00000	00000
Sigournea	?????	?????	?????	?????	?????	?????	?????	?????	?????
Doragnathus	00?00	?????	?????	?????	?????	?????	?????	?????	?????
Spathicephalus	00000	??10?	0000(01)	{01}10??	{01}0000	12100	0(12)211	01100	00000
Metaxygnathus	?????	?????	?????	?????	?????	?????	?????	?????	?????
Sclerocephalus	10100	12101	00000	1001?	00000	12000	01211	01100	00100
Cheliderpeton	202?0	?0101	00000	1001?	00001	12000	01210	01100	00(01)00
Archegosaurus	10300	10100	00100	1001?	00000	12000	01(12)00	01100	00100
Konzhukovia	20100	1{12}100	00000	1001?	00000	12(01)00	01210	00100	00100
Lydekkerina	00200	10101	00000	(01)001?	00000	11000	0121(01)	11100	00100
Beiyanerpeton	10?00	1110?	10(01)00	0?000	{01}{01}102	10?00	?{34}?01	11?2?	1???1
Pangerpeton	{13}?200	????1	?????	?????	{01}??02	?????	?????	?????	?????
Ymeria	00?00	?0???	?????	?????	0?0??	?????	?????	?????	?????
Densignathus	?????	?????	?????	?????	?????	?????	?????	?????	?????
Chelotriton	10301	12101	00000	{01}01??	0?001	12?00	?{34}201	10?2?	????1

	1–5	6–10	11–15	16–20	21–25	26–30	31–35	36–40	41–45
Palatinerpeton	??200	??01	?00??	000??	0?000	??000	?{012}21?	0????	????0
Glanochthon	(13)0200	10101	00000	1001?	000(01)(012)	1200(01)	012(01)1	01100	00100
Archaeovenator	20{34}??	11101	00?00	00000	10000	12100	12201	01002	01000
Platyoposaurus	00400	1{12}100	00100	1001?	00011	12(01)00	01210	01100	00100
Trihecaton	?????	?????	?????	?????	?????	?????	?????	?????	?????
Erpetosaurus	10001	10101	10000	20000	00010	10000	02210	00100	10110
Mordex	1010?	1?100	00010	20000	{01}2001	1(12)000	01211	01100	00000
Branchiosaurus	10?0?	??101	00000	00000	{01}{01}002	11000	01201	01000	00000
Pholidogaster	0010?	?110?	?0?00	{02}100?	10010	12001	02211	00100	00000
Palaeoherpeton	{03}02?0	1?10?	00??0	?????	00000	1200(01)	10211	01100	10100
Neopteroplax	20310	1{12}101	00000	{02}0???	10010	11000	10(12)11	011{01}0	?0?00
St. Louis tetrapod	?0?0?	?{01}1??	?0???	?????	0????	1?0??	??{12}??	?????	?????
Parrsboro jaw	?????	?????	?????	?????	?????	?????	?????	?????	?????
Elginerpeton	00???	0????	?????	?????	?????	?????	?????	?????	?????
Australerpeton	00300	12100	00(01)00	1001?	00000	12000	01210	01100	00(01)00
Quasicaecilia	{012}????	??101	00100	{01}0???	{12}????	10100	?{34}211	00?2?	0?0?0
Casineria	?????	?????	?????	?????	?????	?????	?????	?????	?????
Llistrofus	??1??	??10?	10000	?0000	10000	10100	?4201	01100	01000
Bystrowiella	2030?	???0?	00000	{01}1?1?	?000?	12100	1{012}211	00001	11000
Coloraderpeton	?????	?????	?010?	20000	0?0?0	1000?	12210	01100	100?1
Pseudophlegethontia	3030?	?????	?????	?????	?????	???0?	1{012}2?0	01100	100??
Perittodus	????0	?????	?????	?????	?????	?????	?????	?????	?????
Diploradus	{02}0?0?	?????	?0???	?????	0{02}000	???0?	??{12}1?	?1100	000?0
Aytonerpeton	20?00	0?1??	?????	{02}?00?	00001	?????	?????	?????	?????

	46–50	51–55	56–60	61–65	66–70	71–75	76–80	81–85	86–90
Eusthenopteron	10000	0000?	00000	000?1	00020	00000	00000	00?0?	00000
Panderichthys	0?0?0	0000?	00000	000?1	00020	00000	00000	00?0?	20000
Ventastega	00101	?001?	10000	000?0	0002?	00?11	1000?	00010	22002
Acanthostega	0????	00010	10000	000?1	10020	00000	10000	00010	20102

	46–50	51–55	56–60	61–65	66–70	71–75	76–80	81–85	86–90
Ichthyostega	0????	01011	00000	00(01)?1	10020	00001	00000	00021	21000
Tulerpeton	?????	?????	?????	?????	?????	?????	?????	?????	?????
Colosteus	0????	010{02}0	00000	010?1	00100	0{01}?00	0000?	100{12}?	10000
Greererpeton	0????	01021	00000	000?1	10000	00000	0000?	1001?	20000
Crassigyrinus	00101	0001?	00000	000?0	00120	00001	00000	1001?	22101
Whatcheeria	01101	0101?	00000	000?0	0012?	0{01}?11	?000?	00022	22112
Baphetes	01011	0102?	(01)0000	000?0	10120	0100?	10000	10020	21200
Megalocephalus	0???1	0102?	(01)0000	001?0	10120	0010?	10000	1002(01)	21200
Eucritta	01011	010{02}?	00000	001?0	10120	0{01}??1	0000?	100??	2?{12}02
Edops	01000	0102?	00000	001?1	10120	00?01	10000	100??	20000
Chenoprosopus	01000	0101?	10000	001?0	00120	01100	10001	10022	10000
Cochleosaurus	01000	0100?	10000	(01)01?1	10120	01100	10000	10022	20000
Isodectes	0101?	00000	00000	001?0	00100	01110	?000?	10122	20000
Neldasaurus	0100?	01001	00000	001?0	10110	01010	?000?	10022	20000
Trimerorhachis	0101?	01020	10000	001?1	1(01)(01)20	00000	00000	101(12)2	21000
Balanerpeton	01010	010??	10000	001?0	00120	00010	00000	101{12}2	22002
Dendrerpetidae	010(01)0	000??	10000	001?0	001(12)0	00010	(01)0001	10(01)(12)2	(12)0(01)00
Eryops	0???0	0101?	10000	101?0	10120	00000	10000	10122	20000
Acheloma	0???0	0101?	10000	001?0	00120	00010	00001	12?12	22002
Phonerpeton	0???0	0102?	00000	000?0	00120	00010	00001	12?12	22002
Ecolsonia	0????	0100?	00000	001?0	00110	00010	?0001	12?22	21002
Broiliellus brevis	0???0	00010	11000	001?0	00110	02010	0000?	11122	21002
Amphibamus	0???0	000??	11000	001?0	10110	00010	?0001	10122	22002
Doleserpeton	0???0	0000?	01000	001?0	10110	00010	?0001	10112	22002
Eoscopus	0???0	0101?	11000	001?0	10110	00010	?0001	10112	22002
Platyrhinops	0???0	0101?	(01)1000	(01)00?0	10110	00010	?0001	10122	20002
Micromelerpeton	0???0	0100?	(01)0000	001?0	00120	00010	?000?	10112	22002
Apateon	0???0	0101?	11000	001?0	10120	00010	?000?	101(12)2	22002
Leptorophus	1???0	0101?	10000	001?0	00120	00010	?000?	10112	22002
Schoenfelderpeton	0???0	0?0??	00000	001?0	00110	0?010	?000?	10112	22002

	46–50	51–55	56–60	61–65	66–70	71–75	76–80	81–85	86–90
Albanerpetidae	?????	??1??	????1	?????	??10?	01?1?	0????	10122	2?0??
Eocaecilia	0????	??000	00?01	?????	??100	00010	?001?	10122	00100
Karaurus	?????	??1??	?1??1	?????	??000	1????	?011?	10112	2?0??
Triadobatrachus	?????	??1??	?1??1	?????	???10	?????	?????	1????	22???
Valdotriton	?????	??1??	????1	?????	??100	1????	?1???	10122	2?0??
Caerorhachis	?1?00	0?01?	00000	001?0	00120	??0??	?000?	1?122	?????
Eoherpeton	00100	0001?	10000	00??0	00?20	00010	1000?	10022	10?00
Proterogyrinus	00000	0001?	10000	001?0	00120	01110	?0000	10022	22001
Archeria	00000	0001?	10000	001?0	00120	01110	10000	10022	22002
Ph. attheyi	01000	0001?	10000	001?0	00120	01011	10000	10022	22000
Anthracosaurus	01000	0101?	11000	000?0	00120	00011	10000	10022	22001
Ph. scutigerum	00?00	0001?	1?000	00??0	00?20	00100	00000	10022	22000
Bruktererpeton	?0?00	0000?	1000?	?????	?????	?????	?????	?0022	2????
Gephyrostegus	00100	0000?	11000	001?0	00110	01010	00000	10122	22002
Solenodonsaurus	0???0	0100?	00000	001?0	0011?	01?10	00000	100??	20000
Kotlassia	01100	0103?	00000	000?0	101{12}0	0{01}?10	00000	10022	10000
Discosauriscus	00100	0003?	11000	001?0	00110	01010	00000	10022	22001
Seymouria	01100	0103?	11000	000?0	00120	01010	10000	10022	10100
Diadectes	0???(01)	0000?	01(01)10	001?0	00110	01010	00000	10122	(01)0002
Limnoscelis	0????	0002?	01000	001?0	00100	01010	1000?	10122	00000
Captorhinus	0????	001??	?0??0	000?0	00100	01110	1000?	10022	22000
Paleothyris	1????	0000?	01010	001?0	00100	01010	0000?	10?22	22002
Petrolacosaurus	1????	0000?	01010	001?0	00100	01010	0000?	10122	22002
Westlothiana	0????	1001?	11?00	?01?0	01000	0{01}?1?	?000?	10?22	1??0{01}
Batropetes	0????	??00(01)	01000	00(01)10	00110	0??(01)0	01???	?0122	(01)1102
Tuditanus	?????	??0?0	01?00	00010	01000	01?10	0000?	10?22	10000
Pantylus	0????	??001	00000	00011	01100	02010	1000?	10022	00000
Stegotretus	0????	??00?	00100	00010	01?00	02010	0000?	10022	00000
Asaphestera	0????	??0?0	01000	00010	01000	01010	0000?	10122	00001
Saxonerpeton	0????	??000	01000	00010	0?000	01010	0011?	10?22	00000

	46–50	51–55	56–60	61–65	66–70	71–75	76–80	81–85	86–90
Hapsidopareion	0????	??000	0?100	00??0	010?0	0?010	0????	10122	?000{01}
Micraroter	1????	??001	01100	00010	00000	0??10	0011?	10122	00000
Pelodosotis	1????	??001	01100	00010	00000	0??10	0011?	10122	00000
Rhynchonkos	1????	??000	00000	10110	01000	0??10	0????	10122	00000
Cardiocephalus	1????	??000	00100	00010	011?0	01010	?????	10122	00000
Euryodus	1????	??000	00100	00010	01000	00010	0011?	10122	00000
Microbrachis	0????	??000	01000	00010	01100	01010	0000?	10122	10000
Hyloplesion	0????	??000	01000	00?00	01100	01010	0000?	10122	10000
Odonterpeton	0????	??1??	?0??0	000?0	0?100	01010	0011?	10?22	00000
Brachydectes	?????	??0?1	?0?01	?????	??100	1????	?1???	1{01}122	00000
Acherontiscus	?????	?????	????0	0?0?1	10?0?	0??10	0????	1?0??	??000
Adelospondylus	0????	??11?	10???	?????	??110	01010	00000	1????	?1000
Adelogyrinus	0????	??1??	10??0	010?0	?011?	0{01}?10	?0000	1?0??	21000
Dolichopareias	0????	??1??	?0??0	010?1	10???	0{01}?10	0????	10022	??000
Scincosaurus	0????	??000	01000	00011	10101	01010	10?0?	10022	10000
Keraterpeton	0????	??000	10000	00000	10101	01?10	0000?	10022	10000
Batrachiderpeton	0????	??000	11000	00001	10101	01010	?000?	10022	00000
Diceratosaurus	?????	??000	10000	00001	10101	01?10	?000?	10022	10000
Diplocaulus	1????	??000	11000	01001	10101	01000	1000?	10?22	21000
Diploceraspis	0????	??000	11000	01000	10101	01010	1000?	10?22	21000
Ptyonius	0????	1000?	00000	100?1	10100	01010	0000?	10022	22000
Sauropleura	0????	10000	10000	100?1	10100	00010	?000?	10022	22000
Urocordylus	?????	??000	???00	100?1	10?00	0001?	0000?	1????	??0?0
Lethiscus	?????	0?00?	10000	00???	??020	01010	0000?	?0112	22000
Oestocephalus	?????	10000	00100	00???	??0{02}0	00010	?000?	100??	12000
Phlegethontia	?????	??1??	?0??0	00??0	????0	01010	?0???	12022	22000
Ariekanerpeton	00100	0003?	01000	001?0	00110	01010	0000?	10022	10001
Leptoropha	10000	000??	01000	001?0	10110	0??10	?000?	1????	10?01
Microphon	10100	0003?	01000	001?0	(01)0120	01010	(01)0000	10122	00001
Capetus	00000	000??	10000	001?0	10120	00000	10000	10122	00000

	46–50	51–55	56–60	61–65	66–70	71–75	76–80	81–85	86–90
Notobatrachus	?????	??1??	?1??1	?????	???10	1????	?(01)1??	10022	2?0??
Vieraella	?????	??1??	?1??1	?????	????0	1????	?????	101??	2????
Orobates	1???0	1000?	01110	001?0	00110	01?10	00000	10122	00002
Ossinodus	01100	0000?	00000	000?0	01120	00010	10000	0????	1?00?
Pederpes	01101	0102?	01000	000?0	00?2?	0{01}010	0000?	000??	12012
Silvanerpeton	00001	00010	10000	001?0	00110	01?10	0000?	10022	12002
Tseajaia	0???0	0000?	01110	000?0	00110	01?10	0000?	10122	21002
Utegenia	00000	0001?	01000	001?0	00110	01010	0000?	10022	12002
Gerobatrachus	????1	0100?	???00	00??0	10110	00010	00???	?01??	22002
Chroniosaurus	0???0	0000?	10000	001?0	1011?	00011	1000?	10112	22010
Micropholis	0????	01001	(01)0000	00(01)?0	10110	00010	00001	10122	2200(012)
Nigerpeton	?1101	0101?	?1000	001??	0?120	0?000	1????	?0022	20000
Saharastega	?1?0?	0?0??	?1000	001?1	00?0?	0{01}110	1000?	?{01}112	00000
Iberospondylus	0???0	0100?	10000	?01?0	10110	00?10	10001	10022	22002
Tungussogyrinus	1???0	0000?	01000	101?0	00110	0?01?	?????	10112	22???
Utaherpeton	?????	?????	????0	?0???	?0???	01?10	?0??0	?0?22	2200{12}
Caseasauria	0????	0000?	01110	001?0	00000	00?10	0001?	10122	12101
Goreville microsaur	?????	?????	????0	100??	1????	0???0	?????	?????	?????
Sparodus	?????	??0{023}?	00000	10010	11100	0??10	?0???	?????	0??0?
Liaobatrachus	?????	??1??	????1	?????	???10	1????	?0110	10112	220??
Acanthostomatops	1???0	0(01)00?	11000	(01)01?0	(01)0110	00000	10000	10(01)12	20001
Deltaherpeton	0????	0002?	00000	100?1	10100	01?00	0000?	10011	20000
Karpinskiosaurus	01101	010{12}?	11000	001?0	00120	00010	00000	10112	10000
Carrolla	0????	?????	?1??0	?01?0	01100	0{01}?10	0????	?0122	21000
NSM 994 GF 1.1	01?0?	0001?	1?00?	?????	??020	0??1?	?0000	1????	22??{12}
Crinodon	1????	??001	01000	10010	01000	01010	0000?	1??{12}?	20002
Sigournea	?????	?????	?????	?????	?????	?????	?????	?????	?????
Doragnathus	?????	?????	?????	?????	?????	?????	?????	?????	?????
Spathicephalus	0???1	0102?	11000	000??	10120	0000?	10010	10?22	2{12}2?{01}
Metaxygnathus	?????	?????	?????	?????	?????	?????	?????	?????	?????

	46–50	51–55	56–60	61–65	66–70	71–75	76–80	81–85	86–90
Sclerocephalus	0???0	000(01)?	10000	000?0	10120	0000(01)	10000	10122	20000
Cheliderpeton	1???0	000??	(01)0000	000?0	1012?	00?00	1000?	10022	20000
Archegosaurus	0???0	0101?	10000	001?0	10120	00010	10000	10022	21000
Konzhukovia	0???1	0100?	10000	000?0	10?20	01100	10000	10022	22000
Lydekkerina	0????	01011	10000	00(01)??	10120	0(01)100	10001	10122	22000
Beiyanerpeton	?????	??1??	?1??1	?????	??10?	1????	?1???	10?12	2?0??
Pangerpeton	?????	?????	?????	?????	?????	?????	?????	??1??	2????
Ymeria	?????	?????	?????	?????	?????	?0???	?0???	????{01}	?????
Densignathus	?????	?????	?????	?????	?????	?????	?????	?????	?????
Chelotriton	?????	??1??	????1	?????	??100	1????	?001?	10022	10100
Palatinerpeton	0???0	?00??	??000	00??0	?0120	0101?	?000?	10???	22?00
Glanochthon	(01)????	0(01)010	10000	001?0	(01)0120	000(01)0	10000	10022	2(12)000
Archaeovenator	0????	1000?	11?10	101?0	?0000	01010	0011?	11122	?2002
Platyoposaurus	0???1	0100?	10000	00(01)?0	10120	01010	10000	10022	20000
Trihecaton	?????	?????	?????	?????	?????	?????	?????	?????	?????
Erpetosaurus	0????	0100?	10000	000?0	10(01)20	00110	00000	10122	22000
Mordex	1???0	0000?	10000	001?0	10110	0??10	00001	12???	21002
Branchiosaurus	????0	0?01?	11?00	001?0	10010	00?10	0000?	101{12}2	22002
Pholidogaster	0????	01020	00000	000??	1?0{12}?	01?00	1000?	?00??	2{01}000
Palaeoherpeton	01000	0001?	11000	001?0	01120	01?11	?0000	10?{12}?	22000
Neopteroplax	01101	010{13}?	1?000	001?0	10120	01?01	10000	10122	22100
St. Louis tetrapod	?????	?????	?????	?????	?????	?????	?????	?????	20???
Parrsboro jaw	?????	?????	?????	?????	?????	?????	?????	?????	?????
Elginerpeton	?????	?????	????0	?0???	?0???	?????	?????	????1	?????
Australerpeton	(01)???(01)	0101?	10000	000?0	1(01)121	00010	10001	10022	22000
Quasicaecilia	1????	??1??	?1???	?????	??1?0	0?010	?????	?0122	20?02
Casineria	?????	?????	?????	?????	?????	?????	?????	?????	?????
Llistrofus	0????	??00?	00100	00110	01020	0??10	0011?	1????	10000
Bystrowiella	0???0	??02?	11100	001?0	0?110	00?10	10???	????2?	22002
Coloraderpeton	????0	110{12}?	10000	?0???	??020	00?10	00?10	000??	{01}{01}{01}00

	46–50	51–55	56–60	61–65	66–70	71–75	76–80	81–85	86–90
Pseudophlegethontia	?????	?10{01}?	1000?	?????	??0?0	?????	?000?	?0?{02}?	22?00
Perittodus	?????	?????	?????	?????	?????	0??1?	?????	?????	?????
Diploradus	?????	?????	?????	?????	?????	00?10	?000?	?????	?????
Aytonerpeton	?????	?????	?????	?????	?????	00?10	0????	?002{02}	??0??

	91–95	96–100	101–105	106–110	111–115	116–120	121–125	126–130	131–135
Eusthenopteron	02001	00000	01002	00000	00100	00000	0?0?0	00000	00?02
Panderichthys	00001	00000	01002	00000	0010{01}	000?0	0?0?0	00000	00?0?
Ventastega	0?000	00001	00000	10000	0010?	???00	??000	0????	?{01}???
Acanthostega	02001	00001	11001	10010	00200	00000	01000	00000	01001
Ichthyostega	0(01)032	00000	{01}1001	10100	00100	(01)0000	0?000	00000	00?02
Tulerpeton	0????	?????	3?01?	?01??	?????	?????	?????	?????	?0???
Colosteus	000?0	00002	20??1	????0	?010{01}	0?0?0	0?010	00000	11???
Greererpeton	00011	00002	21001	01100	00101	00000	0?010	00000	01011
Crassigyrinus	000?1	00001	3100{01}	10000	00100	00000	??000	0???0	11?0?
Whatcheeria	00012	00002	1?001	00??0	00?0{01}	010?0	?????	00???	0???1
Baphetes	00011	00002	{23}1010	01100	11100	01100	0?100	0???0	020?1
Megalocephalus	00011	00002	31010	01100	11100	01100	0?000	0???0	0(01)?11
Eucritta	000?1	0000?	??01{01}	011?0	11101	0?0?0	??100	0????	?????
Edops	00001	0000{04}	41001	01?00	?1100	011?0	?100?	00000	1?010
Chenoprosopus	00111	00003	3?012	01100	?1100	0?110	000?0	01101	12?10
Cochleosaurus	111?1	0000{04}	4?012	01100	11100	01110	010?0	01101	12?{13}?
Isodectes	010?1	0000{23}	{23}1004	01100	??10{123}	???{01}0	001?0	01100	01010
Neldasaurus	02010	0000{23}	{23}?0?2	11100	?0100	0?010	010?0	01100	01010
Trimerorhachis	00011	00003	{123}100(23)	0(01)000	00101	00010	010?0	01100	11010
Balanerpeton	020?1	00004	41012	11100	11100	01110	010?0	01110	12?1?
Dendrerpetidae	020?1	00004	?1012	11100	11100	01110	010?0	01101	12010
Eryops	00013	0000{04}	41012	01100	11101	01110	010?0	01100	120(145)0
Acheloma	02013	00004	4?012	01100	11100	01110	010?0	01101	10050
Phonerpeton	02012	00004	41012	(01)1100	11100	01110	000?0	01100	12?50

	91–95	96–100	101–105	106–110	111–115	116–120	121–125	126–130	131–135
Ecolsonia	01013	00004	41012	?1100	1110{01}	00110	010?0	01111	1?050
Broiliellus brevis	020?3	00004	41?15	01100	1?101	???20	010?0	01110	12???
Amphibamus	020?2	00004	?0014	01110	11?01	001{12}0	010?0	01111	12???
Doleserpeton	02013	00004	40114	01011	0020{23}	???10	?10?0	01111	1205{02}
Eoscopus	020?3	00004	?0013	11110	?1101	0?110	010?0	01110	12???
Platyrhinops	020?3	00004	40014	?1110	11101	01110	011?0	01110	02?{45}?
Micromelerpeton	020?1	00004	{234}1003	00100	00101	10010	010?0	01110	10???
Apateon	02014	00004	?1003	01110	00101	10010	010?0	01110	02???
Leptorophus	020?3	00004	41103	01101	00101	10010	010?0	01110	02???
Schoenfelderpeton	020?3	00004	41103	01111	00102	10000	010?0	01110	02???
Albanerpetidae	1?0?1	01014	4???{123}	?????	?????	?????	?????	?1010	1??42
Eocaecilia	1?030	00004	41105	11001	0010{23}	???10	000?0	01?00	02042
Karaurus	1?0?3	01?14	?0105	?101?	???13	???01	011?0	11110	10?4?
Triadobatrachus	??0{13}4	01?1?	????5	????1	0120{23}	???00	?10?0	0111?	???4?
Valdotriton	1?0?2	01?14	?0105	1100?	???13	???01	100?0	1111?	12042
Caerorhachis	0{12}0?1	0000?	?1011	?1100	11100	01100	10100	00100	12???
Eoherpeton	02032	00004	4{12}??{123}	????0	0?000	0?000	?01?0	00?0?	1???1
Proterogyrinus	02031	00003	4{12}??1	????0	01100	01000	0?000	0000?	1?01?
Archeria	02032	00003	????1	?????	????{01}	?0000	0?000	0000?	??011
Ph. attheyi	020?1	00003	3(12)101	01100	01101	00000	0?100	00000	?2???
Anthracosaurus	02033	00003	32101	01100	01100	00000	0??00	00000	02011
Ph. scutigerum	00?31	00003	32101	01100	0?100	00000	??100	00000	12011
Bruktererpeton	02??1	0?0??	?1012	?1100	1100{12}	?1?{123}?	0????	00000	00???
Gephyrostegus	020?2	00004	42011	01?00	11001	01120	0?000	00000	1????
Solenodonsaurus	020?2	00004	?????	?????	?????	?????	?????	?????	?????
Kotlassia	01012	0000?	????{01}	0??01	?010{01}	1?020	??000	0???0	1?010
Discosauriscus	02012	00004	{34}2010	01100	11100	11120	0?000	0???0	12?1?
Seymouria	02012	00004	42010	01100	1110(01)	11120	0?000	0???0	12010
Diadectes	0(12)024	00004	4210(01)	01101	01101	10120	0?000	00000	02?21
Limnoscelis	01023	00004	42111	01111	11101	10130	0?000	00001	121{1235}1

	91–95	96–100	101–105	106–110	111–115	116–120	121–125	126–130	131–135
Captorhinus	02020	00004	42101	01101	01103	???20	0?000	00000	02121
Paleothyris	00021	00004	42101	01101	1110?	???30	0?000	00000	02121
Petrolacosaurus	02023	01004	42111	01101	11101	10130	0?000	00000	02121
Westlothiana	020?1	0000?	????{12}	?????	11101	11100	??0?0	0000?	?????
Batropetes	02023	00104	41102	01111	0110{123}	????2	01??0	01000	12141
Tuditanus	??0?4	0000?	4???1	?????	?????	??00	??0?0	0010?	???3?
Pantylus	1?011	00004	42101	01100	11003	???00	0?000	00(01)01	12130
Stegotretus	010?2	00004	?2101	01100	11003	???00	??000	00101	12030
Asaphestera	00013	00004	41??{12}	?????	1?10?	????0	0?1?0	0011?	??140
Saxonerpeton	020?3	0000{34}	4211{12}	01101	1?101	???10	0?0?0	0000?	?2?{1345}?
Hapsidopareion	??0?{012}	0?114	4111{12}	?110?	?0101	10110	0?0??	0000?	0?13?
Micraroter	(01)2013	00104	41{01}1{12}	01101	10101	11000	1?1?1	0000?	?2130
Pelodosotis	1?0?2	00104	4???2	????1	?010?	???00	?10?1	0000?	?2130
Rhynchonkos	1?0{23}2	00004	4110{12}	00101	00100	10010	010?1	00000	12130
Cardiocephalus	(01)20?1	00104	4{12}101	00101	00101	10000	0?101	0000?	?2030
Euryodus	(01)20?1	00004	4{12}01{12}	0?101	10101	11010	0?(01)?1	0000?	?2030
Microbrachis	020?2	00003	3{01}111	01101	11101	11110	0?000	00000	02?30
Hyloplesion	020?2	00003	42111	0??01	1110{12}	11120	01000	00010	02??0
Odonterpeton	020?0	00004	42111	01101	10101	101{12}0	0??00	00000	02?{13}{02}
Brachydectes	1?031	01?14	42102	0??01	01{12}03	???02	0???0	01?00	12131
Acherontiscus	??0??	00003	?????	?????	?????	?????	?????	?????	?????
Adelospondylus	01?10	0000{34}	3???{0123}	?????	1?101	11100	0???0	0????	?????
Adelogyrinus	00??0	0000?	3????	?????	?????	?????	?????	?????	???1?
Dolichopareias	000??	00003	?????	?????	???0?	?????	?????	?????	?????
Scincosaurus	020?3	0000(34)	?1102	?11?1	11103	???{01}2	0?1?0	0000?	???4?
Keraterpeton	020?2	00003	?????	?????	?????	?????	?????	?????	?????
Batrachiderpeton	020?3	00003	31100	?1001	00103	???01	0?100	0???0	02?40
Diceratosaurus	020?3	00003	?11(01)1	11(01)01	11{12}0?	???01	011?0	0000?	12?4?
Diplocaulus	010?4	0000?	31103	01001	00103	???01	011?0	01110	1204{02}
Diploceraspis	01014	00003	31105	01001	0010{23}	???01	011?0	01110	12040

	91–95	96–100	101–105	106–110	111–115	116–120	121–125	126–130	131–135
Ptyonius	020?1	00004	41101	?1001	0010?	???00	01000	01100	02?{13}{01}
Sauropleura	02010	00004	4210{12}	01101	00103	???00	0?0?0	00000	02?{13}?
Urocordylus	????0	0000?	????1	????1	?010?	????0	??0??	0010?	???{13}?
Lethiscus	02?{13}0	01104	421?{12}	10000	??101	1?110	000?0	01000	10?12
Oestocephalus	00010	01?14	4???{012}	?????	00103	???10	0???0	0????	??012
Phlegethontia	020?0	01114	4???{12}	?????	???13	?????	?????	?0?0?	???12
Ariekanerpeton	02012	0000{34}	{234}001{01}	01100	01100	11120	0?000	0???0	02?10
Leptoropha	0201?	00?0{234}	????{01}	?????	?????	???{23}0	??0?0	0????	?????
Microphon	02013	0000{34}	{234}211{01}	00100	10100	11020	0?000	0???0	02???
Capetus	?00?1	00004	4101{12}	?110?	??10?	???10	011?0	0111?	?2???
Notobatrachus	1?0?0	11014	4010{34}	0100?	???13	???10	111?0	01110	1?042
Vieraella	1?0?2	110??	?01?{34}	?10??	????3	???10	111?0	01111	10???
Orobates	00033	00004	421?2	?11?1	01101	10120	??0?0	00000	12121
Ossinodus	001?2	0000{12}	{12}{01}11{01}	10000	?0100	10000	0?000	0???0	0????
Pederpes	????2	?0001	?1011	?????	00100	010??	0??0?	0000?	?0???
Silvanerpeton	010?1	00004	?101{01}	01100	0110?	???10	??100	0???0	1??{01}?
Tseajaia	000?4	00004	?2?0{12}	?1?01	01100	10120	0?0?0	00000	0??21
Utegenia	02014	0000{34}	{234}101{01}	01100	00101	11020	0?000	0???0	02?1?
Gerobatrachus	02??4	000??	?0115	?11??	?1202	10110	010?0	?111?	1??{1345}?
Chroniosaurus	020?1	0000{04}	?2011	01100	1010{01}	01120	0?010	00000	02?1?
Micropholis	02012	00004	4100(34)	01100	0(01)20(01)	001(12)0	0(01)0?0	01111	1(02)040
Nigerpeton	1?113	00?02	{23}?002	11100	11100	0?110	010?0	01111	01010
Saharastega	1?0{13}3	0000{34}	?{12}?12	?100?	11{01}00	?1110	011?0	01110	11?1?
Iberospondylus	020?2	00003	41012	01100	11100	01110	?1??0	01110	12010
Tungussogyrinus	02??4	00?0?	?1??3	0??0?	??100	???10	010??	01110	1????
Utaherpeton	0{12}0?1	0?004	4???{0123}	?????	1?10?	?????	?????	?????	???{13}?
Caseasauria	0(01)013	01004	4???{012}	?????	?????	???30	??0?0	0000?	???{1235}1
Goreville micro.	?????	0000?	?1?1{012}	???00	1?10?	????0	????0	?????	???3?
Sparodus	02030	00004	4????	????0	1000?	?????	?????	?????	???{34}0

	91–95	96–100	101–105	106–110	111–115	116–120	121–125	126–130	131–135
Liaobatrachus	????4	11014	40104	0101?	??13	??10	110?1	0111?	1?042
Acanthostomatops	020?3	00004	41012	11100	11101	01110	010?0	01111	10?{345}?
Deltaherpeton	000?1	00002	2?0??	????0	?????	0????	?????	?????	?????
Karpinskiosaurus	020{13}3	00004	4{12}010	00100	11100	01120	00000	0???1	10?{134}1
Carrolla	02?34	0??14	42102	01101	01101	10101	011?0	0000?	??0{45}2
NSM 994 GF 1.1	02032	000?3	?????	?????	?????	0???0	??0??	?????	??0{123}1
Crinodon	000{23}4	00004	{34}1011	11100	11100	01100	0??00	00001	1????
Sigournea	?????	?????	2????	?????	?????	?????	?????	?????	?????
Doragnathus	?????	????{12}	{12}????	?????	?????	?????	?????	?????	?2???
Spathicephalus	02034	0000?	20100	010??	????{01}	10100	0?100	0???0	02?11
Metaxygnathus	?????	?????	{12}????	?????	?????	?????	?????	?????	?????
Sclerocephalus	01011	0000(34)	41012	01100	1(01)100	010(12)0	0(01)0?0	00(01)0 (01)	(01)20{34} {02}
Chelidorpeton	000?1	00003	?????	0????	?????	?????	?????	?????	?????
Archegosaurus	00011	00003	3?012	10100	00201	10010	000?0	01100	02040
Konzhukovia	02011	00003	?1002	11000	00101	00010	010?0	01100	11050
Lydekkerina	02012	00003	31014	10000	00100	(01)0010	010?0	01110	10040
Beiyanerpeton	1????	11?13	?0104	?1011	?0203	??11	011?0	1111?	?00{1345}{01}
Pangerpeton	?????	?1?1?	41105	?10??	???1?	??11	000?0	1111?	?0?4{01}
Ymeria	?????	??0?{12}	200?{012}	10?00	?0100	0?000	0????	0???0	0{01}???
Densignathus	?????	?????	1????	?????	?????	?????	?????	?????	?????
Chelotriton	1?0?4	01014	40104	1100?	??13	???1{01}	111?0	0111?	10042
Palatinerpeton	????1	?000?	?1012	11100	11100	01110	010?0	01110	12???
Glanochthon	0(012)0?1	00003	??0?2	10100	00100	(01)0010	010?0	01100	02?{34}?
Archaeovenator	000{23}4	01004	42??1	0??01	11102	10130	010?0	00?01	1?1?1
Platyoposaurus	00011	00003	3?012	11100	10101	01010	000?0	00101	01050
Trihecaton	?????	?????	4????	?????	?????	?????	?????	?????	?????
Erpetosaurus	0(12)0?0	0000 {123}	{04}1012	01100	01100	00110	110?0	01100	11?10
Mordex	020?2	00?04	?1?13	???0?	1??0?	??10	?1??0	01110	12???

	91–95	96–100	101–105	106–110	111–115	116–120	121–125	126–130	131–135
Branchiosaurus	020??	00004	????3	???0?	????{012}	?????	?????	?11??	1????
Pholidogaster	00??1	00001	2????	?????	?????	?????	?????	?????	?1???
Palaeoherpeton	02031	00003	????{12}	?????	?????	???10	?01?0	00?0?	??01{01}
Neopteroplax	?{01}0?1	00004	3211{12}	01100	01100	000{01}0	????0	00?00	02???
St. Louis tetrapod	?????	0???{123}	2??11	??1?0	???0{01}	0???0	?1???	??00?	?????
Parrsboro jaw	?????	?????	3????	?????	?????	?????	?????	?????	?????
Elginerpeton	?????	????1	0????	?????	?????	?????	?????	?????	?????
Australerpeton	01011	00003	{23}?014	10100	00200	10000	010?0	01000	0104{02}
Quasicaecilia	020{03}4	00?14	????3	?????	??{01}0 {23}	???01	011??	00???	???32
Casineria	?????	?????	?????	?????	?????	?????	?????	?????	?????
Llistrofus	01010	01114	42112	?1100	1110?	???10	010?0	00000	0?131
Bystrowiella	020?3	00?04	4????	0????	?????	?????	?????	?????	?????
Coloraderpeton	000?0	01?0?	{01}?10{12}	?0???	00?00	10010	000?0	0??0?	?0?12
Pseudophleg.	02??0	01?0?	????{123}	?????	?????	???{01}0	000?0	0000?	?????
Perittodus	?????	????{123}	?????	?????	?????	?????	?????	?????	?????
Diploradus	02???	00?0?	????{01}	?????	?????	???00	??0??	0????	?????
Aytonerpeton	?????	????{12}	{12}{01}011	01100	11101	01100	?1?10	0?000	01???

	136–140	141–145	146–150	151–155	156–160	161–165	166–170	171–175	176–180	181–185
Eusthenopteron	00??0	0000?	10000	20000	00000	10000	00010	00100	0?0??	00000
Panderichthys	00??0	00001	00000	00000	000?0	00000	00010	00100	01000	00000
Ventastega	00???	?100?	00011	01000	10000	00000	00010	00100	01000	00010
Acanthostega	00???	?100?	00110	01000	10010	10010	00110	01100	11000	00010
Ichthyostega	00??0	0100?	00101	11000	10000	00010	00110	01100	11000	00010
Tulerpeton	?????	?????	?????	0000?	????0	{01}0???	?????	?????	?????	?00??
Colosteus	1????	?2???	0????	1010?	?????	?????	?0?10	01?00	1??00	?0001
Greererpeton	10110	12000	00101	10101	10010	20010	00010	01000	10000	10001
Crassigyrinus	00101	01000	00101	00001	00010	20010	00101	01010	?????	00001
Whatcheeria	?????	?2??0	00011	00001	10010	10010	00110	01100	11021	00010

	136–140	141–145	146–150	151–155	156–160	161–165	166–170	171–175	176–180	181–185
Baphetes	00000	12000	00101	0000?	?0?10	?0010	?????	0?0?0	?0??0	10000
Megalocephalus	000?0	12000	00001	00001	20110	20010	00111	01110	11100	10000
Eucritta	00101	02000	0????	?000?	?????	?????	?????	?????	?????	?00?0
Edops	10010	02011	01???	00000	10110	20010	10101	01010	10110	10000
Chenoprosopus	10000	?2001	0????	?000?	?0110	20010	?0???	0???0	?????	?0010
Cochleosaurus	12010	02001	11???	00000	20110	20011	?0101	01010	10110	10010
Isodectes	10000	02010	2????	0000?	?0110	21010	?????	????0	10121	10001
Neldasaurus	10000	02010	0????	0000?	?0110	20010	?????	?????	?????	10001
Trimerorhachis	12000	02011	11???	00000	?0110	20010	?0111	0(01)010	00100	10001
Balanerpeton	12010	0201{12}	01???	00000	10110	20010	10101	01010	101??	10001
Dendrerpetidae	12010	020(01) (12)	0????	?000?	?0110	20010	?????	????0	?0???	10001
Eryops	10010	02001	01???	00000	10110	20010	101(01)1	01(01)10	10121	10010
Acheloma	10110	0201{01}	01???	0000?	{12}???0	?1010	?0101	?????	?????	10010
Phonerpeton	10000	02001	01???	00000	20110	21010	10101	01010	10121	10010
Ecolsonia	100?0	02011	01???	00000	10110	21010	10101	01010	10121	10000
Broiliellus brevis	10011	02011	0????	?000?	????0	?1???	?????	?????	?????	10000
Amphibamus	11000	02111	0????	?000?	?0??0	??010	?0???	0???0	?????	1?200
Doleserpeton	02000	02111	01???	2000?	?0110	21010	1????	01??0	11121	11200
Eoscopus	12???	??0??	01???	0000?	?0110	2????	?????	????0	?0???	?0000
Platyrhinops	10000	02102	01???	00000	10110	20010	10101	01010	101{12}?	10201
Micromelerpeton	10000	02011	01???	00000	10110	21010	10???	01010	10121	100?0
Apateon	10000	02111	0????	?000?	?0??0	??010	?????	?????	?????	10000
Leptorophus	00000	02011	01???	???00	10110	21010	10001	01010	10121	10000
Schoenfelderpeton	10000	02011	{012}1???	00000	?0110	21010	?????	????0	0??21	10000
Albanerpetidae	??0?0	0?01{12}	31???	2000?	?1??1	??1??	?1???	1???1	?????	00000
Eocaecilia	000?0	02???	21???	2000?	?1??1	??1??	?1???	1???1	?????	11200
Karaurus	10000	02112	3????	2?00?	?1??0	2?1??	?1???	1???1	?????	?????
Triadobatrachus	100?0	02?11	0????	2??1?	?1???	??1??	?1???	1???1	?????	?????
Valdotriton	10000	02012	31???	2000?	?1??1	??1??	?1???	1???1	?????	?1?00
Caerorhachis	?1001	02000	00100	00001	101?0	20010	00101	01010	10110	10001

	136–140	141–145	146–150	151–155	156–160	161–165	166–170	171–175	176–180	181–185
Eoherpeton	?0101	020?1	1????	?0001	20110	20010	00101	01010	101?0	10000
Proterogyrinus	0{12}101	02001	0????	00001	{12}0?10	20010	?0?0?	01010	1010?	10010
Archeria	0{12}101	02001	00001	(02)0001	20110	20010	00101	01010	10111	10000
Ph. attheyi	01101	02001	0????	20000	10110	20010	10101	01010	10100	10010
Anthracosaurus	0?101	02000	0????	(12)000?	10110	2?010	101??	01010	10100	?0010
Ph. scutigerum	0{12}101	02000	00001	00001	20110	20010	00101	01010	10111	10000
Bruktererpeton	?????	?????	0????	?????	?????	?????	?????	?????	?????	?0010
Gephyrostegus	01111	0?000	01???	00001	20110	20010	00001	00010	101{12}1	10000
Solenodonsaurus	00??0	0????	0????	2000?	????0	??010	?????	?????	?????	?0000
Kotlassia	00121	02002	11???	20001	20110	20010	00111	01110	11121	000?0
Discosauriscus	02120	02000	11???	20001	20110	21010	00101	01010	10121	10010
Seymouria	00121	02000	11???	20001	20110	21010	00101	01010	10121	10010
Diadectes	00121	0(12)000	11???	10001	?1??0	21010	01???	1???0	11121	10210
Limnoscelis	0{12}101	0{12}000	11???	10001	21??0	21010	01???	01010	10110	10010
Captorhinus	00111	02000	11???	1000?	?1??0	21010	01???	1???0	11121	10010
Paleothyris	00111	02000	1????	20001	?1??0	21010	0????	????0	11121	10010
Petrolacosaurus	00111	02000	11???	20001	?0110	21010	01???	1???0	11111	10010
Westlothiana	000?1	02000	0????	2000?	????0	??0?0	?????	?????	?????	?0000
Batropetes	00000	02112	41???	20001	?1??0	21010	11???	1???1	?????	00000
Tuditanus	00010	02010	0????	?000?	????0	?1010	?????	?????	?????	?0010
Pantylus	00020	02010	11???	10001	?0110	21010	01???	1???0	00021	00010
Stegotretus	00020	02000	2????	1000?	????0	?1010	?????	????0	0????	?0010
Asaphestera	??0??	?2???	1????	2000?	????0	?1010	?????	?????	?????	?0000
Saxonerpeton	00010	02000	1????	2000?	????0	?1???	?????	?????	?????	?0010
Hapsidopareion	?00?0	0211?	{12}????	20001	?1??0	21011	0????	?????	?????	00010
Micraroter	00010	12(01)01	2????	20001	?0110	21010	0????	?????	?????	00001
Pelodosotis	02020	12010	2????	?000?	????0	?1010	?????	?????	?????	?0000
Rhynchonkos	02010	02011	21???	2000?	????0	21010	?????	????0	11110	00000
Cardiocephalus	00020	02(01)00	2????	20001	?0110	21011	?????	?????	?????	00100
Euryodus	00010	0200(02)	2????	20001	?1??0	21011	0????	?????	?????	000(01)0

	136–140	141–145	146–150	151–155	156–160	161–165	166–170	171–175	176–180	181–185
Microbrachis	00020	02102	11???	20001	10110	21010	00101	01010	10120	10000
Hyloplesion	100?0	02011	1????	2000?	?0?10	?1???	?????	?????	?????	?0010
Odonterpeton	000?0	02001	2????	2000?	?0??0	?????	?????	?????	?????	?0000
Brachydectes	00000	02?01	41???	2000?	?1??0	21010	?1???	1???0	11121	00000
Acherontiscus	?????	?????	1????	2000?	????0	?1???	?????	?????	?????	?00??
Adelospondylus	00?0?	0????	1????	2000?	?0??0	?1010	?????	?????	?????	?00?0
Adelogyrinus	?????	?{12}???	1????	2000?	?0010	?1010	?????	?????	?????	?0000
Dolichopareias	?????	?????	?????	?????	?????	?????	?????	?????	?????	?000?
Scincosaurus	00000	02?11	3????	20?0?	?????	?????	?????	?????	?????	?00?0
Keraterpeton	?????	?????	2????	2000?	????0	?1010	?????	?????	?????	?000?
Batrachiderpeton	000?0	0200?	{23}1???	20001	?1??0	21010	01???	1???0	10000	00000
Diceratosaurus	00???	?????	{34}????	2000?	????0	?10??	?????	????0	1?0??	?00?0
Diplocaulus	00000	02002	{23}????	20001	?0110	21110	0????	?????	?????	00000
Diploceraspis	00000	02002	{23}1???	20001	?1??0	21111	01???	1???0	11010	10000
Ptyonius	10010	02000	1????	2000?	????0	?1010	?????	?????	?????	?0000
Sauropleura	00000	02001	01???	20001	?1??0	?1010	0????	????1	?????	10000
Urocordylus	?????	?????	{012}????	2000?	????0	?1010	?????	?????	?????	?000?
Lethiscus	00??0	0100?	1000?	20001	21??0	01010	001?1	?????	?????	10000
Oestocephalus	00000	02012	0????	?000?	?1??0	21010	0????	?????	?????	1?000
Phlegethontia	00??0	??0??	{23}1???	2?00?	?1??1	??1??	?1???	1???1	?????	??000
Ariekanerpeton	01120	02000	11???	2000?	20110	21010	?0101	01010	10120	10010
Leptoropha	?????	????0	?????	?000?	?????	?????	?????	?????	?????	?0000
Microphon	00??0	02000	?????	2000?	?0?10	?1010	?????	?????	?????	?0000
Capetus	1????	?200?	0????	??00?	?0?10	2?010	?????	????0	?????	?0011
Notobatrachus	10000	02111	21???	2001?	?1??1	??1??	?1???	1???1	?????	?020?
Vieraella	?????	???1{12}	2????	2001?	?1??1	??1??	?1???	1???1	?????	?1?0?
Orobates	001{12}1	02000	2????	10001	?1??0	21010	0????	1???0	11121	10210
Ossinodus	?????	??00?	0????	0??0?	?00??	??01?	?????	?????	?????	?001?
Pederpes	00100	0{01}000	0????	???0?	??0??	10???	?????	?0???	?????	?0?10
Silvanerpeton	00101	02000	0????	?0001	?0110	?0010	?????	0???0	10100	10000

	136–140	141–145	146–150	151–155	156–160	161–165	166–170	171–175	176–180	181–185
Tseajaia	00?20	01000	1????	20001	21??0	21010	0????	01010	10120	10010
Utegenia	02120	02000	01???	2000?	?0110	21010	?0101	01010	10110	10010
Gerobatrachus	?????	???1?	{01}????	?????	????0	2?0??	?????	?????	?????	??00?
Chroniosaurus	1{12}101	02000	0????	2000?	{12}0110	2001?	00111	01010	111??	10000
Micropholis	10000	02111	0????	?0000	?0110	21010	?????	????0	11121	10000
Nigerpeton	10110	02010	01???	0000?	101?0	?????	101?1	01?1?	???21	?0000
Saharastega	10100	02011	1????	???0?	101??	?????	10101	0101?	?????	?0000
Iberospondylus	?0?10	0?0??	0????	20001	{12}0110	21010	?0101	01010	10111	10000
Tungussogyrinus	10000	0??12	{012}????	???0?	?????	?????	?0100	?????	?????	?0000
Utaherpeton	?????	?????	{012}????	??00?	?????	?????	?????	?????	?????	??000
Caseasauria	?????	??000	1????	20001	?1??0	21010	?????	????0	???21	?0010
Goreville micro.	?0000	02002	1????	?0?0?	?????	?????	?????	?????	?0???	?0?0?
Sparodus	?????	?????	3????	???0?	?????	?????	?????	????0	0????	?0010
Liaobatrachus	00100	12(01)11	11???	2?01?	?1??1	??1??	?1???	1???1	?????	?0?0?
Acanthostomatops	02000	02012	1????	?????	?????	?????	?????	?????	?????	?00??
Deltaherpeton	?????	?????	00???	1010?	100?0	?00?0	?00?1	01010	101??	?00?1
Karpinskiosaurus	02121	02000	11???	20000	{01}0110	21010	0????	????0	1??21	10010
Carrolla	10000	02111	4????	2?00?	????0	210?0	0????	?????	?????	0?{02}00
NSM 994 GF 1.1	0????	??0??	0????	?????	?????	?????	?????	?????	?????	??0??
Crinodon	00010	02002	1????	?000?	????0	?10?0	?????	????0	0????	??00?
Sigournea	?????	?????	?0???	0000?	20110	21010	00110	01100	11010	100??
Doragnathus	?????	?????	?1???	20001	{12}0110	20010	00110	01100	11000	10000
Spathicephalus	0001?	?2011	00011	2000?	{12}0??0	?????	?0111	?????	?????	?0000
Metaxygnathus	?????	?????	?011?	01000	000?0	10000	000?0	00?00	0?0{01}0	000??
Sclerocephalus	10000	0201(12)	01???	00000	00110	20011	00101	01010	10121	10010
Chelidерpeton	?????	?????	?????	??00?	????0	?0???	?????	?????	?????	?00?1
Archegosaurus	10101	02002	01???	00000	10110	20010	00101	01010	10111	10010
Konzhukovia	101{01}0	02001	0????	?????	?????	?????	?????	?????	?????	?000?
Lydekkerina	10100	02002	11???	00000	{12}0010	20010	1000(01)	00010	10(01)21	000(01)0
Beiyanerpeton	10?00	020??	31???	2?00?	?1??1	??1??	?1???	1???1	?????	?0?00

	136–140	141–145	146–150	151–155	156–160	161–165	166–170	171–175	176–180	181–185
Pangerpeton	10001	02011	1????	?000?	?1??1	?????	?????	????1	?????	?????
Ymeria	?????	?????	?01(01)1	0100?	?00?0	100?0	00010	00100	01000	?0010
Densignathus	?????	?????	?0100	01001	100?0	10010	00001	00010	01000	000??
Chelotriton	000?0	02??{12}	01???	2000?	?1??0	211??	?1???	1???1	?????	0??00
Palatinerpeton	?????	?????	11???	00000	20110	21010	10101	01110	11121	1001?
Glanochthon	12100	02012	01???	00000	101?0	200?0	10???	????0	10(01)21	10011
Archaeovenator	?0101	0?000	11???	20001	?1??0	21010	01???	????0	11121	10000
Platyoposaurus	12111	02011	01???	(01)0001	201?0	210?1	10101	01010	1012?	10000
Trihecaton	?????	?????	?????	??00?	?????	??0??	?????	????0	???21	?0?00
Erpetosaurus	10120	0{12}002	0????	0010?	?0??0	?00?0	?????	?????	?????	?000?
Mordex	11010	0?011	{01}????	0??0?	????0	??0??	?0101	01010	101??	?0010
Branchiosaurus	10010	0?012	{012}????	?000?	????0	??0?0	?????	?????	?????	?0000
Pholidogaster	?????	?????	{01}????	{01}010?	?0?10	?00??	?????	?????	?????	100?1
Palaeoherpeton	??101	02100	0????	?????	?????	?????	?????	?????	?????	?00??
Neopteroplax	??1?1	02?00	0????	?0001	?0110	20010	?????	????0	1?12?	10?10
St. Louis tetrapod	?????	?????	{0123}0???	?0101	?0010	?????	00?0?	?????	?????	?00?0
Parrsboro jaw	?????	?????	?0100	0??00	001??	?????	00101	01010	1011?	100??
Elginerpeton	?????	?????	?0111	0000?	00000	10000	00010	00100	0?0??	000??
Australerpeton	10110	02011	01???	(01)0001	{12}0110	?10?0	00111	01110	111{12}1	10000
Quasicaecilia	?00?0	0211{12}	4????	?????	?????	?????	?????	?????	?????	?????
Casineria	?????	?????	?????	?????	?????	?????	?????	?????	?????	?????
Llistrofus	10000	02112	21???	2?00?	?1??0	21011	0????	?????	?????	00010
Bystrowiella	?????	?????	1????	?????	????0	??0??	?????	?????	?????	?00??
Coloraderpeton	001{01}1	01000	0000?	20000	01??0	{01}1010	001?1	01?10	1?100	000?0
Pseudophleg.	001{01}?	01000	3????	?000?	?1??0	{01}10?0	?????	?????	?????	?00??
Perittodus	?????	?????	?011?	?0000	{12}00?0	?00?0	000?0	00?00	1?000	?00?0
Diploradus	00?10	02011	?000?	1??00	{12}00?0	?????	00110	01100	110?0	0??00
Aytonerpeton	?????	?????	?010?	1000?	00010	?????	00011	01110	111??	?0001

	186–190	191–195	199–200	201–5	206–210	211–215	216–220	221–225	226–230	231–235
Eusthenopteron	00000	00000	00000	000?0	00000	00010	01011	00000	0000?	00000
Panderichthys	00000	00000	00000	00000	01000	00011	01011	0000?	?????	???00
Ventastega	00000	00011	00000	100??	?????	?????	?????	????1	??00?	?????
Acanthostega	00000	00011	00000	10000	00001	00000	10021	10011	00000	11020
Ichthyostega	00020	00012	10001	01??0	0?001	00100	10021	12011	01100	01000
Tulerpeton	000?0	0?10{12}	00001	01011	00011	10001	101?1	1101?	?????	11020
Colosteus	000{01}0	???00	010??	?????	?????	?????	???1?	1????	?????	?????
Greererpeton	00000	00100	01000	01110	01011	10000	10110	12010	01001	11120
Crassigyrinus	00010	0?1?1	000??	???10	01001	{12}000?	10011	100?1	0?00?	11120
Whatcheeria	00020	00(01)02	10?1?	01100	01011	10000	10121	??011	01000	0?0?1
Baphetes	00010	0011{12}	?????	???00	01011	2000?	1112?	1?0?1	0100?	????0
Megalocephalus	00010	?????	?????	?????	?????	?????	?????	?????	?????	?????
Eucritta	00020	0?11(01)	0000?	??1?0	??011	200{01}?	1?12?	11011	0??0?	???20
Edops	00020	?????	?????	????1	1?111	21110	1112?	12011	??101	0?1?0
Chenoprosopus	00010	???0?	??0??	?????	?????	?????	?????	????1	0?10?	??1??
Cochleosaurus	00020	01110	010?0	??1??	?????	?1?1?	1?12?	12011	0100?	1?120
Isodectes	000{12}0	0?111	000??	???1?	11??{01}	2111?	?1120	12011	0??0?	???20
Neldasaurus	00000	???00	0100?	0?111	?1111	21?1?	11120	12011	0?00?	{12}?1??
Trimerorhachis	00000	???10	010??	???11	11111	21?10	11120	12011	0?10?	11120
Balanerpeton	00000	01110	0000?	??1??	1?012	?111?	11120	11010	0100?	1?020
Dendrerpetidae	00000	01110	00000	011??	1?011	2111?	11120	11011	01101	2?120
Eryops	00000	01(01)10	11101	01111	11111	21110	11120	12011	0(01)101	21120
Acheloma	000{12}0	011??	???01	01111	11111	21110	01120	??011	11101	20110
Phonerpeton	00020	011??	?????	01?11	11?11	2111?	???2?	??01?	????1	?01??
Ecolsonia	00020	01110	10001	01111	11111	211{12}0	1?120	??010	?1001	2?1??
Broiliellus brevis	00000	011??	???0?	??1??	??112	?1?11	11120	????1	01001	{12}????
Amphibamus	00000	01110	000??	?11??	1?112	?1?21	11120	1?0??	0100?	{12}??20
Doleserpeton	00000	01110	?1000	011?0	1?112	?1121	11120	11011	01001	20120
Eoscopus	00000	??110	00000	0????	??11?	?1?2?	11120	12011	01001	101?0
Platyrhinops	00000	01110	0000?	????0	1?111	21?21	11120	12011	01?0{12}	20120

	186–190	191–195	199–200	201–5	206–210	211–215	216–220	221–225	226–230	231–235
Micromelerpeton	00010	???10	0000?	????1	??112	?1???	??120	110?0	0??0?	?????
Apateon	00000	01110	10000	011??	1???2	?1?2?	11120	11011	0?00?	???20
Leptorophus	000(01)0	???10	000??	?????	??112	?1?2?	??120	110?1	0??0?	???2?
Schoenfelderpeton	00000	????0	?00??	?????	??112	?1?1?	??1{12}0	110??	?????	?????
Albanerpetidae	0002(12)	?????	???11	??1?0	1?112	?1121	11120	11011	?????	???20
Eocaecilia	00000	?????	???11	011?0	1?112	?1121	11110	1101?	?????	20110
Karaurus	?????	?????	???0?	??110	1?112	?1?21	11120	11010	0??0?	???20
Triadobatrachus	?????	011??	???1?	011?0	1?112	?112?	?1120	12011	?001?	00?20
Valdotriton	0002?	?????	???01	011?0	1?112	?1?21	11120	1201?	?????	2?120
Caerorhachis	0000?	?????	?????	?????	?????	?????	?????	??011	0110?	1?1?0
Eoherpeton	00020	??1??	???01	01?10	01011	10?00	11121	????1	01101	??1?0
Proterogyrinus	10000	0?111	00000	01110	01011	20100	1?121	11011	01100	{12}1120
Archeria	10000	00112	01000	01111	01011	20110	11121	12011	11101	21120
Ph. attheyi	10000	?????	?????	?????	?????	?????	?????	?????	?????	111??
Anthracosaurus	00020	?????	?????	?????	?????	?????	?????	?????	?????	?????
Ph. scutigerum	10000	0?1?{12}	??00	010??	0?0?1	{12}??10	1112?	1201?	?????	?????
Bruktererpeton	000{01}0	??1??	0????	01?10	11011	20111	11120	1201?	?????	{12}0120
Gephyrostegus	00000	01112	00001	01110	11011	20111	11120	12011	01101	10120
Solenodonsaurus	00010	011?2	100??	????1	1?011	21?10	11120	1?00?	?????	?????
Kotlassia	00010	0?1?2	11000	011?1	??111	21110	11120	??011	01101	21120
Discosauriscus	00021	01102	10010	0100?	11011	21?1?	10120	110?1	1?101	?0020
Seymouria	00020	01102	?1011	01111	11011	21110	10120	12011	11101	21020
Diadectes	00020	01102	10011	01101	11011	21110	10120	12011	11101	21120
Limnoscelis	00020	011?2	10001	01?01	11011	21100	10120	12011	11101	11010
Captorhinus	00020	???12	10001	01111	11011	21110	11120	12010	01?01	11120
Paleothyris	00010	01112	10001	01?11	11011	21121	11120	12010	01001	10120
Petrolacosaurus	00010	01112	10001	01111	11011	21121	11120	12010	11001	{12}0120
Westlothiana	0001{01}	??1??	???0?	?1?11	11011	21111	11110	12011	11001	10120
Batropetes	00032	0?111	1000(01)	011?0	1?012	?1111	11120	12011	(01)?001	20100
Tuditanus	00020	01112	000??	????1	??01?	?1?11	11120	1{12}0?1	01101	{12}01?0

	186–190	191–195	199–200	201–5	206–210	211–215	216–220	221–225	226–230	231–235
Pantylus	001?0	01102	00000	01100	11011	21111	11120	12010	0100?	101?0
Stegotretus	001?0	????{12}	10000	?11??	??1??	?1?1?	???20	12011	0??01	10110
Asaphestera	00020	??11	10001	11??1	??011	21?1?	??120	??0?1	0?00?	???{12}?
Saxonerpeton	00020	01112	10001	??1?0	1?012	?1111	11120	12011	0100?	001{12}0
Hapsidopareion	00020	?????	?????	?????	?????	?????	?????	????1	?????	?????
Micraroter	00020	01102	1001?	?11??	?????	?????	?????	????1	01001	{12}01??
Pelodosotis	00020	01112	1000?	1?1?0	1?011	21111	11120	12011	0??01	{12}0120
Rhynchonkos	00020	?????	?????	?????	?????	?????	?????	?????	?????	?????
Cardiocephalus	001??	?????	?????	?????	?????	?1?{12}?	?1?2?	1101?	???01	?????
Euryodus	001?0	??1?2	10001	?1?10	11111	21111	11120	??011	01?01	{12}0120
Microbrachis	0002?	01112	100??	????0	1?012	?1111	11110	12011	01001	{12}0120
Hyloplesion	00020	011?{12}	1??01	????0	1?012	?1111	11110	12011	0100?	10120
Odonterpeton	00020	011??	?????	??1?0	?????	?1?11	11110	110??	?????	?????
Brachydectes	00030	01110	100??	??1?0	??112	?1?1?	11110	11000	0?00?	{12}0120
Acherontiscus	001?0	????0	010??	?????	?????	?????	?????	?????	?????	?????
Adelospondylus	110{012}0	????0	?????	?????	?????	?????	?????	?????	?????	?????
Adelogyrinus	11010	0?1?0	010??	??1??	?????	?????	???0?	?????	?????	?????
Dolichopareias	11000	?????	?????	?????	?????	?????	?????	?????	?????	?????
Scincosaurus	00031	01100	10001	?11?1	1?01?	?1111	1?110	12011	0?002	{12}0120
Keraterpeton	00030	11100	010??	??1?0	??11?	?0?01	10110	0101?	0?00?	???20
Batrachiderpeton	00030	11100	010??	?????	?????	?????	?????	?????	?????	?????
Diceratosaurus	00030	11100	010??	??1?0	??11?	???01	10110	01011	0?00?	???20
Diplocaulus	00020	11100	01010	111??	1?011	21?1?	11110	1201?	?????	20020
Diploceraspis	00030	??00	010??	??1??	?????	?????	?????	?????	?????	?????
Ptyonius	00020	01110	0100?	?11?0	??11?	?011?	11110	11011	0?002	???20
Sauropleura	00020	01110	0100?	?11?1	??11?	?0110	11110	11011	1?002	???20
Urocordylus	00020	01110	0100?	??1?0	??01?	?011?	10110	11011	0?0?2	{02}1120
Lethiscus	0?020	?????	?????	??1??	?????	?????	???0?	?????	?????	?????
Oestocephalus	(01)0020	01???	?????	??1??	?????	?????	???0?	?????	?????	?????
Phlegethontia	00020	01???	?????	??1??	?????	?????	???0?	?????	?????	?????

	186–190	191–195	199–200	201–5	206–210	211–215	216–220	221–225	226–230	231–235
Ariekanerpeton	00020	01102	1001?	0???1	11011	21?1?	10120	11011	1?10?	???20
Leptoropha	00023	?????	?????	?????	?????	?????	?????	?????	?????	?????
Microphon	00023	?????	?????	?????	?????	?????	?????	?????	?????	201??
Capetus	000{01}0	????0	000??	?????	?????	?????	?????	?????	?????	?????
Notobatrachus	00000	011??	???10	011?0	1?112	?1?21	11120	12111	0?01?	00?2?
Vieraella	0002?	011??	???1?	??1?0	??112	?1?21	11120	1211?	???1?	????2?
Orobates	00020	0110?	10?11	01111	11011	21100	11120	12011	10101	20120
Ossinodus	00010	00112	?11?1	?1?0?	0101?	0000?	?01{12}1	??0?1	0?10?	200?0
Pederpes	0002?	0111{12}	1011?	0??1?	01011	1000?	1012?	110?1	0?00?	00021
Silvanerpeton	00010	0?111	0000?	????0	??011	20?1?	11121	120?1	0??0?	????2?
Tseajaia	00020	01102	1?011	011?1	1?011	2111?	10120	12011	11101	21120
Utegenia	00020	011?2	1001?	?????	??01?	???1?	1?120	110?1	1?10?	???20
Gerobatrachus	00000	011??	?????	?????	?????	?1?2?	?1?2?	??11	0??0?	?????
Chroniosaurus	00010	????2	000??	?????	1?011	211{12}?	1?12?	????1	0110?	????0
Micropholis	000(01)0	01110	0?010	??1??	??1??	?1?1?	?112?	12011	01001	10110
Nigerpeton	00000	?????	?????	?????	?????	?????	?????	?????	?????	?1???
Saharastega	00000	?????	?????	?????	?????	?????	?????	?????	?????	?????
Iberospondylus	00010	?????	?????	?????	?????	?????	?????	?????	?????	?????
Tungussogyrinus	00022	?????	?????	?????	?????	?1?2?	?1?2?	100?1	0101?	???20
Utaherpeton	00020	????0	100??	?????	1?01?	?111?	?1110	12011	?????	???20
Caseasauria	00020	??1?2	11001	0???1	1?011	21?11	11120	??11	??101	{12}1120
Goreville microsaur	0?02?	?????	10???	?????	??0??	?1?1?	111??	?????	?????	{12}01{12}?
Sparodus	00020	01112	1??01	????0	1?11?	?1111	11120	12011	01001	2????
Liaobatrachus	00000	0110?	???10	011?0	1?112	?1121	11120	12111	?101?	00?2?
Acanthostomatops	00010	01110	1?0??	??1?1	1????	?1?1?	?1?2?	120??	01?0?	???00
Deltaherpeton	00000	?????	?????	?????	?????	?????	?????	?????	?????	?????
Karpinskiosaurus	000(12)0	?????	?????	?????	?????	?????	?????	?????	?????	?????
Carrolla	0003{02}	?????	?????	?????	?????	?????	?????	?????	?????	?????
NSM 994 GF 1.1	000{12}0	0?11?	?????	????0	0?0??	?0100	1112?	1201?	?????	?????
Crinodon	00020	?????	?????	?????	?????	?????	?????	?????	?????	?????

	186–190	191–195	199–200	201–5	206–210	211–215	216–220	221–225	226–230	231–235
Sigournea	00000	?????	?????	?????	?????	?????	?????	?????	?????	?????
Doragnathus	00000	?????	?????	?????	?????	?????	?????	?????	?????	?????
Spathicephalus	10000	?????	?????	?????	?????	?????	?????	?????	?????	?????
Metaxygnathus	000?0	?????	?????	?????	?????	?????	?????	?????	?????	?????
Sclerocephalus	00010	01110	01001	00111	11112	?111?	??120	12011	0?10?	?1120
Cheliderpeton	000{12}0	0111{01}	010??	??1??	?????	?1?1?	????2?	12011	???0?	?1??0
Archegosaurus	00000	01110	010??	??1??	?????	?1?1?	????2?	12011	1?10?	??120
Konzhukovia	00010	?????	?????	?????	?????	?????	?????	?????	?????	?????
Lydekkerina	00010	011(01)1	01000	00111	11111	21111	11120	12011	0?101	21110
Beiyanerpeton	000{012}0	?????	???0?	??1?0	1?11?	?1?21	11120	11011	01?1?	2?010
Pangerpeton	000{01}0	?????	??01	????0	1????	?1?2?	?1120	11011	0??0?	???20
Ymeria	00020	?????	?????	?????	?????	?????	?????	?????	?????	?????
Densignathus	000{012}0	?????	?????	?????	?????	?????	?????	?????	?????	?????
Chelotriton	??00?	?????	???0?	??1?0	?111?	?1?21	11110	1201?	???0?	?1?20
Palatinerpeton	000{12}0	011??	?????	?????	?????	?????	???{12}?	????1	0??0?	??1?0
Glanochthon	000(12)0	01110	000??	?????	?????	?1?1?	?1?2?	1{12}0?1	0??0?	????2?
Archaeovenator	000{12}0	????2	10?01	0????	?????	?1???	?1?2?	????1	?1?01	00??0
Platyoposaurus	00000	0?1?0	0?00?	??1??	?????	?????	???{12}?	?1011	0??0?	211??
Trihecaton	00020	????2	11?11	11?10	11?11	21111	?1120	12011	0??0?	1?12?
Erpetosaurus	00000	0?1?0	010??	?????	??1?1	{12}0?1?	?1?2?	1{12}011	0?0??	??1??
Mordex	00000	???1?	01???	?1???	0????	?1?1?	01120	1{12}011	11?0?	??120
Branchiosaurus	00010	???10	1?0??	?????	?????	?1?1?	?112?	1{12}011	???0?	???20
Pholidogaster	00020	??110	000??	?????	?????	???{01}?	????2?	1?0??	???0?	{12}??20
Palaeoherpeton	000{12}0	?????	?????	?????	?????	?????	?????	?????	?????	?????
Neopteroplax	??01?	?????	?????	?????	?????	?????	?????	?????	?????	?????
St. Louis tetrapod	000{012}0	?????	?????	?????	?????	?????	?????	?????	?????	?????
Parrsboro jaw	000{012}0	?????	?????	?????	?????	?????	?????	?????	?????	?????
Elginerpeton	00000	??1??	?????	?????	?????	?????	?????	????1	0?00?	??0?0
Australerpeton	00000	0?110	01001	0???1	1?1??	?1?1?	?1120	12010	1?101	2?120
Quasicaecilia	?????	?????	?????	?????	?????	?????	?????	?????	?????	?????

	186–190	191–195	199–200	201–5	206–210	211–215	216–220	221–225	226–230	231–235
Casineria	?????	001??	??11	??1??	??01?	?1?1?	11?20	1?0?1	0??0?	?????
Llistrofus	00020	?????	?????	?????	?????	?????	???{12}?	?201?	?????	?????
Bystrowiella	000?0	011?1	000??	????1	1?011	21110	?1120	?????	?????	1012?
Coloraderpeton	000{12}0	?????	?????	?????	?????	?????	???0?	?????	?????	000{12}0
Pseudophlegethontia	000?0	01???	?????	?????	?????	?????	???0?	?????	?????	000?0
Perittodus	00020	?????	?????	?????	?????	?????	???{12}?	?10??	?????	00020
Diploradus	??01?	?????	?????	?????	?????	?????	?????	?????	?????	??01?
Aytonerpeton	00000	?????	?????	?????	?????	?????	?????	????0	0??0?	??0?0

	236–240	241–245	246–250	251–255	256–260	261–265	266–270	271–275	276, 277
Eusthenopteron	0000?	???0?	00000	?0?00	01010	00001	00010	00000	00
Panderichthys	00???	???0?	00000	0??00	0000?	?0?01	000?0	00000	00
Ventastega	?????	?????	?????	?????	?????	?????	?????	?????	?0
Acanthostega	00100	00000	00000	00?00	01010	00001	00011	00000	10
Ichthyostega	00000	00001	11000	???00	01010	00001	00001	00000	10
Tulerpeton	11010	111??	?????	?????	???10	0??01	0????	????0	1?
Colosteus	?????	?????	?????	???00	???10	000?1	1?0?{12}	??001	3?
Greererpeton	01000	0?101	00110	00?00	01010	00001	1001{12}	00001	2?
Crassigyrinus	1110?	???0?	10000	0??00	???0?	?0?01	0?0?{12}	00000	{1234}?
Whatcheeria	0101?	???01	11000	???00	00010	10001	0001{12}	00000	{1234}?
Baphetes	0110?	?????	??1?0	???00	0?0??	????1	?00??	??0??	{1234}?
Megalocephalus	?????	?????	?????	?????	?????	?????	?????	?????	??
Eucritta	01???	???00	00000	?????	?????	?????	?????	?????	{1234}?
Edops	11???	?????	?????	0??00	?1?10	00001	0?0?{12}	?0000	{1234}?
Chenoprosopus	?????	?????	?????	????0	?????	?0?01	0?0?{12}	?0???	??
Cochleosaurus	01???	???00	00000	0??0?	???10	00001	0?01{12}	?10??	3?
Isodectes	01???	???00	1???0	???0?	?????	?0?0{01}	??01{12}	??0?0	31
Neldasaurus	?????	???0?	00000	???00	???10	00001	1?01{12}	00000	3?
Trimerorhachis	0110?	???01	10000	00?00	01010	00001	10012	01000	{23}1
Balanerpeton	01000	01100	00001	00?00	01010	00001	0001{12}	00000	31

	236–240	241–245	246–250	251–255	256–260	261–265	266–270	271–275	276, 277
Dendrerpetidae	01??0	01100	00001	???00	01010	00001	0001{12}	00000	{23}1
Eryops	01110	01100	01000	(01)0?00	01010	00001	00012	00000	31
Acheloma	01??0	0110?	00001	00000	01010	00001	0000{12}	00000	{23}?
Phonerpeton	?????	???0?	0000?	???00	01010	00001	00002	?0000	??
Ecolsonia	?1000	0??0?	00000	???00	01010	00001	00012	00000	{1234}?
Broiliellus brevis	?????	???0?	00000	???00	?????	?0001	0?0?{12}	?0000	??
Amphibamus	0100?	???00	00001	?0?00	01010	00001	00012	01000	31
Doleserpeton	0110?	???00	00001	0??00	01011	?0101	00012	01000	3?
Eoscopus	01000	01100	00001	00?00	01010	?0001	00012	01000	31
Platyrhinops	?1??0	?1100	00001	???00	01010	00001	00012	00000	31
Micromelerpeton	?1???	???00	00000	???00	???10	00001	0??12	00?00	31
Apateon	01???	???00	00001	???00	?????	?0?01	0?0?2	00000	31
Leptorophus	?1???	?????	?????	???00	?????	?0???	????{12}	?0000	3?
Schoenfelderpeton	?????	?????	?????	???00	?????	?????	0???{12}	?0?00	3?
Albanerpetidae	?1??0	01100	00001	11100	0?011	10102	0001{12}	01000	31
Eocaecilia	01??0	0??01	00001	11100	00011	10101	00012	11010	{1234}1
Karaurus	01???	???00	00001	1??00	010??	?011{02}	00012	010?0	31
Triadobatrachus	01000	0??00	00001	01100	?????	?010{02}	0?01{12}	?1000	?1
Valdotriton	01?0?	???00	00001	?1100	010??	?011{02}	00012	01010	31
Caerorhachis	01??0	11100	10000	?0?00	01011	00001	00012	00000	{1234}?
Eoherpeton	0110?	???0?	10000	0?000	???11	00001	1?00{12}	00000	??
Proterogyrinus	01100	11100	10000	00?00	01011	00001	10012	00000	2?
Archeria	01100	11100	10000	00?00	01011	10000	10012	00000	2?
Ph. attheyi	?????	???0?	10000	???00	01011	10000	1000{12}	?0000	??
Anthracosaurus	?????	?????	?????	?????	?????	?????	?????	?????	??
Ph. scutigerum	?????	???00	10000	0??00	???11	10000	1?00{12}	?0000	{1234}?
Bruktererpeton	01000	1??00	10000	???00	01011	00001	?001{12}	00000	2?
Gephyrostegus	01101	11100	10100	00?00	???11	00001	0?01{12}	00000	2?
Solenodonsaurus	?????	???00	10000	???00	???11	10101	0?01{12}	00000	{123}?
Kotlassia	01000	0?100	10100	01?00	01011	11101	000?2	0?000	{1234}1

	236–240	241–245	246–250	251–255	256–260	261–265	266–270	271–275	276, 277
Discosauriscus	0100?	???00	00000	00?00	01011	11?01	00012	00000	2?
Seymouria	01100	01100	00000	00?00	01011	11101	00012	00000	21
Diadectes	01001	11?00	10000	01?00	01011	11101	00012	00000	21
Limnoscelis	01100	0??00	10000	01?00	01011	11101	00012	00000	21
Captorhinus	01??1	11100	10000	01?00	01011	11101	00012	01000	21
Paleothyris	01001	11101	10000	01?00	0?011	10101	0001{12}	00000	21
Petrolacosaurus	01001	11100	10000	01?00	01011	11101	00012	00000	21
Westlothiana	11000	1110?	1000?	???00	01011	11101	0001{12}	01000	{123}1
Batropetes	01000	01100	10000	01100	0(01)0??	?010{02}	00012	?1000	31
Tuditanus	01001	11?01	10000	???00	?????	?010?	0??1{12}	?1?00	31
Pantylus	0???1	01100	10000	11?00	0?0??	?000{02}	00012	11000	3?
Stegotretus	0????	?????	?????	???00	?????	?000{02}	0?01{12}	11000	{1234}?
Asaphestera	?????	???0?	10000	???00	?????	?010?	0??1{12}	?1?00	{1234}?
Saxonerpeton	01000	01100	10000	??100	?????	?000?	0??1{12}	11?00	{1234}?
Hapsidopareion	?????	????0	?????	001??	?????	??0??	?????	?????	??
Micraroter	?????	???00	10000	01?00	01011	10001	00012	01000	??
Pelodosotis	0100?	???00	10000	0??00	???11	10001	0??1{12}	?1?00	{1234}?
Rhynchonkos	?????	?????	?????	?????	?????	?????	?????	?????	??
Cardiocephalus	?????	???00	10001	?1?00	???11	10101	0?01{12}	01000	{1234}?
Euryodus	0100?	?????	?????	(01)1?00	???11	10101	0?01{12}	01000	{1234}?
Microbrachis	01000	0??00	10000	?1?00	000??	?010{02}	00012	11000	41
Hyloplesion	01000	01100	10000	?1?00	000??	?000{02}	0001{12}	?1000	41
Odonterpeton	?????	???00	10001	??100	?????	?000{02}	0???{12}	11?00	4?
Brachydectes	01???	???01	10010	00?00	000??	?000{02}	1001{12}	11000	31
Acherontiscus	?????	?????	1???1	???00	00011	10000	100??	??00?	??
Adelospondylus	?????	???0?	10110	???0?	?????	?000{02}	1???{12}	1??00	??
Adelogyrinus	?????	???0?	10110	0??0?	?????	??00{02}	1???{12}	10?00	??
Dolichopareias	?????	?????	?????	?????	?????	?????	?????	?????	??
Scincosaurus	01??0	0010?	10000	?1011	111??	?010{02}	00012	11100	31
Keraterpeton	01???	???0?	10000	?1011	110??	?010{02}	01002	00100	31

	236–240	241–245	246–250	251–255	256–260	261–265	266–270	271–275	276, 277
Batrachiderpeton	?????	?????	?????	??011	110??	?010{02}	00002	00100	??
Diceratosaurus	01???	???0?	10000	?1011	110??	?010{02}	01102	00100	21
Diplocaulus	01???	???00	00000	11111	110??	?111{02}	01112	01?00	{23}1
Diploceraspis	?????	?????	?????	11111	110??	?011{02}	01102	01100	?1
Ptyonius	01???	???00	10000	?1?11	111??	?010{02}	00002	01100	31
Sauropleura	01???	0?10?	10000	01?11	111??	?010{02}	00002	001(01)0	31
Urocordylus	01???	???0?	10000	?1?11	111??	?010{02}	00002	01100	21
Lethiscus	?????	???11	00001	11100	?????	?010{02}	0??12	01??0	?1
Oestocephalus	?????	???21	00001	?1?10	?????	?010{02}	0?002	01110	?1
Phlegethontia	?????	???21	00001	01100	?????	?010{02}	0?002	01110	?1
Ariekanerpeton	?1???	???00	10000	???00	???11	?1?01	0?01{12}	00000	21
Leptoropha	?????	?????	?????	?????	?????	?????	?????	?????	??
Microphon	?????	?????	?????	?????	?????	?????	????{12}	?????	??
Capetus	?????	?????	?????	?????	?????	?????	?????	?????	??
Notobatrachus	?1???	???00	00101	11100	?????	?010{02}	0?01{12}	01000	31
Vieraella	?????	???0?	00101	1??0?	?????	?010{02}	0?01{12}	01000	31
Orobates	01000	11000	10000	0??00	01011	11?01	00012	01000	21
Ossinodus	1???0	???0?	00000	???00	010??	?000?	?001{12}	??0?0	{1234}?
Pederpes	0110?	???00	01000	?0?00	0??10	?0001	0?01{12}	00000	{1234}?
Silvanerpeton	?1??0	01100	10000	???00	01011	00001	000?{12}	??000	2?
Tseajaia	?1??0	01?0?	10000	01?0?	???11	11101	0?01{12}	00000	2?
Utegenia	?1???	???00	10000	???00	01011	1??01	000?{12}	00000	21
Gerobatrachus	?????	???00	00001	1?1??	???11	??001	0?0??	0????	{234}1
Chroniosaurus	01??0	0??0?	10?0?	?0?00	00011	10100	00012	0?000	{1234}?
Micropholis	?1??0	??101	?0001	?0?00	???10	00001	0?0?{12}	??000	3?
Nigerpeton	?????	?????	?1000	????0	?????	?00?0	???0{12}	?0??0	??
Saharastega	?????	?????	?????	?????	?????	?????	?????	?????	??
Iberospondylus	?????	???00	01000	00000	010??	?0001	00012	?00?0	??
Tungussogyrinus	0100?	?????	?????	?????	?????	?0?0?	????{12}	????0	31
Utaherpeton	0100?	???00	10000	???00	00011	?0102	000?2	01000	31

	**236–240**	**241–245**	**246–250**	**251–255**	**256–260**	**261–265**	**266–270**	**271–275**	**276, 277**
Caseasauria	01??1	01101	00000	1??00	0?011	10?01	0001{12}	?1000	{234}?
Goreville microsaur	?????	???0?	10000	00?00	01011	10001	00012	01000	{1234}?
Sparodus	?????	???00	10000	???0?	?????	?010{02}	0?01{12}	0?00?	{1234}?
Liaobatrachus	?1???	?1100	00101	1110?	?????	?010{02}	0?01{12}	01000	31
Acanthostomatops	?1???	???00	00001	???00	00010	00001	00012	00000	31
Deltaherpeton	?????	?????	?????	?????	?????	?????	?????	?????	??
Karpinskiosaurus	?????	?????	?????	010??	???11	11101	0?00{12}	01000	??
Carrolla	?????	?????	?????	?????	?????	?????	?????	?????	??
NSM 994 GF 1.1	?????	???00	1???0	00?00	???1?	10001	??00{12}	?00?0	??
Crinodon	?????	?????	?????	?????	?????	?????	?????	?????	??
Sigournea	?????	?????	?????	?????	?????	?????	?????	?????	??
Doragnathus	?????	?????	?????	?????	?????	?????	?????	?????	??
Spathicephalus	?????	?????	?????	?????	?????	?????	?????	?????	??
Metaxygnathus	?????	?????	?????	?????	?????	?????	?????	?????	??
Sclerocephalus	01000	01100	11100	00?00	01010	00001	00012	00000	31
Cheliderpeton	01000	01100	1?100	???0?	?????	?0001	????{12}	?????	{23}?
Archegosaurus	11000	01100	10100	00?00	01010	00001	0001{12}	00000	31
Konzhukovia	?????	?????	?????	?????	?????	?????	?????	?????	??
Lydekkerina	0100?	0??00	10100	0000?	?0?10	00001	0?0?{12}	00000	{234}?
Beiyanerpeton	0100?	???00	00001	?1100	010??	?011{02}	000?{12}	0?0?0	31
Pangerpeton	0100?	???01	?000?	??1??	?????	???1{02}	0?0??	?????	3?
Ymeria	?????	?????	?????	?????	?????	?????	?????	?????	??
Densignathus	?????	?????	?????	?????	?????	?????	?????	?????	??
Chelotriton	?1??0	01101	10000	11101	01???	?011?	00012	011?0	31
Palatinerpeton	0100?	?????	?????	?????	?????	?????	?????	?????	{1234}?
Glanochthon	?????	???0?	1?1??	?????	???10	00001	????{12}	??000	{1234}?
Archaeovenator	01001	11100	10000	01000	01011	10101	0001{12}	0?00?	{234}?
Platyoposaurus	?????	???01	11100	00?00	01010	00001	0001{12}	00000	3?
Trihecaton	?????	???00	10000	01100	00011	10101	0001{12}	010?0	{1234}?
Erpetosaurus	?1???	???00	00100	?????	???10	00001	???1{12}	?00??	{123}?

	236–240	241–245	246–250	251–255	256–260	261–265	266–270	271–275	276, 277
Mordex	01???	???00	?????	????0	0?01?	????1	????2	?????	31
Branchiosaurus	?1???	???00	?????	???00	?????	?????	????{12}	?????	31
Pholidogaster	?1??0	?11??	1???0	???00	01010	0?001	100?2	0?00?	{1234}?
Palaeoherpeton	?????	?????	?????	?????	?????	?????	?????	?????	??
Neopteroplax	?????	?????	?????	?????	?????	?????	?????	?????	??
St. Louis tetrapod	?????	?????	?????	?????	?????	?????	?????	?????	??
Parrsboro jaw	?????	?????	?????	?????	?????	?????	?????	?????	??
Elginerpeton	?????	?????	?????	????0	?????	?00??	???0{12}	??0??	??
Australerpeton	0110?	???00	10100	?0?00	01010	00001	0001{12}	00000	{1234}?
Quasicaecilia	?????	?????	?????	?????	?????	?????	?????	?????	??
Casineria	?????	???00	10000	???00	???11	?0001	0?01{12}	000?0	2?
Llistrofus	?????	???00	10000	???00	?????	?000{02}	0?0?{12}	1100?	{1234}?
Bystrowiella	?110?	???0?	10100	???00	?1?11	10100	0?01{12}	00000	{1234}?
Coloraderpeton	?????	???21	0????	111?1	?????	?010{02}	?????	00110	??
Pseudophlegethontia	?????	???21	00001	?110?	?????	?010{02}	0?0?2	01010	?1
Perittodus	?????	?????	?????	?????	?????	?????	?????	?????	{1234}?
Diploradus	?????	?????	?????	?????	?????	?????	?????	?????	??
Aytonerpeton	01???	?????	0????	?????	?????	?????	?????	?????	{1234}?
